# Use of standardised patients for healthcare quality research in low- and middle-income countries

**DOI:** 10.1136/bmjgh-2019-001669

**Published:** 2019-09-12

**Authors:** Ada Kwan, Benjamin Daniels, Sofi Bergkvist, Veena Das, Madhukar Pai, Jishnu Das

**Affiliations:** 1 School of Public Health, University of California Berkeley, Berkeley, California, USA; 2 McCourt School of Public Policy and School of Foreign Service, Georgetown University, Washington, District of Columbia, USA; 3 ACCESS Health International, New York City, New York, USA; 4 Department of Anthropology, Johns Hopkins University, Baltimore, Maryland, USA; 5 Department of Epidemiology & Biostatistics, and McGill International TB Centre, McGill University, Montreal, Quebec, Canada; 6 Center for Policy Research, Delhi, India

**Keywords:** standardized patients, quality of care, health care providers

## Abstract

The use of standardised patients (SPs)—people recruited from the local community to present the same case to multiple providers in a blinded fashion—is increasingly used to measure the quality of care in low-income and middle-income countries. Encouraged by the growing interest in the SP method, and based on our experience of conducting SP studies, we present a conceptual framework for research designs and surveys that use this methodology. We accompany the conceptual framework with specific examples, drawn from our experience with SP studies in low-income and middle-income contexts, including China, India, Kenya and South Africa, to highlight the versatility of the method and illustrate the ongoing challenges. A toolkit and manual for implementing SP studies is included as a companion piece in the online supplement.

**Summary box**

- The standardised patient (SP) methodology is regarded as a gold standard for quality of care measurements in healthcare settings. The method is growing in popularity in low-income and middle-income country (LMIC) settings, creating new opportunities to answer multiple research questions regarding quality of healthcare. The method can also be used in routine quality improvement programmes.
- We present a conceptual framework accompanied with examples from unpublished and published replication SP data to show how four types of research questions have been approached using different research designs and sampling techniques. These questions are difficult to answer with other methodologies typically used to assess quality of care without inducing considerable biases.
- Implementing the method is nuanced and must be done carefully. Therefore, we draw from our experience implementing SP work at both small and large scale to provide a toolkit and manual for implementing the SP method in LMIC settings.

## Introduction

The idea that governments should provide equitable access to high-quality health services or effective universal health coverage is rapidly gaining centre stage.^1 2^ Here, the quality of a health service is intrinsically tied to ‘effectiveness’, or the likelihood that when a patient visits a health facility, his condition will be correctly diagnosed and managed with a high level of patient safety. To ease communication, we use she/her/hers for providers and he/his/him for patients.

Measuring and defining effectiveness, unfortunately, remains difficult because patients can be misdiagnosed, undertreated or overtreated depending on their specific condition.^3–5^ For instance, a patient who received antibiotics is considered overtreated if he had viral pharyngitis, but not pneumonia. Worse, patients can be simultaneously misdiagnosed, undertreated and overtreated. A patient with a heart attack may be misdiagnosed as suffering from pneumonia; undertreated because he was not given an aspirin; and overtreated because he was given antibiotics instead (this particular combination was noted in Nairobi, Kenya, eg, Das *et al* 2). Low quality of care is harmful for individual patients, the broader population, and the health system if, for example, the proper management of contagious conditions is delayed, the overuse of antibiotics leads to antimicrobial resistance, and unnecessary procedures and medicines lead to increased costs of care. That we now have the tools to measure such deficits in care is largely due to the increasing use of standardised patients (SPs) as a method to measure healthcare quality.

## SP methodology: strengths and limitations

SPs are people recruited from the local community and extensively trained to present the same prespecified condition to various providers. For instance, an SP may be trained to portray angina, reporting to the doctor with ‘crushing chest pain’ when he woke up and accurately responding to questions and examinations that the doctor then performs. This method is fundamentally different from other proposed quality measures, both in the richness of the data and its ability to avoid typical biases or confounding issues arising from patient sorting and casemix.

Consider, for example, four traditional methods of quality measurements for healthcare: (1) interviewing patients after they receive services (exit interviews), (2) interviewing providers to assess their knowledge (provider interviews and vignettes), (3) analysing data from claims or medical records (record abstraction), and (4) observing patient–provider interactions (direct patient observation).^6–12^ We briefly discuss the strengths and limitations of these methods alongside the SP method below but refer the reader to a more thorough discussion in section 1 and table 1.1 in the online supplementary 1.

10.1136/bmjgh-2019-001669.supp1 Supplementary data

Relative to these methods, the use of SPs confers five distinct advantages. First, even when substantial information is collected through patient observations or exit surveys, at best the observers see what the doctor recommends. They cannot truly know what the patient is suffering from. We can, therefore, examine specific overall metrics of care (eg, consultation time or antibiotic use), but since we cannot assume whether more or less of each type would be ‘better’, we cannot ascertain the effectiveness, let alone appropriateness, of this care. In contrast, because researchers designed an SP case with angina symptoms, for example, they can thus correctly infer that a patient given antibiotics and sent home was incorrectly managed. Researchers can, therefore, assess the care received, including misdiagnosis, overtreatment and undertreatment, against prespecified benchmarks for the condition of interest.

Second, the use of SPs allows researchers to minimise measurement issues related to patient sorting and casemix, which confound observed relationships in administrative and patient data. This comes from the fact that the same SP can visit multiple providers with an identical presentation. Third, since providers are unaware when they are interacting with an SP, biases from the Hawthorne effect and social desirability whereby providers change their behaviour or survey responses because they know they are being observed or surveyed are avoided.^10^ Fourth, the ability to design many aspects of the condition presented—down to the way the SP dresses, carries himself and behaves—allows researchers to tailor the mix of conditions presented to a given context and a given research question. When combined with specific research designs and sampling techniques, the SP method can help answer important questions that have proven difficult or impossible to tackle with data from real patients. Fifth, the SP method is necessary to identify the gap between what providers know and what they do in practice (ie, the know-do gap).

These advantages of the SP method have vastly expanded the scale and scope of SP studies beyond the first population-based study in a large, representative sample of providers in India.^13^ SP protocols have now been developed for a range of medical conditions and implemented in multiple countries around the world and have proven to be a better fit for studies where researchers are interested in (1) understanding clinical practice (rather than knowledge, which are better measured through medical vignettes) and (2) quantifying the extent of overtreatment and undertreatment (currently the only method that produces reliable estimates) based on specific tracer conditions. For example, SPs have been used to answer questions related to quality differences between providers in the public and private sectors, whether providers treat men and women differently, and the impact of medical training on quality of care.^12 14 15^ Further, provider behaviour can be compared across different methods, adding further insights into the determinants of quality.

Nevertheless, the SP method has its limitations and challenges. For instance, the method is limited to a one-time interaction with a provider and has not yet been validated for multiple, sequential visits to the same provider as may be required for a chronic condition. The SP method is also not feasible for conditions that require physical signs to be evident (eg, trauma and pregnancy). As for assessing quality of care for childhood conditions, the SP method has been used with and without real children present, which requires different, yet detailed precautions.^16^ Further, the SPs are trained to present their symptoms and history in a manner that should not lead the provider in a wrong direction, but in real-life situations, many patients have difficulty presenting the symptoms and history of their disease in a clear manner, and the provider could be misled because patients give a very confused picture of their symptoms. Even further, the identity of SPs themselves may lead to different estimates–quality estimates from a more educated SP population may not be generalisable for less educated patients. Ethical considerations are also paramount, as both providers and SPs could be subject to harm in the conduct of the research if initial scoping of the setting and proper mitigation strategies are not put in place.

The richness of the data from SPs and the ability of the SP method to account for multiple biases in observational studies yields unique information that can save lives. With this in mind, research teams will want to assess the costs and trade-offs of implementing SPs relative to other data collection methods. We have found that developing the capacity to implement an SP study is the most costly investment (in terms of time, effort and finances) of the SP method. Once this capacity has been developed, with sufficiently large samples, the costs of an SP survey compared with collecting observational data with other methods are roughly equivalent or even lower. This is because, even though the initial SP set-up and training costs are high, the cost of each interaction is relatively low. (For a richer discussion on actual costs, we refer the reader to pp. 47–48 of the online supplementary file, where we discuss costs per interaction across studies.)

Recognising that the rapid expansion of quality measurement using SPs has brought with it increasing demands on the method and a new set of questions, this practice paper has two main goals. The main article presents a regression-based framework for quality of care research using the SP method. Using examples from published studies and ongoing research, we illustrate how SP studies and the data they generate can be designed to answer a variety of descriptive and causal research questions that go beyond basic quality measurement. We discuss issues that have arisen with SP measurement, including those that are yet to be satisfactorily resolved. We highlight that many of these problems are common to all quality measurement methods, but become especially salient because of the richness of the data that SPs provide. The main article thus seeks to answer: ‘Can an SP study contribute to my research question of interest, and if so, what issues should I be aware of?’

We complement the main article with a comprehensive online supplementary file that includes our SP Toolkit and Manual, which has been developed through successive iterations across multiple SP studies dating back to 2008. The online supplementary file includes detailed discussions of ethical issues, institutional review boards (IRB) concerns, costs, SP recruitment and training, and real examples of questionnaires and data structures. It accumulates the wisdom of teams as well as multiple IRB and ethics committees to help conduct SP studies in a valid and ethically robust manner from designing the study and obtaining ethical approval to field implementation. Specifically designed as an updated ‘How To’ guide for those planning an SP study in the field, it answers the question: ‘Having decided to do an SP study, how should I actually implement it?’

We make three further observations. First, these resources complement our recent practical overview of SP implementation in the field.^17^ Second, this article’s focus is on the econometrics and statistics of the SP method with most examples drawn from our own studies in low-income and middle-income countries (LMICs). This focus reflects our own expertise and familiarity when it comes to quality measurement in healthcare and therefore the areas where we can provide the most value. Third, SPs are new to healthcare quality measurement in LMICs, but are a well-developed tool in research on discrimination in housing and labour markets where they are referred to as ‘audit studies’. For those interested in the general issues with such approaches, we refer readers to the overview by Bertrand and Duflo.^18^

## Statistical framework

The starting point for our framework is the condition-specific quality of care delivered during a patient–provider interaction. Researchers may be interested in a single quality of care outcome associated with a question (eg, Was the SP’s condition correctly treated? Did the SP receive antibiotics unnecessarily?), a set of outcome variables (eg, the co-occurrence of correct treatment, unnecessary antibiotics, adherence to checklist items), or even a quality index that combines different outcome variables. We take the perspective that a quality outcome in a given interaction reflects a combination of inputs that vary at provider, patient and facility levels. In a multiple regression framework:

$$Quality_{ijkt}=\alpha +\beta *Provider_{it}+\gamma *Patient_{jt}+\delta *Facility_{kt}+\epsilon_{ijkt}$$

where $Quality_{ijkt}$ is some quality of care outcome of interest given by provider i to patient j at health facility k at time t; $Provider_{it}$ is a vector of provider characteristics at time t; $Patient_{jt}$ is a vector of patient (or client) characteristics at time t; $Facility_{kt}$ is a vector of facility characteristics at time t; and ϵ is an error term. Relevant provider characteristics include provider education, training, qualification, age, and sex; patient characteristics can include disease severity, socioeconomic status, being empowered, gender and other demographics; and facility characteristics can include facility size, level of care, public or private, management, as well as community characteristics, such as urban or rural.

This formulation directly links policy concerns to study design and implementation questions in the field. For example, if we are interested in a provider training intervention or incentive programme, β can capture provider-level improvements; if we are interested in the effects of patient empowerment (or other patient characteristics) on the outcome Quality, γ can capture the associated change between empowered and not empowered patients; and if we are interested in facility-level changes, δ would describe these, such as improvements in management or infrastructure, or declines in caseload. This model can be further adapted to account for longitudinal data with time-varying variables, such as assessing quality before and after an intervention or in quality improvement efforts.

The multiple regression framework also highlights the role of SPs in quality measurement. To begin with, we cannot define a condition-specific Quality metric without SPs or an assumption that the doctor correctly diagnosed the patient. The assumption of correct diagnosis has now been invalidated through multiple SP studies, where correct diagnosis rates are typically below 50%.^19^ Further, methods such as exit interviews and observations of real patients are subject to confounding (and therefore, biassed estimates of β, γ, and δ), because unobserved patient characteristics may be highly correlated with provider-level and facility-level characteristics. Very sick patients may choose to visit doctors with better training, which confounds the link between training and outcomes, or poor patients may only be able to access poor performing providers. Additionally, exit interview data is confounded further by sample selection issues and the willingness to be interviewed. Virtually, all hospital systems, therefore, include an ex-post risk adjustment for casemix, but (1) these adjustments themselves are subject to critique^20^ and (2) the data requirements for such adjustments are seldomly met in LMIC settings. The SP method addresses both of these problems, because the same condition is presented to every provider or facility in the sample–by design there is no correlation between unobserved patient characteristics and facility/provider characteristics.

Armed with rich data and without the risk of confounding due to casemix, SP studies can then elicit the average level of Quality for various populations, or can examine how observed differences in inputs predict variation in Quality (eg, some facilities have better infrastructure than others). Finally, the SP method can answer causal questions about differences in quality via methods drawing on ‘experiments of nature’ or through induced variation using experimental methods. One example is varying whether sampled providers receive an SP that demands antibiotics vs one that does not to assess the effect of patient demand on antibiotic use.

Using this statistical framework, we provide research examples drawing from our experience and specific data sources from SP studies listed in table 1. Each study used the SP method to evaluate quality or the performance of providers across different components or dimensions of care (eg, history questions asked, physical examinations conducted, diagnosis or referral made and treatment dispensing or prescribing). All data were analysed and visualised in R and Stata V.14 (StataCorp).

**Table 1:** Data sources by article and SP study

Authors (Date)	Short title	Setting	Sector	Health service(s) Aassessed	Level: sample size*	Data source descriptions
Daniels *et al* (2017)	Use of SPs to assess quality of healthcare in Nairobi, Kenya	Urban Kenya	Public and private	Asthma, childhood diarrhoea, tuberculosis (TB), unstable angina	Facility: 166	Nairobi SP data
		Multiple Countries	Public and private	Asthma, childhood diarrhoea, TB, unstable angina	Facility: 2255	SP data for international comparisons
Das *et al* (2015)^21^	Use of SPs to assess quality of TB care	Urban India	Private	TB (four cases)	Provider: 250	Validation study for four TB cases
		Urban India	Private	TB (one case)	Provider: 69	Know-do gap for textbook TB case
Das *et al* (2016 Science)^15^	The impact of training informal healthcare providers in India	Rural India	Private	Asthma, childhood diarrhoea, TB, unstable angina	Provider: 860	SP data
Das *et al* (2016 AER)^12^	Quality and Accountability in Healthcare Delivery	Rural India	Private	Asthma, childhood diarrhoea, TB, unstable angina	Provider: 1109	SP data
		Rural India	Private and Public	Asthma, childhood diarrhoea, TB, unstable angina	Provider: 455	Same providers at public and private locations
Kwan *et al* (2018)^22^ and Daniels *et al* (2019)^14^	Variations in the quality of TB care in urban India and use of SPs to assess gender differences in quality of TB	Urban India	Private	TB (four cases)	Facility and provider: 2602	Interactions for four cases weighted for representative levels of quality across two cities
		Urban India	Private	TB (one case and variant)	Provider: 101	Interactions for one case to assess effect of diagnostic report
Satyanarayana *et al* (2016)^23^	Use of SPs to assess antibiotic dispensing for TB by pharmacies in urban India	Urban India	Private	TB	Pharmacist: 1200	Interactions for two cases weighted for representative levels of quality across three cities
		Urban India	Private	TB (two cases)	Pharmacist: 2593	Medicines for interactions across three cities
Sylvia *et al* (2015)^29^	Survey using incognito SPs shows poor quality care in China’s rural clinics	Rural China	Public and private	Asthma, childhood diarrhoea, unstable angina	Lev: 82	SP data
		Rural China	Public and private	TB	Provider: 274	SP data
Sylvia *et al* (2017)^27^	TB detection and the challenges of integrated care in rural China	Rural China	Public and private	TB (one case)	Provider: 486	Know-do gap for textbook TB case
Unpublished (n.d.)	Qutub Project, 2014 to present	Urban India	Private	TB (four cases and variants)	Round 1: N=1636 interactions (n=999 with AYUSH, n=637 with allopathic facilities and providers); Round 2: N=2231 interactions	Quality of care surveillance conducted with stratified, random samples of providers

*Number of interactions available for analysis at facility and/or provider level in replication data.

SP, standardised patient.

## Research examples with the SP method

Our specific examples focus on how SP data can be used to study: (1) quality of care in the population, (2) variation across facilities, (3) variation across providers and (4) variation across patients. Throughout, we provide concrete examples and highlight specific problems that require further research.

### Quality of care in the population

We first discuss how the SP method can be used to obtain ‘average’ measures of quality in a population. We focus on examples provided by a series of studies where SPs presented tuberculosis (TB) cases, beginning with a pilot study that validated the use of the SP method for TB. Das *et al* confirmed that: (1) study participation placed minimal risks on SPs and consenting healthcare providers; (2) a very small percentage of SPs were detected and (3) SPs were able to recall accurately what occurred during interactions, as verified against audio recordings.^21^

Following the validation study, these SP cases for TB were then implemented at a larger scale with representative provider and pharmacist samples in multiple Indian cities.^22–26^ These studies have shown, for instance, that there is little difference in quality of care between one of India’s richest and one of its poorest cities. Subsequently, the SP TB cases have been translated and contextualised for use in China, South Africa and Kenya.^19 26–28^ The standardised field implementation, SP case design and data collection protocol continue to allow for international comparisons of provider behaviours related to TB care, informing policy and decision-making.

#### The problem of appropriate weights

Through this process, two issues have arisen related to the construction of weighted estimates. We discuss these in some detail as the concerns are clearer with SP data, but are pertinent for quality measurement.

The first is how to weight different components of a multidimensional quality measure. For instance, provider behaviour can be assessed against a set of necessary history questions and physical examinations (checklist adherence), against management decisions or against the use of unnecessary or harmful medicines. These measures can yield very different rankings of quality such that any aggregate index is quite sensitive to the weights of the components in the index.

The second is how to move from measures of quality for the provider population to measures for the patient population. Consider, for example, a village with two doctors, one with low quality=0, and one with high quality=100. The mean quality of *providers* in the sample is 50. But if caseloads vary, this is not an accurate estimate of the average quality that *patients* actually receive: if $s_{h}$ is the share of patients belonging to the high quality doctor, the average quality that patients receive is the share-weighted sum $(1-s_{h})*0+s_{h}*100$ = $100*s_{h}$.

The two measures, therefore, answer two different research questions. The mean quality of providers in the sample answers the question: ‘If a patient chooses a doctor at random from the population, what quality should she expect to receive?’ By contrast, the mean quality for patients answers the question: ‘Given the observed patient shares across providers, what is the average quality that patients receive?’ While the first question can be answered using data from the SP study alone, the second question requires additional information on patient shares for each doctor. When we are interested in a specific illness, these shares will also need to be illness-specific.

**Figure 1:**
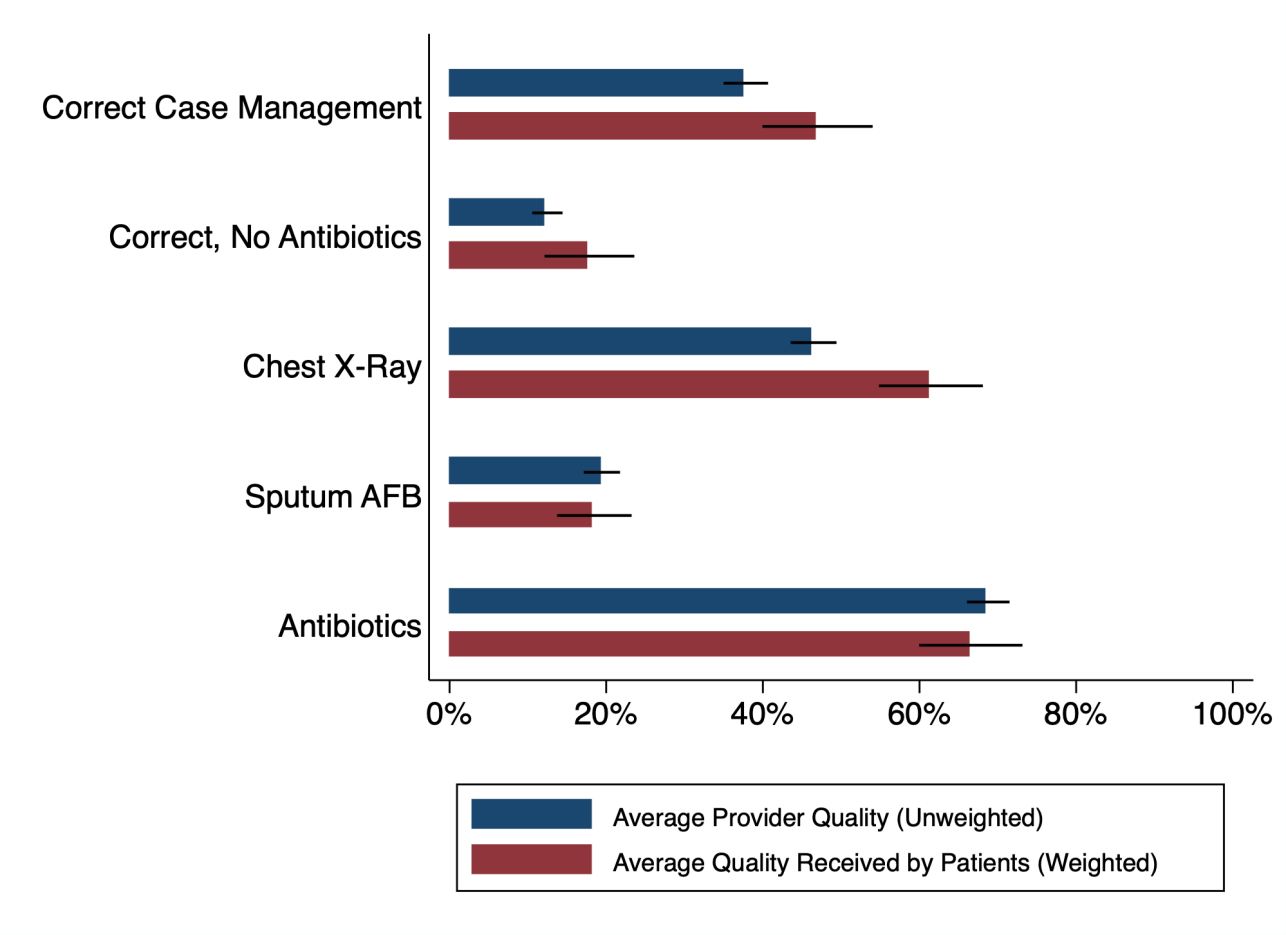
What is average provider quality versus what is the quality an average patient receives? Different dimensions of quality for SPs presenting with various forms of TB are compared with demonstrate differences in average provider quality (unweighted SP data) versus average quality received by patients (SP data weighted by number of patients in the waiting room). Visualisation demonstrates that quality for average patients is better than the average quality of providers. Source: data from Patna baseline, four TB cases, Qutub project, published in Kwan *et al*.^22^ SP, standardised patient; TB, tuberculosis.

These are not just academic concerns. Figure 1 uses data from Kwan *et al* 22 to construct two different measures of correct TB care management: one that penalises the use of unnecessary or harmful medicines (‘correct, no antibiotics’) and a second that does not (‘correct case management’).^22^ For each, we present unweighted measures (blue), as well as estimates weighted by patient shares based on the number of patients in the clinic at SP arrival (red). In addition, we present components of the correct management index—whether the provider asked for a chest X-ray or a sputum acid-fast bacilli (AFB) test (both appropriate management based on national standards), as well as whether the provider gave antibiotics (considered unnecessary). Our patient weights are necessarily crude, as these facilities seldom maintain patient records, let alone patient records for specific presenting conditions.

Many providers who recommend a chest X-ray or sputum test also give unnecessary antibiotics. Consequently, there is a significant decline in the proportion of SPs who were correctly managed when we penalise providers for the use of unnecessary medicines. Furthermore, estimates weighted by caseload increase correct case management proportions as well as the use of chest X-rays but do not appreciably decrease the use of antibiotics. In this sample, individuals are more likely to visit providers who correctly manage patients, so that the quality that patients receive is higher than the mean quality of providers in the sample. However, providers who correctly manage patients are, if anything, slightly more likely to also give unnecessary antibiotics—here, there is little difference between patient weighted and unweighted estimates.

Addressing the sensitivity of correct management to the weights of the components in the index is conceptually difficult, and there may be no single ‘correct’ answer to the question, ‘Who is a better doctor?’ Therefore, our recommendation—as we have done in our previous papers—is to clarify the components of the index and present each component separately. This allows researchers and policy-makers to assess which components of the index drive the result and to potentially reweight the index according to their specific concerns. Alternatively, researchers may opt not to use any index at all, and simply provide various empirically observed data points, particularly where cross-contextual comparisons may be an issue.^26^

Moving from the mean quality of providers in the sample to the mean quality that patients receive is conceptually simpler, but demanding in terms of the data. Most private facilities do not maintain patient records, and given the low levels of accurate diagnosis documented in SP studies, it is next to impossible to obtain illness-specific caseloads by provider in some settings. One strategy that Sylvia *et al* 29 successfully used in China combined household surveys with hypothetical questions (‘Who would you visit if you had a cough for 2–3 weeks?’).^29^ This is a complicated and expensive exercise that works only when the provider sample is small. In an urban area with 20 000 healthcare providers, any reasonable household sample will still yield zero visits to most providers, leading to erroneous results. Given the massive variation in patient load across health clinics in LMICs, weighted and unweighted estimates may be substantially different and this difference is fundamentally linked to the correlation between patient load and quality. Unfortunately, we currently do not have a good estimate of this correlation. We do urge researchers working with quality to collect as much data as possible on patient load, which may come from clinic records or just by counting the number of patients in the waiting room.

### Variation across and within facilities

Once weighting issues have been resolved, several research designs are possible. One design examines the link between facility characteristics and quality of care. For instance, we have shown previously that measures of structural quality (eg, whether the facility has a back-up generator, number and percentage of trained healthcare providers) are not associated with quality of care as measured by SPs.^13^

**Figure 2:**
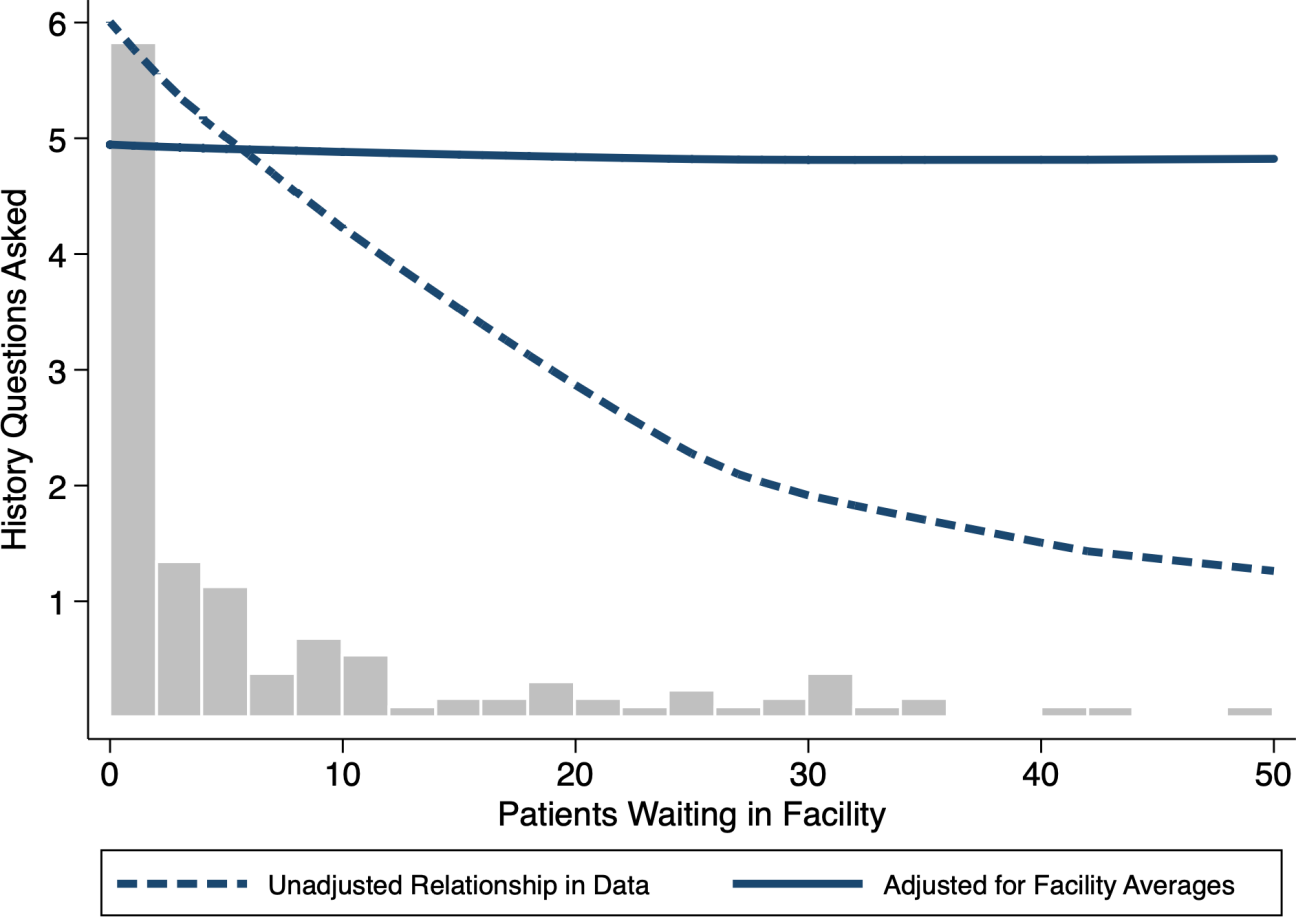
Do care dimensions vary across number of patients in the waiting room? Bars reflect the number of facilities where SPs report the number of patients waiting on arrival. An unadjusted local polynomial fit of raw data suggests that the number of history questions asked decreases sharply with the number of patients waiting in the facility (dashed line). When adjusting the data with a facility fixed effects model, the relationship disappears between caseload variation within facilities and the number of history questions that SPs were asked (solid line). Source: Daniels *et al*.^19^ SPs, standardised patients.

As another example of how facility characteristics can alter quality of care, we consider the question of whether quality of care varies with caseload. Maestad *et al* have shown that in Tanzania, measures of effort do not vary with caseload using the size of the catchment area as an instrument for caseload in an instrumental variables specification.^30^ Figure 2 shows another example from Nairobi, Kenya using data from Daniels *et al*.^19^ Here, the histogram shows the relative frequency of facilities for the numbers of patients in the waiting room on SP arrival. An unadjusted local polynomial fit clearly shows that the number of history questions asked decreases sharply with the number of patients waiting in the facility (dashed line). However, this observational relationship may be driven by the fact that low-quality facilities are systematically located in slums where utilisation is higher. If so, it does not follow automatically that increasing the caseload at a given facility would decrease the effort or quality within that facility.

To answer this question, we exploit the fact that 3–4 visits were conducted at each of the 42 facilities in the data and, critically, these visits were spread across multiple days with varying waiting times (Mondays are the busiest as facilities are closed on Sundays). This allows us to further examine whether variation in the caseload *within* each facility was correlated with the number of history questions asked. This ‘fixed-effects model’, borrowed from the economics and statistics literature, fully accounts for facility characteristics that do not vary over time by focusing only on the variation that arises within each facility. In other words, we can statistically examine whether the same SP received worse care on Monday when the facility was busier compared with Thursday, when there were fewer patients. Strikingly, there is now no relationship between caseload variation within facilities and the number of history questions that SPs were asked (solid line, figure 2). Whether the SP visited on Monday and waited 6 hours in a crowded facility or on Thursday when wait times were much lower had zero impact on the care they received, at least measured by the extent of history taking.

**Figure 3:**
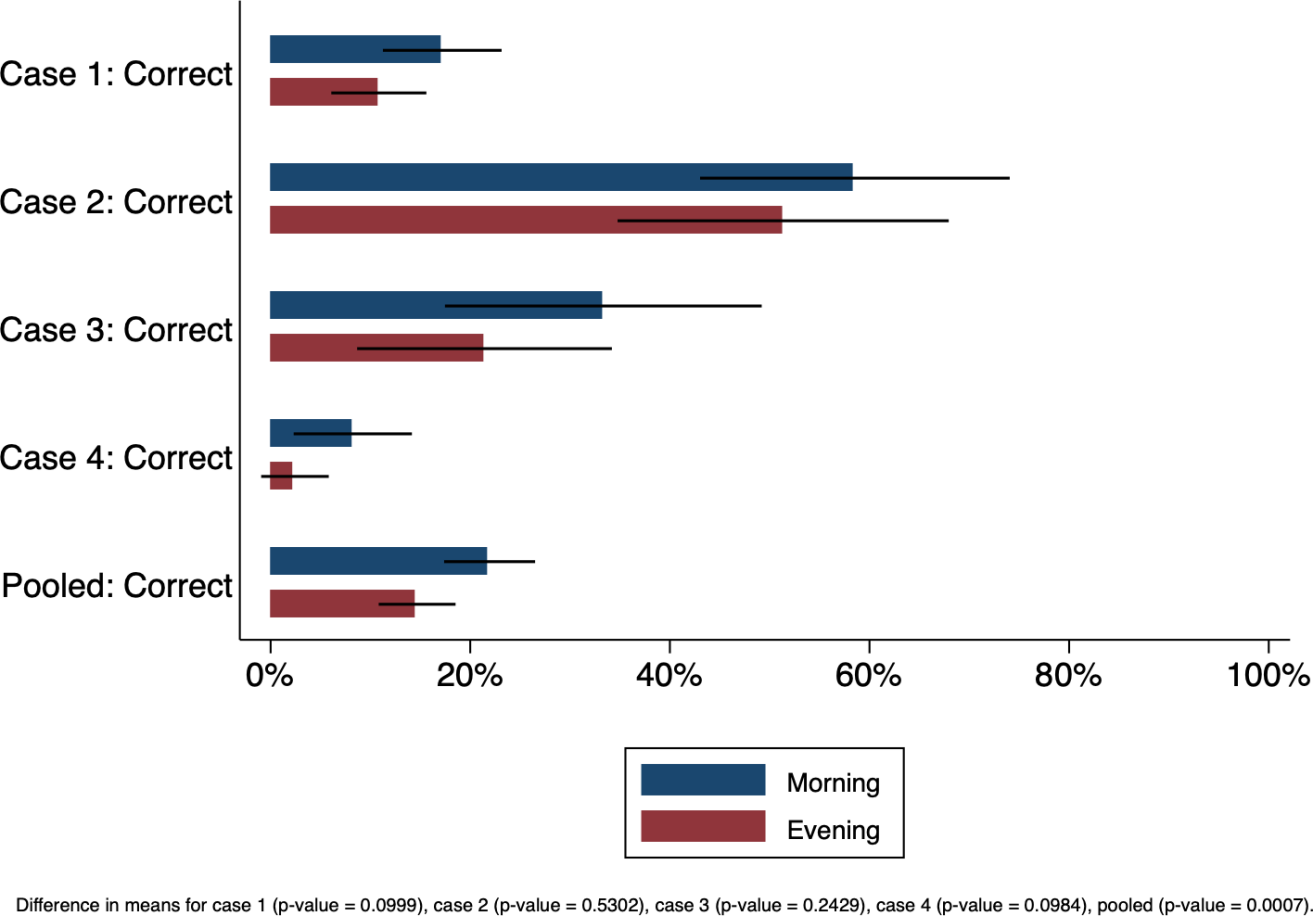
Does quality vary across operating hours? Percentage of providers correctly managing the patient in the morning or evening across SP cases. Case 1—a classic case of presumed tuberculosis with 2–3 weeks of cough and fever; Case 2—a classic case of presumed tuberculosis in a patient who has had 2–3 weeks cough and fever and who has also taken broad-spectrum antibiotic and carries an abnormal chest X-ray; Case 3—a tuberculosis case who carries a positive sputum smear report for tuberculosis and Case 4—a multidrug-resistant tuberculosis suspect with previous, incomplete treatment for tuberculosis. Source: Data from Kwan *et al*.^22^ SP, standardised patient.

Designs that exploit within-facility variation can be tightened further by controlling and randomly assigning when SPs visit facilities across conditions and SPs. In Mumbai, India, where private clinics have distinct ‘morning’ and ‘evening’ operating hours, we incorporated random assignment in the timing of SP visits. During fieldwork, we randomly assigned SPs to visit clinics either in the morning or evening. Figure 3 depicts the percentage of providers correctly managing the case in the morning versus evening across four TB cases. Across all cases, performance falls for the same SP if the visit is in the evening, with statistical significance when we pool all cases together (p=0.0007).

Using naturally occurring variation—in these examples, by facility caseload or time of day—is one example of how SPs can be used to study associations between quality of care and facility characteristics. Similar questions can be explored within randomised controlled trials with facilities assigned to ‘treatment’ receiving a separate package of interventions relative to those in ‘control’. One difficulty that researchers should be aware of is that randomising SPs to facilities is logistically harder and can significantly increase costs. A typical SP study usually takes advantage of the geography of the sample so that SPs visit providers who are located close to each other on the same day. Randomisation implies that this geographical advantage is lost so that the number of potential visits may decline sharply, increasing the duration of the study.

### Variation across providers

Our third example looks at the relationship between provider characteristics and quality of care, focusing on associations, randomised controlled trials and natural experiments. We also discuss how combining SP and other measures of quality can lead to a better understanding of performance deficits.

#### Associations

Qualifications are one key determinant of quality of care, and several studies show that providers with higher qualifications are more likely to correctly manage SPs, although they are no less likely to give inappropriate and unnecessary medicines. These are again associations—if more altruistic providers are more likely to enter medical training, then the association between qualifications and quality of care reflects both the differences in training and in altruism. However, assessing the actual patterns of variation in care quality across a population is valuable in its own right.^13 22 27^

#### Randomised controlled trials

To evaluate causal impacts, one option is to combine SP measurement with a randomised controlled design. Das *et al*, for instance, evaluated a multitopic training programme among informal providers in India.^15^ The impact of different training was identified through random assignment of training across a group of providers, and further, the trainers were not told what conditions the providers will be evaluated on. This triple-blind approach, where the training institution is blinded to the conditions that will be tested, SPs are blinded from programme assignment, and providers do not know that they are treating SPs, opens up new possibilities for evaluating general multitopic training, as ‘teaching-to-the-test’ is effectively eliminated. In that particular study, the authors showed the training increased correct management, but had no impact on the use of unnecessary medicines and antibiotics. A similar strategy was used to assess whether a social franchising and telemedicine intervention improved the quality of childhood illness management in Bihar, India. The study found zero improvements in the clinical management of childhood illnesses using SPs.^31^

#### Natural experiments

An alternate research design examines how quality of care varies by the location of the provider. In the USA, for instance, there are systematic differences in quality by geography and a recent research design compares the performance of the same provider across different locations.^32^ Similar to this approach, an SP study conducted in India demonstrated variation in quality for the same provider depending on whether they are practising at their private or public clinic.^12^ They showed that the same doctor was more likely to correctly manage the same SP in their private rather than public sector location. In terms of adherence to checklists, the impacts were so large that they catapulted the same doctors from among the worst in the sample when practising in the public sector to among the best when practising in the private sector. We highlight that researchers could have compared the same doctor in multiple locations without SPs, but this would have always left open the question of whether practices differed due to differences in patients and casemix. This application of the SP method allowed researchers to demonstrate that individual provider behaviour is in fact malleable and that quality difference between the two sectors is driven by differences in provider effort levels.^33^ Future research should examine the reasons for these observed differences through mechanisms like resource availability, incentive structures, and provider and patient beliefs and preferences.

#### Multiple quality measurements

Combining the SP method with other survey methods also allows researchers to understand the determinants of quality of care. For instance, one leading candidate for poor performance is that providers do not have the requisite knowledge or training to treat these cases. To assess this possibility, researchers have sequenced SP visits to be followed by clinical vignettes for similar patient scenarios.^21 27 34^ That is, after an SP visits the doctor, a survey team assesses knowledge using clinical vignettes. One member of the team acts as a patient, presenting exactly the same condition as the SP previously, and all the responses of the provider are then recorded and compared with their performance with SPs.

**Figure 4:**
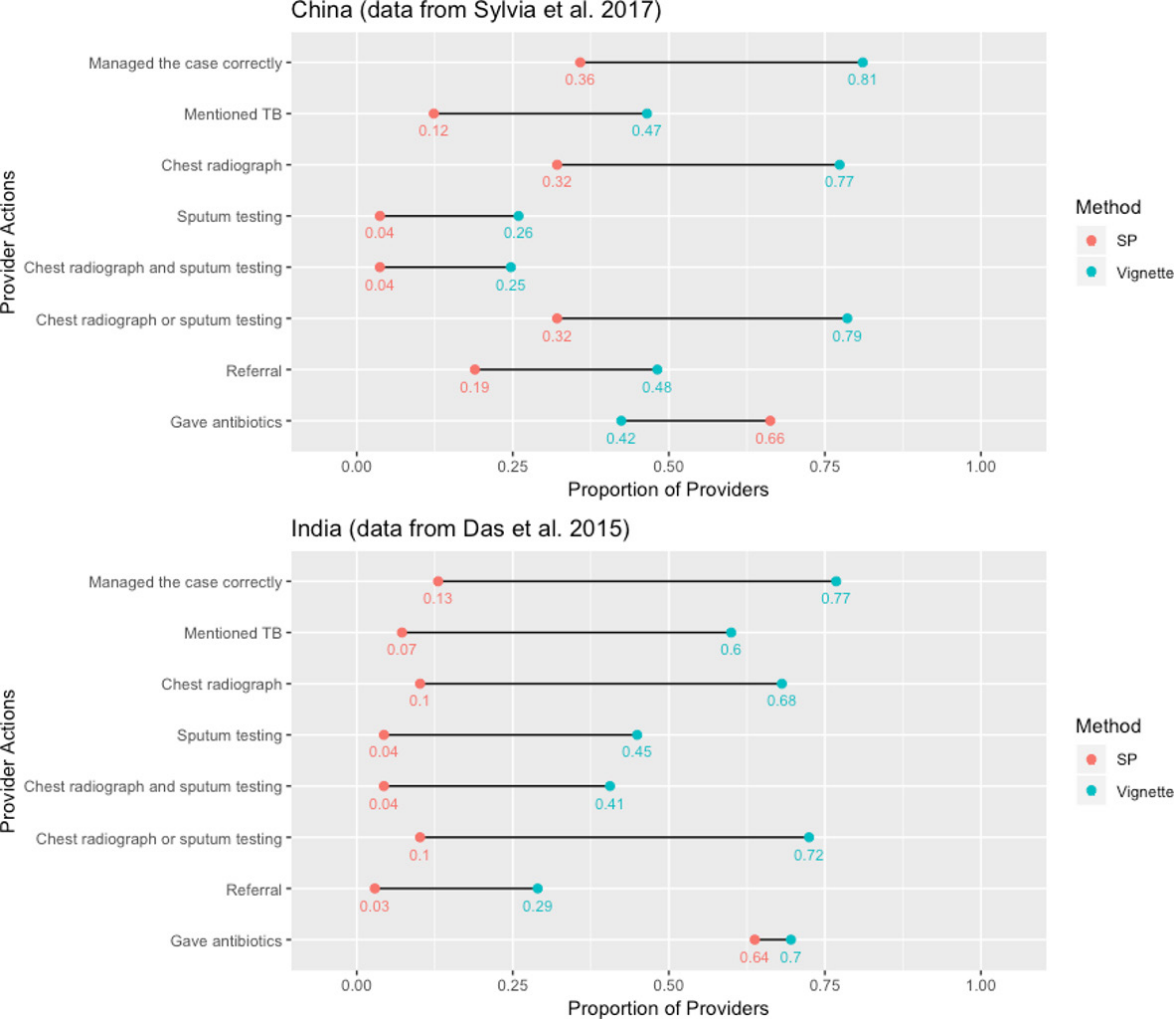
To what extent does provider knowledge differ from actual practice? Gaps (black bars) between knowledge (blue) and performance (red) measures for TB suspect with 2 weeks cough and fever. sources: Das *et al* 21, Sylvia *et a.l* 27 SP, standardised patient; TB, tuberculosis.

This approach has uncovered an empirical regularity in most studies, that has come to be known as the ‘know-do gap’ after Das *et al*.^35^ Figure 4 shows this ‘know-do gap’ among providers in China and India based on data from Sylvia *et al* 27 and Das *et al* 21 respectively.^21 27^ The case presented in both studies is an individual with 2–3 weeks cough and fever who should be suspected for TB. Correct case management is defined as ordering a chest X-ray or a sputum test or referring the patient. There are clearly dramatic gaps between knowledge and practice for the same set of doctors so that poor performance cannot be attributed to a lack of knowledge for these cases. Similar results have been documented for childhood illnesses in India.^34^ Note that the know-do gap reverses when it comes to the use of unnecessary medicines (although the gap is still in the direction of lower quality in practice than in knowledge)—in China, provider recommendations for antibiotics are greater for SPs compared with clinical vignettes. Complementing Currie *et al*’s findings that financial incentives are the main driver of antibiotic abuse in China, it is likely that providers give unnecessary medicines when doing so increases their profits.^36^ This ‘reversed’ know-do gap shows that both poor knowledge and incentives play a key role in the use of unnecessary medicines.

Combining SP measurements with randomised controlled trials, natural experiments such as dual practices, or multiple measurement methods have uncovered novel results in the literature. Future work might expand on such insights by combining SP data innovatively with data from large routine surveys like the Demographic and Health Surveys or World Bank Service Delivery Indicator Surveys, data available from previously published studies, data from drug sales records or insurance records, or other new sources of information. These studies will continue to uncover important regularities and new questions: Are lower performing providers practising where the poor or more disadvantaged reside? Why do we see doctors practice far below their knowledge frontier? Does this reflect a lack of incentives in the market, or is there a fundamental step in translating knowledge into practice that we do not fully understand?

### Variation across patients

The final category of research questions helps us understand the sensitivity of provider decisions to patient characteristics. We discuss three types of studies within this domain. The first asks whether providers treat different groups of patients differently. For instance, are men treated differently from women? What about patients from different socioeconomic levels or patients from racial, ethnic or religious minorities compared with the population? The second varies the presentation of the SP to better understand doctors’ behaviour. For instance, the question of whether doctors overuse antibiotics due to financial incentives can be addressed by varying the SP presentation to remove these incentives from the interaction. The third type again alters SP presentations, but here the goal is to better understand whether certain types of patient education programmes can enable complementary responses from providers. We illustrate examples of each type of study.

#### Type 1: using variation across SP presentation to detect discrimination

Men are much more likely to be notified for TB compared with women in India. To assess whether this is because men are more likely to be diagnosed accurately, Daniels *et al* sent 22 male and female SPs to providers in two cities in India with the same case presentations.^14^ Surprisingly, they found that although providers asked different questions of men and women (men are more likely to be asked about smoking and alcohol history), there were no differences across a broad range of outcome indicators including correct management, adherence to checklists or the use of unnecessary medicines. Planas *et al* 37 demonstrated a similar equivalence for family planning across different ethnic groups in Peru, suggesting that equal treatment by patient characteristics may hold across a range of attributes.^37^ One key advantage of these studies is that doctors were blinded from knowing who was an SP, thereby eliminating social desirability biases inherent in surveys and direct questioning.^38^

Although this design is conceptually simple, it raises two tricky issues. One (partially resolved) question is how to recruit SPs. For instance, if women and men are treated the same when they are educated or middle income, but not when they are illiterate or poor, there may be no discrimination in a study where the SPs are all educated and middle income. Daniels *et al* used data from a recently completed household survey to argue that the characteristics of the SPs matched those of patients presenting to private providers in urban populations, but this equivalence may not hold without deliberate recruitment when hiring SPs for studies, as well as subsequent training to ensure the SP case is portrayed in a manner true to the designed characteristics.^14^

A second question is whether the study is adequately powered to detect differences between men and women, given that there may be variation within each category as well. This highly technical, but critical issue has not been addressed in the literature on audit studies in labour economics. Daniels *et al* approach the problem through simulations, showing that the variation across the SPs was small in their sample and could be dealt with through standard statistical tools.^14^ We are not aware of existing analytical corrections and urge researchers using audit studies for assessing discrimination to account for this additional source of error in their computations.

#### Type 2: inducing variation in SP presentation to understand doctor behaviour

A second design varies SP presentations to better explain the behaviour of doctors. Currie *et al* 36 document that cuts in health budgets led hospitals in Beijing, China to compensate physicians through a profit sharing arrangement whereby physicians received a portion of the sales they generated at hospital pharmacies.^36^ To investigate whether these financial incentives could be linked to the overuse of antibiotics, Currie *et al* 39 send SPs who either indicate that they will purchase their medicines through an outside pharmacist or through the hospital.^39^ All SPs present with viral pharyngitis, which does not require the use of antibiotics. They show that the proportion of SP interactions resulting in the prescription of an antibiotic falls from 85% to 14% when financial incentives are removed. By contrasting their results with other SP presentations (also part of their study), they argue that financial incentives are a far more important predictor of antibiotic use compared with patient demand.

In contrast, financial incentives appear to have less of an effect on the likelihood of correct management. In Kwan *et al*, half of the SPs trained to depict multidrug-resistant TB were also asked to carry a positive sputum microscopy report from a government hospital.^22^ Through the interaction, the SPs made it clear to the provider that they had a test result but did not know what it implied. This study was designed to test a tight null hypothesis: if the only reason that doctors in the private sector do not correctly manage patients is to increase their income through repeat visits, the provision of additional diagnostic information that the SPs themselves cannot interpret should have no impact on their behaviour. In contrast, Kwan *et al* show that doctors are more likely to correctly manage their patients when the quality of diagnostic information improves, even though this can lead to financial losses in some cases.^22^

#### Type 3: inducing variation in SP presentation to understand the link between patient and provider behaviour

Types 1 and 2 studies can feed into policies aimed specifically at the behaviour of doctors. For instance, finding discrimination can lead to changes in medical education. In China, the findings of overprovision arising from financial incentives ultimately led to the removal of the financial link between diagnosis and treatment.^36^ In contrast, type 3 studies are designed to change patient behaviour to improve the quality of their interactions.

As part of a TB programme in Mumbai, India, providers were expected to offer vouchers for free lab testing (eg, chest X-rays for individuals with TB symptoms). Anecdotal evidence suggested that providers were not always handing out vouchers when the patients were eligible, and early SP visits confirmed this to be true. To determine if patient self-advocacy could influence voucher receipt, we randomly assigned SPs into two categories. One group would complete the interaction as usual (we refer to this group as ‘regular SPs’). But in the second group, if the SPs had been offered a lab test but not a voucher, they were asked to say at the end of their interaction, ‘But doctor, I heard these tests were free.’ For brevity, we refer to this group as ‘empowered SPs’. These were conducted across the same four SP TB cases described earlier.

**Figure 5:**
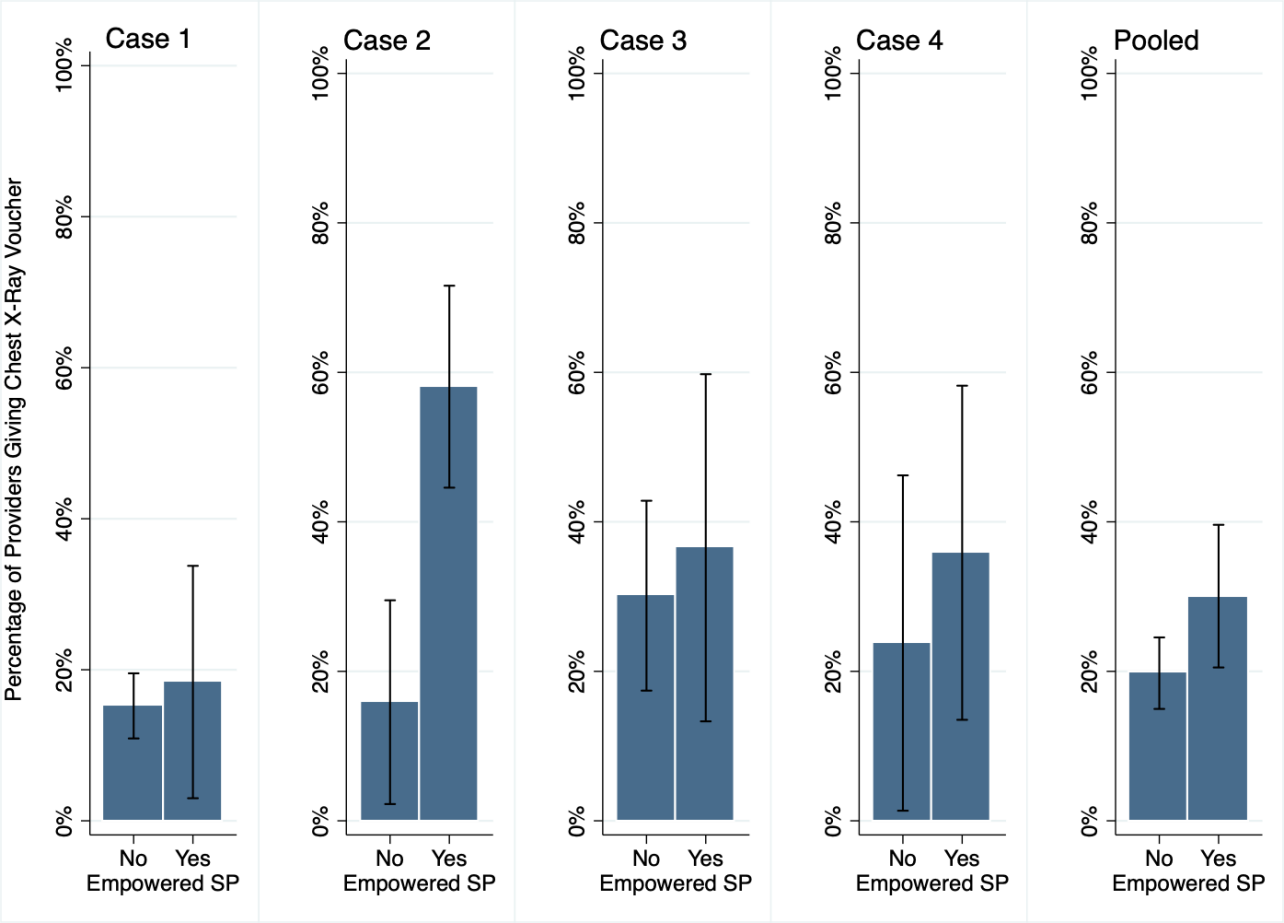
Can patient empowerment influence quality of care or other outcomes? Across case and for all cases pooled, results for the main outcome of receiving a voucher conditioned on a chest X-ray being ordered are shown comparing regular versus empowered SPs, controlling for sample type, SP case and provider qualification; Standard errors are clustered at SP individual level. Source: unpublished data, Qutub project. SP, standardised patient.

Our experimental design ensured that there were no statistical differences (data not shown) between empowered and regular SPs along the outcomes that were determined prior to the request for free tests. We find that regular and empowered SPs were just as likely to be correctly managed, be referred, receive tests for diagnosing TB (chest X-ray, sputum AFB microscopy and GeneXpert), or receive medicines. Encouragingly, empowered SPs were more likely to receive a voucher for chest X-rays with differential voucher dispensing across the cases, as shown in figure 5. The fraction receiving a voucher increased from 20.3% to 28.6% across all cases, with a particularly marked increase for case 2.

However, as we learnt from studies like this, if a small number of individual SPs present the variant of the standard case, statistical significance is difficult to obtain. Indeed, when we apply the variance correction suggested by Daniels *et al*, the effects become statistically insignificant, because we cannot distinguish the effect of the variant presentation from characteristic differences across individual SP. Daniels *et al* provide a discussion on this.^14^ As research teams have continued to ask more specific and challenging questions using SPs, this experiment acts as a cautionary tale that standard errors and power calculations are yet to be clearly determined.

## Conclusion

Innovative applications of the SP method have allowed researchers to document and understand the multiple dimensions of patient–provider interactions around the world. We can now provide clear, evidence-based statements of what happens when a patient with a particular condition visits a provider. Additionally, we can now begin to ask focused questions about the causes of specific outcomes and our ability to improve quality. These studies have uncovered significant deficits in care, but also tremendous variations across types of providers and across settings.

Three broad themes recur throughout our discussion of the SP methods. The first is that the SP method becomes more powerful when used in combination with other methods of measurement and novel research designs. Exploratory research that uncovers context-specific details of the health system (dual practice in India, payment practices in China), such as ethnographic and qualitative research, when blended with SP measurement can yield important insights into key questions.^40 41^ Moving forward, SPs can offer new data for understanding payment systems and insurance programmes, since SPs are uniquely situated to capture data on patient expenditures and quality outcomes. They also can identify systematic issues linked to patient safety and can be linked to medical records and administrative data.

A second theme that emerges is that SPs alone cannot solve the difficult issues that arise, for instance, from the multidimensional nature of quality. As the SP method is used to answer a broader range of questions, further questions will arise, and new tools will need to be developed. Detecting discrimination required us to better understand the role of SP recruitment, and estimating quality required us to better understand the difference between provider-weighted and patient-weighted estimates. We view this process of discovery—and in some cases, resolution—very much as a community effort that drives the science further. In doing so, multidisciplinary collaborations will prove critical.

The third is that the SP method will have inherent limitations, and there are certain questions that will remain outside the ambit of SP measurement, at least in the foreseeable future. First, the SP method has only been validated for one-time interactions between patients and providers, and thus the SP method is not ideal for health services that require sequential visits, such as for health conditions requiring continuity of care or hospitalisation. Although the SP method is not ideal for these health services, whether an alternative quality of care measurement method is more appropriate (eg, interviewing providers with vignettes or analysing administrative data over time) depends on the setting, research questions and resources available for research. Second, ethical considerations limit the use of SPs to health conditions that do not put individuals at risk of extensive procedures or hospitalisation. Finally, compared with other common methods of obtaining quality of care data, the SP method is harder to implement, requires deep contextual knowledge of the research settings and has high training costs. Multiple precautions must be taken in order to implement SP research ethically and in a standardised manner and for SP data to hold scientifically valid and unbiased interpretations.

To date, the SP method has primarily been used to measure levels of quality and to document variations and correlations with quality outcomes across provider and patient populations. The statistical framework outlined in this paper provides a broad, high-level guide for study design. In order to devise causal explanations and mechanisms for observed quality differences, future studies could consider using the SP method in combination with randomised trials that induce variation in facility or provider characteristics. These characteristics could range from management structures or resource constraints at health facilities, to population health or patient/provider selectivity and to healthcare provider beliefs, preferences, training and motivation. The SP method is a flexible and useful tool in the field for both developing new research questions through exploratory analysis and for implementing targeted experiments in an agile study design.
