# Supplementary material for: Use of standardised patients for healthcare quality research in low- and middle-income countries
Source: BMJ Glob Health. 2019 Sep 12;4(5):e001669. doi: 10.1136/bmjgh-2019-001669 (PMC6747906; doi:10.1136/bmjgh-2019-001669)
Supplement: Supplementary data [file bmjgh-2019-001669supp001.pdf]

Using Standardized Patients to Measure Health Care Quality

A Manual and Toolkit for Projects in Low- and Middle-Income Countries

Release v1.3
Last updated on June 21, 2019

Updated versions of the manual and annexes can be accessed at:
https://www.qutubproject.org/

Ada Kwan[1], Sofi Bergkvist[2], and Benjamin Daniels[3]
with
Jishnu Das[4], Veena Das[5], and Madhukar Pai[6]

[1]School of Public Health, University of California at Berkeley, Berkeley, USA; Development Research Group, The World Bank, Washington DC, USA. Email: adak@berkeley.edu.

[2] ACCESS Health International, New York City, New York, USA. Email: sofi@accessh.org.

[3] Development Research Group, The World Bank, Washington DC, USA. Email: bdaniels@worldbank.org.

[4]Center for Policy Research, New Delhi, India; Development Research Group, The World Bank, Washington DC, USA, Email: jdas1@worldbank.org.

[5]Department of Anthropology, Johns Hopkins University, Baltimore, USA. Email: veenadas@jhu.edu.

[6]McGill International TB Centre, McGill University, Montreal, Canada; Manipal McGill Centre for Infectious Diseases, Manipal Academy of Higher Education, Manipal, India. Email: madhukar.pai@mcgill.ca.

To use this material as a whole, or in part, please cite this manual as:

Kwan A, Bergkvist S, Daniels B, Das J, Das V, and Pai M. *Using Standardized Patients to Measure Health Care Quality: A Manual and Toolkit for Projects in Low- and Middle-Income Countries.* 2019.

This material is the Online Supplement to the following manuscript:

Kwan A, Daniels B, Bergkvist S, Das V, Pai M, Das J. The use of standardized patients for health care quality research in low- and middle-income countries. Submitted to *BMJ Global Health, 2019.*

All content in this document unless otherwise noted is licensed under the Creative Commons Attribution-NonCommercial 4.0 International License. To view a copy of this license, visit http://creativecommons.org/licenses/by-nc/4.0/ or send a letter to Creative Commons, PO Box 1866, Mountain View, CA 94042, USA.

Abstract

Measuring the quality of healthcare is a vexing problem: Quality is multidimensional and deficits in care can manifest as doing "too much" or "too little". One way to address several outstanding problems in the measurement of quality is through the use of standardized patients – people recruited from local communities and extensively trained to depict the same conditions to multiple providers. The standardized patient methodology offers a unique strategy to assess health care quality. Since providers see the "same" patient, confounders arising from differential patient and case-mix are better controlled for than when understanding health care quality through other methods, such as administrative data or medical records. Further, researchers know what illness the patient has; therefore, the performance of healthcare providers can be directly compared to national and international standards of care for that condition. The authors have developed expertise in the use of the standardized patient method in small-sample, large-sample, and population-based studies, particularly in India, China, Kenya, and South Africa. Based on that experience, this manual provides an overview of the use of standardized patients, extensive training material and methods, as well as detailed questionnaires, IRB applications, and a suite of material that can be used in future such studies.

Acknowledgments

This manual and its contents are the products of numerous individuals who have worked together across time and space to think through, iterate, and improve upon the fine details required for implementing the standardized patient method in low-resource settings.

For their unwavering effort, openness in sharing their experiences, and answering countless questions, many thanks go to Ranendra K. Das, Rajan Singh, Purshottam, Chinar Singh, Geeta, Devender, Varun Kumar, Anand Kumar, Babloo, Charu Nanda, and the standardized patients at the Institute for Socio-Economic Research on Development and Democracy (ISERDD); Amy Dolinger at the International Finance Corporation (KePSIE Project); and Sean Sylvia, Alexis Medina, and Scott Rozelle at Stanford University (REAP). We are grateful to members of the Technical Advisory Groups of the Qutub Project in urban India, the KePSIE Project in Kenya, and the ZASP study in South Africa, including Puneet Dewan, Peter Small, Shibu Vijayan, Nita Jha, Sirisha Papineni, Ramnath Subbaraman, and Srinath Satyanarayana, for their roles in the design and implementation of the SP method. We are thankful to Vaibhav Saria for his review and contributions, which have made the content throughout the manual richer; Sabnam Mahmuda and Angela Salomon for their editorial support; Brian Chan, Manoj Mohanan, Diana Tabak, and Alaka Holla of the MAQARI Project from which some of this material is based.

We acknowledge funding from Grand Challenges Canada, the Bill and Melinda Gates Foundation (OPP1091843), and the Knowledge for Change Program at the World Bank. The findings, interpretations, and conclusions expressed here are those of the authors and do not necessarily represent the views of the World Bank, its Executive Directors, or the governments they represent.

Making this manual error-free and evolving the standardized patient method for low-resource settings are left as collaborative exercises between the authors and readers.

Table of Contents

ACRONYMS & ABBREVIATIONS 8
STUDIES MENTIONED IN MANUAL 8
SECTION 1. INTRODUCTION 9
SECTION 2. OVERVIEW OF LITERATURE 19
SECTION 3. FEASIBILITY ASSESSMENT 47
3.1 Budget estimates and considerations 47
3.2 Review relevant regulation 48
3.3 Conditions or ailments appropriate for SPs 49
3.4 Information about patients, providers, and the healthcare market 52
3.5 Media and political considerations 54
SECTION 4. DESIGNING AN SP PROJECT 56
4.1 Project Proposal 56
4.2 Planning 56
4.3 Frequently asked questions 57
SECTION 5. CONDUCTING AN SP PROJECT 60
5.1 Selection of conditions, study considerations, and scientific rigor 60
5.2 Approval of Institutional Review Board or Independent Ethics Committee 60
5.3 Registering an SP study with a rigorous experimental design 65
5.4 Selecting a Technical Advisory Group 66
5.5 Government approvals and buy-in 68
5.6 Using audio recorders for SP recall verification 69
5.7 Staffing requirements 73
5.8 SP recruitment 75
5.9 Agreement with SP 83
5.10 Provider Mapping and Recruitment 83
5.11 Pharmacy Mapping and Recruitment 85
SECTION 6. SP CASE DEVELOPMENT 86
6.1 Selecting the clinical presentation of cases 86
6.2 Conceiving the SP cases: script and exit interview development 88
6.3 Developing medical artifacts for SPs to carry during the interaction 92
6.4 Allocating the cases to SPs 93
6.5 Designing the SP exit questionnaire 94
6.6 Designing vignettes 97
6.7 Designing a detection survey 98
SECTION 7. SP TRAINING 101
7.1 Schedule for training 101
7.2 Logistics for training 107
7.3 Training Activities 107
SECTION 8. OTHER TRAINING CONSIDERATIONS & PRE-PILOT PREPARATION 122
8.1 Assessing study environment for SP work and learning from other projects 122

8.2 Revisions in the script ... 126
8.3 Change in case allocation ... 126
8.4 Removal of non-suitable SPs ... 127
8.5 Add-on tools to test during training and dry runs ... 127
SECTION 9. PILOT ... 128
9.1 Daily fieldwork plan for pilot ... 128
9.2 Schedule for the SP visits ... 131
9.3 Potential schedule issues ... 131
9.4 Data collection checklist ... 131
SECTION 10. FIELDWORK ... 135
10.1 Sampling and scheduling ... 135
10.2 Logistical plan for fieldwork ... 136
10.3 Monitoring fieldwork ... 139
10.4 Fieldwork outputs ... 140
SECTION 11. DATA ENTRY, PROGRAMMING & ANALYSIS ... 141
11.1 Data files ... 141
11.2 Checklist and treatment grading ... 144
11.3 Electronic data capture approach ... 147
11.4 System for verification and quality checks ... 151
11.5 Data management and analysis ... 152
11.6 Incorporating secondary data sources ... 153
11.7 Data interoperability across multiple partners ... 154
SECTION 12. DISSEMINATION OF RESULTS ... 156
SECTION 13. CONCLUSION ... 157
GLOSSARY ... 158
REFERENCES ... 159
TABLE 2.1 REFERENCES ... 160

ANNEXES ... A1–A87
Annex A. Sample budget & justification templates (Section 3.1)
Annex B. Description of SP method for IRB submission (Section 5.2)
Annex C. Template for provider consent form (Section 5.2)
Annex D. Template letter of full disclosure at study completion in lieu of consent (Section 5.2)
Annex E. Ethical considerations for SP study (Section 5.2)
Annex F. Study authorization letter template from National Government (Section 5.5)
Annex G. Study authorization letter template from Local Government (Section 5.5)
Annex H. Health screening questionnaire for potential SPs (Section 5.8)
Annex I. SP confidentiality agreement template (Sections 5.9, 8.5)
Annex J. SP script – sample from Qutub Project (Section 6.2)
Annex K. SP exit questionnaire – sample from Qutub Project (Sections 6.2, 6.5)
Annex L. Follow-up detection survey and vignette – sample from Qutub Project (Sections 6.6, 6.7)
Annex M. 3-week SP training schedule
Annex N. Supervisor fieldwork schedule – Example (Section 9.2)

Annex O. SP fieldwork schedule – Example (Section 9.2)
Annex P. SP comments – Edited (Section 10.4)
Annex Q. SP data files (Section 11)
Annex Q1. SP data files – Provider universe master code example (Section 11.1)
Annex Q2. SP data files – Sample master code file example (Section 11.1)
Annex Q3. SP data files – Schedule and tracking master code file example (Section 11.1)
Annex Q4. SP data files – SP staff master code file example (Section 11.1)
Annex Q5. SP data files – Medicines master code file example (Section 11.1)
Annex Q6. SP data files – Exit questionnaire master data dictionary file example (Section 11.3)

ACRONYMS & ABBREVIATIONS

IEC – Independent ethics committee
IRB – Institutional review board
ISERDD – Institute for Socio-Economic Research on Development and Democracy
KePSIE – Kenya patient safety impact evaluation
M&E – Monitoring and evaluation
MAQARI – Medical advice, quality, and availability in rural India
QoC – Quality of care
Qutub – Quality of tuberculosis care
RCT – Randomized controlled trials
SP – Standardized patient
SPDES – Standardized Patient Data Entry System
TAG – Technical Advisory Group
WHO – World Health Organization

STUDIES MENTIONED IN MANUAL

Project Name	Location	Institutions Involved
West Bengal Study	West Bengal, India	Development Impact Evaluation (DIME) at World Bank, International Finance Corporation (IFC), Development Research Group at World Bank
KePSIE Project<br>*Kenya Patient Safety Impact Evaluation*	Nairobi, Kenya	DIME at World Bank, IFC, Development Research Group at World Bank
MAQARI Project<br>*Medical Advice, Quality, and Availability in Rural India*	Rural India	Harvard University, Johns Hopkins University, Duke University, University of Toronto, World Bank
Qutub Project<br>*Quality of Tuberculosis Care Project*	Urban India (Delhi, Mumbai, Patna)	McGill University, World Bank, Johns Hopkins University, Institute of Socio-Economic Research on Development and Democracy
ZASP Study<br>*Standardized patients: An approach to understanding the realities of South Africa's TB cascade*	South Africa	McGill University; HIV/AIDS, STI, and TB (HAST) Program at the Human Sciences Research Council (HSRC) in Pretoria, South Africa
REAP study<br>*Rural Education Action Program*	China	Stanford University

SECTION 1. INTRODUCTION

In order to improve the health of the world's population, health systems must be designed to deliver health with sufficiently high quality. Delivering health to the benefit of populations involves not only monitoring and improving access and coverage of health services (the "quantities" of health services), but also ensuring that health services are delivered well (the "quality" of health services) and implementing quality improvement strategies when they are not. Otherwise, efforts to improve access and coverage will result in patients obtaining care with unknown and varied quality, which can result in more sickness and high financial and non-financial costs to patients, health systems, and economies. But first, what is high quality? High quality care refers to care that is safe, effective, patient-centered, timely, efficient, and equitable. However, understanding and improving levels of health care quality poses several logistical challenges. **This manual focuses on how to implement quality of care survey techniques in the context of low- and middle-income countries (LMICs).** This introduction section presents the purpose of understanding quality of care, summarizes various quality measurement techniques with an emphasis on the standardized patient methodology, and outlines the manual.

Measuring quality in LMICs can offer responses and further discussion to the following types of questions: *What happens during a clinical visit between a patient and a health care provider? How is care provided to patients, and what is the quality of care provided to patients? What are the ways in which health care providers can improve their interaction with patients and improve quality of care?* These questions have proven very hard to answer in high-income countries, although considerable progress has been made using administrative records and chart abstraction. In low-resource settings, such administrative records and patient charts are either not kept or contain poor and inaccurate information. Consequently, quality measurements have relied on customized surveys of healthcare providers using a multitude of techniques. **This manual is written to guide implementers through decision-making processes that help identify the nuances, provide considerations, and lay down protocols to address critical aspects in implementing the standardized patient methodology.** The authors have been involved in several such studies, and because the method is being rapidly scaled up in diverse settings to measure quality, this manual aims to summarize the key steps towards successful standardized patient implementation based on lessons from previous studies.

Standardized Patients ("SPs" also known as "fake patients" or "mystery clients" or "simulated patients") are a methodological tool to help capture practice, processes, and services in the health sector. SPs are individuals recruited from local communities and extensively trained to present tracer health conditions to health care professionals at health facilities or pharmacies. The purpose of mimicking a real encounter in such a way between patients and health service professionals is to gather information on what type of interaction would occur in reality. Since real encounters are the byproduct of patient and provider characteristics and decision-making patterns, SPs offer a perspective into provider characteristics and decision-making patterns while keeping the patient standardized. Gathering this information across many providers has helped with understanding and improving quality of health care, patient safety issues, training outcomes, or policy implementation in different settings of interest.

After interactions with the health sector, SPs in research studies are trained to precisely debrief their encounter, and various aspects of each SP interaction are recorded in a structured questionnaire. The structured questionnaire is similar to patient exit questionnaires, which are surveys completed by patients after visiting health facilities. Information about the interactions captured in the SP structured questionnaires become analyzable data. Through design, these data can then assess a range of outcomes, such as health care providers' adherence to international and national guidelines for the health condition presented by the SP, as well as history questions asked, diagnosis given, diagnostic tests ordered, treatment dispensed or prescribed, referrals or clinical instructions, and observed adherence to patient safety issues, such as hand-washing prior to examination. Throughout this manual, examples from SP projects implemented in India, Kenya, China, South Africa, and other LMICs are provided for the purpose of helping teams implement the SP methodology in resource-limited, global health settings.

What can be achieved with SPs and how?

Examples of objectives for previous SP studies include:

1. To understand provider practice
2. To assess processes and behaviors occurring during a clinical interaction
3. To assess standards of care or policy implementation at a certain point of time
4. To implement monitoring or a surveillance system benchmarked in standards of care

5. To ensure high quality medical education through realistic scenarios that test and train health care professionals with face-to-face patient encounters

One of the first and main questions that SPs can help answer in low-resource settings is, "Who provides care?" For instance, in an SP study in rural and urban India, Das et al. find that in 63% of their SP interactions conducted at public health clinics, SPs were seen by an individual who did not have any medical training, despite governmental policy requiring these facilities to be staffed by trained health professionals (1). During the initial stages of study design, one question to ask is, "Is it important to assess policy on health providers who see patients?" Then, when designing the study implementation, different research questions can result in different logistical issues to work out. For example, the question, "Who provides care?" turns into a non-trivial challenge for the field team, which must then ensure that an SP actor sees the person intended – a consistent challenge across previous studies. How does a team implementing SPs decide whether to visit health facilities as walk-ins or with appointments to see specific health care providers? This manual will address considerations for these nuances.

SPs can also capture more complex dynamics in a patient's interaction at a health facility or with a health care provider. For example, more complex research questions in which SPs have been or can be used to help answer include:

- What is the average level of quality of care received by patients visiting providers, and to what extent does quality vary?
- What is the variation of provider practice in a given setting?
- What is the general situation concerning medicine and health care delivery?
- To what extent are diagnostic tests and treatments ordered for patients presenting with certain conditions?
- To what extent is a new or existing policy being adopted by a health sector?
- Do doctors treat male and female patients differently?
- What is the take-up of a new diagnostic technology in the health sector?
- What do physicians or pharmacies charge as fees?
- How much time do health providers spend with patients during patient visits?
- What is the extent to which unnecessary or harmful medicines are prescribed or dispensed?
- What are current levels of quality for drug dispensing or prescribing?
- Do health care professionals treat fully compliant or empowered patients differently?

Kwan A, *et al. BMJ Global Health* 2019; 4:e001669. doi: 10.1136/bmjgh-2019-001669

Why SPs? A variety of different methods are available to measure quality of care!

Across LMICs, several methods exist to assess quality of care, including vignettes, patient exit interviews, direct clinical observation, medical record or chart abstraction, as well as SPs. General descriptions of each method are provided below.

- **Vignettes** are interviews conducted with health care providers to understand the process of a patient-provider interaction. A vignette can be structured with a specific case, and providers can then respond with the type of questions they would ask the patient, tests they would order, medicines they would prescribe, referrals they would make, and any other further instructions they would give to the patient.
- **Patient exit interviews** are surveys completed by patients to learn more about their experience at the health facility on the day of visit. Exit interviews are often used to understand patient satisfaction measures and other facets of the patient's experience with the health provider, other health workers, and the facilities during the visit.
- **Direct clinical observation** techniques offer a way to capture actions, the sequence of actions, and the duration of those actions as done by health providers. These details are captured by directly observing the health provider or the environment in which health workers provide services to patients.
- **Medical record or chart abstraction** leverage the information captured in paper or electronic medical records, billing, drug prescriptions, or other charts to understand provider actions, such as tests ordered, medicine prescribed, and other aspects of patient management documented.
- **SPs** are recruited individuals trained to simulate standardized cases during presentation at a health facility or to a health care professional, after which they complete an exit questionnaire that provides details on the interaction.

Depending on the research questions of interest, any of these methods can be more or less appropriate to conduct; however, interpretation of results vary, and each method has a unique set of limitations (for more thorough discussion of budget requirements and considerations once quality of care methods and health conditions of interest have been selected for study, see Section 3.1). For example, when comparing vignettes to SPs, vignettes are better for assessing provider knowledge, and SPs are better at assessing provider practice. In the next paragraphs, the different methods are described

and compared against each other, and we explain why the SP method is the gold standard measurement tool to understand provider practice.

Reprinted and adapted from the Medical Advice, Quality, and Availability in Rural India (MAQARI) Project Manual (2), Table 1.1 summarizes the aforementioned quality measurement methods across their abilities to assess provider knowledge and practice, to provide limitations and biases when considering each method, and to outline illnesses that can be captured.

Table 1.1. Measures of quality, reprinted and adapted from the MAQARI Project (2)

Measure of Quality	Measures Knowledge	Measures Practice	Accounts for Case-Mix	Accounts for Patient-Mix	Limitations and biases when considering each method	Illnesses Covered
Provider Vignettes	Yes	No	Yes	Yes	By design: Vignettes measure the maximum a provider can do and is affected by social desirability bias. Very easy to include as a survey when research team is already conducting health facility surveys; however, vignettes can overestimate provider practice.	All
Patient Exit Interviews	No	Yes	Yes	No, but can account for service-mix	By design: Exit interviews measure the maximum a patient recalls and is affected by social desirability bias. Challenges exist with sampling patients and ensuring high rates of participation, and providers may not always refer to clinical or technical procedures by name with patients.	All; however, recruitment of patients is more difficult if research team is interested in rarer health conditions or patient characteristics.
Direct Clinical Observation	No	Yes	No	No	Direct observation is biased by Hawthorne effects; however, Leonard and Masatu (2007) show big	Limited in two ways: (A) "serious" illnesses like unstable angina will show up on a sporadic

					Hawthorne effects begin to decline with the time spent observing (3). Costs of paying enumerators to observe for long periods of time can be high and may not be relevant for rarer conditions. Ethical challenges exist for data collectors who observe bad or harmful practices being performed on real patients.	basis, and (B) the observer never knows what the patient actually has—and doctors frequently make incorrect diagnoses.
Medical Record or Chart Abstraction	No	Yes	No	No	Although appropriate in settings with very strong records, LMIC settings typically have incomplete, varied (paper, books, electronic), or no records in low-resource settings. Records if they exist also often only measure patients after a specific diagnosis is made and do not accurately or consistently reflect patient-provider interactions.	More relevant for provider actions that occur after diagnosis is made and for conditions where health records are kept. If records exist and are high quality, this method can be better for conditions where patients visit providers for multiple, sequential visits (e.g., chronic conditions)
Standardized Patients	No	Yes	Yes	Yes	Assesses one-time interaction (and not follow-up or subsequent visits) to a single facility or provider. More extensive planning, recruitment, and training costs, and significant capacity and skill in implementation is needed to address the details in conducting such a study.	Limited to (A) diseases that don't have any obvious physiological symptoms (which cannot be mimicked), and (B) conditions that don't require invasive exams—particularly in LMICs.

There are several reasons that may warrant implementing one of the above methods over the others. Because of this, it is critical to understand the differences across these methods when selecting the most appropriate quality of care measure to implement for your purposes.

- *How do vignettes differ from SPs?*

First, vignettes are easier and less expensive to implement than SPs, and have a wider breadth of illnesses that can be assessed. However, vignettes result in biased estimates if the objective is to understand actual practice. This is due to the know-do gap, which has been documented in a variety of settings for a variety of conditions and represents the difference between what providers state they would do in a hypothetical situation and what they actually do when presented with a real patient. In one study that took place in rural India, Mohanan et al. (2015) found that when presented with a child diarrhea case vignette, 21% of health care practitioners prescribed potentially harmful treatment; whereas, when an SP presented at the clinic and described a child at home with diarrhea, 72% of the same practitioners prescribed potentially harmful treatment (4). Similarly for adult illnesses, Das et al. (2015) reported in their study on tuberculosis that 73% of providers in their sample ordered a chest X-ray or sputum smear microscopy test during the vignette interview, but only 10% did the same when an SP with a classic textbook case of presumed tuberculosis visited the provider (5). In summary, providers report actions based on their knowledge, which may be well measured by a vignette; however, there is evidence that demonstrates that what providers know often is different from what they practice.

- *How do vignettes or SPs differ from patient exit interviews?*
  Patient exit interviews offer a way to understand the interaction between the patient and provider. Where a vignette constructs a case scenario that is presented to the provider in an interview setting, the exit interview is a survey that contains questions for the patient usually at the end of the clinic visit. For these reasons, the patients' responses to exit interviews will reflect what patients recall or care about (e.g., user experience) and are less able to estimate more specific aspects of care that are relevant for quality. For example, a patient may know his blood was being drawn, but may not necessarily be able to report the specific blood tests that were ordered by the doctor or whether the test was recommended by guidelines or certain protocols.

- *It seems like all provider actions of interest can be observed, but what are the limitations of direct observation?*
  Direct observation, unlike provider vignettes and exit interviews, can provide data on provider practice, especially when the observer is trained to identify the actions of interest. Before selecting this approach to assess quality of care, one should consider

several notable considerations and limitations to direct observation. One main limitation of direct observation is: what patients actually have is unknown to observers. For this reason, direct observations therefore rely on general observable metrics of care that are assumed to be higher with better quality, such as consultation time or number of exams, which is not always accurate for quality measures, since consultation time and exams can be both unnecessary and necessary. Chan et al. (2012) in the MAQARI project field manual describe five other limitations to direct observation (2). To paraphrase, these include:

(i) The patient mix and the type of illnesses that different providers and facilities see may confound observed quality.

(ii) When patient-provider interactions are observed in medical facilities, the observer may not know the actual diagnosis, and this makes it challenging to know whether the provider actions were correct.

(iii) Rare events of interest will require long periods of observation. Individuals with, for example, less prevalent health conditions or conditions that do not require frequent visits will be observed less frequently as more common, less life-threatening illnesses, which suggests that the observation period to capture quality for the former conditions will be long (e.g., several weeks).

(iv) Having an observer trained to monitor providers' actions of interest offers an ethical dilemma in the moment providers make an incorrect or potentially harmful action. Should the observer step in and correct the provider?

(v) The Hawthorne effect, or the phenomenon where individuals alter their behavior when they are being watched, biases the results of direct observation. Since quality of care is effectually a measure of performance, the individuals who are to be observed may feel as though their job, salary, or reputation is on the line. This may result in the observed individual performing more of the actions they believe they should be doing and less of the actions they know they should not be doing. Further, when doing research and obtaining consent, the individual who is to be observed may decline consent.

- **What information do SPs give that cannot be captured from medical record or chart data?**

  Since medical records and charts are documents produced at health facilities, they can provide more clinically accurate information than exit interviews, because they do not rely on patient's ability to discern or remember clinical actions. However, there are several limitations that must be considered when interpreting data from medical records or charts. First, an assessment of quality of care cannot be made on actions that are not part of the medical record. Second, it must be assumed that all patients seen are entered in the record, and all information is an accurate representation of what occurred. Third, in many settings in LMICs, medical records simply do not exist, and when they exist, information is often sparse. In contrast, SPs are trained to recall specific aspects of the interaction with a provider and of the health facility of interest, and upon leaving the health facility, the SPs complete an exit interview that captures the pre-determined information that is desirable to collect.

- **Would the SP methodology be appropriate to implement in any setting?**

  The SP method also has current limitations, which should be considered before deciding to apply it in any setting or may warrant further methodological development. First, assessing child-related conditions requires detailed attention to address ethical considerations and appropriate precautions are taken. One example of the use of SPs with real children is published by Rowe et al. (2012; ClinicalTrials.gov Identifier: NCT00510679), which describes a quality improvement intervention assessment in Benin for the Integrated Management of Childhood Illnesses with real children accompanied by adult actors portraying caretakers (6). Second, conditions that may receive a potentially harmful procedure, regardless of whether it is an accurate procedure or not, require appropriate techniques to be put in place for SPs to feasibly avoid any risks. These will vary from setting to setting. As Chan et al. (2012) state in the MAQARI Project Manual, "Although invasive examinations do not preclude the use of SPs in medical education in high-income countries, in typical clinics in low-income countries, any kind of invasive examination (including the use of a thermometer) or treatment (e.g., injections) can result in a health risk to the SP" (2). Third, the SP methodology has not been used to conduct follow-up visits at scale. Understanding quality for certain health conditions or to ascertain some research questions may require information that captures the actions of a provider after the same patient has visited him

Kwan A, *et al. BMJ Global Health* 2019; 4:e001669. doi: 10.1136/bmjgh-2019-001669

or her several times. The Qutub project in urban India conducted a follow-up visit pilot in Delhi with three SPs, who were trained to return as fully compliant tuberculosis symptomatics to 19 providers for up to 3-4 visits each (not published). Although none of the SPs were detected in any of the visits, a follow-up study at-scale has not been conducted, and follow-up visits may not be appropriate for other health conditions in other settings without serious considerations (such as understanding the likelihood in which real patients return for a follow-up visit upon provider suggestion) and subsequent piloting to ensure SPs are not detected (7).

Purpose and structure of the manual

This guiding manual is written to support work with SPs, particularly in LMICs. The next section provides a list of relevant research literature discussing SP studies across different continents (Section 2). Then, information is provided on how to assess the feasibility of SPs, particularly given health conditions of interest (Section 3), followed by elements for designing an SP project (Section 4). The manual then provides a set of tools for conducting an SP study (Section 5) and outlines how training can be structured with descriptions of SP training experiences in different countries (Section 6). A pilot should be carried out before larger engagements with SPs are initiated. Thus, this manual then gives details of what should be considered before a pilot (Section 8) and guidelines for how to conduct a pilot (Section 9).

Data entry, programming, and analysis require detailed preparations and ongoing management. The manual provides guidelines and examples from different countries (Section 10). Fieldwork, which includes health provider or facility selection options based on specific questions of interest, monitoring, operations, and fieldwork outputs, is also detailed (Section 11). Given the SP methodology can be unfamiliar to certain audiences of the SP findings, a description of the types of audiences and arenas for results dissemination is provided (Section 12). The document concludes in Section 13.

SECTION 2. OVERVIEW OF LITERATURE

Although this manual and toolkit focuses on settings in LMICs, the content stands on the shoulders of the many efforts that have implemented or are currently implementing SP studies around the world. Before a feasibility assessment, project design, and project implementation occurs, it is important to learn about the different applications of the SP method, the variety of contexts, and what has been learned from SP projects and about the SP method. For that reason, it can be helpful to refer to earlier studies with SPs regardless of whether they were implemented in an LMIC setting. Table 2.1 organizes SP studies and other helpful publications by geography. For each publication, the table organizes the setting, health conditions assessed, and provides a summary (for bibliography see Table 2.1 References). The table is not comprehensive, but aims to serve as a helpful resource while providing a lens into the variety of SP work in the literature.

Table 2.1. SP studies and projects categorized by geography.

AREA	AUTHORS	YEAR	SETTING	HEALTH CONDITIONS	TITLE	STUDY TYPE	SUMMARY
Global	Bate R, Mooney L, Hess K	2010	19 cities in developing and middle-income countries	Essential medicines	Medicine registration and medicine quality: a preliminary analysis of key cities in emerging markets	Observational, Cross-sectional	This study tested and compared the quality of registered essential medicines (anti-malarials, antibiotics, anti-mycobacterials) to non-registered or those with an unknown registration status. "Covert shoppers" (n= unknown) were used to procure 2065 medicines from private pharmacies in a variety of middle-income countries. Registered medicines consistently demonstrated significantly higher quality (lower failure rate) compared to unregistered or unknown medicines, across all geographies and types of medication.

Davis DA, Mazmanian PE, Fordis M, Van Harrison RT, Thorpe KE, Perrier L	2006	Australia<br>Canada<br>New Zealand<br>United Kingdom<br>United States	N/A - review of methods	Accuracy of physician self-assessment compared with observed measures of competence: a systematic review	Systematic review	This review examined 17 studies comparing physician self-assessment to other forms of external observational assessment. The majority (13/20, 65%) found little, no, or an inverse relationship between self- and external assessment, suggesting that physicians have a limited ability to accurately self-assess.

Using Standardized Patients to Measure Health Care Quality

Fitzpatrick A, Tumlinson K	2016	Kenya Uganda (LMICs)	N/A – review of methods	Strategies for Optimal Implementation of Simulated Clients for Measuring Quality of Care in Low and Middle-Income Countries	Review	This study draws on experiences from studies conducted in public and private-sector facilities in Kenya and Uganda. The study highlights the benefits and challenges to using the SP method to assess quality of care. The SP design accurately measures actual provider behavior; however, SP studies face many challenges. SPs must be appropriate (accurately represent facility's clientele, have strong recall) and training protocols must be detailed to ensure SPs are standardized. Protocols around confidentiality and SP risk mitigation must also be developed and strictly adhered to. Other challenges faced by SP studies include complex ethical approval, extensive training of team members, and collecting data on prices within consultations. The authors conclude that the unique information gathered from SP studies far outweighs the various costs.
May W, Park JH, Lee J	2009	Global (not-specified)	N/A - review of methods	A ten-year review of the literature on the use of standardized patients in teaching and learning	Systematic review	This review examines and reports on 69 studies pertaining the use of SPs for educational purposes, commenting on whether they affect knowledge, skills, and performance of learners. The majority of "learners" Using SPs were in medicine and nursing, and SPs were most often utilized to teach communication and clinical skills. Most often, improvements were measured as changes in knowledge, skills or attitudes, but few reported on behavioral change (which could actually affect patient outcomes). Studies had generally weak research designs, with no RCTs and few used control comparison groups.

Kwan A, *et al. BMJ Global Health* 2019; 4:e001669. doi: 10.1136/bmjgh-2019-001669

Using Standardized Patients to Measure Health Care Quality

	Mesquita AR, Lyra DP, Brito GC, Balisa-Rocha BJ, Aguiar PM, de Almeida Neto AC	2010	Global (not-specified)	N/A – review of methods	Developing communication skills in pharmacy: a systematic review of the use of simulated patient methods. Patient education and counseling	Systematic review	This review examined 15 studies relating to the use of simulated patients in assessing or improving communication skills among pharmacists. The authors concluded that the SP methodology is under- and misutilized as an educational or training tool, as the vast majority of studies used the SP methodology strictly to assess behavior. This review did not attempt to examine the quality of pharmacist-patient communication reported within the studies.
Africa	Brown L, Tyane M, Bertrand J, Lauro D, Abou-Ouakil M, Demaria L	1995	Morocco	Family planning	Quality of care in family planning services in Morocco	Observational, Cross-sectional	This study examined quality of care at 50 national family planning service points in Morocco. 22 mystery (simulated) patients observed a total of 25 provider-client interactions in order to identify gaps and improve service delivery. Strengths of the service delivery included availability of equipment and supplies, training of personnel, and mechanisms to encourage continuous use; limitations included abundance of counselling materials and availability of contraceptive pills. Mystery clients did not present themselves as new or returning customers, which negatively impacted the ability to interpret quality of delivery that may differ in these two scenarios.
	Christian CS, Gerdtham UG, Hompashe D, Smith A, Burger R	2018	South Africa	Tuberculosis	Measuring Quality Gaps in TB Screening in South Africa Using Standardised Patient Analysis	Observational, cross-sectional	In this study, 143 SP interactions simulating symptoms of presumed TB were conducted to measure the quality of TB screening in South African primary health centers. Sputum tests and HIV tests were conducted in 84% and 47% of consultations, respectively. Other noted gaps in screening include providers not mentioning the importance of returning to receive test results (28%), and neglecting to inquire about household TB (54%).

Daniels B, Dolinger A, Bedoya G, Rogo K, Goicoechea A, Coarasa J, et al.	2017	Kenya	Angina<br>Asthma<br>Diarrhea<br>Tuberculosis	Use of standardised patients to assess quality of healthcare in Nairobi, Kenya: a pilot, cross-sectional study with international comparisons	Observational, cross-sectional	This study aimed to validate the use of SPs in Kenya and assess quality of care of several tracer conditions. 14 SPs presenting with either asthma, child diarrhea, tuberculosis or unstable angina participated in 166 health care interactions in 42 private and public clinics. Zero SPs were detected or exposed to unsafe conditions. Proper case management occurred in 53% of interactions (ranging from 10-80% across conditions), and there were no consistent differences in care between public and private sector facilities. However, results only represented "one-off" interactions with providers, instead of potentially longer-term or more frequent care received by "real" patients.
Dizon-Ross R, Dupas P, Robinson J	2015	Ghana<br>Kenya<br>Uganda	Health products (Prevention)	Governance and the effectiveness of public health subsidies	Observational, cross-sectional	In this paper, "mystery clients" were used to detect local agency concerns impacting subsidies for a preventative health product (bednets) by antenatal services. Service delivery with subsidies was found to be of relatively high quality in all areas – coverage for eligible participants (80%), extortion or bribery (1.4%), and leakage of products to the ineligible (4.7%). This is potentially explained by high levels of job motivation and accountability within healthcare workers. However, given the clear ineligibility of the mystery clients (all clients were male, whereas eligible persons must be female), the degree of leakage identified may have been underestimated.

Ezire O, Okekearu I, Adeniyi F, Faweya O	2015	Nigeria	Sexually transmitted infections	Analysis of Health Facility Based Barriers and Facilitators To Use Of Sexual And Reproductive Health Care Services Among Most At Risk Populations (MARPS): Evidence From A Mystery Client Survey In Nigeria	Observational, cross-sectional	This study used SPs (termed mystery clients) to assess facilitators and barriers to accessing sexual and reproductive healthcare services in Nigeria. SPs represented "most at-risk populations", including men who have sex with men (n=4), female sex workers (n=4), or injection drug users (n=4) who were seeking STI and HIV counselling and testing. A total of 327 consultations and interviews were completed with the SPs in 33 unique healthcare facilities. The primary outcome was the willingness of the SP to refer a community member to the facility, based on aspects of location, staff training, promo materials, facility management, privacy, healthcare worker attitude, and quality of services. Healthcare worker attitude, perceived quality of services, and availability of de-stigmatizing promotional materials were significant in an SP's willingness to refer the service, but there was no association found with privacy. There was no assessment of SP performance (in training or through measurement of detection).
Fitzpatrick A, McLaren Z	2017	Uganda	Malaria	The Impact of Public Health Sector Stockouts on Private Health Sector Care: Evidence from the Ugandan Antimalarial Market	Before- and after-trial	"Mystery shoppers" were used to estimate the effect of public sector drug stockouts on malaria drug pricing and quality in the private sector. 933 purchases were made from 452 outlets in 114 villages of Uganda. During times of a stock-out, prices of drugs were $0.75 higher for those that the shopper requested, and $1.01 higher for drugs that the vendor recommended to the shopper. No change was found in the chemical quality of drugs dispensed. The poorest and least educated customers appeared to drop out of the market, suggesting that high prices influence demand.

Harrison A, Wilkinson D, Lurie M, Connolly AM, Karim SA	1998	South Africa	Sexually transmitted infections	Improving quality of sexually transmitted disease case management in rural South Africa	Observational, cross-sectional	Syndromic case management of sexually transmitted diseases (STDs) was examined in 10 rural primary care clinics of South Africa using both simulated (44 visits) and real (49 visits) patients. 9% of simulated patients received correct case management, defined as receiving correct drugs, condoms, and partner notification cards. Appropriate drug treatment and counselling was received in less than half of interactions. For some indicators, simulated and "real" patients produced contradictory results (particularly regarding staff attitudes), although this may be attributed to real patients being biased towards more positive reviews ('courtesy bias').
Hetzel MW, Dillip A, Lengeler C, Obrist B, Msechu JJ, Makemba AM, et al.	2008	Tanzania	Malaria	Malaria treatment in the retail sector: knowledge and practices of drug sellers in rural Tanzania	Observational, cross-sectional	This study explored the quality of advice and treatment provided at private retail outlets dispensing anti-malarial drugs. 111 shops were visited by mystery patients trained on one of the following cases: (1) 2-4 month old child with fever and problems with feeding; (2) 2-4 year child with recurring fever, problems with feeding, diarrhea, and tiredness; or (3) adult with recurring fever, headache, dizziness and loss of appetite. Although quality of serve delivery was generally low, drug stores tended to provide superior quality of care as compared to "general stores". In drug stores, SPs had much higher odds of receiving the correct dosage or appropriate treatment (Odds Ratio=9.6), but at a much higher price (Odds Ratio=11.3). Nevertheless, general shops are an important first point of contact with the healthcare system.

Kwan A, *et al. BMJ Global Health* 2019; 4:e001669. doi: 10.1136/bmjgh-2019-001669

Kohler PK, Marumo E, Jed SL, Mema G, Galagan S, Tapia K, et al.	2017	South Africa	Sexually transmitted infections	A national evaluation using standardised patient actors to assess STI services in public sector clinical sentinel surveillance facilities in South Africa	Observational, cross-sectional	SPs were used to assess quality of STI delivery in 50 public clinics across 9 provinces in South Africa. In particular, data was collected on the number of essential STI services that the SP received – (1) if offered HIV test, (2) if provided condoms, (3) if counselled on partner notification, and (4) if provided correct syndromic treatment. A total of 195 SP interactions were recorded. 18.7% of SP interactions received all of the essential services, although this was significantly higher in men than in women (25.1% vs. 12.3%). The majority (67.1%) were offered an HIV test, but only 6.3% of men were counselled on male circumcision and only 26.3% of women discussed family planning measures. Only 4% of SPs were detected. The authors note that providers performed better in 2nd or 3rd visits from SPs, suggesting possible improvement as a result of the first exposure.
Mchome Z, Richards E, Nnko S, Dusabe J, Mapella E, Obasi A	2015	Tanzania	Reproductive health	A 'Mystery Client' Evaluation of Adolescent Sexual and Reproductive Health services in Health Facilities from Two Regions in Tanzania	Qualitative, observational, cross-sectional	Thirty-three health facilities providing youth reproductive health services in Tanzania were visited a total of forty-eight times by SPs. SPs either requested (1) condoms, (2) information on STIs, or (3) information on family planning. SPs struggled with poor signage and direction to the clinic, experienced a lack of confidentiality and faced negative attitudes from health care workers and receptionists. Health workers also demonstrated "paternalistic" attitudes and lacked knowledge regarding reproductive health. Because the health care workers were anonymous (i.e., names were not known), it was not possible to follow-up with them in order to assess level of training or knowledge pertaining to adolescent reproductive health services.

Rowe AK, Onikpo F, Lama M, Deming MS	2012	Benin	Diarrhea<br><br>Malaria	Evaluating health worker performance in Benin using the simulated client method with real children	Observational, cross-sectional	This study used both the SP method and Conspicuous Observation (CO) to assess performance indicators in healthcare workers in Benin who were a part of a quality improvement intervention (Integrated Management of Childhood Illness [IMCI] strategy). SPs were mothers (aged 24-43) and their children (under 5); mothers would report their children were demonstrating signs of uncomplicated malaria and diarrhea (fever, diarrhea, and one episode of vomiting). 54 visits were completed in 54 distinct health facilities, and SPs were detected in 3.7% of visits. Performance of healthcare workers was poor, with most failing to ask about diarrhea when it was not outright offered and thereby incorrect treatment of almost all diarrhea cases. Performance as measured by CO was positively biased when compared with SP measures, but this result was only significant for 5 indicators due to the relatively small sample size.
Tumlinson K, Speizer IS, Archer LH, Behets F	2013	Kenya	Family planning	Simulated clients reveal factors that may limit contraceptive use in Kisumu, Kenya	Mixed-method, observational, cross-sectional	In this study, 6 female SPs were trained to undergo a family planning counselling session in 19 public and private health clinics in Kenya. SPs presented a "preferred method" of contraception (oral contraceptive pill, injectable, or IUD) with 52 unique providers in a total of 134 visits. SPs reported excessively long wait times and lack of access to providers, being asked to pay additional informal fees, and receiving rude/disrespectful treatment. SPs were granted their preferred method in 90% of interactions (either directly or by referral), but several providers lacked knowledge surrounding the efficacy of contraceptive methods. Detection rates were not systematically measured, and only assessed by asking SPs about how confident they were in their performance.

Using Standardized Patients to Measure Health Care Quality

Tumlinson K, Speizer IS, Curtis SL, Pence BW	2014	Kenya	Family planning	Accuracy of standard measures of family planning service quality: findings from the simulated client method	Observational, cross-sectional	This study used the SP method as a gold-standard to test the validity of 3 other methods of quality assessment in the context of family planning services: (1) provider interviews, (2) client interviews, and (3) observation of client-provider interactions. The authors concluded that quality was consistently over-estimated when using non-SP methods of assessment, particularly in helping a client to select a contraceptive method, discussing side effects, teaching the client how to use the method, and telling the client when to return for resupply/follow-up. Conflicting data may be a result of client bias ('courtesy bias'), modification of behavior under observation (Hawthorne effect) or poor recall among clients. Providers were assessed for a single interaction, which may not necessarily reflect their behavior on all visits, and did not allow for measurement of behavior consistency.
Wafula F, Dolinger A, Daniels B, Mwaura N, Bedoya G, Rogo K, et al.	2016	Kenya	Angina<br>Asthma<br>Diarrhea<br>Tuberculosis	Examining the Quality of Medicines at Kenyan Healthcare Facilities: A Validation of an Alternative Post-Market Surveillance Model That Uses Standardized Patients	Observational, cross-sectional	This study aimed to validate the SP method as a post-market surveillance model for assessing and strengthening quality for medicines. SPs presenting with either diarrhea, asthma, tuberculosis or unstable angina visited 42 facilities in Nairobi, Kenya, resulting in a total of 166 interactions and 300 medicines dispensed. 60 of the 300 medicines were tested for quality, of which 10 (17%) did not comply with monograph specifications. Furthermore, 5 of these were given inappropriately (all for cases presenting with unstable angina). The authors note the small sample size and the potential for false negative tests as possible study limitations.

Americas	Adamo G	2003	USA	N/A - medical education	Simulated and standardized patients in OSCEs: achievements and challenges 1992-2003	Review	This study broadly discusses the use of SPs for medical education and assessment. It highlights strategies and best practices for SP recruitment and case development, including which health conditions best fit to the SP method and how to assess SP training characteristics. It postulates operational and research questions pertaining to the use of SPs including ethical concerns, level of training required, types of people that best fit the SP role, and how to maximize data quality. It discusses resource constraints, test security for medical education, proper data management, and the development of web-based resources and organization.
	Barrows HS	1993	USA	N/A - medical education	An overview of the uses of standardized patients for teaching and evaluating clinical skills	Review	This study provides an overview of SPs as they are used in medical education and training, where SPs are defined both as "simulated patients" and real patients trained to present their illness in a standardized fashion. There are many benefits to using SPs in this context, given that they are available at any time and in any setting, they are trained and prepared to receive sub-par care (avoids mistreatment of real patients), and they allow students to practice their skills in emergency or sensitive medical conditions. The study documents a list of physical findings that SPs can simulate (from abdominal tenderness to wheezing), and discusses the history and development of the SP method.

Garcia P, Hughes J, Carcamo C, Holmes KK	2003	Peru	Sexually transmitted infections	Training pharmacy workers in recognition, management, and prevention of STDs: district-randomized controlled trial	Longitudinal experimental (intervention)	In this study, 14 low-socioeconomic districts in Lima, Peru were randomized to receive either an STD training intervention (focused on urethral discharge, vaginal discharge, genital ulcers, and pelvic inflammatory disease) or a control (diarrhea training intervention). Trainings were offered to all pharmacy workers in the district and incorporated didactic lessons and interactive discussion. A total of 2223 workers participated, and were then assessed by standardized simulated patients at 1, 3, and 6 months after training. Trainings effectively improved pharmacy worker's practices pertaining to STDs, including significantly more frequent recommendations for condom use, and better recognition and management for all STD syndromes as compared to pharmacy workers in the control group.
Maynard Tucker, Gl	1994	Haiti	Family planning	Indigenous perceptions and quality of care of family planning services in Haiti	Observational, cross-sectional	Haitian housewives (n=unknown) were trained to seek out and present as "mystery clients" to family planning services in Haitian clinics. The SPs regarded the most important quality attributes to be information adequacy and competence of the provider. They uncovered deficiencies in services, including paternalistic attitudes of the staff, lack of competency of the provider, and lack of an informed choice.

Using Standardized Patients to Measure Health Care Quality

Peabody JW, Luck J, Glassman P, Dresselhaus TR, Lee M	2000	USA	Chronic obstructive pulmonary disease<br><br>Coronary artery disease<br><br>Diabetes mellitus<br><br>Lower back pain	Comparison of vignettes, standardized patients, and chart abstraction: a prospective validation study of 3 methods for measuring quality	Observational, cross-sectional	This study uses the SP methodology to validate the use of clinical vignettes to assess competence of physicians working in outpatient primary care clinics in the US. SPs presented to 160 physicians with either a "simple" or "complex" manifestation of lower back pain, diabetes mellitus, chronic obstructive pulmonary disease, or coronary artery disease (8 cases total). Physicians were later given the same case as a clinical vignette. Quality scores (given as a percentage of process criteria adequately met) were highest when using the SP method (76.2%), followed by vignettes (71.0%) and then chart abstraction (65.6%). Clinical vignettes consistently showed similar results to SP scores, indicating that in an outpatient setting, clinical vignettes are an affordable and effective option for measuring quality of care. The authors note that use of clinical vignettes is conditional upon their careful construction and quality criteria must be linked to evidence-based guidelines.
Planas ME, García PJ, Bustelo M, Cárcamo C, Ñopo H, Martinez S, et al.	2014	Peru	Family planning	Utilizing Standardized simulated patients to measure ethnic disparities in family planning services in Peru	Crossover randomized trial - protocol	This protocol describes a study which will evaluate whether Peruvian health providers contribute to ethnic disparities in the provision of family planning services by using SPs that present as either indigenous or mestizo (indigenous/Spanish descent). SPs report on the proportion of technical tasks completed in each group. This protocol discusses the case description in detail, as well as procedures for recruitment, training of SPs, randomization and blinding, and describes study tools and analytical techniques.

Using Standardized Patients to Measure Health Care Quality

Planas ME, García PJ, Bustelo M, Cárcamo C, Martinez S, Ñopo H, et al.	2015			Effects of Ethnic Attributes on the Quality of Family Planning Services in Lima, Peru: A Randomized Crossover Trial	Crossover randomized trial	This study used SPs to evaluate whether healthcare providers in Peru deliver differential quality of care to indigenous vs. mestizo patient profiles. A total of 702 consultations were conducted; 351 with both indigenous- and mestizo-presenting SPs. Both SPs requested contraceptive advice but differed in clothing, hairstyles, accessories, and other attributes characteristic to the two ethnicities. No differences were observed in the quality of care delivered to the two ethnic profiles, as measured by proportion of expected technical tasks (36.2% vs. 37.0%, p=0.23) and socio-emotional tasks (75.0% vs. 74.5%, p=0.59) performed by the provider. Likewise, no differences were observed in total amount paid, time spent with provider, or number of visits necessary to receive FP services. Because consent was waived and provider identities were not obtained, authors were unable to collect provider demographics or ensure that no provider was counted twice in the analysis.
van der Vleuten CP; Swanson DB<br>Swanson DB, van der Vleuten CP	1990;<br>2013	USA	N/A - medical education	Assessment of clinical skills with standardized patients: state of the art;<br>Assessment of clinical skills with standardized patients: state of the art revisited	Review	This review describes large-scale studies of the psychometric characteristics of SP-based tests used for medical education and assessing clinical skill. The major source of error in measuring clinical skill is due to variation in examinee performance from one assessment to the next whereas disagreements between raters or discrepancies between SPs playing the same role have less of an effect. The authors recommend several improvements for SP-based tests: (a) focusing on assessment of history taking, physical examination, and communication skills, (b) adoption of a "mastery-testing framework" for interpreting scores, and (c) developing improved procedures for using SP methods to determine pass-fail standards.

Asia	Chalker J, Ratanawijitrasin S, Chuc NT, Petzold M, Tomson G	2005	Thailand Vietnam	Tracer conditions	Effectiveness of a multi-component intervention on dispensing practices at private pharmacies in Vietnam and Thailand—a randomized controlled trial	Randomized Control Trial	In this study, SPs were used to assess the effectiveness of 3 interventions applied sequentially at urban, private pharmacies in Vietnam and Thailand: (1) enforcing regulations governing dispensing of prescription only drugs, (2) education, and (3) peer-review. SPs asked for either steroids or antibiotics. Each pharmacy (n=146) was visited by 5 SPs at baseline and at least a month after applying each intervention. In Vietnam, the number of illegal steroids and antibiotics dispensed, as well as the number of pharmacists who asked no questions and gave no advice was significantly reduced post-intervention. In Thailand, only dispensing of illegal steroids was reduced. The authors note that the differential success of the intervention could be due to country-specific contextual factors.
	Currie J, Lin W, Zhang W	2011	China	Flu-like symptoms	Patient knowledge and antibiotic abuse: Evidence from an audit study in China	Observational, cross-sectional	SPs presented with identical flu-like complaints in 229 interactions with physicians within large general public hospitals and township hospitals in China. Physicians received one of two patient presentations: a patient who was instructed to ask a question that demonstrates their knowledge of appropriate antibiotic use, and another who stays quiet. Patients who demonstrated knowledge of antibiotic use received fewer antibiotic prescriptions, had lower overall drug expenditures, and received more information from the physician regarding possible side-effects. The authors note the challenges in educating and empowering real patients to speak up, and suggest concurrent interventions such as placing posters about inappropriate antibiotic use.

Currie J, Lin W, Meng J	2014	China	Flu-like symptoms	Addressing antibiotic abuse in china: An experimental audit study	Observational, Cross-sectional	SPs presented with "flu-like symptoms" and further indicated one of the following "incentives or disincentives": 1) nothing was said about preferences, 2) patient requested antibiotics, 3) patient requested to buy drugs elsewhere, 4) both 2 and 3, 5) a "gift" (pen) was given at the start of the consultation, 6) patient displayed knowledge on risks of antibiotic use, or 7) 3 and 6. SPs visited 200 physicians in a total of 620 visits. Antibiotic prescription at baseline was 55%. When antibiotics were requested, this rose to 85% (if drugs were to be purchased onsite); when patients indicated they would purchase elsewhere, it dropped to 14%. Displaying knowledge of antibiotic practices reduced prescription by 20%. Offering a gift significantly improved service quality and reduced drug expenditure. Eliminating the financial incentive to prescribe had the largest effect on antibiotic prescription and eliminated the unwarranted prescription of harmful Grade 2 antibiotics.
Daniels B, Kwan, Satyanarayana S, Subbaraman R, Das RK, Das V, et al.	2019	India	Tuberculosis	Use of standardised patients to assess gender differences in quality of tuberculosis care in urban India: a two-city, cross-sectional study	Observational, Cross-sectional	This study used SP visits to assess whether gender differences in tuberculosis case detection occur because of provider practices. 24 SPs completed 2602 interactions (1900 male, 702 female) at 1203 private facilities in Mumbai and Patna, India. There were no differences between males and females in the percent of SPs correctly managed, nor in measures of medication use or laboratory testing. This result was consistent regardless of the provider's gender or qualifications, and within both cities. Results only reflect experiences of patients who were visiting a provider for the first time, and only private provider practices in wo urban areas of India.

	Das J, Chowdhury A, Hussam R, Banerjee A.	2016	India	Chest pain<br><br>Diarrhea (child)<br><br>Respiratory distress	The impact of training informal health care providers in India: A randomized controlled trial	Randomized controlled trial	This randomized controlled trial evaluated the effect of a training intervention for informal health care providers in rural India, who provide up to 75% of primary care services in this context. 72 training sessions to improve general primary care were provided over 9 months to 152 informal providers. SPs were used to assess clinical practice for common illnesses (chest pain, diarrhea, and respiratory distress). Training increased the likelihood of providers to adhere to condition-specific checklists by 15.2% compared to control-group providers. Case management improved by 14.2%, but still remained lower than doctors in public clinics. Training did not affect the use of unnecessary medicines or antibiotics. Results regarding quality of care cannot be extrapolated to conditions for which invasive tests are required (a general limitation of the SP methodology). Additionally, the sample of informal providers may have been influenced by selection bias.

	Das J, Holla A, Das V, Mohanan M, Tabak D, Chan B;<br><br>Das J, Holla A, Mohpal A, Muralidharan K	2012; 2016	India	Angina<br><br>Asthma<br><br>Dysentery (child)	In urban and rural India, a standardized patient study showed low levels of provider training and huge quality gaps;<br><br>Quality and accountability in healthcare delivery: Audit-study evidence from primary care in India	Observational, cross-sectional	Quality of care delivered by public and private providers in primary health care services or India were assessed by SPs presenting with one of 3 illnesses commonly afflicting low-income people. In the case of dysentery, the SP was a father or mother requesting medication for a child that was not present in the interaction. A total of 926 interactions were completed. Providers had very low levels of medical training (11% reported having a medical degree) and demonstrated large deficits in quality including low levels of history taking and examinations, and an emphasis on giving medications. Diagnosis was correct in 12.2% of cases, and a harmful treatment was given in 41.7% of cases. Case definitions were restricted to those that could minimize harm to the SPs (i.e., did not require invasive examinations) and so results may not generalize to communicable diseases.

	Das J, Kwan A, Daniels B, Satyanarayana S, Subbaraman R, Bergkvist S, et al.	2015	India	Tuberculosis	Use of standardised patients to assess quality of tuberculosis care: a pilot, cross-sectional study	Observational, cross-sectional	This study was the first to use SPs to measure quality of TB care in Delhi, India. 100 healthcare providers (allopathic, alternative, and informal providers) were recruited and consented to receive one of four cases presenting with tuberculosis, for a total of 250 interactions. Cases differed by description and presentation (TB symptoms, TB symptoms + previously treated with antibiotic, TB symptoms + positive sputum smear report, TB symptoms + positive sputum smear report + suspicion of drug-resistance). This research aimed to validate the SP method in assessing TB care and assess care using national standards. Detection was low (5%) and measures of SP skill (recall as measured by audio recording). On average, 35% of essential checklist items were conducted, and 21% of cases were correctly managed. Post-study knowledge surveys revealed that provider knowledge is much better than provider practice (e.g., knowledge was 63% higher than practice for ordering a chest X-ray or sputum test). Results from this pilot study may not be generalizable across India, or between the private and public sector. Additionally, assessment of medication dispensing practices was challenging given the magnitude of loose/unlabeled pills.

Using Standardized Patients to Measure Health Care Quality

Kwan A, Daniels B, Saria V, Satyanarayana S, Subbaraman R, McDowell A, et al.	2018	India	Tuberculosis	Variations in the quality of tuberculosis care in urban India: A cross-sectional, standardized patient study in two cities	Observational, Cross-sectional	This study used SPs presenting with 4 TB case scenarios in order to assess and compare the quality of TB care among by private sector providers in two cities of India. Specifically, quality of care was compared between providers with an allopathic degree (i.e., MMBS) vs. an alternative, minimal, or no degree. A total of 2,652 SP-provider interactions occurred in 1,203 health facilities over the study period. Overall, TB cases were correctly managed 37% of the time, but unnecessary medicines were given in nearly every interaction. The odds of correct case management were 2.80 times higher for MBBS-designated providers compared to non-MBBS providers. Nevertheless, a wide range of quality was observed within each stratum of qualification. Like most SP studies, this design is limited in its ability to assess care or practices reflecting more than a single interaction with the healthcare system.
Lu F	2013	China	Endocrine and cardiovascular disease; insured vs. uninsured	Insurance coverage and agency problems in doctor prescriptions: Evidence from a field experiment in China	Observational, Cross-sectional	Research staff posed as family members of patients and presented doctors with lab reports pertaining to 2 patient profiles: (1) elevated triglycerides, high blood sugar and high blood pressure, or (2) hypertension. They also indicated whether they had government insurance and if they intended to buy medicine from the doctor (incentive to prescribe) or elsewhere (no incentive). Patient 1 and patient 2 were "received" by 100 and 96 doctors, respectively, across 25 hospitals in Beijing. When incentivized, doctors prescribed 43% more drugs to insured vs. uninsured patients; when there was no incentive, the prescribing practices were equal. Analysis suggested that 80% of the increased drug expenditure under insurance coverage was motivated by doctors' financial interest rather than an improvement in patient welfare.

	Miller R, Goodman C	2017	India	Diarrhea<br><br>Tuberculosis	Do chain pharmacies perform better than independent pharmacies? Evidence from a standardised patient study of the management of childhood diarrhoea and suspected tuberculosis in urban India	Observational, cross-sectional	Pharmacy chains in India are a growing phenomenon, but the quality of care provided has thus far been understudied. This research examines the quality of chain pharmacies in Bengaluru Using SPs that present with either diarrhea for an absent child, or suspected TB, and compares this quality to private independent pharmacies. From a total of 333 interactions (103 in chain, 230 in independent), less than half properly managed TB or referred to a clinic or doctor, and as many as 17% offered harmful or unnecessary medications. Zero shops correctly manage diarrheal cases. In general, chain and independent pharmacies performed similarly, except chains tended to sell fewer "harmful" medications compared with independent shops. History-taking and advice-giving was seen to be of substandard quality.

	Mohanan M, Vera-Hernández M, Das V, Giardili S, Goldhaber-Fiebert JD, Rabin TL, et al.	2015	India	Diarrhea<br><br>Pneumonia	The know-do gap in quality of health care for childhood diarrhea and pneumonia in rural India	Observational, cross-sectional	Quality of care for two prevalent childhood illnesses were assessed using both vignette interviews (provider knowledge) and unannounced SPs (provider practice). A total of 340 interactions took place, with 178 providers receiving cases of childhood diarrhea, and 162 receiving an SP presenting with childhood pneumonia. 80% of providers included in the study were found to have no formal medical degree. The correct treatment for diarrhea (oral rehydration salts, ORS) was never offered to an SP, and offered only 3.5% of the time when presented with a clinical vignette. Additionally, potentially harmful treatments were offered to SPs 71.9% of the time. The correct treatment for pneumonia was offered to SPs by 13.0% of providers. The authors note that children themselves did not present to the providers, but were represented by proxies (parental units).

	Satyanarayana S, Kwan A, Daniels B, Subbaraman R, McDowell A, Bergkvist S, et al.	2016	India	Tuberculosis	Use of standardised patients to assess antibiotic dispensing for tuberculosis by pharmacies in urban India: a cross-sectional study	Observational, cross-sectional	This study utilized SPs presenting with one of two TB cases (Case 1 = pulmonary TB symptoms; Case 2 = microbiologically confirmed pulmonary TB) to pharmacists working in 3 Indian cities (Delhi, Mumbai, and Patna). Correct case management was defined as referral to a healthcare provider and no provision of either antibiotics, steroids. 622 pharmacies were sampled and a total of 1200 interactions were completed. Correct management occurred in 13% of Case 1 SPs and 62% of Case 2 SPs. Similarly, antibiotic use was lower among Case 2s (16% vs. 37% in Case 1). No pharmacists actually dispensed anti-tuberculosis drugs, although 38% dispensed either an antibiotic or steroid which could cause TB diagnostic delay. Differences in quality care for both cases were almost entirely attributable to the difference in referral behavior. This study was not able to uncover reasons for inappropriate or incorrect practice, and does not reflect practices of pharmacists in rural areas.

Sylvia S, Shi Y, Xue H, Tian X, Wang H, Liu Q, et al.	2015	China	Angina<br><br>Dysentery	Survey using incognito standardized patients shows poor quality care in China's rural clinics	Observational, cross-sectional	Four SPs were used to measure quality of healthcare (more specifically, clinical skill as they pertain to dysentery and angina) in rural China, with the aim of assessing recent health care reforms designed to expand access to quality care in rural areas. 82 interactions were completed in 36 village clinics and 12 township health centers within 6 counties of southern Shaanxi province, where income and life expectancy are among the lowest in the country. SPs were recruited from the sample counties. Across both conditions, correct or partially correct treatment was provided 53% of the time, but a fully correct diagnosis occurred in only 26% of interactions. On average, clinicians spent 1.6 minutes consulting with patients. No clinicians addressed every essential checklist item. The authors note that standards of care reflect western models which may not be appropriate for providers that specialize in traditional Chinese medicine.
Sylvia S, Xue H, Zhou C, Shi Y, Yi H, Zhou H, et al.	2017	China	Tuberculosis	Tuberculosis detection and the challenges of integrated care in rural China: A cross-sectional	Observational, cross-sectional	The objectives of this study were threefold: (1) assess the ability of rural Chinese healthcare practitioners to detect and refer TB patients, (2) determine measurable differences between provider knowledge (competence) and practice, and (3) evaluate initiatives aimed at

					standardized patient study		integrating healthcare to make use of grassroots providers. Unannounced SPs presenting with classic TB symptoms conducted a total of 274 interactions in village clinics, township health centers, and county hospitals within 3 provinces of china. Overall, 41% of cases were correctly managed (using a "lenient" definition of a referral OR a chest X-ray OR sputum test), but 61.3% of providers prescribed antibiotics unrelated to TB. Provider knowledge surveys revealed case management rates that were 45% higher than actual practice, and antibiotic prescription rates that were 24% lower than actual practice. This study did not examine how providers would treat patients presenting with confirmed (through lab report) or recurrent TB (indicating potential drug-resistance).
Australia	Benrimoj SI, Werner JB, Raffaele C, Roberts AS, Costa FA	2007	Australia	Various	Monitoring quality standards in the provision of non-prescription medicines from Australian Community Pharmacies: results of a national programme	Longitudinal Observational	SPs (called "psuedo-patients" in this study) were used to observe and measure behavioral interactions between Australian pharmacists and their patients during a period of implementation of a national program addressing standards of practice. A total of 7785 visits were conducted in 4282 pharmacies (64% of pharmacists received 2 visits, and 17% received a third visit) over a period of 3 years. SPs presented with 59 unique scenarios that were either symptom-based requests ("something for diarrhea") or product-based requests ("aspirin"), and interactions were audiotaped for assessment. Pharmacists were scored as "unsatisfactory", "satisfactory", or "excellent". At baseline (prior to the national program), 34% of providers were "unsatisfactory", and 23% providers were "excellent". Over time, however, these numbers improved significantly, with significantly more scoring "satisfactory" and "excellent".

Kwan A, et al. BMJ Global Health 2019; 4:e001669. doi: 10.1136/bmjgh-2019-001669

Europe	Beullens J, Rethans JJ, Goedhuys J, Buntinx F	1997	UK	N/A – review of methods	The use of standardized patients in research in general practice.	Review	This article reviews advantages and disadvantages of Using SPs for general practice and research purposes. The SP method overcomes many of the disadvantages that exist with direct observation, given that the presentation of the case is accurate, the SP's perception of the physician's behavior is accurate and reliable; SPs are believable; and detection is generally low. The main disadvantages of the SP method are that it is a time-consuming and expensive process, which limits the number of physicians that can be assessed. The SP method is also limited in assessing diseases or conditions which require multiple interactions with the healthcare system (as SP visits are generally limited to a single cross-sectional consultation).
	Cleland JA, Abe K, Rethans JJ	2009	UK	N/A – medical education	The use of simulated patients in medical education: AMEE Guide No 42	Review	This review provides an overview of the use of SPs for medical education. SPs present many advantages (availability, flexibility in range of clinical cases, willingness to undergo scenarios multiple times, ability to provide feedback) and disadvantages (high cost and human resource requirements). Critical to the successful implementation of the SP methodology is the recruitment of able, suitable and credible SPs. The review also discusses how to train and use SPs for teaching and assessment purposes, how to monitor SP performance, and compares the use of SPs in Europe and Asia.

Rethans JJ, Sturmans F, Drop R, Van Der Vleuten C, Hobus P	1991	the Netherlands	Diabetes<br><br>Diarrhea<br><br>Headache<br><br>Shoulder pain	Does competence of general practitioners predict their performance? Comparison between examination setting and actual practice	Observational, cross-sectional	This study aimed to compare the knowledge (competence) and practice (performance) of general practitioners in the Netherlands. 36 practitioners were unknowingly visited by covert SPs to assess performance under normal conditions, and then were later presented with the same cases under known, controlled "testing" settings and asked specifically to perform to the best of their ability in order to assess competence. Competence scores were consistently higher than performance scores, and the lowest scores were seen in the diabetic case. However, when calculating efficiency scores (number of "obligatory" or "essential" actions taken compared to number of total actions taken), physicians scored higher in actual practice (with SPs) than under testing conditions. This study did not study provider attitudes, and results may be influenced by selection bias given that only 31% of doctors approached agreed to participate.
Rutter PM, Horsley E, Brown DT	2004	UK	Abdominal pain<br><br>Headache	Evaluation of community pharmacists' recommendations to standardized patient scenarios	Observational, cross-sectional	In this study, a single covert research acted as an SP consulting with 28 pharmacists in order to examine pharmacist practices as they pertain to facilitating "patient self-care" and providing advice. 14 queries from the SP related to headache, and the other 14 related to abdominal pain. Pharmacists performed better (asked more relevant questions and provided more relevant advice) when counselling on abdominal pain than headache. However, the expected outcome (referral) occurred in only 7 of the consultations for headache (50%) and 8 of the consultations for abdominal pain (57%). Several pharmacists declined to participate, which the authors attribute to the fact that covert methods are not generally welcomed by participants.

Watson MC, Skelton JR, Bond CM, Croft P, Wiskin CM, Grimshaw JM, et al.	2004	Scotland	Vaginal candidiasis	Simulated patients in the community pharmacy setting–Using simulated patients to measure practice in the community pharmacy setting	Randomized Control Trial	This study describes the use of simulated patients to measure professional performance of community pharmacy staff. 384 simulated patient visits were conducted with pharmacy staff who were enrolled in a randomized control trial to receive an educational intervention aimed at modifying over-the-counter dispensing practices for treatment of vaginal candidiasis. Pharmacists in the study thought they detected an SP on 9 different occasions, of which only 4 corresponded to actual SP visits. Many pharmacists reported apprehension in participating, but the majority (82%) agreed that the SP method was an acceptable research method for the community pharmacy setting. Furthermore, 34% reported previous experience of SP visits, 20% of which were for research purposes. This study does not report on outcomes of the RCT.
Wind LA, Van Dalen J, Muijtjens AM, Rethans JJ	2004	the Netherlands	N/A – development of an evaluation tool	Assessing simulated patients in an educational setting: the MaSP (Maastricht Assessment of Simulated Patients)	Validation study	The authors aimed to develop a valid, reliable and feasible tool to evaluate SPs, which are commonly used in medical education in Maastricht, the Netherlands. The objectives were to design and then validate the tool through interviews with students, teachers, and experts who are frequently involved with SPs. From these interviews, a written checklist with 21 total indicators assessing authenticity during the consultation and feedback after the consultation was created. Feasibility and reliability were investigated through 398 assessments and completed checklists. 12 checklists were required to reliably assess a single SPs performance.

SECTION 3. FEASIBILITY ASSESSMENT

3.1 Budget estimates and considerations

Costs of an SP project can vary based on the scale, scope, and complexity of the project. Section 3.1 will help (1) calculate back-of-the-envelope estimates for an SP project based on previous projects, and (2) create a budget and budget justification given a comprehensive list of considerations for a project in LMIC setting.

To calculate back-of-the-envelope estimates for an SP project, a sense of what costs can vary across projects can help. For consideration, costs per SP interaction are provided from projects in India, Kenya, and South Africa. First, in a multi-city study in India that took place between 2014-2017 with approximately 8000 SP-provider interactions, initial SP interactions cost approximately US$150 per interaction; however, after three years of the project, the field team became more efficient in data collection operations and more familiar with the work environment. Additionally, the fixed costs from initial training were divided across many more interactions. Because of this, the "per SP-provider interaction" cost decreased to approximately US$60 by the end of the three-year project period. However, studies in different countries will have different fixed costs, which can make the per interaction costs vary. In a 2018 SP study in three regions of Kenya (n=468 interactions), budget estimates per SP interaction were US$150. What is included in both the India and Kenya project estimates are: SP training and per diems, supervisor and SP salaries, fieldwork and transportation costs, costs of the interaction (including provider consultation fees, costs from purchasing medicines prescribed/dispensed during the interaction), and survey programming and data entry costs (including server where applicable).

The per interaction cost estimates also can vary widely with: (1) the size of the SP team and supervision staff, which will primarily depend on how many SPs are needed to reach desired sample sizes; and (2) project scale, since a team that has implemented the SP method and is tasked to conduct a study at a large scale can learn quickly about how to make the fieldwork progress efficiently without sacrificing quality of the data. For the two examples from urban India and Kenya, the following costs are excluded: out-of-country principal investigators, project staff, and travel costs. These have been left out because the project duration and other research activities of the two projects differ, and when budgeting for an SP study, the team will want to

add those in as appropriate for the project. As an example of how those costs can change per interaction cost estimates: a smaller study in South Africa (estimated n=400) that budgets for in-country investigators and partner costs comes to US$900-1000 per interaction.

When it is time to create a budget for an SP project, the following is a near-comprehensive list of aspects to consider:

- Human resources (see Section 5.7 for staffing requirements)
  - Field team: project manager, senior supervisors, junior supervisors, and SPs
  - Research team (if applicable): principal investigators, co-investigators, research manager, data analysts, consultants
  - Data team: programmers and data quality assurance support (for electronic data collection), data entry officers (for paper-based data collection)
- Technical Advisory Group meeting costs for SP study and case design
- Pre-training field visits, including communication and transportation costs
- Training costs, including supervisor and SP per diems, and refresher training costs
- Fieldwork
  - Fixed costs: communication, stationery, computers or tablets, audio recorders for verifying SP recall (Section 5.6)
  - Recurrent costs: consultation fees and diagnostic test and medicine costs
  - Room and board budget, including internet costs, for field team
  - Local transportation and per diem costs for supervisors and SPs
- Travel costs for research team to conduct SP design, fieldwork, and dissemination
- Secure server and hosting fees for data storage and transmission

It is important to provide the assumptions in the budget, or prepare a budget justification to complement the budget. The budget justification should follow the same sequence as the budget. Please see Annex A for a sample budget and budget justification templates.

3.2 Review relevant regulation

SPs can be recruited to visit health care providers and health facilities, including hospitals, pharmacies, and laboratories. Country regulations related to hiring people will have human resource and legal implications. For example, when reviewing the regulation for hiring people in a country, it is important to consider the maximum length of fixed-term contracts and

minimum wages. A good resource for recent labor market regulation across countries is available with the Doing Business Project.

If the SPs are to work in a different country, it is often easier to contract them through an entity registered in the country. That entity would be responsible for complying with labor regulation. It is also important to consider the geography of work to assess if there will be work in protected areas or areas with security risks requiring special approvals.

If the SPs are engaged for research purposes, the regulation for research in the country must also be considered: what approvals are required, by whom and by when. Most countries require an approval by an institutional review board (IRB) or an independent ethics committee (IEC). The IRB approves, monitors, and reviews biomedical and behavioral research involving humans. Studies with SPs can be considered behavioral research and generally require an approval from an IRB. Thus, IRBs that have approved studies with SPs have often motivated the approval with the importance of the research because little is known about the area subject to the study.

3.3 Conditions or ailments appropriate for SPs

An important consideration with respect to the evolving SP method at this time is that the current method limits the types of tracer health conditions SPs can portray – thus, the design of cases must satisfy particular criteria, and those who are implementing the SP method are encouraged to push the boundaries of the methodology for the purpose of improving social welfare while exercising care in the needed delicacy of this type of work. In the following Section 3.4, we describe four broader aspects that are strongly relevant for implementing an SP study, but focus on health conditions in this section.

Four aspects must be considered when assessing whether a certain health condition can be warranted for study under the SP method. First, conditions with obvious symptoms that cannot be mimicked by an otherwise healthy adult generally have demonstrated challenges for implementation; however, they are not impossible as Rowe et al. (2012) exhibit one study that has successfully and ethically assessed childhood illnesses with adult actors and real children (6). Second, cases have to be chosen so that the likelihood of invasive examinations is minimized, and appropriate techniques are devised to avoid invasive examinations, if offered. Thus, a number of gynecological and obstetric conditions cannot be used as tracer conditions.

Third, tracer conditions must have salience in the local context. For example, the incidence of cardiovascular cases and respiratory cases has been on the increase in India, and to improve clinical management and care for individuals with these conditions, we must understand current levels of care quality. Fourth, the ability to compare the results with those from other countries can allow contextualization of quality of care within a broader context and later influence global health policy, as well as enhance the discussion for national policy.

It is recommended that tracer conditions that have been successfully implemented in other countries be used to test suitability of the SP methodology in a different region for the first time. Health conditions that now have been implemented in several countries by authors and advisors of this manual are (A) unstable angina in a 40-45 year old male (8, 9), (B) asthma in a 20-25 year old male and female (8, 9), (C) a classic case of suspected tuberculosis, as well as cases carrying a sputum-smear microscopy test result positive for TB, and presumed multi-drug resistant case in a 30-35 year old male and female (5, 7, 10, 11), (D) pneumonia in a child who requires antibiotic treatment and who is accompanied by a father who reports respiratory distress (4), and (E) diarrhea or dysentery in a child who is sleeping at home and whose relative has come to the clinic to obtain medication (4, 8-10). These conditions are listed in Table 3.3.1, along with project names, locations, and encountered challenges. Further, a valuable resource on these and other health conditions that have been assessed with SPs is Section 2. There, multiple studies focused on pharmacy dispensing, family planning, malaria and sexually transmitted infections detail the circumstances in which SPs are a suitable methodology, as well as implementation challenges (11-15).

After making the decision on whether certain health conditions of interest are appropriate to implement with the SP method, there must exist protocols or guidelines that can provide the conditions to state what levels of quality of care are expected or sufficient in order to assess quality of care. For example, international recommendations (e.g., World Health Organization guidelines or recommendations), national guidelines, essential practice policy, quality of care standards or performance indicators can serve as benchmarks for determining levels of quality for a tracer condition of interest. Additionally, health assessment scales and indices may exist on quality of care for health conditions of interest. These guidelines can complement the formation of a Technical Advisory Group to advise the SP work (Section 5.4).

Table 3.3.1. Snapshot of conditions already assessed with SPs in quality of care projects.

Tracer Condition	Example projects (locations)	Challenges and limitations	Proposed resolutions
Cardiovascular diseases: Angina, Myocardial infarction (MI)	• West Bengal study (India)<br>• MAQARI (India)<br>• REAP (China)<br>• KePSIE (Nairobi, Kenya)<br>• Qutub (urban India)	Angina or MI involves irregular heart and pulse rate which SP may not imitate at the facility	SP with a family history of Cardiovascular diseases can be used
Asthma	• West Bengal study (India)<br>• KePSIE (Nairobi, Kenya)<br>• Qutub (urban India) to assess spillover effects	Asthma is more common among children. SP cannot show active symptoms.	SP with a child at home who has exhibited asthma symptoms or use of another quality of care measure because SPs cannot be children.<br>For the adult asthma case, an SP can describe the asthma incident that happened the previous day.
Diarrhea, Dysentery	• MAQARI (rural India)<br>• REAP (China)<br>• KePSIE (Nairobi, Kenya)<br>• West Bengal study (India)<br>• South Africa	The SP cannot bring a child displaying symptoms of diarrhea to the clinic. It may not be realistic that parent go without child in some societies.<br>Loose pills are often used which is difficult to identify.	Was considered unrealistic to have parent leave child at home when going to clinic in South Africa. Decided to develop a case of an adult coming to clinic to ask about sick niece living in a region known for poor water quality.
Tuberculosis	• KePSIE (Nairobi, Kenya)<br>• QuTUB (urban India)<br>• ZASP (urban South Africa)	Patients are often asked to come back in a few days after prescribed treatment. Completing the loop of care is challenging for the same SP-provider combination. The SP risks detection if sent back to the same clinic with test results and without symptoms<br><br>In countries where TB is likely to be related with HIV as co-infection (and in countries where the health systems are designed to detect TB patients when they test HIV positive), an SP cannot be trained to take an HIV test.<br><br>Cannot capture important outcomes such as adherence to TB treatment.	Different SPs with different treatment history and disease severity

Important questions to ask when selecting a health condition for an SP study:

When selecting health conditions for SPs to exhibit, critical questions to ask and aspects to consider include:

- What is the experience of real patients with these conditions, and how do these patients maneuver through the existing health system? Relatedly, would an SP be able to mimic the aspects of these patient pathways to provide accurate data for the study of interest, especially without straying from typical patient profiles providers see?
- How frequently do the providers of interest see real patients as walk-ins with these conditions?
- Should the patient bring diagnostic reports for the interaction with the provider? How can these reports be reproduced?
- Is there a need to display physical symptoms if the healthcare provider requests?
- Do providers ask for a follow-up visit for these conditions? If so, since follow-up SP visits have only been piloted and have not yet been conducted at a large scale, the SP method may not be the best for answering research questions related to quality of care in these circumstances. This may also be an opportunity to conduct a validation study to see whether follow-up visits are possible for a given context.

Notably, when considering the use of SPs for a new health condition or for a complex project, such as one in which SPs are used for continuous monitoring or quality of care surveillance for interventions, a pilot study with SPs is strongly recommended for several reasons. First, a pilot study can provide an opportunity for both the management team and the field team to learn aspects of the environment, which may be critical for success but at a time with lower stakes. Second, a pilot study will provide insight into logistics and proper sequencing of SPs across health facilities or providers in order to minimize the risk of SP detection.

3.4 Information about patients, providers, and the healthcare market

When SP data collection begins, one of the big priorities for the entire team is to ensure that SP detection is low. For this reason, it is important to conduct field visits and to gain an understanding of patients, providers, and the healthcare market ahead of data collection to answer the question: **Does it make sense for health providers or pharmacists to receive a patient like the SP?**

The process of conducting field visits and analyzing the health market will inform the decision if the use of SPs is feasible. This information will also be critical when designing the SP cases. Here are some questions to consider with explanations for why the answers are important for assessing feasibility of implementing an SP study.

Critical questions to ask when assessing if the SP method is feasible in a health system:

- **What fraction of patients is usually familiar to the doctor (urban, semi-urban, rural)?** This is important to decrease the risk of SP detection. For example, in rural areas, health care providers will know a high fraction of the patients, since many of them will come from the same area and have lived there for a long time. If an SP visits, the case portrayed by the SP (and the SP's training) will have to encapsulate rationale for why and how the patient arrived at the provider's clinic.
- **Do patients who visit the clinic speak specific languages or dialects?** If yes, the team will need to recruit SPs who speak those languages and dialects. These SPs will be critical for refining the details of case presentation at clinics before data collection begins (see Section 6.2 for developing the SP cases and Section 7.1 for integrating this process into the training of SPs). Additionally, the exit questionnaires may need to be available in those different languages or dialects.
- **Do patients who visit the clinic identify with certain ethnic, racial, indigenous, or tribal classifications?** If understanding quality of care by race, for example, is an important research question, then the study design, sample size and power calculations, and who is recruited and trained as SPs can accommodate this as a rigorous study.
- **Are there any risks to SPs with invasive procedures? What percentage of outpatients receives injections?** If there are many risks and/or if the percentage of outpatients receiving injections is far from zero, the risk posed to SPs hired for the potential study may not justify the study. Despite SPs successfully being trained to avoid injections in previous studies (see Section 7.3 for SP training techniques for risk mitigation strategies), if a health condition of interest almost always guarantees an injection from providers participating in the study, this will result in several consequences to address, including termination of the study due to ethical issues and increased risks to SPs and possible detection during attempts to avoid injections.
- **How are healthcare providers or pharmacists compensated? Is any compensation directly related to the sale of medicines?** Not only does the answer to this question

provide information on determinants of quality of care, it also informs the budget for an SP study, as SPs will reimburse providers and pharmacists.

- **Where do people purchase medicines?** Is there an in-house pharmacy or do patients travel to an independent pharmacist or chemist shop? Understanding the medicines that are dispensed or prescribed is an aspect of quality of care that may be desirable to the study team, and the answer to this question will inform processes to accurately purchase the medicines linked to any given SP interaction.

More important questions to ask when understanding the health system or market for SP study:

- What are the delays in detection and reasons for the delays in the current healthcare market?
- To which providers would patients generally go with this condition? Is it common among providers to refer someone with this condition, and to where do they refer?
- Are there any sets of rules for doctors who practice in both public and private sectors?
- What data exist on the doctors of interest? For instance, is there a central database on doctors where they are assigned, their qualifications, and when they joined, etc.?
- Are phone consultations allowed?
- Are there national and local guidelines for the conditions considered for the study?
- Is it common for patients to deny treatments from doctors (such as injections)?
- Do patients generally arrive at the providers' facility with some form of identification, such as an ID, driver's license, or insurance card?
- How do people choose the healthcare providers for outpatient services?
- Is there a system with appointments? Is there a waiting time?
- How do patients pay for services and medicines?
- What are issues that may occur during patient-provider interactions?
- What happens if a mother comes asking about a child who is sick but the child is not there?

3.5 Media and political considerations

Before initiating the project, all throughout the study duration, and after the study, it is important for study teams to be cognizant of media, social media, and political dynamics. This means being careful about when and how to discuss findings from the study with media and political stakeholders, since it can jeopardize the study. Being careful not to discuss the study or to jeopardize the identities of the SPs also extends to social media. Not being vocal or strategically

minimizing public discussion about the study both before and during will only protect the SPs and field team on the ground, which in turn protects the implementation of the study to achieve its objectives. It is advised that only after findings have been published should there be any attention directed at the study team and the study, and this should ideally and carefully be done through media releases and through close work between journalists and the study team. Deliberate attention to the media and political environment will immensely help protect the team, prevent any retaliation against the study which can affect the field teams, and help avoid any misconstruing of details that can harm the study and the staff involved at both the time of the study and any future implementation in the given context.

SECTION 4. DESIGNING AN SP PROJECT

4.1 Project Proposal

The objective of the project should be clearly defined in the introduction of the project proposal. The objective is generally to assess health care provider practices, such as quality of care, patient safety practices, medication use, pricing, and the like. The purpose may be to inform stakeholders who can influence the current healthcare services in a defined market, or to assess how healthcare provision changes as a result of an intervention or in response to differing case presentations. The proposal can be strengthened by arguing the importance of understanding and measuring quality of care, for which the SP method is the gold standard.

Importance of assessing quality of care

Many healthcare reforms focus on improving access to care (either through inputs, such as infrastructure, training of health providers, or demand-side interventions with increased insurance coverage), while the quality of the services accessed is often overlooked. Adherence to checklist guidelines is one of the key measures of quality of care. As briefly described in the introduction, the method considered as the gold standard to assess provider practice and quality of care is the SP method. Successful SP studies require the development of checklist items and standardized answers to questions that the providers may ask. The foremost criterion is that the items in the checklist must cover aspects of care that a provider should complete to both diagnose the underlying illness of interest and rule out competing explanations. The key is that the SP case and scenario allow for differential diagnosis. Like other elements of SP study designs, these details should be documented in the proposal in advance of fielding the study, so that judgments are not affected by results.

4.2 Planning

The proposal should include an overview of the main activities and estimated timelines. Aside from the initial project conceptualization, examples of main activities in sequence are listed below. Each activity is explained in detail in the following sections.

1. Approval by ethics committee
2. Selection of technical advisors
3. Assurance of approvals (e.g., government approvals)
4. Recruitment of SPs

5. Mapping and selection of providers for study
6. Obtaining provider consent as required
7. SP case development
8. Training SPs and finalizing SP cases
9. Developing standards for data management and analysis
10. Finalizing plans and protocols for fieldwork
11. Data collection and analysis including treatment grading
12. Dissemination of results

4.3 *Frequently asked questions*

Is approval by an ethics committee required?

Institutional review boards (IRBs) or independent ethics committees (IECs) approve, monitor, and review biomedical and behavioral research involving humans. Studies with SPs can be considered as behavioral research and generally require ethical approvals from an IRB or IEC. Publishing results from SP studies in certain research journals also require the statement of ethics, including study review numbers and IRB/IEC institutional names and locations for reference to demonstrate ethical conduct. However, provisions such as informed consent may not apply to the individual health care providers observed during the study, as in the case of a study with significant public health implications for which consent can be obtained from an appropriate official. Similarly, as the SPs themselves are usually employed in the same fashion as other survey enumerators, they are not typically subject to IRB restrictions beyond typical occupational safety concerns, which are covered in this manual. All studies reviewed in preparation of this manual have received approvals from institutional review boards. Section 5.2 provides specific details on the submission and approval process for IRB and IECs, as well as further resources on the ethical discussion surrounding the use of the SP method.

What is in the SP exit interview?

The SP exit interview can include any elements or outcomes of interest in which the field team can note and the SP can identify during the visit and recall after the interaction. The SP exit questionnaire from the Qutub and KePSIE projects in India and Kenya, respectively, contained the following sections:

- Cover page with form number; facility ID; provider ID; provider, facility, and visit details; date, start and end time of interaction; number of patients in the waiting room at arrival and departure; other details on the characteristics of the visit

- History questions asked by the provider (case-specific and enumerated with "other" option)
- Any clinical or physical examinations attempted (generally not case-specific and enumerated with "other" option)
- Diagnostic tests ordered (case-specific and enumerated with "other" option)
- Whether diagnosis (and details if mentioned), referral (and details if specific), and "return to provider" instructions (and details) were provided
- Medicines prescribed and dispensed, including price, quantity, place of purchase, and ATC code when possible
- Vouchers, coupons, subsidies, discounts, or incentives received, particularly when specific health programs are being assessed
- Global assessment of the provider and the clinic: quality markers (such as "did the provider use a cell phone during the interaction") and subjective judgments ("do you believe this provider created a private environment for your interaction")
- Prices charged for consultation, labs, and medicines, itemized when possible and aggregated with notes when not

Who writes and develops the scripts for the SPs?

Developing a new SP case requires collaborative and interdisciplinary efforts. These efforts are also needed when adapting an existing case to a new setting, evolving a case into a closely related health condition, and "translating" a case to a new region in the same country or a new country altogether. For this, the key aspect is the "SP script" or "SP narrative", which is essentially the SP's identity. The SP script requires detailed development and the field team's fidelity to keep in mind and maintain the clinical presentation and the illness narrative, as well as the contextual presentation and discussion of highly personal health conditions in both private and professional settings. The process of developing the script is briefly described below and discussed in more depth in Section 6.

1. For the clinical presentation: Clinicians, researchers, policy makers, individuals who create the international and national guidelines for the health condition of interest, and the research team should review the literature, discuss the priorities arising from the context, and make an agreement on the main clinical outcomes of interest.
2. For the human narrative: Anthropologists and qualitative researchers can lead the script writing, and the nuances can be filled in and corrected in a series of exercises over time with the supervisors and SP recruits.

More specifically, the different stages in which various individuals who have key roles in writing and developing the SP scripts (in addition to the research team, previous projects, and the body of literature) are:

a Designing a new case: Technical Advisory Group, anthropologists, and SP recruits
b Adapting an existing case to a new setting or region: anthropologists, local clinicians, local experts, supervisors, and SP recruits
c Evolving a case into a closely related condition: anthropologists, clinicians, individuals who produce the international and national guidelines for the health condition of interest (critical for identifying the correct clinical presentation), and SP recruits (helpful in identifying the correct words to describe the physical sensations of the condition).

Kwan A, *et al. BMJ Global Health* 2019; 4:e001669. doi: 10.1136/bmjgh-2019-001669

SECTION 5. CONDUCTING AN SP PROJECT

5.1 Selection of conditions, study considerations, and scientific rigor

The design of a study with SPs depends on the ailments selected for the study. Section 3.3 provided general details on what to consider when selecting ailments appropriate for SPs. This section further discusses the process of designing aspects for SP portrayal given the selected health conditions and study context.

When selecting a condition for SPs to exhibit, there are important considerations to take.

- Delay in detection and reasons for the delay
- Risk of invasive procedures
- Ability to reproduce diagnostic reports if relevant for the interaction
- Display of physical symptoms
- Providers seen for this condition, common referrals and location of the point referral
- Expectation for follow-up visits

It is recommended to select conditions for which there are international guidelines, standard treatment guidelines in the country, or performance indicators. Even if these are available, it is recommended to have a Technical Advisory Group with respected specialists who review the selected cases for SPs. See Section 5.4 for more details.

As stated earlier, even if the selected condition for the SPs has been used in other countries, a pilot study is strongly recommended when expanding to new geographies.

5.2 Approval of Institutional Review Board or Independent Ethics Committee

This section continues discussion from Section 4.3 on ethical approvals for SP study. SP study teams will also find a publication by Rhodes and Miller (2012) and the interpretation of existing guidance for patient safety research done by the World Health Organization in 2013 to be very useful for comprehensive ethics discussion on implementing the SP method (16, 17).

Different countries have different regulations about approvals for studies involving human subjects. Many countries require approval by an institutional review board (IRB), sometimes called an independent ethics committee (IEC), for studies involving human subjects. Studies

with SPs classify as human subject research as per the United States Department of Health and Human Services. Universities and larger healthcare providers can have these committees. The time for approval can differ significantly between different committees and it is advisable to find out the average time for approval before finalizing the application to a specific committee.

Many IRBs and IECs have not reviewed studies with SPs in the past. It is therefore recommended that a clear description of the methodology be provided. The application package should also include a motivation for why this methodology is preferred to other methodologies. It is important to clarify how the project is minimally intrusive, and why Using SPs will involve no more than minimal risk to participants (both SPs and providers). See Annex B for a description of the methodology for IRB submission that was used in the Qutub project in urban India.

Approvals from IRBs or IECs must be obtained in advance (before a pilot study is done). Substantial changes, including changes to the cases used by SPs, require an amendment to the approval.

How long does it generally take between submission and approval from ethics committees?

Depending on IRB and IEC meeting schedules, it may take upwards of three months to seek and obtain permissions. This will depend, of course, on several events, including: (i) whether the ethics committee has previously reviewed other research studies using the SP method, (ii) whether the study team seeks a waiver of provider informed consent, and (iii) whether the study team or other contacts have conducted a pilot study, and if there is a need to wait for data to be analyzed.

What to expect from an ethics committee?

- SPs are not a familiar topic among ethics committees.
- Ethics committees may require:
  - Confirmation and justification that there are no obvious or perceived risks to health providers who will be involved in the study, that doctors will receive their usual consultation fees as they would from any other patient in such setting (there is no economic loss for the doctor to participate), and that if the provider confronts them and challenges them, SPs are trained to reveal themselves as SPs. For projects that have informed consent from providers, ethics committees

will require that SPs be trained – to alert the provider that permission has been received from the provider and that the effort is a part of a project for which providers have been previously informed and have consented. For cases in which permission or informed consent has not been taken with IRB approval (for example in the case that a waiver of informed consent minimizes risks to detection and protects the scientific validity of the study), the ethics committees will require that when SPs are confronted by doctors who accuse them of being fake in any manner that could jeopardize the safety of the SP, the SPs have been trained to indicate that they are part of a research project and can provide a telephone number of a contact person or call in a supervisor who will be in the field.

- Confirmation and justification that the study does not pose any risk to real patients of the health provider (e.g., if clinics see on average 15-20 patients a day and the providers spend 3-5 minutes per patient as demonstrated in previous studies, an SP visit will not substantially add to the waiting time for any patients). Additionally, if a medical emergency occurs at the clinic, the SP, who is employed by the research team, is trained to immediately step aside.
- Confirmation and justification that the study does not pose any risk to SPs. The justification should include an actual or estimated SP detection rate, an explanation that the condition of interest does not evoke any invasive procedures that place SPs at risk, and confirmation that full debriefs and proper training will occur throughout the study to ensure that SPs are able to avoid all invasive examinations.
- A statement about the potential benefits of the study. For example, the study may serve to assess the usefulness and impact of the SP method to evaluate quality of care for the condition of interest which can inform policy and further the goal of reducing the prevalence, incidence, disease burden, or health costs related to the condition. The statement may also disclose that health providers in the study will not necessarily have any direct benefits.

Options for obtaining provider informed consent or a consent waiver

Necessary steps in research involving human research subjects include (i) obtaining ethical clearance from an ethics committee to ensure that the study is ethically sound and (ii) obtaining informed consent from human research subjects. If the SP method is being used for research,

research protocols submitted to IECs are required to state that clinic and/or provider informed consent will be obtained ahead of data collection. In case a consent form is required by the IRB or IEC, Annex C contains a template provider consent form from the KePSIE project in Kenya that may be adapted.

Depending on the research calling for the SP method, obtaining provider informed consent may jeopardize the study objectives. In their 2012 analysis commissioned by the United States Department of Health and Human Services, which assessed the ethical implications of SP studies, Rhodes and Miller state (16):

> *Several relevant considerations that both favor and oppose soliciting consent for simulated patient studies. Making research participation condition on informed consent protects the autonomy of research subjects and shields them from unreasonable exposure to research risks. However, scientific validity is also an important ethical principle of human subjects research, as the net risks to subjects must be justified by the value to society of the knowledge to be gained. The use of simulated patients to monitor access is a naturalistic and scientifically sound experimental design that can answer important policy-relevant questions, with minimal risks to human subjects. As interaction between researchers and subjects increases, however, so does the need for consent.*

The report concludes:

> *As long as adequate protections of confidentiality of research data are in place, minimally intrusive simulated patient research that gathers policy-relevant data on the health system without the consent of individuals working in that system can be ethically justified when the risks and burdens to research subjects are minimal and the research has the potential to generate socially valuable knowledge.*

For a waiver of informed provider consent in SP studies, it must be justified that the lack of participant consent is unlikely to adversely affect the welfare of the participants and that other methodological options have been carefully studied but cannot answer the research questions required. The investigators involved in the research must make an educated decision to select the SP approach after determining that the research questions of interest (e.g., on quality of care) can only be answered by Using SPs. Based on the experiences from either the research team conducting the SP study or other researchers implementing the SP method in a similar context, would the research team recommend any changes or adaptations to the method, such

as after a pilot? For example, based on the experience of the research team or others in a similar context, would the combination of greater spacing between the SP interactions (e.g., instead of sending an SP every day to a provider, an SP is sent once every two weeks) and a waiver of informed consent bring detection rates down? Is a health intervention being evaluated, and does that intervention bring doctors together often who could potentially discuss the SP study (e.g., during continuing medical education trainings) and share details on the physical characteristics of the SPs, which would bring detection rates up? It is worthwhile to think through the answers to these questions as they may justify the request for a waiver of informed consent.

Ethics committees that have granted a waiver of provider informed consent have granted the waiver conditional on an agreement with the research team that after the study is completed, all providers involved in the study will receive a letter of full disclosure. Such a letter should offer providers a chance to further discuss any aspect of the findings or methodology and register any concerns; however, given the confidentiality of research, no individual data on any clinic or provider can be disclosed. For example, the letter can provide a description of the study, the SP method, and a contact that providers can reach if they have any questions or concerns.

The Qutub project, which was a quality of care surveillance study in two cities in India, received IRB approval with a waiver of provider informed consent contingent on informing the providers who were sent an SP about the study upon its completion. In this context, the lack of participant consent was unlikely to adversely affect the welfare of the participants, yet obtaining provider consent would have increased the likelihood of SP detection, since the providers were a part of a network that would have frequent in-person meetings. Annex D contains a letter of full disclosure template from the Qutub project in urban India that can be used if a provider consent waiver was granted contingent on informing participants after the study completion.

Since submitting an SP study to an IRB committee for ethical clearance requires addressing the ethical considerations, Annex E contains the section on ethical considerations from the Qutub study IRB protocol submission. Based on findings from the Qutub pilot study (Das et al., 2015), the research team was able to demonstrate for the main study in two urban Indian cities that: (i) there would be no financial losses incurred by providers as the SPs would pay providers whatever they charged in their clinics; (ii) there would be no added inconveniences

to other patients as SPs would be trained to step aside if there was an emergency that demanded the provider's attention; and (iii) there would be only minor inconvenience to other patients as the average consultation times were low – usually three to seven minutes (5). The reader may also refer to the ethical considerations section and supporting text in Kwan et al. (2018) for a treatment of waiver of informed provider consent in the Qutub study (7).

Information that is relevant to include in the IRB/IEC submission packet:

- Rationale for using SP method over other quality of care methodologies
- Validation of SP methodology for condition(s) being assessed
- Higher level permissions if granted for the study (federal, state, city)
- Ethical considerations
- Provider consent form or discussion on waiver (if applicable), including letter of full disclosure
- Description of maintenance of confidentiality of the research data involving relevant mechanisms to protect confidentiality of participating health providers
- Drafts of exit questionnaires with SP scripts

Several ethics committees have approved studies with SPs with the motivation that the available evidence about quality of care is limited, and the results from the study are important to understand the current situation. This is important to keep in mind for projects in which the SP method is proposed as a monitoring function or surveillance system as such interventions go beyond a one-time, cross-sectional study.

5.3 *Registering an SP study with a rigorous experimental design*

Researchers leading an SP study with a rigorous experimental design (e.g., randomized control trial) will benefit from registering the study. Websites to register SP studies include:

- **ClinicalTrials.gov**
  https://clinicaltrials.gov/
  Note: certain medical journals will not publish results from a clinical randomized control trial (RCT) unless it has been registered on this website.

- **American Economics Association (AEA) RCT Registry**
  https://www.socialscienceregistry.org/

Note: Increasingly popular, the AEA RCT registry is for RCTs and impact evaluations in the fields of economics, political science, and other social sciences. It should not be used for clinical trials.

In randomized evaluation designs, the SP method can also qualify as a triple-blinded study when the following assignments are masked: (1) SP conditions are blinded from agencies implementing the intervention, (2) SP conditions are blinded from clinics and providers in the evaluation sample, and (3) the hired SP actors are blinded from the treatment assignment. One example of this is the evaluation described in Das et al. (2016) (9). In line with proper research transparency conduct that is becoming more common, researchers implementing a rigorous SP study are also suggested to draft a pre-analysis plan ahead of data collection that details the hypotheses and analytical procedures. One non-SP study that demonstrates the use of pre-analysis plans alongside an analysis is Casey et al. (18)

5.4 Selecting a Technical Advisory Group

Sections 3.3 and 5.1 identified the importance of having international recommendations, national guidelines, or performance indicators to guide the definition of quality of care and subsequently the aspects of the patient-provider interaction that the SP will report. Further, a Technical Advisory Group to advise on the SP work will be useful for solidifying the measures of quality of care. The composition of the Technical Advisory Group can have implications on the credibility of the SP method. The scripts for the SPs should build on the national treatment guidelines and reviewed by a Technical Advisory Group with authority and expertise on the health ailments of interest. One option is to select people that have developed or approved standard treatment guidelines in the country to become members for the Technical Advisory Group. It is recommended that the Technical Advisory Group include individuals in medicine, but also other disciplines. For example, in the Qutub project, economists facilitated discussion on rigorous evaluation techniques in the context of desired policy interventions, and anthropologists were able to shape discussion towards the personal presentation of patients, in addition to their clinical presentation, and aspects of the milieu in which providers operate and patients seek care.

It is important to have a short and clear project description to share with the invited members of the Technical Advisory Group. The invitation should preferably come from the Principal

Investigators, and the responsibility of members of the Technical Advisory Group should be clearly defined in the invitation. One of the main responsibilities of the group is to prepare the cases and guidelines for the SPs and discuss the exit interviews. These processes are critical for defining what data will be available to analyze.

It is suggested that initial drafts of the SP scripts are developed prior to the Technical Advisory Group meeting. The meeting can be used to discuss feedback and identify potential considerations for the work. The exit interviews can be developed during the meeting. There will be disagreement within the group, and it is important for the facilitator or study team to have a plan for how to handle disagreement between experts.

The SP scripts and exit interviews should be revised after the first Technical Advisory Group meetings. The revised scripts and exit interviews should be circulated to all the members of the group for any further feedback. It should be clear how long the members have to get back with any further feedback before the scripts and exit interviews are finalized.

It is advised to remain in close contact with at least one member of the Technical Advisory Group. This member can be available to review the experience and provide feedback after training of SPs and dry runs (i.e., practice visits conducted at the end of training and before fieldwork with real health providers or pharmacies). Many SP scripts and exit interviews have to be revised after these sessions, and having Technical Advisory Group members continuously involved is important to confirm that clinical aspects of the SP cases remain aligned with the study objectives. One or two members can work on behalf of the Technical Advisory Group to endorse the final version of the scripts and exit interviews.

The data from exit interviews can be used to "grade" the clinical experience. In other words, elements of the clinical experience – history questions asked, diagnostic tests ordered and not ordered, medicines prescribed and not prescribed – can be considered together or separately as appropriate, harmless, or harmful for the designed SP case, and the grading process, if relevant for the study, is an exercise to unpack these nuances of quality of care. It is suggested that members from the Technical Advisory Group be engaged to decide how providers should be graded. Section 11 discusses the process for grading medicines dispensed or prescribed during the SP interactions.

5.5 Government approvals and buy-in

Studies with human subjects may require government approvals in addition to approvals from IRBs and IECs. It is important to review the local laws, regulations and standards that govern human subject research prior to the study, and prior to any pilot study, with SPs.

> The International Compilation of Human Research Standards 2017 edition includes critical information for 126 countries: *https://www.hhs.gov/ohrp/sites/default/files/international-compilation-of-human-research-standards-2017.pdf*

Treatment protocols should be drafted or agreed upon by representatives within national or subnational entities to encourage in-country ownership. It is further recommended that the medical council affiliated with national or subnational health policy is involved for the development of treatment protocols.

Some governments are more likely to use research findings if they have been informed about the studies in advance. It can be good to identify the most effective ways to communicate research results with policy makers before the study starts. It is in some places good to establish a formal or informal process to keep representatives in government and other interest groups informed through the study period.

Annexes F and G contain study authorization letter templates from a national government and a local government office, respectively. Descriptions for each are as follows:

- **Study authorization letter template from National Government:** This letter introduces the study and the significance of the research to the national government. It is addressed to healthcare providers, both public and private, from the respective office of the national government (e.g., Ministry of Health) requesting participation in the research and contact information for more details. This letter is also used to request additional authorization letter(s) from local or regional government units in carrying out the study activities.
- **Study authorization letter template from Local Government:** This letter provides authorization from local or regional government authorities to carry out study activities. It is addressed to the research team and/or the national county government unit endorsing the study, with a copy to all local government health units and healthcare providers within that region. It is a supplement to the study authorization letter for the national government.

5.6 *Using audio recorders for SP recall verification*

One option to verify what occurred during the SP interaction is to have SPs wear audio recorders. There are both advantages and disadvantages to using audio recorders, and these should be assessed before deciding on whether to use them in the interactions.

What are the benefits of using audio recorders?

- To reduce the burden placed on SPs for remembering and accurately recalling what occurred during the interaction
- To help the project team verify what has happened in the field and compare it with what has been reported during the exit interview
- To listen to recorded test runs for supervisor and SP training purposes
- To check memorization and conduct accountability checks, especially for projects where fieldwork is long in duration: random selection of SPs throughout the course of the survey can be done to make sure they are doing the interactions with consistent fidelity to the SP cases

What are the disadvantages of using audio?

- Recorders can only pick up audio data.
    - In some experiences, this means that the history part of the interaction is usually audible, but other parts of the interaction that involve clinical or physical examinations cannot be accurately captured (e.g., when the patient makes an 'ahh' sound, the debriefer can assume a throat examination is done; however, differentiating between an auscultation and taking deep breaths cannot be done without asking the SP). For the medicine section, debriefers can identify which medicines were dispensed, but usually they are not able to tell which dosage matches to what medicine with audio only (e.g., while handing different medicines to the patient, the provider says, "Take this at night" and "Take this one three times a day."
    - Understanding the full interaction cannot be done with audio only and an exit interview in some cases would still have to be conducted. From the Qutub pilot in Delhi, there wasn't anything captured on audio that was not being captured normally with the exit interview, but the reverse was not true.

- Recorders must be hidden. Depending on the quality of the audio recorders, this can be challenging to do as they must be placed to pick up sound with high quality and definition without being noticed.

How to select a good audio recorder:

Recorders intended for recording during large conferences and lectures in large rooms often have settings that are good for cancelling background noises in health facilities. Additionally, audio recorders with noise cancelling features, voice-balancing features, and high-quality, cordless microphones are advantageous. Microphones that can attach by cord to audio recorders are not recommended, since some of the conditions the SPs reenact involve physical examinations (e.g., auscultations) and vitals being taken. During these types of examinations, the microphone cord can be discovered by the provider, which not only increases the risk of detection, but may lead to a premature termination of the interaction with potentially grave consequences if provider consent has not been obtained with a transparent description of the use of audio recorders.

What situations or cases would be good for audio recorders?

Audio recorders are good when the setting for the interaction is known to be quiet.

What situations or cases would be not as good for audio recorders?

Audio recorders are not so good for settings that are known to be noisy (e.g., with fans or televisions running, crowded spaces). For example, one can imagine that audio recordings of interactions with health care providers located in low-income bazaars that spill out into the street will contain a variety of noises (e.g., loud horns from passing trucks, street vendors selling products entering the soundscape), making it difficult to decipher what is being recorded and potentially rendering the audios useless.

What should the SPs know about audio recorders?

The play button in some audio recorders are designed to be easily pushed and can thus be more easily activated by accident. In the same vein, these audio recorders can easily be turned off when that is the opposite intention.

If the decision is to move forward with using audio recorders, the protocol can be found in Box 5.6.

Box 5.6. Protocol for using audio recorders during SP interactions

1. Exit interviews
- The cover page or supervision check section should have fields in the exit questionnaire form to help distinguish questionnaires entered from audio recordings or from recall (or observation). Examples of this are,
  - ☐ *Was an audio recorder used during this interaction?*
    → *Responses: Yes, No*
  - ☐ *How was the interaction done?*
    → *Responses: Recall (SP Memory), Audio, Observation*
  - ☐ *Audio recorder number:*
    → *[Space for recorder number]*

2. SP training on audio recorders
- During the initial stage of training, SP trainees should be advised that they might be conducting the interaction with providers with audio recorders. SPs should be made aware of the following objectives of audio recordings:
  - To reduce the burden placed on SPs for remembering what occurred during the interaction
  - To help the project team verify what has happened in the field and compare it with what has been reported during the exit interview
  - To listen to recorded mocks and learn and adjust
  - To check memorization, especially for projects where fieldwork is long in duration
  - To improve accountability
- Training on audio recorders can happen during general training. It is normal to expect a bit of anxiety from the trainees and SPs with the use of audio recorders. SPs should be trained on:
  - How to operate basic features (on/off/hold, recording)
  - How to turn on and off the mute option for beeping sounds
  - How to turn on and off the noise cancellation and voice balancer functions
  - How to save recordings in folders inside the device
  - How to charge the device with the built-in USB
  - How to download files

- How to conceal the audio recorder during visits to the clinic

3. Conducting dry runs with the audio recorders

- Each trainee should have at least 2 dry runs with the audio recorders.
  - Incorporate the audio recorders into the character. For example, in the Qutub study, female SPs found it best to put the recorders in a shopping bag that was made out of cloth (which is common). This solved the problem that a plastic bag would create, and it didn't require the female SPs to carry anything in their pockets.
  - Debrief – Debriefing on the experience is very important in general and also throughout the use of audio recorders. It is common that adding the audio recorders will make the SPs nervous. It is important for the project team to coach the SPs and encourage them to share their experience across the team.
  - This is a good time for the project team to check the audibility of the recordings and whether any settings need to be adjusted on the recorders (most audio recorders have an instruction manual and a quick test of the different settings will help determine which is most suitable for the environment of the health facilities).

4. Allocating audio recorders to the SPs and managing the recorders and recordings during fieldwork

- Depending on the budget for the project, all SPs may not have an audio recorder at all times. Suggestions on how to allocate the audio recorders are:
  - If the recordings will be used for rigorous analysis, then the audio recorders can be randomly allocated to the SPs during fieldwork.
  - If the recordings will be used for accountability checks, then the recorders can be purposively or randomly allocated to the SPs every so often during fieldwork.
- Management of audio recorders – Each night after the day's interactions have been completed, the audio recorder's memory must be checked, the files must be transferred, backed up, and named, and the audio recorders' batteries must be recharged.
- File management of recordings – After every day in the field, any SPs given an audio recorder must return the recordings while keeping track of which files on the audio recorder are linked to which interaction.

5. Data entry of exit interviews filled out from audio recorders

- Since the forms will be filled out for audio and recall (via the inclusion of questions on the cover page and/or section for supervision check), the data can be differentiated during data entry. It is ideal if the audio files and the recall files are separated for better data management.

6. Lessons from audio-recorded data vs. recall data

- Comparison of audio recorded and recall data can help answer the following questions:
  - Does the audio pick up items on the checklist that an SP does not recall?
    - If yes, this suggests that the SP needs to improve his recall of checklist items after the interaction.
    - If yes and the SP does not improve their recall (or if the audio substantially picks up more checklist items than recall), then the project team should consider letting him/her go.
  - Is the audio quality of the recording high enough to capture the checklist or are segments too noisy or missing (i.e., when portions of the audio recording are missing, it is impossible to fully capture whether all checklist items were completed)?
  - Is there strong agreement between the two measures?
    - To assess agreement between the two measures, the analyst can assess correlation coefficients between the SP exit questionnaire data and the exit questionnaire data filled in from audio recordings.
    - If there is strong agreement, then recall is just as good at measuring what happens during the interaction. In fact, recall may be better if (1) there are parts of the audio that are not interpretable or inaudible, and (2) aspects of the interaction cannot be picked up by audio record only (e.g., physical examinations such as auscultations).

5.7 *Staffing requirements*

The field team that works to accomplish an SP project usually includes: senior supervisors, junior supervisors, and SPs. It is favorable to setup and work with a field team that is designed to support interactions for a team of fewer than 20 SPs. In most situations and projects, there is no urgency to successfully complete a large number of interactions in a short amount of time. Thus, in a large project like the Qutub project, which resulted in over 8000 SP interactions

across two cities, it was better to operate with a team of three senior supervisors, seven junior supervisors, and 12–18 SPs, while going for as long as was needed to successfully complete all assigned interactions, rather than hire a 20+ SP team with correspondingly large supervision staff to get interactions done in a couple of weeks.

However, if researchers do find themselves working on such a project where the study benefits are greater with faster completion rates, it would be wise to mitigate the daily management challenges that can occur with having a large team of SPs interacting with providers in the field. With a large team of SPs (e.g., 20+), the number of supervision staff must also increase because the frequency and rate of SP exit interviews per day will increase, while accounting for the geographical spread of daily fieldwork. In the Qutub study, each SP was completing between 2 and 6 interactions per day (completion rates varied because of field challenges, primarily spending time locating health facilities without street names and finding closed health facilities when providers moved to different locations or closed temporarily for sickness or travel). Further, having a team of 20 SPs completing interactions at these rates can place a burden on not just the debriefing processes, but also the data entry and management processes.

General responsibilities, which can be used for drafting terms of references, and the number suggested for each staff type are:

- **Senior supervisors** – Generally, 1 senior supervisor for every 5 SPs and 2 junior supervisors. For example, 2-3 for a team of 10–14 SPs; 3-4 for a team of 15-20 SPs). Responsibilities include:
    - Developing the field plan to complete the interactions
    - Dividing the schedule and assigning individual schedules to staff
    - Identifying moments when refresher training is needed
    - Ensuring that SPs are presenting with the standardized case and are not straying from the script
    - Making sure treatment coding (see Section 11.2) and data entry and management processes are maintained (see Section 11)
    - Identifying key topics to communicate with the research team; addressing any concerns that would require input from the research team; incorporating fieldwork needs with the research team

- Meeting with the entire team of SPs on a regular basis to get direct feedback on their experiences, addressing field challenges, and supporting SPs so they stay motivated (as the SPs do tough work, ensuring that they feel well and stay motivated are important)
- Sending daily and weekly updates to the research team

- **Junior supervisors** – Generally, 1 junior supervisor to 2-3 SPs. For example, 3-4 for a team of 10-14 SPs; 5-7 for a team of 15-20 SPs. Responsibilities include:
  - Ensuring that SPs complete the patient-provider interaction specific components of the exit questionnaire within 1-2 hours of the interaction
  - Debriefing (i.e., capturing the interaction details on the exit forms) the SPs within 1-2 hours of the interaction
  - Supporting the SPs in conducting interactions at correctly sampled locations
  - Identifying any issues in the field or experienced by SPs and alerting senior supervisors of them
- **SPs** – Responsibilities include:
  - Conducting interactions
  - Scoping the field, such as checking addresses and facility operating hours
  - Debriefing with supervisors within 1-2 hours of the interaction
  - Reporting any issues that are occurring in provider interactions
  - Identifying any challenges given the environment
  - Alerting supervisors of issues in the field (e.g., events that may hinder data collection activities)
  - Keeping trained in the case and assuring exit strategies are refreshed in unexpected cases of risk

5.8 SP recruitment

This section contains the process for SP recruitment, which is critical for the success of many aspects of the project. (See Section 8 for further details on human resource management of SPs, such as factors influencing case allocation to SPs and the removal of non-suitable SPs.) Box 5.8.1 highlights the SP recruitment for the Qutub project. The process for SP recruitment and frequently asked questions for different steps of the process are:

1. Recruiting potential SPs
   a. *How long does recruitment take?*

i. The Qutub pilot project in Delhi spent 15-20 days on actual recruitment, after which 22 individuals were selected for the 3-week training. The 1-month pilot occurred with 17 SPs. At each of these stages, the set of SPs that is suitable for the study gets smaller. Section 7 describes this in further detail.

b. *What should happen before recruitment begins?*

i. Elements of the SP script(s) should be developed for the cases, such as age, build, educational level, and gender, which are characteristics that will identify the individuals who should be recruited.

ii. Understanding the localities that are represented in the SP cases, such as where the SP and the providers to be visited are from.

c. *What is the best way to recruit individuals who fit the role of an SP?*

i. Advertising the positions to individuals who are engaged or who have been engaged in survey or research activities is one of the previously used recruitment strategies. Existing networks with survey and research entities can prove to be very helpful, but it must be kept in mind that collecting quality of care data through SPs is not similar to other methods of data collection that hire enumerators or data collectors. One area of caution is that individuals already employed by market research firms to conduct surveys are often working on multiple surveys at a time or can be employed for one survey after another. This can improve their confidence in conducting surveys but can also result in a cavalier attitude that may not be fitting for the SP method, which requires an adaptable attitude and strong work ethic towards data quality and accuracy. Further, what differs is the responsibility for the SP to both act and memorize in a real scenario that requires strong improvisation. This is described later in this list.

ii. In other SP projects, study teams received recommendations for SP recruits through a snowballing effect. Some project teams contacted other survey organizations in the locale. Other study teams contracted survey firms who helped develop the SP case profiles and subsequently worked to identify individuals who fit those profiles.

d. *The disadvantages of hiring professional actors*

i. A strong description of the importance of recruiting SPs locally, rather than real actors is provided below from *Details Matter*, a piece on SP implementation by Das V et al. (n.d.) (19):

> *While actors might be able to enact the script in which they present the symptoms of a disease in a clinical setting, they cannot anticipate all the situations that might arise. Since health providers can share many of the cultural presuppositions of the patients from low-income areas, it is better to recruit SPs from communities being studied. A disease, after all, is more than the sum of clinical symptoms – a patient is not only a body on which different symptoms appear, he or she is a social-cultural being. Thus, the patient's experience of disease is mediated by his or her social setting, the attitudes of others, and the languages (including words, tones, gestures) that circulate in a given community. This requires an intuitive grasp over community norms that determine our sense of the "rightness" of ways of representing a disease in a particular local setting and being able to answer questions posed by practitioners about one's medical as well as social history.*

2. Interviewing SPs
    a. *How many SPs to interview?*
        i. An initial batch of 50 individuals was interviewed for KePSIE study, which targeted 25 SPs for training and 8-10 SPs for the final survey.
    b. *What to look for during selection and recruitment?*
        i. Criteria for selection and recruitment are largely influenced by the SP cases developed for the study. For that reason, level of education should reflect the SP case developed.
        ii. Individuals who are conscientious, good memorizers, improvisers, and possess self-control are often strong candidates for being an SP.
    c. *What is in the SP exit questionnaire?*
        i. Briefly described in the introduction, the exit questionnaire captures information that will become data for analysis. These have included: aspects about the provider or pharmacy (e.g., name and location) and the interaction (e.g., date and time, SP details), as well as information

about the SP-provider interaction. SPs will be required to memorize these during training. Section 6 and 7 discuss these in further detail.

d. *Group interviews are a good strategy!*
   i. Group interviews should begin with a vague, general introduction to the project as a data collection study. Total project information is usually not provided to the potential SPs until they begin training. Particularly, if the interviews are occurring in the study location, it is better to be incognito about the process until the training begins.
   ii. The project team can show videos of experiences in the clinic. Each participant in the group should then be asked to write down their observations after the video – for example, the recruits can be asked to describe whether they thought the provider in the video was good or bad.
   iii. Observing the videos without being told to be "naturally observant" provide an ability to capture how opinionated and detailed each individual describes situations and interactions. Since the writing exercise is conducted after the video is shown, this helps assess how individuals can hold onto the reality of the video without straying far during their recall. The video can be replayed several times (from experience, interviewees sometimes were not able to decipher that the same video was shown), and the writing exercise can be conducted again.
      1. The one-on-one interview below is a good time to ask the interviewee whether or not they can picture themselves being comfortable in the scenario portrayed in the video.
   iv. After the writing exercise, it is advised to hold a group discussion to debrief everyone's experiences in both watching the video and writing down observations.

e. *Conducting one-on-one interviews after the group interview helps identify important characteristics of a strong SP* - Throughout this interview, the project team would benefit from taking notes on communication characteristics for each individual. Examples of characteristics to note include: making eye contact, audibility, fluidity of verbal descriptions, speed of speech, confidence, tendency to exaggerate or boast, interest in participation and suitability to the role. To offer a standard measurement strategy, the project team can implement a Likert scale

(e.g., 1=not at all, 2=slightly, 3=considerably, 4=moderately, 5=extremely) to assess the subjective characteristics across participants. Additional aspects can influence but may not necessarily determine the suitability of an individual as an SP, beyond the ability to act. A few notable aspects to acknowledge during one-on-one interviews are:

i. Punctuality: Being considerate of time is important for conducting scheduled visits at clinics and ensuring communication with supervisors for proper and timely debriefing.
ii. Work experience in health settings or occupational background, as well as relevant experience in surveys: These are not necessary but those who have experience with health surveys may know how to endure the logistics and pace of fieldwork. At the same time, individuals who have a plethora of survey experience may have a set idea of what surveys should be, and that mold can prevent the ability of a potential SP to be flexible in the learning process required for SP fieldwork. This applies for work experience in health settings as well.
iii. Education levels: Finding individuals with education levels fitting the SP case and script is not necessary. At the same time, it is not required that SPs have high education.
iv. Strong feelings and/or opinions for or against the health system, health facilities, or providers: These may or may not interfere with the acting required by an SP. One way to attend to this aspect is to discuss with each potential SP any previous experiences with the health system, health facilities, or providers and how it made them feel. Among those with strong feelings or opinions towards the health system, the project team would benefit from internally discussing whether an individual is suitable on a case-by-case basis.
v. Comfort level in examinations at health facilities for the purpose of the project: In some settings, it is possible that SPs would be asked to provide urine, stool, or sputum samples.
vi. Availability throughout project schedule: It is possible for fieldwork to be six days a week. In these cases, the project team should ensure individuals' availability on Saturdays (if applicable) and the number of hours per day for the duration of the project.

Kwan A, *et al. BMJ Global Health* 2019; 4:e001669. doi: 10.1136/bmjgh-2019-001669

vii. Age and gender characteristics: Whether individuals are fitting or flexible in terms of the age range and gender depicted in the scripts and cases is critical for recruitment. Some projects of shorter duration have had difficulties recruiting individuals from older age groups for SP cases with an older profile.

viii. Good health and physical condition: Potential SPs must undergo a personal health assessment or one conducted by supervisors, for example. This can be captured in a separate exit questionnaire section where the potential SPs can list any family or personal history of health conditions, consumption of medications, etc. A project team should hire seemingly healthy individuals to portray SP cases so that a true health condition does not confound the interaction.

ix. Geographical areas where the SP lives, has worked, or spent extensive time: Noting this information is helpful to see if there is overlap with areas that will be sampled during pilot and/or fieldwork. There are some reasons why this is important. First, SPs may be familiar with the study location, and their knowledge of the locality, such as for transportation, can be beneficial for the entire team. Second, SPs with links to the study location may also know or be related to staff at the health facilities in the sample, and it would be wise to avoid scheduling those SPs for visits there.

x. Other: Any questions raised by the potential SP can be addressed at the end of the one-on-one interviews.

3. Finalizing the list of individuals who will be invited for training (initial SP list)
   a. Rejection phone calls should be done immediately for individuals who interviewed and will not be selected.
   b. Once a date for the Technical Advisory Group meeting is finalized, the study or field team should make confirmation phone calls to initial SP recruits. It must be clear to the selected SPs that (1) they will be paid per-day for training, and (2) it is not guaranteed that they will be selected for the final survey. They should then be asked to accept or reject the offer within a set amount of days.
   c. Scripts for the rejection and confirmation calls should be drafted and then used during the callbacks.
4. Conducting health screening among the SP recruits

a. A health screening should be conducted on each individual who has accepted the offer. An earlier health screening during recruitment may be necessary depend on the type of health scenario selected for the implementation of the SP method, such as for scenarios related to risk factors or non-communicable disease diagnosis. (See Annex H for the health-screening questionnaire). An excel worksheet should be created to capture at least the following individual characteristics: date of birth, age, gender, height, and weight (these data will be collected in the SP master or SP staff code file and can be included in analysis as control variables and to account for SP fixed effects).

Kwan A, *et al. BMJ Global Health* 2019; 4:e001669. doi: 10.1136/bmjgh-2019-001669

Box 5.8.1. QuTUB Project Spotlight: SP recruitment, script development and SP training.

Reprinted with permission from the Appendix (pp. 2-3) of Das et al. Use of standardized patients to assess quality of tuberculosis care: A pilot, cross-sectional study. Lancet Infectious Diseases, 2015.

A total of 17 SPs were recruited from an initial group of 22 who were extensively screened and trained for 3 weeks. These SPs included both those who had participated in previous studies and new recruits. The 17 SPs differed by age, sex, height and weight. The mean age of recruited SPs was 35; the youngest was 24 and the oldest was 51; 10 (59%) were male with weights ranging from 50 to 74 kilograms and heights from 160 to 173 centimeters. Female weights ranged from 40 to 72 kilograms and heights from 150 to 160 centimeters.

Scripts were developed under the guidance of an anthropologist (Veena Das) with active SP participation that described the social and family contexts of the patient. The two most important considerations for script development and SP training were: First, the clinical symptoms and case history had to reflect the social and cultural milieu of which the SP was assumed to be a member, and second, the presentation of symptoms and answers to history had to be consistent with biomedical facts about the disease. SPs brought a lot of socially appropriate understanding of the local vocabularies through which symptoms were to be presented and also about typical life histories that would correspond to the age, sex, caste, religion and class of the character that the SP was portraying. As a simple but crucial example, people among the strata the SPs were drawn from do not often use thermometers to measure temperature but report fever on the basis of the sensation of heat and rapid pulse. The inputs by SPs in script development were crucial from this perspective.

The second issue was to train SPs to present symptoms and answer questions pertaining to case history that were medically correct. For example, all opening statements and questions pertaining to the type of cough and its duration were standardized. A critical part of the training was to help SPs distinguish between questions to which answers could be improvised but had to be appropriate to the social role of the SP and answers that had to be given using local idioms but in a standardized format without any alterations. The dual aim of presenting the disease in a manner that was not misleading and avoiding detection were largely successful because the reasoning behind both objectives was carefully and repeatedly explained to the SPs and because of their active involvement in the script development and hands-on training. SP case scripts, checklists, and vignettes are available from the authors upon request.

All SPs underwent rigorous training for 150 hours that started with a focus on the cases and the development of scripts and proceeded to memorization and appropriate role-playing, as well as techniques to perfect recall of the questions asked and examinations completed during the interaction. Following the training, SPs visited doctors who were working with our team to provide feedback on their presentation and realistic depiction of the cases. Finally, dry runs were completed with unannounced visits to consented providers to help build the confidence of the SPs and take them through a number of "real-life" situations. Once protocols were in place for

5.9 Agreement with SP

Making agreements with SPs is very important to the success of training and fieldwork, as is being transparent with the SPs about what is expected of them. A confidential agreement template for SPs to sign before beginning training can be found in Annex I.

5.10 Provider Mapping and Recruitment

Provider mapping and recruitment is critical for determining a study sample, which becomes the backbone of fieldwork logistics. Certain information collected during the mapping stage may also be relevant for sample stratification. Notably, permission letters from sub-level Ministry of Health offices may be required for recruiting health providers into the provider sample. This should be obtained before recruiting facilities and can be planned with a map and a draft protocol on the strategy for recruitment and visits (including sequencing the order of facilities for SP visits). Box 5.10.1 provides the process checklist for provider mapping in the KePSIE project.

Several options for provider mapping include:

- Obtaining a master list from the Ministry of Health website, followed by a verification exercise to ensure that the provider universe at the study location perfectly matches the master list (e.g., in areas with high provider turnover, a master facility list that was last constructed several years ago may no longer be useful for SP work)
- Lane-by-lane mapping exercise with the mapping team to map the location, address, and other important information
- Other lists of providers can be procured through identified stakeholders, such as hospital systems, provider listing websites, or mapping lists conducted by other researchers or research institutions

Depending on the study, it may be advantageous to recruit and obtain provider consent during the mapping stage. However, if a waiver of provider consent is obtained, strategies that do not involve direct interview may be needed to capture mapping information.

The ideal list of information that is relevant to collect during the mapping stage includes:

- Facility Name
- Facility Location

- GPS Location (Longitude, Latitude)
- Facility Type (1=Public, 2=Private, 3=Social franchise, 4=Faith-based organization, 5=Other)
- Facility Level (e.g., 1=Level 1, 2=Level 2, 3=Level 3)
- Physical Address
- Landmark (*Nearest road, stage or building)*
- Nearest Market(s)
- Detailed instructions on how to reach the facility *(Reading these instructions to team members or assigning other members of the team to locate facilities based on these instructions can help guarantee the sufficient level of detail needed for the directions)*
- Phone number for main contact
- Number of staff members providing primary care *(Staff who actually see the patients for consultations, not the lab technicians)*
- Person who sees the most patients in the facility
    - Name
    - Qualification
    - Age
    - Sex
- Days and hours of operation *(This helps schedule when to send SPs to the facility)*
- Approximate number of patients on an average day
- Busiest day of week
- Approximate number of patients on busiest day
- Busiest time of day
- Approximate number of patients at busiest time
- Least busy day of week
- Approximate number of patients on least busy day
- Does the facility have its own… (1=Yes, 0=No)
    - Lab
    - Pharmacy
    - Imaging
- Is there a registration process for new patients? (1=Yes, 0=No)
- Supervisor Name (Individual who recruited the facility)
- Supervisor Notes
    - Is the facility on a main road or highway? (1=Yes, 0=No)
    - Is the facility easy to locate? (1=Yes, 0=No)

Box 5.10.1. KePSIE project checklist for provider mapping process.

The sequential checklist for provider mapping in the KePSIE project in Kenya included:

a Ministry of Health approvals
b Ethical approval from the non-governmental organization Amref Health Africa
c Country letter with District Ministry of Health stamp
d Consent form
e Facility recruitment form
f Tablets for GPS
g Confirm all providers have consented (consent was not needed for recruitment; however, it made maneuvering through the process easier)

5.11 Pharmacy Mapping and Recruitment

The process for mapping and recruiting pharmacists is fairly similar to provider mapping and recruitment. Often it is the case that these entities are more geographically established, possess lower rates of turnover, and are less organizationally complex than health care providers. From previous SP projects, these attributes have made mapping pharmacists easier than mapping providers. Additionally, for the same reasons, pharmacists have held knowledge in aspects of the health market and the relocation of providers that formerly practiced in the study location. This may be important for determining whether a master facility list is useful and for SP studies that involve waves of data collection, which require attention in reducing attrition rates through extensive tracking procedures.

SECTION 6. SP CASE DEVELOPMENT

This section contains information on how to develop the SP cases, which includes the SP script, exit questionnaire, and other aspects that define what the SPs will portray in the field. For each part in this section, it is critical to acknowledge how involving SP recruits right from the start of case development (with developing the story for the script) has the advantage that the SPs, once hired, do not have to depend on rote learning as the story is incorporated. Involving them at different stages beneficially establishes their ownership over the story as well.

6.1 Selecting the clinical presentation of cases

Regardless of whether the study is based on a particular condition or intervention, a variety of SP cases are available for review and use. Researchers may also want to investigate placebo effects or spillover effects from a program. (For example, the Qutub study in Mumbai and Patna assessed the effects of a tuberculosis program on quality of care and was interested in whether the program had spillover effects on other respiratory illnesses, so the team circulated SPs who were trained to portray asthma.) Researchers may find that questions, lingo, and contextual details from previously fielded cases may be applicable with varying degrees in the new context. The process of selecting, editing, and constructing clinical presentations, exit interviews, and scripts involves:

- **Identifying the appropriate health condition(s) to meet the objectives of the project.**
- **Reviewing conditions that have already been implemented in previous projects.** There is a growing body of SP scripts and exit questionnaires from previous projects, and the structures from these exit questionnaires are a good place to start (see Table 3.3.1 and Section 6.5). The standardized scripts and exit interview surveys that form the basis of the case presentation and data captures can also be adapted to new conditions, and preparing answers to likely provider questions can prevent against unexpected clinical investigations.
- **Conducting research on selected conditions.** This step involves fully understanding clinical and population factors that are related to the health conditions of interest. For example, the study will benefit from understanding the relevant disease burden in the setting of interest and the symptoms associated with the clinical presentation. Section 5.1 of this manual details ways to select appropriate conditions for SP projects, and the remainder of Section 6 will focus on developing the case before SP training occurs.

- **Investigating contextual field characteristics that affect the chosen cases.** For example, the child diarrhea (in absentia) case may be inappropriate or require adaptation in settings where treatment in absentia is outlawed, or adjustments may have to be made when it is socially atypical for females to travel alone outside the home or to visit (male) professional establishments. Some health conditions may naturally result in providers giving medication or injections on the spot, or ordering invasive examinations, such as X-rays or blood draws, which may put the SP in danger and result in ethical issues.
- **Identifying colloquial, vernacular, and local- and foreign-language expressions.** These may relate to parts of the body, physical symptoms, or clinical conditions. For example, diabetics may only discuss their "sugar problems" rather than mentioning "diabetes"; asthmatic patients may use phrases such as, "it felt like something was sitting on my chest" or "I was only able to take the top breath – the bottom breath was stopped" to describe an attack, among others. Anthropologists are instrumental in understanding how an illness is narrated. The particularities, cultural references, local framings, and indigenous understanding of diseases and symptoms will be necessary not only to create a believable SP script but also to prepare for unforeseen questions. During training, recruited SPs from local communities will be very helpful in identifying and correcting for these nuances. (Section 8.1 further details how local SPs can help resolve language and transportation challenges during fieldwork.).
  - Another somewhat related example of the need to account for vernacular is described in *Details Matter* by Das V et al. (n.d.) (19):

    > *Since the terms for confidentiality and secrecy overlap in Hindi, the vernacular language in which all discussions were held, it is imperative that candidates understand that observing confidentiality and being secretive are different. We explained confidentiality in the following way:*
    >
    > *"When we contacted the practitioners for this study we assured them that whatever is observed in their clinics or whatever information we collect from them is going to be used only for research. That is why we do not use their names when we record data – we just give them a number, and in any reports, the names of the practitioners are not used. Similarly, we will not use your names to protect your privacy unless you want us to use your name because you are proud of what you have achieved and want to be named. This is why we will ask you to keep all you learn in the course of this study about doctors or about each other to be completely confidential – not because we are doing anything wrong but*

because we believe in preserving people's privacy when they participate in research. The research will be to enhance public good, to help doctors to make better decisions."

- **Preparing contextually appropriate strategies for avoiding risk situations.** For example, in many settings it is common for health care providers to insist on giving an injection or having the patient take medication on the spot. Depending on the cultural context, it may be appropriate for the SP to refuse on the grounds of not having a family member present. SPs may also simply assert that they are fasting or have another religious limitation, that they are unable to afford spot treatment, or that they will return for the treatment.
- **Reviewing international and national guidelines for the conditions of interest.** This enables understanding of acceptable or prevalent treatments and diagnostics in study settings. This includes a variety of diagnostics and treatments, which have been banned or become unpopular. For example, in India there is a varying degree of trust in sputum acid-fast bacilli microscopy; on the other hand, formally banned diagnostics for settings with high incidence of latent tuberculosis, such as the Mantoux test, may still be ordered. For treatment, in cases of asthma, some settings prefer corticosteroid treatment; others prefer non-steroidal bronchodilators (either inhaled or ingested). In some cases, such as upper respiratory conditions in which an immediate chest X-ray is both an appropriate diagnostic practice and an unacceptable risk exposure for SPs, this review may lead to further refinement of scripted responses and strategies.

6.2 Conceiving the SP cases: script and exit interview development

An SP is trained to portray a case, as determined by the project purpose and objectives. Each case contains a script, and each script is linked with a corresponding exit interview. Further, a case may also include medical artifacts, such as a diagnostic test report or chest X-ray, which would be carried into an interaction by any SP portraying that case. Annexes J and K include a sample script and corresponding exit interview for one of the SP tuberculosis cases implemented in the Qutub project pilot; Annex L includes the follow-up detection survey with the corresponding vignette for the same study (5).

The process for developing the case with script and exit questionnaire begins during the conception of the project and culminates during SP training. (For more on SP training, see

Section 7 and Annex M for 3-week training schedule.) Again, involving the SPs throughout this process will be very beneficial to the success of the project.

Along with clinical characteristics, each SP case presentation requires certain characteristics, such as specific personality traits, emotional intelligence, occupational history, and a life history, to all be developed so that the SP case is humanlike. Individual characteristics of each case should be developed to include the following relevant backstory elements:

1. Day of interaction
    a. Where the SP is coming from – Was the SP coming from work or home (geographical reference) and was the SP alone or with family or friends (social element)?
    b. Where the SP is going – Where is the SP going after the visit, including the mode of transportation?
    c. What triggered the event – What occurred on this day that caused so much worry the patient felt a visit to the health facility was necessary?
    d. Why visit this facility – Why was it convenient to visit this particular health facility on this day, especially where it is common for patients to visit facilities where they are already familiar with the provider?
    e. Other – Why is the SP visiting the facility without a trusted family or friend escort, especially for female patients or older patients? Why does the SP not have identity documents or a working mobile phone on hand?
2. Current life situation
    a. Socioeconomic characteristics, including appropriate dress (such as loose-fitting clothing in cases where weight loss is symptomatic), approximate age, place of birth, extended family/parental place of residence.
    b. Family life, including relationship with spouse (if any), parents, relatives, the number and age of children, occupation, religion/class/caste where relevant, smoking and drinking habits, vegetarianism or other dietary restrictions where relevant.
3. Past life situation
    a. What brought the individual to this region – Did they move from another part of the country and, if so, how recently?
    b. Events that are related to the condition they are presenting with at the health facility that day – Are there concerns regarding the spouse/family, an acute episode that morning

or the previous night, the recommendation of a trusted friend or colleague/coworker, or a public health advertising campaign, if applicable?

During the SP training sessions, the project team with the input of the SP candidates will do activities that help build further characteristics of the SP case with these aspects of the character. This is described later, but involves thinking through what this case would wear, if the individual is naturally confident or not (this may vary by gender, caste/tribe/etc., class), and other essential identity elements.

The case and script development process occurs at various stages of the project as detailed below:

- **Recruitment of facilities and field manager facility visits.** Expert staff and field management teams should scope selected or potentially selected facilities. These individuals should conduct on-site patient observations to understand how patients enter the facility, how large the waiting queue is and what the typical waiting time and registration procedure are. SP field staff may also inquire as to what times are particularly busy or if certain types of patients typically visit the facility (for example, some facilities become de facto pediatric facilities or have other informal specializations). The research team will want to research the environment or social background from which patients would come, such as the typical class, caste, or domestic living situation. Detailed field notes should be kept from these observations. When provider information is being collected, it is also forward thinking to record any note with a linking ID so that provider and facility data can be linked to provider universe datasets, master code files, or future SP data. Including local supervisors who are expected to manage but not actively participate in SP fieldwork is useful to maintain organized information.
- **SP case script drafting.** As part of SP case development, the research team and supervisors must work together to develop a first draft of the SP scripts, incorporating key ethnographic details from the field visits. They should conduct initial script development meetings individually with Technical Advisory Group members to help refine the cases. This will incorporate further ethnographic information gathered from the field or from the phase where clinical aspects of the SP case were developed. For example, field visits can help note the colloquial expressions for symptoms (e.g., for asthma, in India, the phrase, "I produce a whistling sound"; or in Kenya, "I make a sound like a cat").

- **Meetings with Technical Advisory Group members.** Meeting with individuals from the Technical Advisory Group to continue to develop and refine the clinical details and individual characteristics for each script is helpful. The expert panel invariably provides additional insight on the health care providers' perspectives, diagnostic and treatment preferences in response to draft SP scripts and mock case presentations, including likely variance in those practices. They will also be able to offer further suggestions about cultural elements regarding providers' likely dispositions and appropriate reactions to various situations, as well as the patients' expected degree of interactivity, submissiveness, or respectful honorifics to be used by the patients. This may be especially useful if SPs have relocated from outside areas for the job and/or are unfamiliar with the behaviors of another social class, indigenous, tribal or caste group, formal setting, or religious group with whom they will be expected to interact.
- **Meetings with entire Technical Advisory Group**. The whole group should review and agree to the set cases, scripts, and presentations by consensus once the implementing team has finalized them.
- **First week of SP training.** The SPs themselves are indispensable to the process of finalizing the cases, scripts, and questionnaires. During the first week of training, comments from the SP trainees should be actively solicited, and some substantive suggestions should be incorporated immediately to convey the seriousness and trust with which the researcher should regard well-chosen SPs and the upcoming fieldwork. This demonstrates that the SPs' input has real actionable consequences for both themselves and the project, including for their personal safety and protection. Incorporation of further input from SP trainees on cultural factors relevant to their own personal experiences can be the output of active discussion of their personal and family interactions with health care providers and facilities in the past. Afterwards, if the SP work of interest requires language translation or localization of cases, including the scripts and the questionnaire, this can be done with the SP candidates in what is referred to as "mock interviews", which reflect the complex and nonlinear interactions as idiomatic differences from the case drafting language or dialect in such a way that may render certain questions or response options indeterminate or nonsensical if translated literally. In addition, SPs should be able to help in questionnaire design. In particular, SPs can help identify implicit logical connections that are not apparent either in context or in translation. For example (in a non-SP study), the question, "How did the event affect the child's school enrollment?" may have a response option "Never

enrolled" which is intended to indicate, "Because of the event, the child never enrolled." However, it may also elicit the implied response, "The child was never enrolled, so the event did not impact his enrollment" depending on the exact translation and even tonal changes in the enumerator or supervisor's presentation of the question. Similarly, a crucial SP question that begins in English as "Total Price" may be translated as, "What did you have to pay", leading to indeterminacy between "What did you pay out of pocket [on the spot]" and "What was the total amount this would have cost you, inclusive of all listed [but not necessarily incurred on the spot] expenses?"

- **End of first week of training.** During the first week of a 3-week training period (see Section 7 for thorough discussion on SP training), the project team should finalize and freeze the scripts and questionnaires so that SP trainees can begin memorization during week 2 of training. Data staff can then use this time to process pre-fieldwork tasks, such as questionnaire digitization, preparation of quality/consistency checking and daily reporting programs in statistical software, materials for data entry teams, and merging protocols with other data sources. SP candidates should also have individual biometric and health data taken, recorded, and coded on a confidential master staff roster. SPs should be assigned unique IDs and these should be included on the exit questionnaires. Additional information on the SPs should be collected in what is referred to as the SP staff master code file, discussed in Section 11.1.

6.3 Developing medical artifacts for SPs to carry during the interaction

Some cases that are designed to assess how a provider reacts to diagnostic test results will require SPs to bring genuine medical or other artifacts to the interaction. For example, the Qutub project team developed a TB case where the SP carried a chest X-ray and accompanying diagnostic report. The chest X-ray and report were given to the provider when prompted. Upon turning over the chest X-ray and report to the provider, the SP was trained to inform the provider where the X-ray had been conducted. Depending on the extent and characteristics of the study location, such as whether the area has a prevailing public or private sector, this case or similar cases that involve a medical artifact can have the SP explain how the test was done nearby or at a private or government facility. In the same study, the team developed another TB case in which SPs carried a governmental lab report positive with TB. In the cases with the chest X-ray and the positive sputum report, the materials were incorporated after being determined relevant for understanding quality of care for different

stages along TB disease progression. There are several challenges to this process in terms of feasibility, which should be assessed by the project team.

When piloting the chest X-ray, it was challenging for the Qutub team to reproduce the X-rays. There was no way to take a reproducible digital copy and feed it into an X-ray printing machine, and many of the machines did not have such a feature. Other issues related to incorporating an X-ray into the case included:

- Purchasing an X-ray machine was not only expensive, but nobody was ready to consider whether the production could be done, for fear that the machine would break.
- One person tried to put X-ray files on a pen drive, but this was not successful.
- A printing facility provided a print, but the quality was poor. It was done on a sheet used for projectors, which was thinner than the average chest X-ray film, and the outcome was smaller than the normal X-ray negative, rendering the print to look inauthentic.
- Dates and lab names were included in actual test reports, including the signature of a qualified laboratory technician. This introduced complications, such as obtaining appropriate signatures from qualified professionals. Reports also included the name and gender of the patient, and thus, these were to match the SP's actual gender and scripted name.
- Physical exams such as the X-ray must believably match the observable physical characteristics of the SP. Some X-rays may be inappropriate for patients of different genders or statures, which in a typical cohort may vary by up to 20cm within each gender.

As a solution, the study team worked with a member of the Technical Advisory Group to establish a Memorandum of Understanding with a physician with an in-house laboratory to produce chest X-rays specifically for fieldwork. Since chest X-rays were to be dated (e.g., two weeks before the SP interaction) and to match each SP's general physical features and gender, chest X-rays were ordered by the field team.

6.4 Allocating the cases to SPs

Prior SP studies have not shown significant systematic impacts of major SP characteristics such as gender on the outcomes of provider interactions. However, naturally varying characteristics such as height, weight, blood pressure, and other physical attributes have shown mild impacts

on provider treatment choices. In general, it is usually not possible to reject the hypothesis of no idiosyncratic SP effect on the interaction using a simple statistical test on the joint effect of a set of SP fixed effects on interaction outcomes. This may be attributable to any combination of unclassified or immeasurable factors such as skill, physical attractiveness, outgoingness, confidence, effort, or other physical, non-cognitive, or socio-emotional markers of identity and interpersonal interaction.

As a result, SP candidates should be assigned in an as-good-as-random fashion to case presentations and interactions. In practice, this may mean leaving case assignments and within-case scheduling decisions to supervisors of field staff with the guidance that "more competent/confident" candidates should be spread evenly among the cases (on the rationale that all case presentations are equally important) and therefore not systematically correlated with the perceived complexity of any script or case. Allowing the managerial staff to assign individuals within each case based on field needs, such as temporal or geographical proximity, not only gives the managerial staff a heightened degree of autonomy and buy-in to the implementation, but it also reasonably approximates random assignment unless there is a strong reason to suspect this will induce an unacceptable degree of correlation between the physical characteristics of SPs and the characteristics of the providers they visit. For example, managers should receive guidance allowing them to send an SP to geographically proximate locations on a given day at their discretion to maximize work efficiency, and should also be instructed not to assign individual SPs to cover distinct geographic areas on an ongoing basis.

6.5 Designing the SP exit questionnaire

As the script has been previously discussed, this section will discuss aspects of the exit questionnaire. As mentioned, examples of an SP script and exit questionnaire are included in Annexes J and K, respectively (Annex L contains a corresponding detection survey and vignette). Before reading this section, it is worthwhile to skim through these annexes to observe the sections of the exit questionnaire and how they link to aspects found in the script (and vignette, if relevant).

Exit questionnaire sections are typically ordered to generally mimic the flow of the actual clinical interaction with questions that are more pertinent to accurate recall (e.g., history questions) sequenced ahead of other questions. The exit questionnaire in Annex K contains the following sections:

- Cover page
- Essential history information and recommended information taken by the provider
- Clinical examinations conducted by the provider, recommended investigations ordered by the provider
- Diagnosis details
- Medicines prescribed or diagnosed and treatment details
- Referral details
- Global assessment scale
- Errors and detection information
- Supervision check
- Comments

Sections need not be explicitly numbered, as this often leads to more trouble than it is worth. In practice, sections are often rearranged or replicated across different SP cases or different rounds of fieldwork, while deletions or additions are made. Exit questionnaires should be designed with field team user experience, data reproducibility, and analytic interconnectivity in mind, so that SP results within the same clinical condition and across various conditions and locations can be compared effectively with the objective of minimizing the time and costs attributed in the analysis phase. Each section should have an identifiable thematic unifier and short code with correspondingly numbered questions (e.g., an exit questionnaire that ascertains whether a doctor asked 11 history questions can assign history questions 1 through 11 as H1 through H11, with corresponding data codes `h_1` to `h_11`). This practice allows the questions to be quickly matched to other surveys that ask similar questions during data processing. As few questions as possible should be open-ended. Some typical elements are described next.

All questionnaires should begin with a "cover page" listing basic interaction details, even if this appears redundant at first glance. Items such as "facility name", "facility code", "provider name", "provider code", and "address" are essential, even if they ostensibly link to the master sampling list. Even if there were a well-verified master universe list of facilities and providers from which these characteristics are drawn prior to sampling and scheduling, it is inevitable that changes will happen in the field. Staffers may be the only trusted associates who ever visit a specific location in-person during a large study. Detailed address records will not only help staff relocate the facility on subsequent visits, but may also contribute to geocoding in later analysis. GPS

coordinates can also be collected at the time of field visits depending on field constraints, such as the likelihood of detection or suspicion by the provider.

The cover page should also have space for a fully unique "form number" field, which field staff should be able to catalogue and use to refer to original survey forms when questions inevitably arise in data analysis or quality checks. Form numbering allows unique cataloging of any interaction at the original source, across the various data systems and structures that may arise, without reference to codebooks, combinations of uniquely or personally identifying variables (such as facility ID + provider ID + SP case code + visit number + baseline/midline/endline code), or other more complex systems of nomenclature, which would be constructed piecewise at the analysis phase. This must be rigorously checked for uniqueness at every data import. The field and data entry staff must resolve any and all duplicates or mismatches from the fieldwork-tracking sheet (i.e., a file that dynamically tracks the schedule that provides the field team with information on each interaction, see Section 11 and Annex Q).

The Qutub questionnaire structure, for example, involves the development of a single "common" questionnaire base, with most components standardized across all questionnaires and cases only vary within the key "interaction details" and "history questions" sections, as well as the possibility of the addition (or removal) of one or two case-specific questions regarding diagnosis or treatment. For all questionnaires, the cover page, intervention information, location tracking, laboratory tests, pricing, diagnosis and referral, medication, treatment, assessment, errors, supervision, and commentary sections are all fully standardized across all cases.

In practice, laying out this type of questionnaire is best accomplished in a spreadsheet program, such as in Microsoft Excel. Once column widths, margins, headers, and footers are standardized to fill the letter size of the printed sheet, the spreadsheet program's lack of pagination means that items can be developed freely and formatting handled as a final consideration, whereas in Word, tabular formatting may be broken by pagination and auto-formatting as a primary consideration over content. Although this places some constraints on the questionnaire developer (e.g., it becomes difficult, for example, to interleave portrait- and landscape-oriented pages), creative solutions can be found to almost any formatting problem and, in the Qutub project experience, have universally led to more streamlined questionnaires than the open-

Kwan A, *et al. BMJ Global Health* 2019; 4:e001669. doi: 10.1136/bmjgh-2019-001669

ended design of Word would have led to. The row-based design of Excel also enables effortless relocation of survey sections and the replication of sections from one survey to another, as before, ignoring formatting concerns until final field preparation.

6.6 Designing vignettes

An example vignette that can complement an SP study or that can function as stand-alone to assess provider knowledge is included inside the detection survey in Annex L (see Part 3 of the detection survey).

As described in Section 1, vignettes are surveys administered to providers. Vignettes can be designed to provide details of what the doctor knows and what she would do when she is presented with a case that is described in the survey. Administered before or after implementing the SP method, the vignette can provide powerful data with the SP data, allowing for an assessment of the gap between what doctors know (i.e., their competence measured through the vignette) and what they practice (i.e., their effort levels measured through the SP data). This gap has been referred to as the "know-do" gap and is another dimension of quality of health care.

In order to design such an analysis, scenarios need to be implanted in the vignette, and the scenarios require coordination with the SP cases. By replicating the SP case in the vignette – from the opening line to the question responses – the difference between provider knowledge and practice of proper case history, diagnosis, and treatment can be discerned by comparing vignette performance against the appropriate SP case results at the data analysis stage. However, the vignette needs to be carefully designed (so as not to disrupt the comparability) and implemented with enough time gap from the SP interaction that it does not have a spillover effect to or from practice.

The vignette in Annex L contains the following sections:

- Cover page
- History
- Relevant examinations provider mentions conducting
- Relevant examinations provider mentions ordering
- Medicines provider would prescribe or dispense
- Observation notes by enumerator

Vignettes have the potential advantage of being able to offer the provider additional information in the hypothetical case scenario at low cost. For example, the vignette enumerator may be able to give the "results" of the tests that the provider orders immediately (and before asking for diagnosis or treatment decisions). If the vignette is to be used for comparison with SPs, however, this is an inappropriate design, as SPs will typically not complete these during the interaction with the provider. The providers should be allowed to ask any history questions and conduct any physical examinations they like, given the responses the SP would give.

Similarly, providers should be able to order any laboratory investigations they like. However, it may be preferable to not provide the results of laboratory investigations before asking the provider to make a diagnosis and/or order treatment. Vignettes should similarly decline to reveal the true underlying diagnosis before asking for treatment decisions, as this will inevitably lead to very high rates of correct treatment with low correlation to other key predictive factors and extreme dissimilarity to SP results for an otherwise identical case. An alternative vignette design could, for example, ask for a treatment decision on the spot, and in a second stage report that the hypothetical vignette patient returns with the results of the ordered laboratory tests.

Unlike SP cases, however, vignettes typically require consent from providers to participate in the study (whereas unannounced SP cases can be covered by consent waivers with the support of public health officials) and express coordination with the providers in the study to minimize attrition and non-response bias. These practical and analytical obstacles, while not necessarily disqualifying, make clear that vignette implementation is appropriate to a slightly different research objective and study population than SPs, which should be carefully considered and piloted before going to field.

6.7 Designing a detection survey

A detection survey assesses the rate of SP detection and is one way to validate the SP method. It can be administered during the post-interaction stage of the study, along with a vignette (see Annex L).

Data generated from the detection survey are used to assess the detection rate of SPs among the providers visited, and the detection rate is calculated by dividing the number of SPs detected

by the providers (true positives) by the total number of SP interactions conducted. As a rule of thumb, SP studies have reported detection rates around 5%. Although the detection survey and provider vignette are best implemented after SP interactions are completed, planning for these are a part of the pre-interaction process. Below are details on how to implement a detection survey.

The detection survey provided in Annex L was administered by study supervisors for the Qutub pilot project and contains the following sections:

- Recognition and identification of SP
- Basic knowledge of the diseases covered in the SP cases
- Vignettes
- Facility characteristics

A strong detection survey has two phases and can benefit from the requirement of obtaining provider informed consent. This is because introducing the study to providers before they receive SPs can allow for a moment to get them to help with correctly identifying the detection rate. However, since it will be challenging to do this in a large-scale study or an unannounced study except as a subset of the provider sample, this should be considered carefully during study design. Research teams are encouraged to devise an appropriate variation to the two phases below.

- *Phase 1.* A month before the planned SP interactions, the data collection team visits all the providers, and after obtaining consent, providers and clinic staff are informed that some time, for example, in the next 3 months (3 months is sufficient for a 1-month study – it is beneficial to adapt this time window to be larger than when the interactions are planned as to not to prime the provider) the clinic will receive a fake patient (if the clinic is to receive more than one fake patient, the project team should adapt the number of fake patients to the study). If the provider or any clinic staff believe that a patient is fake, the provider should be instructed to continue with the consultation but should also be asked to write down the patient's name, age range, gender, date of visit, presenting conditions, clothing, and any other identifying traits. The data collection team should inform the clinic that after 3 months, the team would return to collect this information to assess if the fake patients were detected.
- *Phase 2.* After the time window has passed and after all interactions have been completed, the data collection team should return to collect the information. All patients

identified as fake by the clinic should be matched with the SP interactions to see how many were true positives and how many were false positives.

- It is also possible to conduct blinded, random phone calls with consenting providers to check whether they believed any patients they received in some past time frame were fake. One word of caution with this approach is that phone calls may remind providers that the study is occurring, which in turn may cause providers to be more vigilant and curious to detect SPs.

Critical things to consider:

- A detection survey must differentiate whether a provider believed *at the moment of the patient's visit* if an individual was a fake patient vs. *after the visit.* Further, understanding why a provider believed an individual was a fake patient during or after the visit is not only helpful for future implementation, but is also an important data element to report when describing the study's detection rate.
- A detection survey is difficult to implement in rural settings. If asked in an interview, a rural provider may quickly think of the patients he or she received in the past couple of months and immediately "detect" the individual who came from out of town.

SECTION 7. SP TRAINING

7.1 Schedule for training

The training schedule for SPs described here will be explained for a group of 20 SP trainees. In this manual, the term "potential SPs" will be synonymous with SP trainees and trainees; "trainers" refer to the individuals who are conducting the training, whether that be members of the project team or a contracted firm or both. Experience in training SPs in India and Kenya has suggested that three weeks is adequate for an initial number of 20 trainees to memorize and internalize the scripts, memorize the exit interviews, increasingly contextualize their characters and the characteristics of the condition, do mock-up interviews, and conduct dry runs. With a three-week training schedule, each week has specific objectives that increasingly educate and train potential SPs for the study:

- **Week 1.** To refine the script narratives, to introduce the structure of the script and exit questionnaire
  - This is done through script and narrative development with group reenactments and introduction of exit questionnaires
- **Week 2.** To practice the scripts, to internalize the identity portrayed in the scripts, and to practice recall and improvisation
  - This is done through script internalization and mock interviews with recall and improvisation skills building
- **Week 3.** To practice as the SP cases in real settings
  - This is done through full mock interviews in the classroom and dry runs in the field

Annex M contains a template for a 3-week SP training schedule in full detail. The other parts of this section will describe training logistics, training activities, and more. Box 7.1.1 summarizes nine tips and strategies for training SPs.

There are two important aspects to keep in mind when training SPs.

First, as mentioned briefly in other parts of this manual, trainers should be aware that the SPs have experience in their local world and that is what they should be building upon. Since the SPs are most often recruited from the local milieu, they will have some idea of the experience

of the illness, the difficulty in accessing treatments, and notions of affordability. The SP should be made aware that if questions are asked for which answers are not prepared then the SP can give vague answers so as to not corrupt the data. For example, if the provider asks, "Have you taken any medicines?" and the script doesn't mention it, the SP should say, "No, no I haven't taken anything." However, if the provider asks an open-ended question, the SP should respond by repeating any information that has been revealed up to that point, taking care not to reveal any new information on the script or any information that goes off script. In the experience implementing the tuberculosis SP case in urban India, the provider was likely to ask why the SP did not seek any help for so long. The SPs then would provide a fitting and vague response such as, "I haven't had the time."

Second, memory retention from training lasts for 4 months. If SPs are trained and do not conduct data collection after 4 months, there is a need to retrain with similar intensity. This is a very general rule of thumb, and the project team should exercise their judgment in determining whether or not the team of SPs should be re-united for either a brief or full training session. The use of audio recorders to verify aspects of the true interaction can provide an idea of which SPs and what parts of the cases can benefit from retraining.

BOX 7.1.1. Strategies and tips to train SPs

1. **After SPs have been selected on the various criteria such as appearance and fit to portray certain socio-economic status, education level, age, language, disease and health conditions that are being studied, efforts must be made to make the SPs understand the research question and methodology.** At the outset, SPs must be made aware that the SP methodology and the research project is not a "sting operation" or a journalistic uncovering of medical practices in the city or country. The research must be explained using language that is familiar to SPs. For example, they can be told that, "Similar to how thermometers measure how high our temperature is we are using this method to measure how to improve medical services for patients and clients seeking care." If possible, SPs should be shown X-rays or reports of diagnostic tests so that they understand the research question more clearly, and it becomes apparent to them why certain symptoms may be so similar that would make differentiating between two or more health conditions challenging. The goal of the health care provider within an encounter with a patient should be to work through the process of differential diagnosis, obtaining an idea of

which health condition is of concern for the patient. To that regard, the SP case – both clinically and socially – must be presented in such a way that it is lifelike.

2. **SPs must be made to understand what standardization means, and this is usually achieved by giving analogies from their local world.** For example, SPs can be told that just as weights for measuring food are required to be the same everywhere, or each car from a company is required to be the same, or the size of shoes needs to be the same and size 9 cannot be big at one shop and small at another shop, similarly healthcare should also be the same and ideally for population health at a high level of quality. This will enable the SPs to understand that they cannot describe their symptoms differently or give more information than what they have been trained to give. This was very crucial for the Qutub project, since many doctors asked whether the SP had taken any medicine (the script stated they had not taken any medicine). Since cough syrups are well known by their name, they had to remember that they could not say that they had taken "Grilinctus syrup" or "Vicks." Indeed, this would be a crucial criterion for eliminating candidates, if they are found to deviate from their script. SPs must be taught to stay calm and say they did not take any medicine (if it is not in the script) and to make excuses such as, "No, I have not had the time to go to the shop to get the medicine" or "I wanted to go buy but the chemist said please go to the doctor first". The issue of standardization once explained will make it clear to them that they all have to present the cases similarly with the same information.

3. **Once the medical script regarding the presentation of the disease is clear, SPs should be engaged in helping to develop the narrative of the case.** They should be asked what the appropriate answers to the questions should be, considering their social world. For example, if the medical script requires symptoms of severe headache of 1 day, then SPs should be asked how they would answer the question, "Why did you not go to the hospital or visit a doctor earlier?" When asking SPs what the appropriate answer would be, answers may range from, "I thought it would pass away", "There was nobody at home to look after the children", or "I could not get time away from work". SPs will be crucial in developing a standardized social narrative that fleshes out the character of the patient and fills details such as children, occupation, place of residence, etc. Thus, while the clinical narrative of the SP is fixed, the social narrative can only be standardized to a certain extent. Since doctors take a wide variety of evidence and clues into consideration while testing or diagnosing a patient, it would be impossible for researchers to anticipate all such evidence. After the medical parts of the script have been standardized, the social

presentation of the SP should also be standardized to ensure no artifacts enter the data. For example, if the study is being designed to measure the utilization of free government services, then the SP must be given some anchor points in the narrative that reflect her need for such services. The SP could be trained to say that she is worried about the money for such services since they are often expensive in the private sector and she has to send money home, or her expenses at home make it impossible for her to afford it. Such narrative scaffolding not only ensures standardization but also guides, directs, and controls the SPs as to what information they can give and how that information must sit well, gel, and not contradict the clinical script. If the SP is not given any narrative then they might give information that might not raise suspicion but bias the doctor. For example, if an SP has to present a headache, when asked what he was doing the night before, he cannot be allowed to say he was out drinking and dancing at a night club. Or, if the SP is presenting breathlessness to measure the quality of care for asthma, then he cannot be allowed to say that he is also a heavy smoker or enjoys cigars.

4. **The standardized script with the fixed answers to the medical questions related to the condition being studied should be given to the SPs to memorize.** SPs can be tested on the questions that require a fixed answer which may range from, "Have you taken any medicines?" to "Are you diabetic?" to "Do you have high blood pressure?", and so on. The SPs' recall will also test whether they are suitable for the study. The social narrative too must have some standardized answers if necessary such as name, occupation, history of the symptoms, etc. Since a wide range of questions can be asked, SPs must be trained on how they can answer these questions in a manner that will not arouse suspicion and will not de-standardize the data. SPs must be trained to give answers to unanticipated questions in a way that will sit well and are congruent with the narrative. For example, if the doctor asks an unrelated question such as if the patient has had a hysterectomy, then the patient cannot answer, "I don't know" but must answer truthfully if they have or have not actually undergone it.

5. **SPs must be prepared for unanticipated questions.** First, SPs should be asked to scout the place of the clinic for a fair amount of time. They can then answer questions like, "Where do you live?" or "Who sent you here?" with answers like "I came here to visit my family member" or "They live near the crossing next to [NAME OF SHOP]". Since many questions that providers ask are dependent on their specialization, if the list of providers for a study is large and contains a variety of specializations then the SPs must be aware that they may be asked a wide

variety of (potentially) unrelated questions. If an SP trained for tuberculosis visits a psychiatrist, she may be asked about her family life to which she must give answers which are in congruence with the clinical script and the SP narrative. This skill is achieved in training by presenting scenarios that enable them to imagine the other person and then eventually their SP character. For example, they might be asked "A is a shopkeeper. What will A do if his neighbors are creating a lot of noise?" SPs amongst themselves will discuss and can come up with a lot of answers such as, "He might go and start a fight" or "Since he is a shopkeeper, he won't risk creating trouble and will try to appeal to them calmly". Finally, supervisors and researchers must keep a close track on large developments in the geographical area of the study. During the Qutub study, earthquakes, local elections, strikes, protests, riots, and bomb blasts hit the research sites. These developments affect the questions providers ask their patients and some of them are important enough to affect the quality of care. For example, after a local election in which a certain caste/tribe has won, clinics and providers might ask "Which caste/tribe do you belong to?" The rationale for asking this in one Qutub study field site was that they did not wish to anger the winning party's caste member by making them wait too long or not treating them properly and risk getting their clinic vandalized. Since the Qutub study was also measuring waiting times, it was necessary to come up with a neutral caste name so as to not affect the study. Another example would be if the provider asked what the SP had for his last meal. If this question is not relevant then the SP should give a standardized answer that would not rouse suspicions or affect the data. The SP should not say they had spicy food or ate outside in a restaurant or food that is not commonly available.

6. **SPs should be given copies of the exit questionnaire so that they can familiarize themselves with the questions that they will be asked after the visit.** This is important because it will direct the attention of SPs to routine actions that are not usually noticed or observed carefully. This will also make the SP aware that the doctor may ask questions in a different manner. For example, the SP should be aware that the question, "How long have you been coughing?" and "Did you have this kind of cough before?" are variations of the same question. By heightening their awareness of small details, such as the use of thermometer, the presence of television, the presence of other people or the absence of privacy, the SP will be able to offer critical insights into the practice of the provider. Furthermore, the SP will also become aware of self-perceived judgements of quality of care – did they like the doctor or not – and also what the reasons were for their opinion. This is an important indicator of how providers are responding to the market or the population

to deliver care. A provider might not be following protocol correctly but might still be very popular with the patients. The SP's judgment of the quality of care will reveal to us the things that are considered important in practice by providers and patients alike yet may never reach the medical textbooks.

A tendency amongst SPs that must be resolved will be to immediately offer all the symptoms they have been trained to present. During their training, SPs must be corrected if they offer evidence without the provider asking. Since SPs must remember not only their symptoms but also when they can affirm or deny having those symptoms, they must be aware that some questions regarding their symptoms were asked or not asked. For example, if presenting a case of dysentery, the SP should not immediately say that he noticed blood in the stool, but should reply in the affirmative only when asked and furthermore distinguish during recall whether the provider asked the question or not.

7. **Strategies to avoid immediate medication, testing, and other medical procedures have already been mentioned and must be adapted to the local settings.** SPs will be the best source of information on which strategies are believable and can be used without arousing suspicion. While detection of the SP is a possibility, they have also been rare in the studies we have conducted, cited, and studied. The SP must be made aware that if a visit is not running according to plan, and the SP is getting anxious that the doctor is suspecting they are a fake patient then they should not immediately jump to the conclusion that they have been detected. There are many reasons why the doctor might suspect that the patient is giving false information, and the fact that they are an SP will probably not be the immediate reason. For example, in a highly competitive private market, a doctor might suspect that another doctor has sent the SP to see what kind of tests and medicines the doctor provides. It could also be possible the doctor suspects the patient is giving false information about his residence and history because of stigma and might actually want to alleviate those concerns by probing further. Doctors might think they are being checked on by government agencies on whether they are following the laws. In short, the SP when getting anxious about discovery should not immediately give in to confess that they are an SP but be aware that there are multiple reasons why the doctor might become suspicious. They can then deploy several strategies to exit the clinic. Some exit strategies include: saying that a relative is waiting outside, pretending they are receiving a phone call which they must answer, or saying they must withdraw money for the fees. The supervisor of the SP should also arrange to call the SP after a set amount of time to inquire if everything is ok – this will also

enable the SP to leave the clinic by pretending there is an emergency without revealing that he or she is an SP.

8. **SPs should not conduct interactions when they themselves are sick or unwell for several reasons.** First, any current sickness experienced by the SP may distract the provider away from the actual case being presented. In likely unobservable ways, the quality of care data from those interactions would be confounded by the actual sickness. Second, since the SPs are to spend time in the waiting room surrounded by other patients or at the clinic where others are sick, it is better for the health of the SP to not be put in a situation where they can become sicker.

7.2 Logistics for training

Accessories suggested for training SPs include:

- Large conference room with chairs and space to break out into groups for the duration of training (ideally, an additional space with several, small rooms can be useful for conducting individual mock interviews with visiting or play doctors)
- Printer and printer paper
- Pens
- Small notebooks for SPs
- Watches to capture time (otherwise, personal mobile devices can be used)

If a survey firm is contracted for data collection activities, then it is likely that receipts will be needed for the interactions, which can cause an issue with interrupting the natural development of the case at the health facility.

7.3 Training Activities

The following are training activities with short descriptions of what a training team can incorporate, should focus on, and look out for.

Continuing selection of SPs

One of the main ongoing activities throughout training is selecting the individuals who will become the SPs for the study. What characteristics define a good SP, and can these be seen during the training?

- The abilities to memorize, play the part, be convincing in the role, improvise, not be overconfident or lacking confidence, and answer consistently even though the question can be asked in different manners.

Activities throughout training that help develop the characteristics of a good SP

- *Developing attentiveness* – Stand-up exercises are helpful to bring attention back to the objective of the project. A session lead can pair people up and ask them to describe what the other person is wearing, what his/her expression looks like, how the person feels, etc.
- *Building oratory skills* – The trainees should be selected throughout training to stand up in front of the group and present the script or case.
- *Gaining clinical understanding* – Clinicians can participate in the training and provide presentations for each condition represented by an SP case. The clinicians can return throughout the training to act in mock interviews and provide a similar experience to shape the SP recruits' learning and set of expectations. Clinicians can lead discussions on characteristics of the typical presentation of patients when they come to the facility, how the patients act, what their facial expressions look like, the worry they carry, the type of family situation they come from, and more.
- *Memorization techniques* – It must be communicated to the trainees that the memorization of their character and the scripts is mandatory for their selection to participate in fieldwork. Also, randomly selecting trainees to respond to questions and varying the training techniques in the classroom can support the learning.
- *Building confidence but maintaining self-control* – Although SPs will be trained to become experts in their cases and scripts, it is important that they do not bring that expertise with them into the clinic, as it will interfere with the study achieving its objectives, and it may also create a risky situation for the SP. "Building confidence" here refers to the SP confidently internalizing the case and script to an extent that the case becomes somewhat seamless with the SP's real persona. With training, the SP should not be nervous in portraying the case, and if so, he or she has not yet internalized the case or built confidence in him or herself in enacting it appropriately.

Throughout training, different training techniques can be used in the classroom. Forming the trainees in different groups and varying the techniques throughout training will help increase attentiveness, allow everyone to focus on specific objectives at appropriate times, and create an enabling environment for training. Techniques that have worked in the past are shown in Box 7.3.1.

BOX 7.3.1. Classroom techniques

1. Large group discussion – Having all the trainees sit in a horseshoe encourages a good group dynamic (which will be important for fieldwork), larger group discussion and learning from other trainees and lessons across all the cases. This technique also discourages trainees from straying from the immediate task and whispering to their neighbor a question or discussion point that is relevant for everyone in the room. Towards the middle of week 1, the trainees should be able to respond to being picked randomly from the session leader to answer questions relevant to their case.
2. Supervisor-led training – Responsibilities for training should be 'decentralized' as much as possible to the individuals who know the fieldwork areas best, use the field protocols, and will be meeting the SPs after their interactions with providers. These individuals by design should be the supervisors who participated in SP and provider recruitment.
3. Groups by SP case – Together the trainees can learn the case, share experiences of family and friends with the condition, understand, and memorize the details for each case.

Activities by week of training are provided below with checklists for the training team (also see Annex M). Throughout these activities, one or more supervisors or other designated individuals should keep track of everything that happened. The information is very useful for feedback or debriefing sessions that can occur amongst all participants.

Week 1 activities

In the middle of week 1, it is good to begin providing individual feedback. Many times, the reason for letting an SP go during the initial stages of training isn't because there is a problem, but because the cases become more refined. Additionally, whether an individual fit that role does not correlate with how good or bad his or her performance as an SP is. A week 1 activity checklist for the training team is as follows:

a. Introduction

- Sell the project to the trainees
- Set clear expectations – Because more SPs are trained than who will actually go into the field, it is important to make this clear and let them know which aspects they will be selected on. One way to do this is to have a week-by-week scoring rubric where the various items for assessing training are differentially weighted based on the learning objectives and desired outcomes.

b. Script and exit interview learning activities

SPs are separated into groups, one for each case, to develop the scripts with the guidance of a supervisor who has been tasked with that certain case.

- Introduce the scripts:
  - Have each SP read the first page of script. Each of the SPs should have a chance to recite the script.
  - Answer any questions or clarify any aspects that are not well understood.
  - Maintain a list of items the SPs bring up in discussions for the script.
- Teach the scripts by case:
  - Conduct exercises in small groups that encourage the SPs to internalize aspects of the scripts, such as discussing the precise age of case, where they come from, or what they do for a profession.
    - For example, the trainees can be told, "*Close your eyes and picture your case. What they are wearing? What type of shoes are they wearing? Build the person without being sick. Where are they and why are they there? What are they thinking?*" Then go around the circle and ask each trainee what he or she pictured.
    - Map out the day – What would this person do every day?
- Build upon their world experience in large group discussions:
  - Have all the trainees return for large group discussion and ask if anyone has experience with the disease. If anyone in the room is willing to volunteer their experience, good questions to ask are:
    - What were they doing when they felt sick?
    - What alerted them that something was wrong?
    - Why did they decide to go to the clinic?
- Teach the opening statement and questions:

- Go over the opening statement and questions. Supervisors leading each group by case should keep notes on suggestions from the trainees, and the trainees can focus on their tasks. This is a chance to discuss any aspects of the script that may be missing but important to include. During this activity in other projects, questions that have been added to the script include:
  - *How will you make the payment today?*
  - *Have you been traveling recently? (Answer: No)*
  - *Is there anything else you want to tell me?* (*Answer: No)*

- Confirm the translations:
  - Translate the script while reading aloud.
    - If the script is written in more than one language (e.g., English and Kiswahili), deliver the script in one single language (e.g., English) even though the actual script is in both languages. Then have one SP start reading the script in the other language (e.g., Kiswahili), translating while reading. It is important that the supervisor keep track of the discussion, especially the words or phrases that caused discussion and what the solution proposed by the trainees was.
      - Preparations: Edit the translations out of the script only for this exercise.
    - This exercise is good for both cleaning the existing translation and helping the SPs think together about the interpretation of the words. Equipped with the notes, the supervisors can revisit suggested changes where the interpretation of one language affects the intended meaning of the other. These can be reviewed and resolved with the research team and the Technical Advisory Group if necessary.
- Conduct a script learning icebreaker:
  - o Set an order among the SP recruits. Have one of the SP recruits start the story in the script, and after one sentence, the next SP continues the story for one sentence. Follow this pattern until the script is complete.
- Hold re-enactments for large group feedback.
- Conduct a first mock with a real clinician or other health care professional. At the end of week 1, each SP can have a one-on-one consultation with a clinician from the advisory group. Doing this the first week creates an opportunity to group the trainees for better learning. For example, a project team may decide to group the trainees into

three main groups: (1) trainees who would be perfect SPs, (2) trainees who are good but have room for improvement, and (3) trainees who may not improve. This activity can be repeated at the end of week 2, and the progress across and within trainees for each SP case can be achieved with more targeted intervention by the project team. Further, there are several sub-activities here:

- The clinician can collect vitals (e.g., blood pressure, pulse oximetry) as a second stage of health screening, but also to prime the potential SPs on what to expect at the facility.
- The clinician can rate the trainees after all have consulted. An excel can be created to track the following indicators:
    - SP name
    - Ranking of SP trainees by general performance from clinical standpoint; this should be completed by the visiting health care professional after all consultations per case have been conducted. This is so that the training team can have a clinical perspective on which SPs may require further work in order to prevent detection. The training team should also be aware of the general ranking of SP trainees throughout the entire training period.
    - Clinician assessment/comments
    - Feedback given to SP trainees after mock interviews
    - Feedback given to trainers on what to focus on in week 2

c. Initial internalization of the character

- Practice storytelling
    - Have the trainees read the scripts. Then have the SPs put the scripts down and retell the story in their own words, in the first person in front of group members. Have the other trainees ask questions to the character.
- Mimic real-life scenarios with the SP characters
    - Have the trainees in character pretend like they are meeting each other on the bus or during lunch with mutual friends. How would they act? What would they talk about?

d. Risk mitigation strategies

During the first week, the SP trainees should be provided a thorough overview of risk mitigation strategies, which will be reinforced in training weeks 2 and 3. Below is a list of events that can put the SP at risk, followed by sentences the SPs can use to avoid the event.

- Injections
  - *I am allergic to the injections.*
  - *I am taking other medications.*
  - *I never get injections. They make my very uneasy/squeamish.*
  - *I am very scared. Please do not give me an injection.*
  - *I don't have the money to pay for this, doctor.*
  - *I don't think I need this. Can you give me more information so I can think about it?*
- Tablets
  - *I am nauseous and feel I will vomit.*
  - *I have not eaten since last night.*
  - *I have just taken other medicines. Let me take your medicines home, and I will take them later.*
  - *I will wait until I get home. I do not want to feel dizzy.*
  - *The SP can also pretend like they have taken the tablets when the provider is not looking.*
- Syrups
  - *I am nauseous and feel I will vomit.*
  - *I don't have the money to pay for this, doctor.*
- Blood pricks
  - *I have taken alcohol.*
  - *I react to pricks very badly.*
  - *I bleed a lot. Another doctor said I have a problem clotting.*
  - *I don't have the money to pay for this, doctor.*
- Blood draw
  - *I am not ready to do this now.*
  - *I want to think about this.*
  - *I need to speak with my husband/wife about this to see if it is ok.*
- Intravenous fluids
  - *My religion does not allow me to do this.*
  - *The last time I had a needle, I reacted badly.*

Towards the end of week 1, if applicable, the trainees should be advised that they might be conducting the interaction with providers with audio recorders (see Section 5.6). The objectives of the audio recordings should be explained to the SPs. The objectives of this are:

- To help the project team verify what has happened in the field and compare it with what has been reported during the exit interview,
- To listen to recorded mocks and learn and adjust,
- To check memorization, especially for projects where fieldwork is long in duration, and
- To improve accountability.

Training on audio recorders can happen in week 2 or 3. It is normal to expect a bit of anxiety from the trainees and SPs with the use of audio recorders. As previously mentioned, each trainee should have at least 2 dry runs with the audio recorders.

Week 2 activities

During week 2, supervisors switch from being coaches to being supervisors (from encouraging a good learning environment (i.e., "I will help you get to know your character better, because I don't want to let you go") to making sure issues don't occur in the field (i.e., "I will be your supervisor in the field, and my job isn't to babysit.").

Since the trainees enter week 2 with a good idea of their cases and scripts, keeping them in case-specific groups is less necessary. There should be a shift from learning the case-specific conditions and scripts to being put into different situations and learning how to respond to them correctly. For this reason, week 2 activities draw on mock interview sessions, which allow the SP trainees to encounter the range of what they may experience in a provider's office.

These activities are designed to help evolve the trainees from the previous week and further develop the different skills learned in week 1. Among general improvements and increased comfort levels, other aspects of SP trainee learning can be assessed to determine whether trainees are well suited to continue in the training, including:

- Ability to act and stay in character
- Ability to observe
- Ability to recall and restructure memories to communicate them later
- Ability to improvise and respond with the standardized case

During week 2, it is also likely that the trainees will begin thinking of different scenarios that could happen at the clinic. During these moments, the training team has the responsibility to remind the SP trainees that they are a character who is worried about their health (or their child's health) and to proceed in character regardless of what happens at the health facility, as long as their actions do not put them (or others) at risk for danger or detection. A week 2 activity checklist for the training team is as follows:

a. Increasingly complex mock interviews

- Rotate among the SP trainee groups – Supervisors and other members should rotate across the case groups and take turns leading each group. This not only prevents the supervisors from getting too attached to the trainees in their group, but also allows them to learn the different cases (at this time, they have only been training and managing the translation for their assigned case).
- Advise trainees to come dressed as the SP case – This will help them internalize the character and provide an opportunity to hold a group discussion on what would be within the range of appropriate clothing and dress for the case descriptions. Box 7.3.2 contains an excerpt from *Details Matter* by Das V et al. which describes how the authors decided what physical characteristics were important while deciding on how SPs should dress in a study in India.

BOX 7.3.2. Excerpt from *Details Matter* on SP clothing and dress (19)

What kind of clothes would the SPs wear and what did their body language say? Let us start with the urban context. The patient with myocardial infraction did not present any major issue since most men of low-income communities wear trousers and shirt. To the initiated eye, the type of trousers and shirt that men from low-income areas wear is different from that which men from relatively affluent backgrounds will wear. Often the clothes that men wear are bought from the local markets or from street stalls set up by local entrepreneurs. One could perform a whole semiotic of the difference in the colors and the cuts – the best way to avoid any mistakes in this regard is to see that the SP wears his normal clothes. If clothes need to be bought for the SP then it is best to buy them from markets in local neighborhoods. The idea is that the SP should blend in the local environment.

Clothes for the women did require some creative thinking on our part. While the thirty year old woman could dress in the normal ensemble of salwar, kammez *and* duppata *[Indian type trousers, long shirt and long scarf] with the signs of married women on her body –*

Kwan A, *et al. BMJ Global Health* 2019; 4:e001669. doi: 10.1136/bmjgh-2019-001669

e.g., a touch of vermillion, a bindi *(red dot) on the forehead and some light jewelry – it was important to remember that bangles worn in the communities included at least a few glass bangles and that the earrings and chain should reflect the fact that it is "costume" jewelry worn by women in everyday contexts. In contrast, the jewelry worn by upper caste or relatively more affluent women will be of gold.*

We also learnt that married women wore bichhuas *(silver toe rings) in these communities as an obligatory sign of marriage. During the interview process, we had to ask the unmarried women who were interviewing for the role of the married mother if they would be prepared to wear these signs on their bodies – while bangles, earrings and the red dot posed no problem since unmarried women too wear these signs – the vermillion on the parting of the hair and the toe ring led to some discussion. This is because these are culturally loaded symbols and an unmarried girl would be accused of being secretly married or having a secret lover if she were to display these signs on her body. One of the women suggested the compromise that the vermillion could be just a touch and the toe rings could be thin bands of silver rather than thick bands with decorative motifs. She pointed out that though she was herself married, she did not wear these signs prominently on her body, because she was working in a "modern" setting and therefore did not consider it appropriate to signal her caste or class identity so prominently. In the end, we did not exclude unmarried women for this role, since we saw that we could replicate the kind of improvisations people make in the flux of everyday life.*

b. Mixed-case group sessions

- SP recruits are randomly assigned to different groups of 3-4 individuals. Individuals take turns: (i) presenting the case, (ii) being the doctor, and (iii) observing and giving feedback.

c. Run through scenarios that SPs may encounter at the facilities, such as:

- No change available when paying consultation fees – What happens when the cashier says that the medicines for the SP's consultation will cost 20?
- Bribing – The pharmacy asks the SP for a bribe.
- Doctor on the phone – The doctor's phone rings, and it sounds like he is fighting with his spouse. The phone call continues and continues.
- At this stage, the team training the SPs can also show videos of clinical visits to see if the SP recruits can articulate opinions on the clinical setting. Here, by watching the video, the point is to see the opinion of the individual. For example, opinions

that SPs have given were of the following kinds: "The doctor was good – he gave an injection – injections cure much more quickly than oral medicines" and "The doctor was good. He gave an injection but he should have tried to console the child" and "I cannot say if the injection was the right medicine but I feel he should have asked the mother some questions." This focus group type setting allows SP recruits to have discussion around issues that they may confront during a clinical encounter. At this time, recruits who applauded providers for certain actions should not be excluded or included for their opinions, but the team should listen carefully to ascertain if they were able to listen and think when other opinions were offered. Recruits who are not able to listen and think when others are expressing their opinions may not be strong candidates for the task at hand.

d. Coach SP trainees not to fall out of character

- SPs must be trained to be just confident enough to take on the characteristics of their SP case. During the interaction, an SP cannot be overconfident (e.g., the SP cannot pretend to be a know-it-all and begin offering unsolicited information to the provider, or show the provider that she knows a lot about the disease).
- SPs must be trained to be nonjudgmental of the providers and any health facility staff during all interactions. During the interaction, the SP must not come out of character if a provider is particularly mean or happy.
- During the exit interview, the supervisors will ask the SP their impression of the provider or health facility with subjective questions, and this is a moment where SPs do not have to be in character, and in fact, they should be encouraged to provide their personal impressions.

e. Memorize the exit interviews – The purpose of memorizing the exit interviews is to ensure SPs begin practicing how to correctly and completely recall aspects of the clinic visit found on the exit questionnaire.

- The training team should encourage the SPs to remember exit interview questions in the order in which they will be asked during data collection. Rehearsing the order of the sections and the questions will help memorization.
- Depending on the structure of fieldwork, some aspects of the questionnaire will not be necessary for the SP trainees to memorize. For example, if sampled health providers send patients away with a paper noting prescribed medicines and the project team decides to purchase the medicines from each interaction at a local pharmacy, these physical components of the interaction can be catalogued and

entered long after the SP's recall period extinguishes (though it is recommended that even with a cataloging and data entry process, these interaction artifacts are entered into the exit questionnaire or photos are taken of them the same day or day after the interaction so that the supervisors can note the interaction as complete for management purposes).

f. Administer tests on material covered during week 1

g. Conduct mock interviews in different areas

- To acclimatize the SPs to various scenarios that they may encounter in the field, supervisors can:
    - Stage interactions in distracting environments, such as in front of people setting up lunch or outside in a parking lot where there are pedestrians.
- To test distractibility while conducting mocks, supervisors can:
    - Answer their phone and have a full conversation
    - Project on the wall a movie showing traffic with noises

h. Assess audio recordings

- The best way to assess trainees on recall is to conduct mock interviews with audio recordings, followed by a full exit interview debrief. Exit questionnaires completed with the audio recordings only can then be compared with the exit questionnaire data. Another way to assess trainees on recall is to play an audio or video taped interview between a doctor and patient, create a gap in time where recall and memory of the interaction may begin do fade, (i.e., where they might have to walk from one room to another), and then test the SP on what questions the doctor had asked. After the SPs have answered what questions the doctor had asked, the trainers can also give them a prepared sheet of questions and answers and ask SPs to report if each statement was mentioned (true or false). Since these skills are developed in training weeks 1 and 2, at this stage SPs should be able to recall about 70% of questions correctly. This provides a good cut-off point for determining a desirable versus a less desirable candidate.

Week 3 activities

The third week of training starts with mock interviews to allow for SP trainees to practice both their recall and improvisation techniques. The week culminates in dry runs, which allow SPs to practice everything they learned in scenarios that are closest to the real SP interaction. The dry

runs are also an opportunity to see what types of medicines, injections, and blood tests are generally prescribed, conducted, or ordered for the cases. The week 3 activity checklist for the training team is as follows:

a. Dry runs

- Identify providers and facilities where SP trainees can conduct dry runs – Providers used for dry runs are not the ones who have been recruited or sampled for the actual fieldwork. Members of the fieldwork team may personally know the providers or be familiar with them to some extent, but they can also be providers with no previous relation to the team.
- Assign SP trainees into teams of two (recommended size) - Each team of two is made up of one SP and one accompanying person.
- Practice arriving on transportation before the facility – During the dry runs, the team has the task of finalizing the procedures for arriving by local bus stations or similar large public spaces before heading to the provider clinic.
- Instruct SPs before they head to the facility – SPs should be given clear instructions on where to go after visiting the health facility. This is the location where each SP will debrief with the supervisor.
- Maintain constant communication with the SP trainees throughout the day – This can be done through supervisors who are charge of a subset of the SP trainees. Be prepared to troubleshoot issues related to locating providers or facilities, navigating traffic, and encountering busy health facilities.

b. Dry run debrief sessions

- Set a meeting time for when SP trainees should return to the training facility – This meeting time is ideally in the afternoon with the assumption that the dry runs can occur in the morning. From the experiences of other SP projects, it is often the case that, especially for trainings that take place in urban areas, SP trainees encounter long wait times, traffic congestion, transportation issues, provider consultation times with small windows, and more during dry runs. These are very likely to occur during dry runs (but will improve as fieldwork goes on), and it is good to make the trainees aware of these possibilities. Many times, pairs or teams that go further or to busier areas for dry runs may return late, but it is still worthwhile to all the SP recruits and the training team that everyone is able to debrief their experiences and learn from others' experiences. In special cases, it is not necessary to require all SPs to wait until everyone has returned,

and debriefs can occur over phone with the training team or included in the following day debriefs. Similarly, not all dry runs will be completed on the suggested day, and these opportunities can be shifted to a subsequent day.

- Debrief together - Debriefing sessions are designed to occur as a large group so that SP trainees, the trainers, and the project team can learn from all the encountered experiences, and so that everyone is present during the resolution of field challenges or confusing protocols. This is also a chance for the project team to incorporate, edit, or eliminate any aspects of the cases, scripts, or exit questionnaires. Debrief sessions are not unique to training, and it is strongly suggested that they be scheduled to continue through the pilot and throughout fieldwork.

With an experienced team or for an endline study, the dry runs can serve as a pilot. If this is the case, the following elements can be considered during dry runs:

1. Completing exit questionnaires – Experienced SPs and supervisors can be responsible for completing the exit questionnaires for dry runs.
2. Iterative design of questionnaires – A project team, supervisors, and SPs can use dry runs as an opportunity to iteratively design questionnaires in real-time. After each iteration, the SPs are trained on any changes and then sent to the field again, followed by a debriefing session. This is not recommended for project teams implementing the SP method for the first time or for non-experienced SPs as the iterative process can result in confusion on what the final case is.
3. Localization of clinical words – Sometimes these are learned in the field, and the team can incorporate this task into the dry runs to improve the SP cases.
4. Refresher training – Some research projects have several waves of data collection. In these types of projects, a team of SPs will have already undergone a 3-week training and even periods of fieldwork, but can experience short breaks in activities. In these situations, dry runs can serve as refresher training without needing the team to undergo the Week 1 or 2 activities. As mentioned in Section 7.1, memory retention from training lasts for four months. Because of this, longer refresher trainings should be organized if there are longer breaks.

BOX 7.3.2. Confidentiality reminder

SPs must be reminded that they cannot gossip about what they have seen in training or in interactions. They must not reveal to anybody in public, social media, or anybody unrelated to the project that “such and such practitioner asked me that stupid question” or any other specific details experienced in a consultation. If asked by family or friends what the job entails, they may say that their work is like that of a market researcher or for the census. Trainers should use locally embedded analogies to communicate the basic concept around confidentiality and how to explain the SPs’ work to others in the community without breaching the confidentiality agreement.

SECTION 8. OTHER TRAINING CONSIDERATIONS & PRE-PILOT PREPARATION

8.1 Assessing study environment for SP work and learning from other projects

The environment where the SP work is planned can introduce many challenges to the project team. Luckily, assessing the environment ahead of fieldwork and learning from the experiences of other projects can help prepare the team to best respond to challenges posed by the setting of interest. Dry runs are an opportune time to learn and also identify the challenges that may be encountered, and debrief sessions with the SPs after their dry runs can allow for a space to resolve the challenges and suggest protocol that can be followed in the future. In this section, lessons from different projects are described, as many specific challenges can be fairly contextual.

What were challenges and lessons from the Qutub project in urban Patna, India?

Addresses, **transportation,** and dust were all challenges in urban Patna. At the time of the study, public transportation did not access interior sections, and staff found themselves walking around communities without signboards or road signs. For these reasons, it was difficult to find providers, particularly at the beginning of fieldwork.

1. At the beginning while the team was becoming acquainted with the area, supervisors resolved that it would be easier to ask rickshaw drivers, owners of side shops, and pharmacists. These people either taxi individuals around the city often or have been in the areas for decades, and thus knew their way around. Pharmacists in the area also received printed prescriptions with addresses and hours of doctors, so they were good informants of where providers could be. Similarly, clinical laboratories were also helpful.
2. Additionally, upon receiving provider universe lists from a city-wide mapping activity conducted by a non-governmental organization (NGO), the field team used Google maps to review each area immediately before physically entering the area, but often times the addresses didn't match up with the specific addresses from the mapping lists. However, Google maps was helpful in locating monuments, neighborhoods, and how main roads connected.

3. For some providers that were not easily located after several weeks or even several attempts, the field team was able to contact a local NGO who was working with the health care providers across the city.

What were challenges and lessons from the Qutub project in Mumbai, India?

The **heat and monsoon rains** impacted transportation use and movement across the city; **addresses** were not existent in slum areas; getting from one place in the city to another in the city often times took 1-3 hours because of the **scale of the city**.

1. The pre-monsoon heat and monsoon rains in Mumbai were considerable enough to change the fieldwork schedule. (This change did not have any effects to the project itself). Specifically, since the monsoon occurs in July-September, the field team preferred to start fieldwork in January and aimed to finish by April when the heat arrives in India. Whether a project team should adjust fieldwork schedules is also a matter of whether seasonality is relevant for the health condition of interest to the study.
2. To some extent, there was no complete resolution for overcoming the transportation times and traffic in Mumbai. However, over the course of fieldwork, the field team became familiar with the city and was able to plan SP interactions based on what they knew about traffic and rush hours. They could additionally plan around city events. Locally recruited SPs were also helpful in maneuvering the traffic throughout the city.
3. Similar to the experience in Patna, the team was able to locate providers without addresses by asking rickshaw drivers, owners of side shops, and pharmacists.

What were challenges and lessons from projects in rural areas?

Project teams conducting SP work in rural areas can also anticipate transportation challenges, as well as the challenge of minimizing the SPs' risks of detection.

1. With respect to transportation challenges, field teams of rural projects have several lessons. First, if there were a village, they would stay in the village and then send SPs and supervisors by foot to conduct the interaction. Second, understanding local public transportation routes has also been helpful (e.g., in one project, there was one bus that would go once a day to an area that had one sampled provider). Third, if the SPs can hitchhike safely, then that can be an option managed by the supervision staff.
2. SP risks of detection may increase when entering an area where (i) the narrative of the SP may not be usually encountered, (ii) if everybody knows everybody in a village and an unfamiliar person enters, or (iii) if the language, dialect, accent, or context of the

individual does not match the people in that area. Training strategies can mitigate many of these challenges. For example, SPs should be trained to come up with a story regarding how they were traveling along the nearby road to get from one nearby village to another when they came across the provider of interest. The provider can be someone that a person in one of the villages recommended.

What are other challenges and lessons across other settings?

1. Some settings are easy to navigate because of transportation systems. It is further helpful to have provider-mapping lists contain details, such as addresses with landmarks. Written instructions are useful.
2. Some areas have multiple names, and the processes of naming these areas can be deregulated so do not have addresses. Leveraging local knowledge of local SPs can help the larger field team figure out how to get to and from locations, and decide on the most convenient modes of transportation. When a field team is unfamiliar with the area, it is expected to be challenging for the first couple of weeks, and over time, the team will get more comfortable with navigating.
3. Costs over time for transportation are important for an SP study to consider. It is expected that moving the team around the study location will be more expensive than later parts of fieldwork. After some time, transportation costs become cheaper. Sometimes, train, bus, and rickshaw options are available. Usually, it is worth taking a longer route for cheaper costs.
4. Local language considerations are nontrivial. Local SPs can help.

Provider consultation hours can have huge variation and can differ across settings

Doctors in Patna, India often work in public and private facilities, and so in the private sector, in which the study was situated, the doctors would be available in the mornings and late evenings. Late evenings were challenging for staff movement, because the city shuts down at 10pm. Going far and coming back became risky, and there were safety concerns. In Mumbai, India, some health providers had very strict consultation hours (e.g., 10-12pm, 3-5pm), and for specialized doctors, it was easy to obtain appointments over the phone. In contrast, any appointments had to be made in person in Patna.

Funding and per diem for the SPs

The model that has worked well in previous projects is for SPs to receive per diem for their time during training, and only once training is completed, SPs are hired. One option is to contract the SPs by day regardless of interactions conducted, since there might be issues with doctors' clinics being permanently closed, long travel or wait times, or outpatient hours not being convenient for a given day. Paying SPs per interaction is not advisable, because this may encourage them to finish more cases in a hurried manner. In this case, the SPs may also find it unfair that they were not being paid when the interactions are not completed to no fault of their own (e.g., they might go to the clinic, but the provider might refuse to see them or the clinic might be closed).

Field team living arrangements

Different living arrangements can be made. For example, the supervisor team in the Qutub project that took place in Patna and Mumbai arranged monthly housing in both cities through contacts. For a project in a more rural setting, supervisors and SPs can assess the costs of setting up accommodations in the nearby town and commuting to the facilities of interest.

Other challenges

Some other challenges and corresponding strategies that supervisors can implement are:

1. SP cases often describe the profession or job of the case. During an SP interaction, providers might inquire more about these jobs and the place of hire or training. If this is not anticipated in advance, and the SP says something that is off kilter, it could increase the risk of detection. Relatedly, in some cultures, it is possible that the female SPs may receive questions about the home and the family. The preparation done during mock interviews and the lessons from dry runs can help standardize these additional aspects of the script. Additionally, SPs can be prepared to improvise or respond to these questions.
2. If staying on schedule or keeping time based on fieldwork protocol becomes a challenge, additional time can be allocated to training or to fieldwork to avoid hurrying field staff for unnecessary reasons.
3. It is becoming more common for some health facilities or doctor's clinics to have surveillance cameras, such as CCTV cameras. Since this is a possibility, instructions can be given to the SPs to not bring out any pieces of paper, such as provider lists or schedules, in public to avoid capture on camera.

8.2 Revisions in the script

Depending on the environment, the project team may realize that necessary revisions need to be made to the script. The narrative for example needs to fit the setting (e.g., the urban area or occupations and corresponding salaries in that area). These details are obtained by talking to people in the community and observing people, especially the ones that are being portrayed in the cases desired. Having local SPs also provides access to patois for proper translation and other details related to the case (e.g., rent, earnings, living conditions). For example, in Patna, India, to develop a case's profession or day-to-day work activities, the Qutub project team found it useful to observe that security guards were quite rare, but salesmen were more frequent. The team decided to include the occupation of salesman for one of the cases. Then, as the project team shifted script development to another city, the team found that being a security guard was more frequent in Mumbai, so that became one of the professions for the same case.

8.3 Change in case allocation

Generally, cases are assigned based on the profile developed for the cases. From the experience of other projects, three main conditions were used to base assignment. The first condition to fit is age, since recruited SPs should fit anatomically and physically to the case. The second condition is appearance to fit the case, and sometimes SPs are asked to wear specific types of clothes or to not shave for men, if relevant for the case. The third condition is performance. Some cases are more complicated than others, and individuals who are able to outsmart the conditions that are given during training (how do they act in times of stress, deal with a variety of questions) are allocated to the more challenging cases.

During fieldwork, SPs can be switched with another SP or to another case for fieldwork when they have already been exposed to providers and their interaction could increase the risk of detection. Switching around SPs across cases also allows for a shifting of human resources when other fieldwork limitations exist, or if training is occurring with new and old SP recruits. However, the project team must be cognizant of other potential issues or areas for increased risk of detection as a consequence. For example, if an SP switches from an asthma case to a tuberculosis case and both cases are to be presented to the same sample of providers, the supervisors must take care to ensure that the individual does not visit the same provider as both cases.

8.4 Removal of non-suitable SPs

Supervisors have to be strict and committed to supporting the individuals recruited for SPs as much as possible; however, if poor behavior continues even after an individual is told to correct his or her behavior, supervisors may want to consider "letting go" of the SP from practice or from the project. When letting go of an SP, the SP should be reminded of any confidential agreement that is signed at the beginning of training between the recruits and study team (e.g., the one included in Annex I says the SPs are not able to speak about the study and if they are found sharing this information, legal action could be taken). SPs are also continually informed about how important the study is, not only for motivation, but also to make sure they feel responsible for the study and are members of the entire project team – this should also be re-emphasized when letting go an SP, in addition to the appreciation of their efforts. Other reasons that might warrant letting an SP recruit go include:

- Poor physical match – If there is no match with the physical description representing the health condition, potential SPs could be let go.
- Poor recall – During interviews of several candidates, one project team found some of the interviewees for potential SP roles could not recall the necessary information during the exit interview debrief period. This issue can also be identified during the training. To identify these issues, the project team can ask whether the SPs are able to not just recall one interaction but several interactions that can occur during the day? This will create a problem and if the individual is unable to correct after 1-2 days of training, he or she will not be a suitable candidate and may pose a risk to the supervision team and the larger study objectives.
- Not listening to rules or safety protocol – If any of the SPs are not able to escape harmful situations, because they are not able to get themselves out, be rude, or speak out to push away from the doctors.

However, if an individual is to some extent a good fit but other recruits are stronger, the team may decide to let the SP go, but inform the SP that the team may reach out to him or her in the future in case a spot does become available.

8.5 Add-on tools to test during training and dry runs

- Technical and operational supervisory checklists
- Confidential agreement for SPs to sign (see Annex I).

Kwan A, *et al. BMJ Global Health* 2019; 4:e001669. doi: 10.1136/bmjgh-2019-001669

SECTION 9. PILOT

9.1 *Daily fieldwork plan for pilot*

From the SP perspective, a day in fieldwork is depicted in Figure 9.1.1. This figure is helpful to demonstrate several aspects of fieldwork, especially those that may differ from other types of data collection. Notably, these include:

- SP daily workload – Multiple providers or facilities can be seen in the same day, depending on the plan and organization of the project. Generally, a good place to start is to schedule an SP to complete one interaction in the morning and one in the afternoon or evening (this is typical from the Qutub project in urban India).
- SP autonomy – The SP does not spend the entire day accompanied by the supervisor. Instead, it is common for there to be several SP-supervisor touch points throughout the day. There are several reasons for this. First, each supervisor is likely to be responsible for several SPs. Second, an SP should conduct visits alone unless the case script specifically is written for otherwise. Third, SPs and supervisors should not be seen together, as this will raise detection risks, since the supervisors will be seen around the study area and may be known as part of a study team.
- Number of attempts before a successful visit – The project team will need to decide how many attempts an SP should make to successfully complete a visit with a provider. In the case when providers had small windows for consultation hours or when a clinic was closed for an unknown duration or when a provider was on vacation for an unknown period of time, the Qutub project team allowed for up to and including three recorded visit attempts before the provider would be "dropped" from the schedule. In order to reduce attrition rates to the study, these providers who had been dropped from the schedule were attempted one last time at the end of the data collection period.

Daily fieldwork responsibilities for the supervisor parallel those for the SP; however, generally a supervisor, which can be senior or junior, is responsible for a group of SPs. In some projects, senior supervisors have also managed junior supervisors, who then manage SPs. In large-scale studies, the supervisors can improve workflows by rotating office responsibilities, such as assigning one supervisor to remain in the office while the others go to the field. Daily office responsibilities for the supervisors include: printing, scanning, completing the medicine section

of the exit questionnaire forms, double checking completed forms, entering tracking form data, preparing for upcoming SP visits, communicating with the research team.

Figure 9.1.1 SP daily fieldwork plan

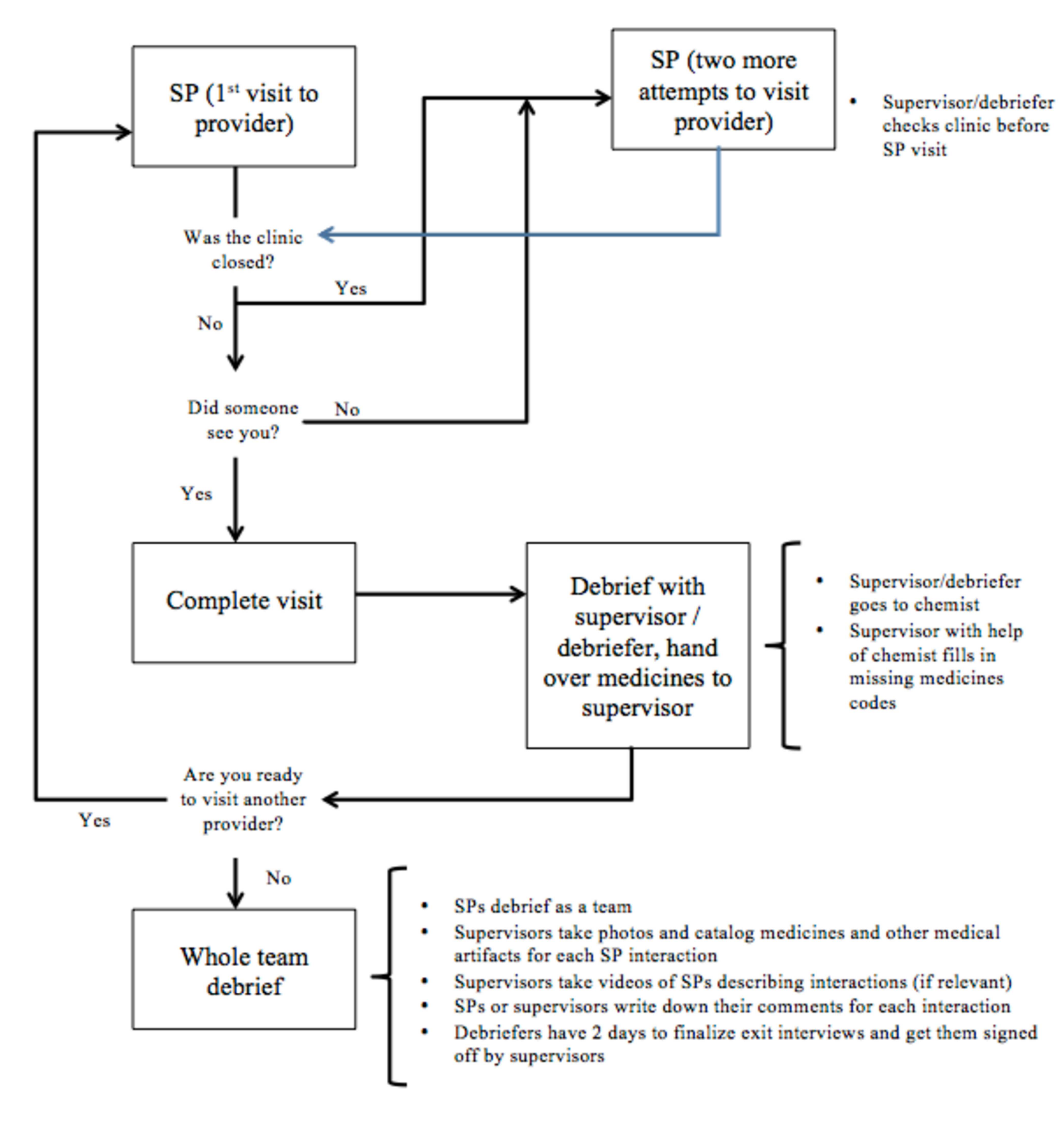

As for daily supervisor field responsibilities, each supervisor takes a small group of SPs (3-5 people) who will be visiting the same geographical area each day. For the Qutub project's pilot in Delhi, supervisors took groups of 3-4 people per day. Modes of transportation that were used included: motorcycles, public transportation, and cars. If there is one motorcycle and the study is in an urban area, a supervisor can take one SP and tell the other SPs to meet at a landmark that is near the location of interest. Landmarks are more preferable as meeting places than areas closer to health facilities, since lingering can cause suspicion among providers or others who may alert providers.

If there are multiple providers who should be visited each day, the SP can go to another provider with public transportation or the supervisor. When the day's work in the field is done, everyone can return to the debriefing location. For the same reasoning as those described for dry runs in Section 7.3 under week 3 training activities, debriefing together as a team should be incorporated as standard fieldwork practice, especially in the initial stages of fieldwork. As the fieldwork team and especially the SPs become more comfortable with their responsibilities, debriefing together may be less frequently needed. The field team may decide to do weekly debriefs together.

Since the SPs provide payment to the provider or the health facility, logistics have to be set up for them to pay for each visit to a doctor or pharmacist, as well as any travel costs involved. One way to set up these logistics is the following. The supervisor can give cash to each SP for transportation and any per diem, purchasing medicines, and consultation fees over a time frame that works for the field team. At the end of each day, SPs then report back how much has been spent. When an SP's money is starting to run out, the SP can get another "top-up" from the supervisor. Anything that's spent in the field for the fieldwork is in this lump sum. Supervisors put these in their log each day, and supervisors also check in with the SPs to see if they have enough money. Supervisors are also responsible for making sure that SP funds are in appropriate denominations (typically smaller), which may involve coordination with local banks or other financial services providers.

Challenges for fieldwork will include managing the variety of people involved (young and old, male and female). A professional stance should be maintained throughout fieldwork.

Kwan A, *et al. BMJ Global Health* 2019; 4:e001669. doi: 10.1136/bmjgh-2019-001669

9.2 Schedule for the SP visits

Both supervisors and SPs can manage their fieldwork best with personal fieldwork schedules. See Annexes N and O for personal supervisor and SP fieldwork schedules, respectively, as well as Annex Q3 for the entire fieldwork schedule and tracking sheet, from which the personal supervisor and SP fieldwork schedules are drawn. The fieldwork schedules or visit roster should list the name of the doctor (or chemist), the name of the doctor's clinic, address, other location details, and tracking details for up to three visits. If the project is more complex and includes micro-experiments (also referred to as experimental or case variants), details relevant for the different SPs should be included in the visit roster (e.g., a project might want to randomly assign SP interactions by: SP gender, morning or night outpatient hours for the interaction time, case if there are multiple cases, or other potential variants or add-ons to a case).

9.3 Potential schedule issues

Fieldwork management issues to consider during the scheduling include: confusion of which SP should be sent, getting lost, and address updates. Many of these issues can be mitigated with strong feedback loops for cross-team communication, careful planning among the supervisors, and clear instructions on organization and reporting structures. When an SP gets lost, he or she should know to call the supervisor to troubleshoot, for example, by obtaining information on how to find the place of interest or organizing a way for the supervisor to get them.

Other schedule issues can be in some regards outside the realm of control of the field and project teams, such as those related to the setting of the study (for further discussion, see Section 8.1). For example, in Mumbai, unpredictable weather patterns during the monsoon and transportation issues were daily and seasonal challenges. In urban Patna, pollution and very cold winter mornings affected the opening hours for the clinics.

9.4 Data collection checklist

This section provides a checklist for data collection needs and the purpose for each.

Equipment needed for fieldwork

- Camera – to take pictures of medicines from the interactions for cataloging and treatment coding

- Mobile phones and credit – to ensure proper communication between supervisors and SPs
- Strong Internet (industrial plan) – to communicate with the research team with email and Skype, to access the data entry system and to upload forms and download necessary materials
- For paper-based data collection system:
    - 2 high-quality scanners – to scan any paper forms or important documents
    - Printer – to print paper forms or important documents
- Laptops with separate keyboards and mouse – to manage schedules, data processes, communication, and data entry among supervisors
- Tablet for videos – to audio-visually document SPs describe any unusual or interesting encounters in the field
- Stationery – to use for printing paper forms or important documents, as well as sending any materials through post
- Watches, stopwatches for SPs – for SP wear in order to assess waiting time, start time, and end time of each interaction without needing to look at the mobile phone or to ask for the time (though SPs have reported that getting screen grabs or screenshots from their phones is a quick and easy way to capture the correct time in and time out)

Finding providers by asking others in the community

- Depending on the setting of the study, it may be easy or challenging to identify the location of a provider when the address is unknown or imprecise. In urban areas, this is more likely to be easier than in non-urban areas.
- Section 8.1 details the experience of SP projects with respect to challenges in locating providers.

Dealing with unlabeled medicines dispensed during interactions

In some cities, there is a practice of dispensing cut-out sections of medicine blister packs, loose tablets, or syrups in small plastic bags. Given that these are often unlabeled and reflect aspects of the patient-provider interaction that are important to capture, the research will want to classify these appropriately. Below is a step-by-step process that can be used for identifying loose medicines once all interactions have been completed, corresponding medicines have been purchased, photos of all medicines have already been taken and catalogued by

interaction, and the medicine sections of all exit questionnaires for the interactions have been completed:

- *Step A. Selecting help.* Supervisors take out 5-6 medicines from the blister packs (so that the names of the medicines are known) and ask 5-6 pharmacists in different areas and in the wholesale market to identify them. Among those who are able to identify them well, three should be recruited and reimbursed for one day of work.
- *Step B – Identification process.* The pharmacists can be invited to come to the field team's office. Procedures for the field team are:
    - Ask the invited pharmacists to sit in three separate rooms
    - Ask each of them to separately identify the unlabeled medicines and have each pharmacist identify the medicine
    - Record what each pharmacist says.
        - In cases where the pharmacists all identify the unlabeled medicines with the same name, it can be said with some certainty that the medicine is what the pharmacists say they are.
        - In cases where the pharmacists do not agree, no strong claim can be made on what the medicine is.
- *Step C – Checking.* The project team must assess whether the pharmacists can be asked other questions that they may know. For example, pharmacists can be asked for each medicine whether they feel that it could be a certain classification or type of medicine or whether it could be something to remedy cough.

For ongoing refresher training and risk mitigation strategies for long fieldwork:

1. During each month of data collection, the supervisors should hold two meetings with all the SPs to review SP safety and risk mitigation strategies and to provide a formal space to debrief all together the experiences in the field. During these meetings, supervisors should go over instructions for the SPs on how to avoid invasive or potentially unclean examinations (e.g., thermometers) and interventions (e.g., injections), such as avoiding the placement of their arms on the table and always asking the provider what he intends to do if he moves toward the SP for any examination.
2. Additionally, there should be a review once a week during which SPs are asked to describe any situation that arose with regard to invasive procedures and what tactics were used to avoid or refuse such events. SPs should also be reminded in these weekly meetings on exit strategies (e.g., if they need to quickly terminate the clinical

encounter and that rather than risk invasive procedures, they should reveal their identities and give the supervisor's phone number to the provider if they feel that the provider is aggressively pursuing an invasive procedure). Any such instance should be recorded as an adverse event with clear documentation of the circumstances that led to the disclosure. These should be immediately reported to the project team for proper reporting.

SECTION 10. FIELDWORK

This section provides information on SP fieldwork, specifically sampling and scheduling providers, creating a logistical plan, monitoring fieldwork, and the outputs of fieldwork.

10.1 Sampling and scheduling

Theoretical sampling structures range from the ideal (a block-randomized or stratified draw from a fixed sample universe) to the practical (probability-proportional-to-size sampling with reweighting when it is impossible to visit each location more than once). Research questions and field practicalities may also induce the research team to develop more complicated sampling schema: it may be desired to visit individual providers at two separate locations of practice or two different providers at the same location, for example. Overlapping and interlocking samples may arise, and careful recordkeeping should maximally record information that affects this stage of decision-making, as it will be crucial to answer the original research questions and inform analysis after all SP interactions are completed. One of the biggest priorities throughout fieldwork will always be to ensure minimized levels of SP risk detection. Thus, if overlapping and interlocking samples arise, the research and field teams must work closely to ensure that the same SP actor is not repeatedly visiting the same doctor within a small amount of time. This is discussed in the next couple of paragraphs.

From the research analyst's point of view, the final sample should be set with the appropriate reproducible sampling strategy (e.g., when using random selection in STATA software (College Station, TX), setting the seed and safekeeping the seed number will ensure reproducibility). The sampling strategy should output a master code file of eligible, ineligible, selected, and non-selected providers for each sampling scheme, which will each be necessary for applying correct analytical weights; assessing loss to follow-up and attrition; and other statistical tasks for which the status of the whole sampling frame is required, as well as for the completion of mandatory reporting tools, such as the CONSORT flow diagram or STROBE Statement checklists. The "selected" subset will then be used in the schedule assignment for actual cases.

The scheduling process must be carefully planned in advance of the final sample draw, so that draft schedules can be reviewed for problems and the programming can undergo quality checks before locking the randomization seed and protocol. Particularly when the sampling

plan is to have each facility receive multiple SP cases and many interactions, the scheduling process may become intricate to avoid assigning too many or too few interactions of each type to a given facility; and if there are second randomizations or micro-experiments within SP cases (morning/evening visits, medical artifacts, specially designed tweaks to case scripts, gender, and the like), it will be important to restrict the number of visits of each type a given facility receives (even if there are multiple providers of interest within a single location) to avoid arousing suspicion. This is especially true if the cases to be presented are highly characteristic or uncommon, or if the facility in question has a low background caseload.

If a study has multiple cases or case variants, scheduling will typically involve randomized assignment of cases to facilities or providers in multiple rounds. Again, it is crucial that the final schedule be set with the appropriate reproducible sampling command. This will typically involve repeated draws from the final sample, appropriately stratified, with the first output in extended (wide-format) version of the sample for data records. This will also have the full case schedule for each provider or facility listed out column-wise. The second output will be an interaction-wise (long-format) schedule, sorted by facility code, for transmission to the field team. This spreadsheet should also have affixed a tracking sheet with the basic information about each provider and appropriate fields for the field team to report why interactions are incomplete and any new information acquired in the field. (See Annex Q3 for the wide schedule output and the long tracking output, further discussed in Section 11.)

The assignment of individual SPs within their respective case interactions typically need not be set randomly, as the convenience and efficiency conditions of active fieldwork are typically sufficiently random so as to leave the characteristics of the visited facilities and providers uncorrelated with any idiosyncratic effect of a given SP identity. Of course, a single SP cannot be tasked to cover single geographic regions or sample subsets day after day, and this restriction should be communicated to the field management. These constraints will rarely pose a problem in practice in all but the largest and most difficult urban environments where transportation access and spatial clustering are a serious concern.

10.2 Logistical plan for fieldwork

Field teams can arrange the fieldwork logistical plan to fit the priorities and responsibilities for the project. For consideration, one logistical plan generalized across previous SP studies is the following. Fieldwork can be assigned from Monday to Friday or Saturday, in accordance with

local working schedules. In previous studies, each SP can attempt on average two interactions per day because of the extensive travel, waiting, and recording time inherent to conducting realistic facility visits. Saturdays can be reserved for communication, management queries, etc., and Sunday will be a rest day for all staff (unless a different day of week is traditional). Supervision checks of paper-based questionnaires, scanning, picture taking, and uploading will likely happen twice a week. This section includes daily, debriefing, and nightly checklists for supervisors.

Daily Checklist for Supervisors

Supervisor daily prep meetings

- Review daily assignments of SPs and (ii) agree as a group on SP allocation for time of day (morning, afternoon, and evening) based on health facility characteristics and schedule. The opening hours of facilities vary widely and are often advertised, with specialist doctors typically having quite limited consultation hours at each facility, so these should be scouted to the best of the team's ability before fielding SPs.
- Prepare and sign-out items for SP use:
    - Sign out audio devices with quality checks for storage space and battery charging
    - Sign out bags for SPs to carry audio devices
    - Check accuracy and function of timing and communication devices (cell phone, watch, battery charge) as these will be used to coordinate field meetings as well as record interaction details
    - Prepare pen and writing pads for interaction notes upon exit
    - Set up money to pay for transport and provider visit and medicines
    - Debrief or provide instructions on how to arrive at the health facility and see any scheduled provider
- Prepare for supervisor debriefs in supervision zones
    - Set up printed exit questionnaires and back-up copies (or electronic versions)
    - Check pens and notebooks
    - Ensure bags and ID sheets for medicines are ready
    - Check tablet or cell phone for GPS-tagged photos if desired or GPS device
- Double checks before going to the field
    - Make sure health facility is not listed on SP recognition excel sheet for each SP – that is, if they had visited the location before

- ○ Confirm health facility appropriateness for case and time of day – specialization, consultation hours, or appointment requirement
- ○ Confirm health facility appropriateness for SP visit and patient load – it may be discovered that a scouted location is primarily pediatric, for example, or that it has introduced inpatient triage, which may render it unacceptably risky

Communication with SPs before field visits

- Time of visit
- Directions and map of area
- Zone characteristics for SP character
- Health facility basic characteristics – facility name, provider name, name of compounder or assistant or male counterpart, street or address, neighborhood, landmarks, transit and walking directions, detailed instructions on how to arrive at clinic
- Location of debrief site – make sure this is very clear, potentially even visiting the site with SPs before interactions begin
- Review each SP's schedule for the day with them – what zones they will be working in and what supervisors they will be reporting to from each location
- Confirm SPs have correct phone numbers and contact details for supervisors, and that the supervisors' phones are on and charged

Confirm field-debriefing site for day

- Not within viewing distance of any health facility from sample
- Central location for SPs to visit without additional travel time or inconvenience
- Review transportation options to/from health facilities and debriefing site with SPs
- Plot on maps (draw for each zone)

Conduct SP exit interviews

- See questionnaire manual for detailed instructions on conducting the SP exit questionnaires
- Interviews should be conducted within 1-2 hours of SP interaction
- Check audios are filed correctly on devices
- Other protocols

Collect and review medicines, prescriptions, documentation, and other artifacts from SP interactions

- Fill out ID slips/tags and bags for collections from each interaction
- Take photos for all the artifacts of each interaction with corresponding ID slip/tag. If all the artifacts have not been collected (e.g., the interaction involved a prescription and

those medicines have not been purchased, then it is better to wait until all the medicines have been purchased before the photo is taken)

- Bag all the artifacts with the corresponding ID slip/tag for each interaction and store the bags for reference at any later time during data collection

Post-Debrief Daily Protocols

Prescription follow-ups and chemist visits

- SPs who received prescriptions rather than having medications dispensed directly to them at the point of service should visit the pharmacy recommended by the provider or other nearby pharmacist and ascertain the prices of all medicines prescribed.

Hand-off medicines and prescription information to drug quality testing team

- All medications obtained from providers and pharmacies should be retained when the study protocol called for it, and in cases where the content or quality of medication is to be tested (e.g., mass spectrometry), safely labeled and stored before being transferred to the appropriate personnel.

Nightly Data Checklist for Supervisors

- Prepare for next day's SP interactions:
    - Check, allot memory, and recharge batteries of audio, video, or mobile devices
    - Organize the SP bags for audio devices
    - Count returned timing devices and make sure they are functional and charged (cell phones, watches)
    - Replace used pens and notebooks
- Prepare next day's items for supervisors
    - Print exit questionnaires and back-up copies
    - Replace used pens and notebooks
    - Bags and ID sheets for medicines
    - Charge tablets, clear memory space, and back up photos and other recordings

10.3 Monitoring fieldwork

Field supervisors should dispatch daily updates to the project team. This should include a brief summary of any confusion or questions that came up during the day's fieldwork for the project team to respond to. The daily update should also provide a list of the interactions that were attempted by each SP and the reasons given for incomplete interactions, in case systematic

problems arise and urgent changes to the field protocol need to be made related to emerging field issues.

If there are new cases, experiments, or other activities going to field, daily, unstructured commentary on these protocols may also be requested by the project team even when no problems or challenges have arisen.

On the data side, the analyst should be able to track the progress of interactions against the schedule from the field team's manual tracking list, as well as against the data progress available from the digital data system. This will allow conflicting records and data issues to be pushed back in real time to data and field teams so that corrections, updates, and clarifications can be made before surveys are archives and interactions forgotten.

10.4 Fieldwork outputs

Fieldwork has several key outputs. First, there is the raw questionnaire data itself – both the physical or digital first copies, as well as the final entered data for analysis. It also produces images of prescription slips and medications for further investigation. Fieldwork may also produce updated records of provider addresses, including GPS coordinates, improved directions, and other mapping or spatial analysis inputs.

Additional options for capturing information on the SP-provider interaction that may be relevant for the study include: video or audio recordings of SPs narrating their interactions and SP or supervisor comments for each interaction. For example, in the Qutub project, interactions that were unique or interesting were identified by the supervisors, and either on the same or following day, these SPs sat down in front of a camera and narrated their experience with the guidance of the supervisors. These videos were then available for the research team to discuss. In the KePSIE project, after each interaction, SPs used audio recorders to record their narration of the interaction experience. These audios were then transcribed and incorporated into a single document for use study team. Annex P contains selected interaction narrations from the KePSIE study.

Finally, the fieldwork-tracking sheet produces the final inputs for the completed master file, recording the final outcome and status of every provider scheduled for SP interactions so that appropriate analysis on attrition and migration can be performed.

SECTION 11. DATA ENTRY, PROGRAMMING & ANALYSIS

This section provides details on: data files, interaction-scoring elements (history checklist and treatment grading), approaches for electronic data capture, setting up systems for data verification and data quality checks, data management and analysis, incorporating secondary data sources, and ensuring interoperability in case multiple partners work with providers.

11.1 Data files

Based on previous projects, SP data analysis requires a minimum of five master repositories of non-SP interaction data and one master data dictionary to support the SP interaction data. Annex Q contains templates for these six files:

1. Provider universe master code file (Annex Q1)
2. Sample master code file (Annex Q2)
3. Schedule and tracking master code file (Annex Q3)
4. SP staff master code file (Annex Q4)
5. Medicines master code file (Annex Q5)
6. Exit questionnaire master data dictionary file (Annex Q6)

The first four of these files can be completed by the start of fieldwork, the fifth can be compiled only after commencement of data collection, and the last file can be compiled once the exit questionnaires are developed. All files are described in detail below.

1. Provider universe master code file(s)

When sampling providers for interactions, it is fairly common to select a subset of providers from a list called the provider universe or provider census list (i.e., the entire population of health care providers if that is the unit of interest). In SP studies, it is crucial to properly maintain a master code file of all providers from which the sample was selected. This code file, at minimum, should have unique IDs; basic identifying and location data; and information on criteria that will be used for selection eligibility or ineligibility. This is essential for several reasons: scheduling SPs, sending SPs to specific locations to find sampled providers, ascertaining attrition rates, and producing appropriate analysis weights either ex ante or ex post. The main project analyst should manage these data, since returning to the file from which the sample was selected will be used at several stages during fieldwork and analysis. It is also essential to archive "frozen" versions of these data at the time of sampling in case changes are made to the sampling universe throughout the course of the project.

Managing this file is not trivial, because providers may move locations, providers can practice at multiple locations, and any given location (e.g., a health facility) can have multiple providers. Thus, multiple "provider universe" files may be needed, for example, when sampling is conducted at the facility/location level. In this case, it will be required to have one file retaining the locations and characteristics of facilities listed in the mapping or census exercises with a separate type of unique ID nomenclature (e.g., a facility ID), as well as a list of providers (e.g., with provider IDs) indicating at which locations they may be found (e.g., facility-provider ID), particularly when there are several providers within each facility or providers practicing across multiple locations. In summary, it is ideal if individual providers have a unique ID from an ID system independent of the facility ID, as the relationships are likely to be complex and evolving. This data structure allows facility characteristics to be easily linked to provider characteristics in the appropriate setting, as well as for both facilities and providers to be correctly matched to SP exit questionnaires with appropriate recording of the interaction details.

2. Sample master code file

Once the sample is drawn from the provider census list ("the universe") and the interaction schedule is set for the field team, a file recording the selected sample should be saved for analysis. This file should not include the case interactions assigned to each facility or provider. Instead, it will record information such as the sample stratification the provider falls into during each wave, whether or not visits could be completed in each wave, the reason for incomplete visits, and other notes that will support final analysis and write-up. It should also maintain linkages between the anonymized study IDs and any other identifiers that will link those observations to non-SP data. This should be consistently maintained and expanded upon throughout fieldwork.

3. Schedule and tracking master code file

Once the sample is drawn from the universe and the interaction schedule is set, a file recording the selected sample and assigned interaction schedule should be frozen and saved for analysis. This file will include two sheets: a "wide-format" sampling schedule and a "long-format fieldwork tracking" schedule. The wide schedule will include the cases assigned to each facility or provider, the basic information used for stratification or block-randomization, weights resulting from the sampling strategy, when appropriate, and the essential fieldwork location and identification information. It should retain unique provider, facility, supervisor, and SP IDs as

necessary to match back to the provider universe files, as well as to the SP data and any other external data sources. The "fieldwork tracking" schedule will be used throughout data collection to track progress of interactions. It contains unique form IDs pertaining to each scheduled interaction and the associated provider-case pairing, as well as details on completed and incomplete visits. Visits may be deemed incomplete if the doctor was temporarily unavailable or refused to see the SP, if the SP waited for 3 hours, etc. This file will be constantly updated throughout fieldwork, and will also be useful at the finalization stage to wrap up unfinished observations, assess attrition and loss to follow-up, and run quality checks on data entry and data completeness.

4. SP staff master code file

This file is the master staff roster, containing unique SP IDs and supervisor IDs, which should be entered on each exit questionnaire. The staff roster should report gender, height and weight, blood pressure, age and date of birth, city of origin, tribe/caste/etc. where appropriate, religion where appropriate (as visual or other social markers such as headgear or other body decoration may be relevant), and existing vitals and health conditions identified on the health screening questionnaires (see Annex H) of all SPs participating in the fieldwork.

5. Medicine master code files

As field data are entered and made available to the research team, the project analyst must maintain a master code file of medicine data. Since medicines being prescribed and dispensed in the SP interactions will be entered by both brand name and generic components (when identified), it is ideal to construct two spreadsheets or files containing key information about medicines.

The first sheet should be a linking file indicating, for every written medicine name, the cleaned set of generic components corresponding to the drug. The first column will record the drug name as recorded in the data, and the remaining columns (which will be unbounded in number but typically capped at about five generics per drug) will record the standardized generic names. The field-recorded names will vary widely, even among the same underlying medication, due to variance in drug labeling, field recording, transcription, data entry, capitalization, punctuation, abbreviation, typographical error, decipherability of provider's handwriting, and other factors. Accurately identifying the generic components of each given medication is essential, since these will be used for the actual treatment grading. This master

sheet may continue to expand as new field observations are recorded. In the Qutub project, for instance, this process resulted in approximately one new unique item recorded for every two interactions. Unless there is preventative strategy, the recorded names should never be cleaned, as they will be the variable used for linking the raw data to the second cleaned generic list. In some instances, particularly when language is a barrier, the ATC code can be recorded in addition to the written name of the medication, since these uniquely link to a set of generic ingredients. The use of the ATC code field takes some additional training for the enumerators, since they will need to have a codebook of common medications and the information to look up new ones (generally, in English).

The second sheet records the characteristics of each cleanly identified generic medication and is likely to reach about 100 unique generics in a large-scale project, from Aspirin to Zinc. Column characteristics will include the compound classification (antibiotic, steroid, psychoactive, narcotic, etc.), the legal classification (over-the-counter, restricted, illegal, etc.), and the suitability of the given compound to each condition of interest (appropriate, unnecessary, contraindicated, etc.), among other project-specific characteristics of interest (e.g., subsidized good or free treatment through a policy or program). The primary advantage of this approach is that the generic file can then be merged directly with the interaction data to provide an indicator of whether each SP received any (and if so, how many) of each generic compound type, which proves indispensable when combination drugs and redundant drugs are common practice in contexts demonstrating high rates of dispensing more than one type of medicine.

6. Exit questionnaire master data dictionary file

The master data dictionary file contains all the variable names and variable labels by case. This file smoothens the data workflow across analysts, data entry operators or data managers, and members of the data firm, and this workflow produces the final SP interaction data set that can be used for analysis. Depending on the exit questionnaires corresponding to each SP case and the structure of the exit questionnaires, there will be some variables that are consistent across cases, and there will be other variables that are case-specific.

11.2 Checklist and treatment grading

This section details the process for scoring the interaction. It is important to note that the development of the guidelines is done by the Technical Advisory Group members, and the treatment coding and grading process for each interaction is conducted by experts who are

selected to look at the medicines. These are two separate processes, but the latter group can be made of members selected from the former group.

Individuals from the project team and Technical Advisory Group should pre-select the history questions that are essential diagnostic checklist components for each case. These should not be indicated on the questionnaire but recorded for later diagnostic calculation. The "checklist" in this case will conceptually correspond with how far along the appropriate diagnostic questioning line the provider used for the SP case of interest. In data analysis, the checklist percentage will be the proportion of the essential questions asked, calculated by a simple average of the binary responses to those items.

One key component for validating the SP methodology is that greater checklist completion for each case should correlate strongly with the correctness of the treatment; that is, that appropriate inquiries should lead to an appropriate clinical conclusion about the SP. Treatment, however, has many dimensions. It first includes positive behaviors, that is, whether a clinically appropriate medicine, referral, or other recommendation has been given. It also includes neutral or negative behaviors, such as the provision of additional symptomatic treatments or the provision of unnecessary drugs like antibiotics or contraindicated medications. Each of these behaviors can and should be coded and considered separately by the data analyst, and in most cases, they do not permit a one-dimensional treatment-grading rubric.

In order to achieve a validation strategy, a joint holistic grading of these behaviors can be carried out in addition and complementary to the binary behavioral analysis. To achieve this, a panel of experts, such as those selected from the Technical Advisory Group, can each be independently presented with the whole treatment behavior set (medication given, tests ordered, and referral or follow-up orders) for each SP interaction. These experts should receive no further information other than the condition being treated. One example of how they can "score" or "grade" the interactions is the following. They can be asked to assess each case's treatment on a holistic Likert scale, such as: 1=Harmful, 2=Inadequate, 3=Adequate, 4=Exceptional, and 5=Ideal. This allows the consideration of medically unnecessary palliatives, interplay between both appropriate and contraindicated drugs, and other harmful drug interactions or complementarities, which would otherwise not be identified in the typical, non-expert analysis. They may also submit notes or comments on each treatment set to provide further insights to

the analysis team. Box 11.2.1 offers a guide for the Technical Advisory Group experts who will participate in treatment grading.

Box 11.2.1. Guide for treatment grading

1. Review exit questionnaires
- It is critical that the Technical Advisory Group members, consultants, or experts who participate in treatment grading (the treatment graders) review blank versions of the exit questionnaires related to the SP project of concern. Each SP project often tends to have various SP cases, which can either represent different conditions or the same condition in different stages of progression.

2. Undergo training for coding procedure (e.g., in Microsoft Excel) or review protocols for SP Data Entry System (SPDES)
- The experts ideally should be trained in coding procedures or proper data entry for the treatment grading. The analyst who will compile and merge the treatment coding data with the other data generated from SP fieldwork can conduct training.
- If coding is done on an electronic platform, such as in an SPDES, the data entry workflow, including interaction scheduling and tracking, questionnaire completion, digitization and upload, data receipt and entry, download, and analysis, can be mocked up in a slideshow for review prior to implementation.

3. Review all the interactions under the same SP case
- The experts should independently review interactions. Each expert should review all the interactions under the same SP case at the same time.
- If an SPDES exists, the experts assigned to treatment review will be able to access their task load (for example, holistically grading treatment appropriateness) by logging into a special account in the SPDES interface. They will see the details of the interactions that they are assigned and the available actions or grades that can be taken on each interaction; new actions will be saved automatically and their outputs added to the final downloadable data file seamlessly.

11.3 Electronic data capture approach

Carrying out some parts of the schedule assignment, SP data entry, and secondary data collection using a digital data management interface can be advantageous, as it can support a well-managed workflow rather than leaving a long paper trail of dated spreadsheets and cross-team communications. Especially when revisions to primary data are concerned, a final raw dataset can incorporate the inputs of other team members before being shipped to the analyst. This serves to avoid complex workflows involving corrections, additions, and other supplements to the questionnaire at the point of analysis. This section will walk through the aspects to consider when digitizing the various input processes.

The metadata and supplementary files needed for the back-end of the digital data user interface are:

- Sample master code file (Annex Q2)
    - (Note: including the provider universe master file (Annex Q1) is not necessary if the sample master code file contains information such as address)
- Schedule and tracking master code file (Annex Q3)
- Staff (supervisor and SP) master code file (Annex Q4)
- Medication generic master list (Annex Q5)
- Data dictionary for exit questionnaires (Annex Q6)

Sampling and scheduling (Users: Data collectors and/or supervisors)

A data entry system that has the correct sample preloaded restricts the field team to providing responses only for interactions that have actually been scheduled, as opposed to unstructured systems where duplicate or missed interactions are more likely to occur. Sometimes more manual processes can allow for better follow-up and tracking in the field. For example, because the identifying information of the sample unit is displayed and the required interaction also indicated, the field team can track their progress towards completion easily and know exactly where each data point is to be entered. In addition, if replacement samples are available for the field team to draw upon, preloading this data and requiring the team to actively drop and replace a scheduled interaction in the interface leaves a record of which interactions were not completed, the reason for non-completion, and the replacement if applicable.

Survey tool for exit interviews in the field (Users: Data collectors and/or supervisors)

Kwan A, *et al. BMJ Global Health* 2019; 4:e001669. doi: 10.1136/bmjgh-2019-001669

Using Standardized Patients to Measure Health Care Quality

As the interactions are completed, whether they are done on paper or digitally, supervisors should check each returned survey and supplementary data (e.g., paper prescriptions, images of medicines) and then upload them to the digital platform. Figure 11.3.1 shows a demonstration of the data workflow for the Qutub project. This should produce a "raw copy" of the field team's input that can be compared against the SP data later. In this way, conflicts across raw, data-entered, and downloaded data can be resolved by quickly looking up interactions in question and tracing problems, missing data, or inconsistencies to the original source.

Figure 11.3.1. Example anonymized workflow for digital design, manual fieldwork, and digital data entry from the Qutub project

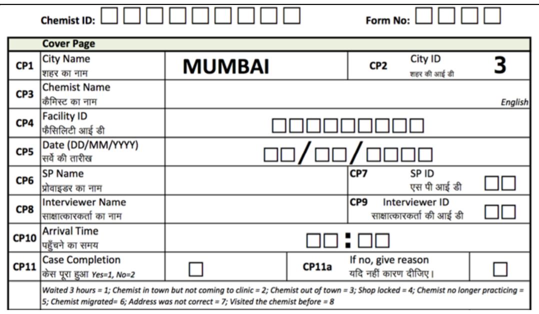

1. The standardized survey form is developed with labeled and coded response items.

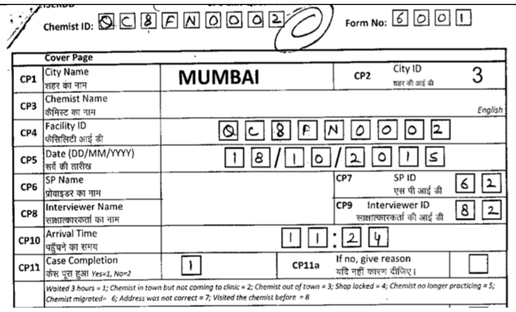

2. Forms are filled in the field, scanned, named, and sent to data entry technicians.

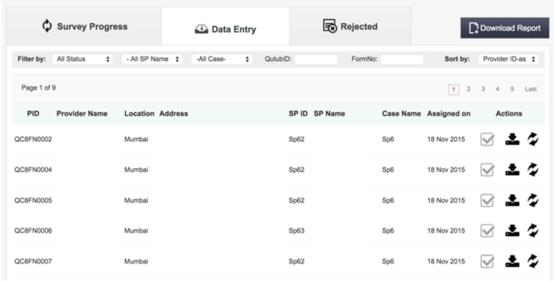

3. Data entry operators enter data for every scheduled survey interaction. Original scans are accessible by various searches and filters.

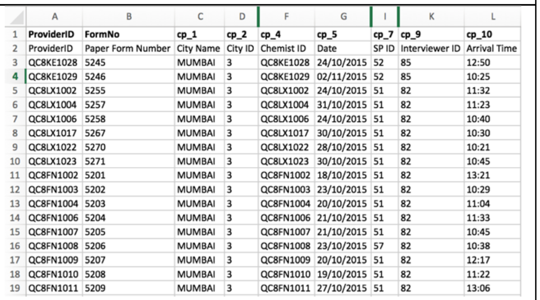

	A	B	C	D	F	G	I	K	L
1	ProviderID	FormNo	cp_1	cp_2	cp_4	cp_5	cp_7	cp_9	cp_10
2	ProviderID	Paper Form Number	City Name	City ID	Chemist ID	Date	SP ID	Interviewer ID	Arrival Time
3	QC8KE1028	5245	MUMBAI	3	QC8KE1028	24/10/2015	52	85	12:50
4	QC8KE1029	5246	MUMBAI	3	QC8KE1029	02/11/2015	52	85	10:25
5	QC8LX1002	5255	MUMBAI	3	QC8LX1002	24/10/2015	51	82	11:32
6	QC8LX1004	5257	MUMBAI	3	QC8LX1004	31/10/2015	51	82	11:23
7	QC8LX1006	5258	MUMBAI	3	QC8LX1006	24/10/2015	51	82	10:40
8	QC8LX1017	5267	MUMBAI	3	QC8LX1017	30/10/2015	51	82	10:30
9	QC8LX1022	5270	MUMBAI	3	QC8LX1022	28/10/2015	51	82	10:21
10	QC8LX1023	5271	MUMBAI	3	QC8LX1023	30/10/2015	51	82	10:45
11	QC8FN1002	5201	MUMBAI	3	QC8FN1002	18/10/2015	51	82	13:21
12	QC8FN1003	5202	MUMBAI	3	QC8FN1003	23/10/2015	51	82	10:29
13	QC8FN1004	5203	MUMBAI	3	QC8FN1004	20/10/2015	51	82	11:04
14	QC8FN1006	5204	MUMBAI	3	QC8FN1006	21/10/2015	51	82	11:33
15	QC8FN1007	5205	MUMBAI	3	QC8FN1007	21/10/2015	51	82	10:45
16	QC8FN1008	5206	MUMBAI	3	QC8FN1008	23/10/2015	57	82	10:38
17	QC8FN1009	5207	MUMBAI	3	QC8FN1009	20/10/2015	51	82	12:17
18	QC8FN1010	5208	MUMBAI	3	QC8FN1010	19/10/2015	51	82	11:22
19	QC8FN1011	5209	MUMBAI	3	QC8FN1011	27/10/2015	51	82	13:06

4. The long-form data are combined into a single .csv file, including all ID information needed to find the paper scan and master code file record.

Data entry (Users: supervisors and data manager, respectively)

If scans of paper questionnaires or other non-digital methods of data capture are recorded, or if open-ended questions need to be coded, it can be advantageous to have a team separate from the survey staff be responsible for the digitization of information. For example, during fieldwork, the Qutub project used paper exit questionnaires, from which scans were uploaded to the digital data system. The system was designed to retain a hard copy of the written questionnaire for reference by field or analysis teams. A separate team was responsible for reading the scans and entering the data into a second interface, which was designed to resemble the survey and allow easy digitization of the data. It would be theoretically possible to have the field team also complete this task, but for a large-scale study, it is more efficient to have the field team focus on fieldwork and to have a data entry team focus on getting the data in order. However, this only works well if there are correct checks, proper organization, operational feedback loops, and strong communication and collaboration in place. Finally, separate data entry provides a quality check and can flag issues that were overlooked at first recording.

Medicine or treatment coding (User: expert panel)

Once the relevant fields are determined for medicines prescribed or dispensed during the SP encounters, a simple interface can be developed allowing the experts conducting treatment grading to log in and begin the grading process for interactions one at a time. A basic screen can display the relevant information for any expert participating in the treatment coding process, and while treatment coders records their inputs, the interface can display their progress, assuring them that their responses are accepted and coded in the correct format, without hassling with outside software or file exchanges.

Data quality checking (User: data manager)

Usually, quality checks including missing values checks, consistency checks between mutually exclusive questions, proper filling-out of conditional questions, extreme values checks, and the like can be automated in a simple system, since the values are accessible to the background software. These interactions can be flagged and automatically sent to the field team and/or the data entry team for review and resolution (such as the case of the 100-year old child who has simply received an additional zero). Additionally, the project team should conduct hands-on checks that can strengthen communication among the team members, help identify issues that may not be caught by a digital system, and provide further avenues to understand the

context and dynamics of the setting, which may be relevant for interpreting the results of the analyses.

Monitoring (User: administrator)

Metadata generated from the survey tool usage should be recorded and reported to the analyst. This includes a progress report showing completed and outstanding scheduled interactions, as well as dropped or replaced interactions along with the reasoning and other comments from the field team about other cases. Other metadata reports can also be generated, such as a provider- or facility-level sheet that can link directly to the master code file, in contrast to the interaction-level reports, which will have several rows for each facility and/or provider. Figure 11.3.2 shows the flow of data collection and data checks that this section assumes.

Figure 11.3.2. Data entry flow diagram edited from the Qutub project.

Note: SPDES refers to the SP data entry system; QoC refers to quality of care.

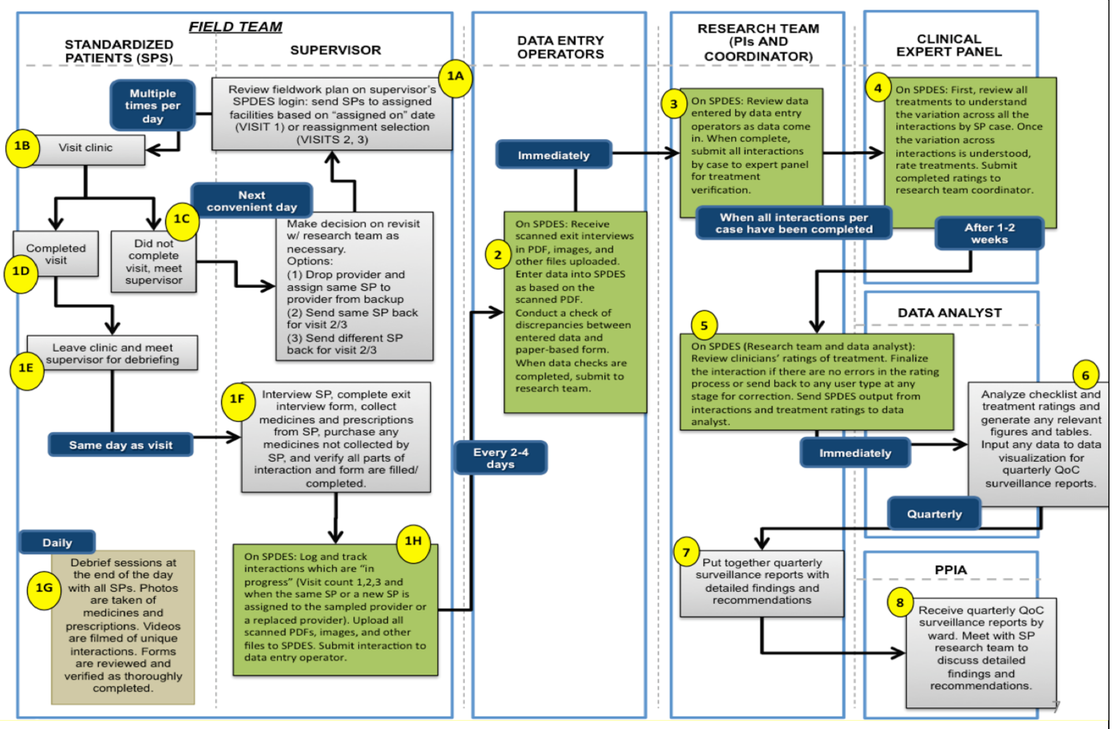

Below are descriptions of what to consider for each aspect listed above.

1. Sampling (Users: Data collectors and/or supervisors)
2. Survey tool for exit interviews in the field (Users: Data collectors and/or supervisors)
3. Data upload and data entry (Users: supervisors and data manager, respectively)
4. Treatment coding (User: expert panel)
5. Provider, supervisor, and SP confidentiality – When coding the treatments received by the SPs, treatment coders should not receive any data that contain identifiable characteristics for providers, supervisors, or SPs. The reason for this is primarily twofold: (1) to protect participants and maintain their anonymity, and (2) to eliminate any coding bias.
6. Data quality (User: data manager)
7. Monitoring (User: administrator)

11.4 System for verification and quality checks

To ensure a smooth analytical process on data gathered from a provider visit, within range and other acceptable values should be strictly defined for every questionnaire item or variable, and the conditionality allowing missing values should also be well defined. Upon receiving the data, the analyst should conduct a high-level verification by eye of the raw data to ensure there are no egregious systematic errors, such as the swapping of adjacent data fields or the receipt of nonsensical information throughout an entire variable.

Then, based on the Qutub study experience, a "strict" verification system is recommended that will exclude any data points not matching the expected input format. For example, in a "Yes or No" question, which has responses coded "1" for "Yes" and "2" for "No" in the questionnaires, the data should expect a value of "1" or "2", and a value of "3" ought to be rejected and flagged for the data entry operator. In the case that the issue was due to a poor scan or illegible handwriting, the data entry team would return the form back to the field team for re-scanning or clarification. Similarly, a field expecting a numerical value ought to be flagged for review even if "harmless" formatting such as "20/-" (a common expression of a rounded price in India) is utilized where a numerical input ("20") is expected. Simply stripping text strings is inappropriate as "2.00" and "2,00" may both mean "two dollars" but could be interpreted as "2" and "200" or even "2,000", respectively, if decimals are accepted but commas are stripped from numerical fields or if commas are interpreted as marking the thousands place.

In the second stage, all unexpected missing values should be listed and returned to the data entry team for verification. This means that the logical structure of the questionnaire should be programmed to flag any required field that is not filled, as well as any conditional (filtered) field whose conditionality (filter) has been triggered. The final list of missing observations to be returned to the data entry team will then include both true missing values and values that were erroneously entered and filtered out during the strict verification. As previously mentioned, the data entry team can verify these against the original data, and when the problem has occurred on the original questionnaire, it can then be referred back to the field team for correction. Corrections should at last be re-entered in the digital data system by the original team when possible to avoid a long collection of line-item corrections accumulating in the analyst's programming.

11.5 Data management and analysis

Data management and analysis should follow a well-defined workflow that prevents unnecessary redundancy or confusion, and given recent trends in research towards transparency, data analysis should also maintain procedures that allow for easy replication – beginning with data construction from the raw data to the analysis dataset. Raw long-form data, coming directly from the data entry teams, should always be maintained in an unedited state in the most complete version. If a segmented data entry workflow is used (as in the case of daily filings by data entry operators), then every filed item should be maintained as unedited. If software is used to automatically add new records to a unified dataset, then the most recent version of that database should be kept, unedited and on hand. These should be stored in folders within a backed-up file system.

To reflect the changing forms of the SP data from its raw form to its analysis form, the main "data" folder should be managed by the project analyst and have the following basic components:

1. **Raw data.** Raw data should maintain a record of the data exactly as it is delivered to the data analyst without any processing or edits, so that original records can always be reviewed by the manager or by other analytical teams. This includes documentation of the survey systems that generated the data, instructions given to enumerators, entry codes, and electronic scans of all original materials, including completed forms.

2. **Metadata.** Metadata files will be essential to processing data automatically. They may include machine-readable resources for completing data preparation tasks. Some examples are manually generated lists matching IDs across various data sources; information on the legal status of various medications, which can be merged onto treatment files; and variable naming and labeling codebooks or data dictionaries (e.g., the Annex Q6) that direct the import process for raw datasets.
3. **Processed data.** Since raw datasets will rarely be in a useable file format for statistical computing, they will need to be imported and cleaned into a "processed" state that is neither the raw data nor the final data for analysis tasks. Processed datasets should reflect "mundane" data changes, such as corrections to typographical errors on raw data and the generation of standardized date and time codes from written information. They will also include reshaped versions of long-format datasets or compiled versions of dynamic databases.
4. **Analysis data.** Datasets for analysis are those used to conduct analyses. They can include merged combinations of processed datasets or reflect the addition of new variables, which are derived from raw data inputs. They should never be derived from raw data nor recombined with each other at later stages.

11.6 Incorporating secondary data sources

Secondary data sources, such as linked administrative data for health facilities or providers in the SP study, GIS records, geographic data, demographic information, government census records, treatment randomization records, sample weights, ethnographic details, and so on may be available for linking with SP results. However, it is rarely appropriate to integrate these data sources directly into SP data directly, but instead these should be stored as raw (secondary) data in their own right, imported into the processed data folder, and then merged into analysis datasets as appropriate during the construction of those files.

The key constraint is the management of data obtained from external sources. If data is provided periodically rather than as cross-sectional data, it will be important to maintain version control or a dated version history, since the providing group may not be keeping diligent records. That is, external collaborators may be updating and overwriting their files without saving historical snapshots, meaning that questions about past versions will require the analyst to provide the agency with the old snapshot of their own data. As much information as possible should be recorded about every file, particularly when there are modifications, merges, and

relationships between the various files that the analyst has discovered or that the data source has communicated.

Furthermore, external data sources may be inconsistent over time, either in structure or content. It is crucial to structure the data so that duplicate, overlapping, or complementary information is flagged, that linking files between various datasets are available, and that the final linking key to the SP data is always apparent for future analysts.

11.7 Data interoperability across multiple partners

When sampling universes or other data sources are developed in cooperation with partners outside the research team, additional methodologies and safeguards will need to be taken with the ultimate goal of having high levels of data quality for analyzing the SP data.

There are several problems that may afflict any research design where a key database is held in part by an outside partner, including de-synchronization of the research and partner versions of the dataset and non-anonymous storage of sensitive records. To handle these problems, the analyst should archive and annotate every piece of data received from the partner. These should be dated and documented, including linking identifying information to other partner records as well as to research records. In all cases, it will be essential to maintain a master code file that links every record used in the research team's work to a single record on the partner's side. These may also be dated or versioned files to reflect continuing updates to the dataset.

For example, consider a frozen "complete" listing of a health provider universe furnished by a local NGO partner. This database may or may not have unique IDs. Consider the extreme case where there are no unique identifiers present as well as the intermediate case where multiple records have the same ID because one individual can be found at multiple locations. In both cases, the research analyst will have to work with the partner to develop and implement an ID nomenclature that allows the partner to track individual records. The project analyst will also have to develop and implement a separate internal ID nomenclature so that individual records from the research implementation can be shared with outside sources, including the partner, without compromising the anonymity of the individual research subject.

The research team will almost certainly have to update this universe list at a later date in coordination with any partners. At that point, various configurations could be received from the field partner. One likely version is a new instance of the same database "frozen" at a different point in time. This version may bear little to no resemblance to the original in terms of recorded content, particularly if the partner's operational needs have changed. Records may be deleted without a trace from updated listings (e.g., say a provider "withdraws" from the treatment group in an impact evaluation design); records added to some providers may not appear in the universe listing; records for some providers may not have IDs matching them to individual records on the universe listing, or they may match to multiple records. The original database may not be archived on the partner side or be unrecoverable at this point, so comparison with the archived version of the original data file can be possible on the research side only if such a safeguard is put in place by the project analyst.

While the range of potential challenges prevents a comprehensive listing of steps needed to avoid them, the general recommendation is for the research analyst to err on the side of over-documentation and over-archiving of datasets and data-relevant communications received from any research partners. In addition, while manual data review is not typically the best way to work with large datasets, when there are idiosyncratic issues with furnished data, a manual review of substantial sections of partner-furnished data may be essential to ensuring the ongoing compatibility of databases.

SECTION 12. DISSEMINATION OF RESULTS

Throughout the course of the project, a variety of stakeholders will become the audience for this work, and a variety of arenas will be available for disseminating and presenting the results. Although the stakeholders and arenas are similar to those in other international projects or health projects, this section serves to identify aspects of dissemination that may warrant care because of the sensitive topic of quality of care. The following is a list of the different arenas to disseminate the results of SP work.

- Closed-door meetings – Particularly when the SP method informs an intervention implemented by a partner or a donor or project sponsor, it is polite to request a closed-door meeting with these partners to discuss the findings.
- Stakeholder meetings – Holding meetings to inform stakeholders, such as donors, representatives from professional associations, and government, will keep the research team up to date on environmental elements that can influence the interpretation of the results and the application of the results.
- Other – Various other options can provide a fitting place to disseminate SP study results.
    - Policy reports
    - Rapid results briefs
    - Journal publications
    - Conference posters and presentations
    - Conferences
    - Workshops

For additional resources, see https://www.qutubproject.org/

SECTION 13. CONCLUSION

This project manual and toolkit contains many comprehensive elements for determining the feasibility and implementing an SP study in low- and middle-income settings. The purpose of constructing such a manual and toolkit is to continue improving quality of health care around the world through ensuring that the SP methodology is implemented with fidelity, building on lessons from previous projects, and evolves as an ethical and appropriate methodology.

GLOSSARY

Detection rate	The percentage of SPs who were detected as not being a normal patient. This equals the number of correct SPs who completed a case that were detected divided by the number of SPs who completed a case. The numerator can be known by implementing a detection survey at least two weeks after all the cases have been completed.
Detection survey	Following SP interactions, a detection survey method assesses the rate of SPs detected over total number of SP interactions in a data collection period.
Dry runs	SP practice visits conducted at the end of SP training and before fieldwork with real health providers at their clinics or pharmacies. Dry runs are used for SP refresher trainings or when slight adjustments are made to the case presentation
Potential SPs	Individuals who have been recruited and/or trained to be an SP, but have not begun fieldwork. Variations of potential SPs are 'recruited SPs' or 'trainees'.
Recruited SPs	A type of potential SP who has been recruited.
Standardized patients (SPs)	SPs are individuals who are locally recruited and trained to depict tracer health conditions.
SP data entry system (SPDES)	The data process, including human resources, data management and quality assurance checks, starting from data collection to analysis is referred to as the SP data entry system throughout this manual.
Trainees	A type of potential SP who begins the SP training.
Treatment grading	The process for categorizing and coding medicines that were dispensed or prescribed during SP interactions with providers or pharmacists. Treatment graders are hired to undergo this process.
Vignette	A knowledge survey that directs questions to a respondent (in this case, a health care provider) and administered by enumerators

REFERENCES

1. Das J, Holla A, Das V, Mohanan M, Tabak D, Chan B. In Urban And Rural India, A Standardized Patient Study Showed Low Levels Of Provider Training And Huge Quality Gaps. Health affairs. 2012;31(12):2774-84.
2. Chan B, Das V, Mohanan M, Tabak D, Holla A, Das J. Standardized Patients and the Measurement of Healthcare Quality: Field Guide, Manual, and Sample Instruments. 2012.
3. Leonard K, Masatu MC. Variations In The Quality Of Care Accessible To Rural Communities In Tanzania. 26. 2007;3(2007):w380-w92.
4. Mohanan M, Vera-Hernández M, Das V, Giardili S, Goldhaber-Fiebert JD, Rabin TL, et al. The know-do gap in quality of health care for childhood diarrhea and pneumonia in rural India. JAMA pediatrics. 2015;169(4):349-57.
5. Das J, Kwan A, Daniels B, Satyanarayana S, Subbaraman R, Bergkvist S, et al. Use of standardised patients to assess quality of tuberculosis care: a pilot, cross-sectional study. The Lancet Infectious Diseases. 2015;15(11):1305-13.
6. Rowe AK, Onikpo F, Lama M, Deming MS. Evaluating health worker performance in Benin using the simulated client method with real children. Implementation Science. 2012;7(1):95.
7. Kwan A, Daniels B, Saria V, Satyanarayana S, Subbaraman R, McDowell A, et al. Variations in the quality of tuberculosis care in urban India: A cross-sectional, standardized patient study in two cities. PLOS Medicine. 2018;15(9):e1002653.
8. Das J, Holla A, Mohpal A, Muralidharan K. Quality and Accountability in Health Care delivery: audit-study evidence from primary care in India. American Economic Review. 2016;106(12):3765-99.
9. Das J, Chowdhury A, Hussam R, Banerjee AV. The impact of training informal health care providers in India: A randomized controlled trial. Science. 2016;354(6308):aaf7384.
10. Daniels B, Dolinger A, Bedoya G, Rogo K, Goicoechea A, Coarasa J, et al. Use of standardised patients to assess quality of healthcare in Nairobi, Kenya: a pilot, cross-sectional study with international comparisons. BMJ global health. 2017;2(2):e000333.
11. Satyanarayana S, Kwan A, Daniels B, Subbaraman R, McDowell A, Bergkvist S, et al. Use of standardised patients to assess antibiotic dispensing for tuberculosis by pharmacies in urban India: a cross-sectional study. The Lancet Infectious Diseases. 2016;16(11):1261-8.
12. Brown L, Tyane M, Bertrand J, Lauro D, Abou-Ouakil M, Demaria L. Quality of care in family planning services in Morocco. Studies in family planning. 1995:154-68.
13. Kohler PK, Marumo E, Jed SL, Mema G, Galagan S, Tapia K, et al. A national evaluation using standardised patient actors to assess STI services in public sector clinical sentinel surveillance facilities in South Africa. Sex Transm Infect. 2017;93(4):247-52.
14. Garcia P, Hughes J, Carcamo C, Holmes KK. Training pharmacy workers in recognition, management, and prevention of STDs: district-randomized controlled trial. Bulletin of the World Health Organization. 2003;81:806-14.
15. Fitzpatrick A, Tumlinson K. Strategies for optimal implementation of simulated clients for measuring quality of care in low-and middle-income countries. Global Health: Science and Practice. 2017:GHSP-D-16-00266.
16. Rhodes KV, Miller FG. Simulated patient studies: an ethical analysis. The Milbank Quarterly. 2012;90(4):706-24.
17. Hurst S, Avortri GS, Bhan A, Edwards S, Lazdins J, Letaief M, et al. Ethical issues in Patient Safety Research: Interpreting existing guidance. World Health Organization; 2013.
18. Casey K, Glennerster R, Miguel E. Reshaping institutions: Evidence on aid impacts using a preanalysis plan. The Quarterly Journal of Economics. 2012;127(4):1755-812.
19. Das V, Geeta, Nanda C, Purshottam, Singh R, Bajaj S. Details Matter: A step-by-step guide to implementing the standardized patient methodology as a research tool. n.d.

TABLE 2.1 REFERENCES

Global

- Bate R, Mooney L, Hess K. Medicine registration and medicine quality: a preliminary analysis of key cities in emerging markets. 2010.
- Davis DA, Mazmanian PE, Fordis M, Van Harrison RT, Thorpe KE, Perrier L. Accuracy of physician self-assessment compared with observed measures of competence: a systematic review. JAMA. 2006 Sep 6;296(9):1094-102.
- May W, Park JH, Lee JP. A ten-year review of the literature on the use of standardized patients in teaching and learning: 1996–2005. Medical teacher. 2009 Jan 1;31(6):487-92.
- Mesquita AR, Lyra DP, Brito GC, Balisa-Rocha BJ, Aguiar PM, de Almeida Neto AC. Developing communication skills in pharmacy: a systematic review of the use of simulated patient methods. Patient education and counseling. 2010 Feb 28;78(2):143-8.

Africa

- Brown L, Tyane M, Bertrand J, Lauro D, Abou-Ouakil M, Demaria L. Quality of care in family planning services in Morocco. Studies in family planning. 1995 May 1:154-68.
- Christian CS, Gerdtham UG, Hompashe D, Smith A, Burger R. Measuring Quality Gaps in TB Screening in South Africa Using Standardised Patient Analysis. International journal of environmental research and public health. 2018 Apr 12;15(4):729.
- Daniels B, Dolinger A, Bedoya G, Rogo K, Goicoechea A, Coarasa J, Wafula F, Mwaura N, Kimeu R, Das J. Use of standardised patients to assess quality of healthcare in Nairobi, Kenya: a pilot, cross-sectional study with international comparisons. BMJ Global Health. 2017 Jun 1;2(2):e000333.
- Dizon-Ross R, Dupas P, Robinson J. Governance and the effectiveness of public health subsidies. National Bureau of Economic Research; 2015 Jul 10.
- Ezire O, Okekearu I, Adeniyi F, Faweya O. Analysis of Health Facility Based Barriers and Facilitators To Use Of Sexual And Reproductive Health Care Services Among Most At Risk Populations (MARPS): Evidence From A Mystery Client Survey In Nigeria. Journal of Tropical Diseases & Public Health. 2015 Aug 19.
- Fitzpatrick, A. and Tumlinson, K., 2017. Strategies for optimal implementation of simulated clients for measuring quality of care in low-and middle-income countries. Global Health: Science and Practice, pp.GHSP-D.

- Fitzpatrick, A and McLaren, Z. The Impact of Public Health Sector Stockouts on Private Health Sector Care: Evidence from the Ugandan Antimalarial Market. Dissertation, University of Massachusetts. 2017.
- Harrison A, Wilkinson D, Lurie M, Connolly AM, Karim SA. Improving quality of sexually transmitted disease case management in rural South Africa. Aids. 1998 Nov 1;12(17):2329-35.
- Hetzel MW, Dillip A, Lengeler C, Obrist B, Msechu JJ, Makemba AM, Mshana C, Schulze A, Mshinda H. Malaria treatment in the retail sector: knowledge and practices of drug sellers in rural Tanzania. BMC public health. 2008 May 9;8(1):157.
- Kohler PK, Marumo E, Jed SL, Mema G, Galagan S, Tapia K, Pillay E, DeKadt J, Naidoo E, Dombrowski JC, Holmes KK. Sex Transm Infect. 2017 Jun 1;93(4):247-52.
- Mchome Z, Richards E, Nnko S, Dusabe J, Mapella E, Obasi A. A 'Mystery Client' Evaluation of Adolescent Sexual and Reproductive Health services in Health Facilities from Two Regions in Tanzania. PloS one. 2015 Mar 24;10(3):e0120822.
- Rowe AK, Onikpo F, Lama M, Deming MS. Evaluating health worker performance in Benin using the simulated client method with real children. Implementation Science. 2012 Dec;7(1):95.
- Tumlinson K, Speizer IS, Archer LH, Behets F. Simulated clients reveal factors that may limit contraceptive use in Kisumu, Kenya. Global Health: Science and Practice. 2013 Nov 1;1(3):407-16.
- Tumlinson K, Speizer IS, Curtis SL, Pence BW. Accuracy of standard measures of family planning service quality: findings from the simulated client method. Studies in family planning. 2014 Dec;45(4):443-70.
- Wafula F, Dolinger A, Daniels B, Mwaura N, Bedoya G, Rogo K, Goicoechea A, Das J, Olayo B. Examining the Quality of Medicines at Kenyan Healthcare Facilities: A Validation of an Alternative Post-Market Surveillance Model That Uses Standardized Patients. Drugs-Real World Outcomes. 2016 Nov 25:1-1.

Americas

- Adamo G. Simulated and standardized patients in OSCEs: achievements and challenges 1992-2003. Medical teacher. 2003 Jan 1;25(3):262-70.
- Barrows HS. An overview of the uses of standardized patients for teaching and evaluating clinical skills. ACADEMIC MEDICINE-PHILADELPHIA. 1993 Jun 1;68:443.
- Garcia P, Hughes J, Carcamo C, Holmes KK. Training pharmacy workers in recognition, management, and prevention of STDs: district-randomized controlled trial. Bulletin of the World Health Organization. 2003 Nov;81(11):806-14.
- Maynard Tucker, GI. Indigenous perceptions and quality of care of family planning services in Haiti. Health Policy and Planning. 1994 Sep 1;9(3):306-17.

- Peabody JW, Luck J, Glassman P, Dresselhaus TR, Lee M. Comparison of vignettes, standardized patients, and chart abstraction: a prospective validation study of 3 methods for measuring quality. Jama. 2000 Apr 5;283(13):1715-22.
- Planas ME, García PJ, Bustelo M, Cárcamo C, Ñopo H, Martinez S, Merino MF, Morrison A. Utilizing Standardized simulated patients to measure ethnic disparities in family planning services in Peru.
- Planas ME, García PJ, Bustelo M, Cárcamo C, Martinez S, Ñopo H, Rodriguez J, Merino MF, Morrison A. Effects of Ethnic Attributes on the Quality of Family Planning Services in Lima, Peru: A Randomized Crossover Trial. PLoS One. 2015 Feb 11; 10(2): e0115274.
- Swanson DB, van der Vleuten CP. Assessment of clinical skills with standardized patients: state of the art revisited. Teaching and learning in medicine. 2013 Jan 1;25(sup1):S17-25.
- van der Vleuten CP, Swanson DB. Assessment of clinical skills with standardized patients: state of the art. Teaching and Learning in Medicine: An International Journal. 1990 Jan 1;2(2):58-76.

Asia

- Chalker J, Ratanawijitrasin S, Chuc NT, Petzold M, Tomson G. Effectiveness of a multi-component intervention on dispensing practices at private pharmacies in Vietnam and Thailand—a randomized controlled trial. Social science & medicine. 2005 Jan 31;60(1):131-41.
- Currie J, Lin W, Zhang W. Patient knowledge and antibiotic abuse: Evidence from an audit study in China. Journal of health economics. 2011 Sep 30;30(5):933-49.
- Currie J, Lin W, Meng J. Addressing antibiotic abuse in China: An experimental audit study. Journal of Development Economics. 2014; 110:39-51.
- Daniels B, Kwan A, Satyanarayana S, Subbaraman R, Das R, Das V, Das J, Pai M. Use of standardised patients to assess gender differences in quality of tuberculosis care in urban India: a two-city, cross-sectional study. Lancet Global Health. 2019 May 1; 7(5):633-643.
- Das J, Chowdhury A, Hussam R, Banerjee AV. The impact of training informal health care providers in India: A randomized controlled trial. Science. 2016 Oct 7;354(6308):aaf7384.
- Das J, Holla A, Das V, Mohanan M, Tabak D, Chan B. In urban and rural India, a standardized patient study showed low levels of provider training and huge quality gaps. Health affairs. 2012 Dec 1;31(12):2774-84.
- Das J, Holla A, Mohpal A, Muralidharan K. Quality and accountability in healthcare delivery: Audit-study evidence from primary care in India. National Bureau of Economic Research; 2015 Jul 23.

- Das J, Kwan A, Daniels B, Satyanarayana S, Subbaraman R, Bergkvist S, Das RK, Das V, Pai M. Use of standardised patients to assess quality of tuberculosis care: a pilot, cross-sectional study. The Lancet Infectious Diseases. 2015 Nov 30;15(11):1305-13.
- Kwan A, Daniels B, Saria V, Satyanarayana S, Subbaraman R, McDowell A, Bergkvist S, Das RK, Das V, Das J, Pai M. Variations in the quality of tuberculosis care in urban India: A cross-sectional, standardized patient study in two cities. PLoS Med. 2018 Mar 9; 15(9): e1002653.
- Lu F. Insurance coverage and agency problems in doctor prescriptions: Evidence from a field experiment in China. Journal of Development Economics. 2014; 106: 156-167.
- Miller R, Goodman C. Do chain pharmacies perform better than independent pharmacies? Evidence from a standardised patient study of the management of childhood diarrhoea and suspected tuberculosis in urban India. BMJ Global Health. 2017 Sep 1;2(3): e000457.
- Mohanan M, Vera-Hernández M, Das V, Giardili S, Goldhaber-Fiebert JD, Rabin TL, Raj SS, Schwartz JI, Seth A. The know-do gap in quality of health care for childhood diarrhea and pneumonia in rural India. JAMA pediatrics. 2015 Apr 1;169(4):349-57.
- Satyanarayana S, Kwan A, Daniels B, Subbaraman R, McDowell A, Bergkvist S, Das RK, Das V, Das J, Pai M. Use of standardised patients to assess antibiotic dispensing for tuberculosis by pharmacies in urban India: a cross-sectional study. The Lancet Infectious Diseases. 2016 Nov 30;16(11):1261-8.
- Sylvia S, Shi Y, Xue H, Tian X, Wang H, Liu Q, Medina A, Rozelle S. Survey using incognito standardized patients shows poor quality care in China's rural clinics. Health policy and planning. 2015 Apr 1;30(3):322-33.
- Sylvia S, Xue H, Zhou C, Shi Y, Yi H, Zhou H, Rozelle S, Pai M, Das J. Tuberculosis detection and the challenges of integrated care in rural China: A cross-sectional standardized patient study. PLoS medicine. 2017 Oct 17;14(10):e1002405.

Australia

- Benrimoj SI, Werner JB, Raffaele C, Roberts AS, Costa FA. Monitoring quality standards in the provision of non-prescription medicines from Australian Community Pharmacies: results of a national programme. Quality and Safety in Health Care. 2007 Oct 1;16(5):354-8.

Europe

- Beullens J, Rethans JJ, Goedhuys J, Buntinx F. The use of standardized patients in research in general practice. Family Practice. 1997 Feb 1;14(1):58-62.
- Cleland JA, Abe K, Rethans JJ. The use of simulated patients in medical education: AMEE Guide No 42. Medical teacher. 2009 Jan 1;31(6):477-86.

- Rethans JJ, Sturmans F, Drop R, Van Der Vleuten C, Hobus P. Does competence of general practitioners predict their performance? Comparison between examination setting and actual practice. BMJ. 1991 Nov 30;303(6814):1377-80.
- Rutter PM, Horsley E, Brown DT. Evaluation of community pharmacists' recommendations to standardized patient scenarios.
- Watson MC, Skelton JR, Bond CM, Croft P, Wiskin CM, Grimshaw JM, Mollison J. Simulated patients in the community pharmacy setting–Using simulated patients to measure practice in the community pharmacy setting. Pharmacy World and Science. 2004 Feb 1;26(1):32-7.
- Wind LA, Van Dalen J, Muijtjens AM, Rethans JJ. Assessing simulated patients in an educational setting: the MaSP (Maastricht Assessment of Simulated Patients). Medical Education. 2004 Jan 1;38(1):39-44.

Using Standardized Patients to Measure Health Care Quality

A Manual and Toolkit for Projects in Low- and Middle-Income Countries

ANNEXES

Release v1.3
Last updated on June 21, 2019

Updated versions of the manual and annexes can be accessed at:
https://www.qutubproject.org/

Ada Kwan[1], Sofi Bergkvist[2], and Benjamin Daniels[3]
with
Jishnu Das[4], Veena Das[5], and Madhukar Pai[6]

[1]School of Public Health, University of California at Berkeley, Berkeley, USA; Development Research Group, The World Bank, Washington DC, USA. Email: adak@berkeley.edu.

[2] ACCESS Health International, New York City, New York, USA. Email: sofi@accessh.org.

[3] Development Research Group, The World Bank, Washington DC, USA. Email: bdaniels@worldbank.org.

[4]Center for Policy Research, New Delhi, India; Development Research Group, The World Bank, Washington DC, USA, Email: jdas1@worldbank.org.

[5]Department of Anthropology, Johns Hopkins University, Baltimore, USA. Email: veenadas@jhu.edu.

[6]McGill International TB Centre, McGill University, Montreal, Canada; Manipal McGill Centre for Infectious Diseases, Manipal Academy of Higher Education, Manipal, India. Email: madhukar.pai@mcgill.ca.

To use this material as a whole, or in part, please cite this manual as:

Kwan A, Bergkvist S, Daniels B, Das J, Das V, and Pai M. *Using Standardized Patients to Measure Health Care Quality: A Manual and Toolkit for Projects in Low- and Middle-Income Countries.* 2019.

This material is the Online Supplement to the following manuscript:

Kwan A, Daniels B, Bergkvist S, Das V, Pai M, Das J. The use of standardized patients for health care quality research in low- and middle-income countries. Submitted to *BMJ Global Health, 2019.*

All content in this document unless otherwise noted is licensed under the Creative Commons Attribution-NonCommercial 4.0 International License. To view a copy of this license, visit http://creativecommons.org/licenses/by-nc/4.0/ or send a letter to Creative Commons, PO Box 1866, Mountain View, CA 94042, USA.

Table of Contents

Annex A. Sample budget & justification templates (Section 3.1) A4
Annex B. Description of SP method for IRB submission (Section 5.2) A10
Annex C. Template for provider consent form (Section 5.2) A13
Annex D. Template letter of full disclosure at study completion in lieu of consent (Section 5.2).......... A16
Annex E. Ethical considerations for SP study (Section 5.2)............ A18
Annex F. Study authorization letter template from National Government (Section 5.5) A23
Annex G. Study authorization letter template from Local Government (Section 5.5) A26
Annex H. Health screening questionnaire for potential SPs (Section 5.8) A28
Annex I. SP confidentiality agreement template (Sections 5.9, 8.5) A36
Annex J. SP script – sample from Qutub Project (Section 6.2) A38
Annex K. SP exit questionnaire – sample from Qutub Project (Sections 6.2, 6.5) A45
Annex L. Follow-up detection survey and vignette – sample from Qutub Project (Sections 6.6, 6.7) ... A55
Annex M. 3-week SP training schedule............ A75
Annex N. Supervisor fieldwork schedule – Example (Section 9.2) A77
Annex O. SP fieldwork schedule – Example (Section 9.2) A80
Annex P. SP comments – Edited (Section 10.4)............ A82
Annex Q. SP data files (Section 11) A86
Annex Q1. SP data files – Provider universe master code example (Section 11.1) A87
Annex Q2. SP data files – Sample master code file example (Section 11.1) A87
Annex Q3. SP data files – Schedule and tracking master code file example (Section 11.1)............ A87
Annex Q4. SP data files – SP staff master code file example (Section 11.1)............ A87
Annex Q5. SP data files – Medicines master code file example (Section 11.1)............ A87
Annex Q6. SP data files – Exit questionnaire master data dictionary file example (Section 11.3)......... A87

ANNEX A. SAMPLE BUDGET & JUSTIFICATION TEMPLATES (SECTION 3.1)

To use this annex as a whole, or in part, please cite:

- Kwan A, Bergkvist S, Daniels B, Das J, Das V, Pai M. Using Standardized Patients to Measure Health Care Quality: A Manual and Toolkit for Projects in Low- and Middle-Income Countries. 2019.
- Das J, Kwan A, Daniels B, Satyanarayana S, Subbaraman R, Bergkvist S, Das RK, Das V, Pai M. Use of standardised patients to assess quality of tuberculosis care: a pilot, cross-sectional study. The Lancet Infectious Diseases. 2015 Nov 30;15(11):1305-13.
- Kwan A, Daniels B, Saria V, Satyanarayana S, Subbaraman R, McDowell A, et al. Variations in the quality of tuberculosis care in urban India: A cross-sectional, standardized patient study in two cities. PLOS Medicine. 2018;15(9):e1002653.

A1. Sample budget templates

Research Team	**Amount per day/person**	**Number of people**	**Number of days**	***Total***
Principal investigators				
International project manager/director and analyst				
Post-doctoral fellows				
Research assistants				
Consultants				
Technical Working Group	**Amount per day/person**	**Number of people**	**Number of days**	***Total***
Meeting venue				
Travel expenditures				
Food and snack during meetings				
SP Training	**Amount per day/person**	**Number of people**	**Number of days**	***Total***
SP/Supervisor (Junior/Senior) per diem during training				
Training location and lunch				
Training supplies				
Transportation to training for field staff				
Accommodation for field staff				
Postings and advertisements for recruitment				

A4

Data Collection Period	Amount per month/person	Number of people	Number of months	*Total*
Provider fees (consultations, tests, medicines)				
Communication costs				
Supplies				
General transportation costs				
Standardized Patient salary				
Standardized Patient transport				
Standardized Patient food allowance				
Standardized Patient communication				
Standardized Patient out of home accommodation				
Local project manager salary				
Jr. Supervisor salary				
Jr. Supervisor transport allowance				
Jr. Supervisor food allowance				
Jr. Supervisor communication				
Jr. Supervisor out of home accommodation				
Sr. Supervisor salary				
Sr. Supervisor transport allowance				
Sr. Supervisor food allowance				
Sr. Supervisor communication				
Sr. Supervisor out of home accommodation				
Medication Coding	**Amount per month/person**	**Number of people**	**Number of months**	***Total***
Auditor salary				
Auditor transport allowance				
Auditor food allowance				
Auditor communication				
Auditor accommodation				

Kwan A, *et al. BMJ Global Health* 2019; 4:e001669. doi: 10.1136/bmjgh-2019-001669

Data Management

Data Entry Software Design

Data Entry cost

Data Entry Survey Cabinet

Survey Storage for 5 years

Survey Printing

Computer Assisted Interviewing

Survey Software License

Survey Programming

Translation

Translation of the survey

Translation of qualitative responses of exit questionnaires

Other expenditures to consider

Technical advisory meeting costs

Permits needed for the study

Insurance of personnel

Miscellaneous (communication, supplies)

A2. Budget justification template for a 3-year SP study

A. Personnel and Fringe Benefits (Direct FTE Costs)	
International Project Manager/Director and Data Analyst	Role: The international PM/analyst will be responsible for coordinating across the SP field team, relevant health institutions, implementing agencies and other medical institutions, and consultants required to successfully complete the project. Along with the PIs, the international PM/analyst will be responsible for producing documentation on the SPs, achieving consensus among stakeholders and responding to specific issues that arise in the deployment of SPs and the interpretation of the data. For the successful completion of the project, the international PM/analyst will work with the PIs and stakeholders to obtain relevant permissions for the study, such as ethics review board approvals and national or local permissions. The international PM/analyst will be responsible for identifying and working with the survey programming and data entry team to ensure high quality data management from the data collection to data analysis stages, as well as working with pharmacists to determine the type and quality of drugs that are prescribed or dispensed during the SP interactions. Finally, the international PM / analyst will work with the PIs to produce timely reports, briefs and short papers for publication.<br><br>Salary: [#]% of time in year 1, and [#]% of time for years 2 and 3, based on an annual salary of $[SALARY].<br>Fringe: [#]%, which includes health insurance, pension contributions and other benefits.
Post-doctoral Fellow	Role: The Postdoctoral Fellow will handle all the sampling, compilation and statistical analysis of the data. The Postdoctoral Fellow will be based in [CITY, UNIVERSITY] with [#] trips every year to [STUDY SETTING] to work with the implementing partners and data entry team. The fellow will first work on the pilot data entry and report production design with the data team that is identified. The fellow will then complete the sampling, accounting for seasonality and random cross-matching of SPs to sampled providers. Finally, the fellow will regularly oversee the data collection, compile the data for dissemination and analyze the data for reports and publications.<br><br>Stipend: [#]% of time for the first 2 years and [#]% of time for year 3, based on an annual stipend of $[STIPEND].<br>Fringe: None.
Local Project Manager	Role: The local project manager will be responsible for the day-to-day supervision of field, data collection, and implementation activities and will be based in [STUDY SETTING]. The local PM is expected to travel frequently to all field sites, and work with the international PM, implementing partners and the PIs on any issues that arise in the field. The local PM will also coordinate among various bodies to ensure smooth implementation of the surveillance system.<br><br>Salary: [#]% of time in year 1, and [#]% of time for years 2 and 3, based on an annual salary of $[SALARY].<br>Fringe: None.
B. Direct Travel	
Trips to [STUDY SETTING]	Purpose: The funds will cover [#] trips to [STUDY SETTING] for the Post-doctoral Fellow, [#] trips to [STUDY SETTING] for the International PM, [#] trips each for the PIs. In year 1, the Fellow will conduct [#] visits for provider sampling; the Fellow or International PM, and the PIs will each travel to work with the SP field team during SP training, and to [STUDY SETTING] during the first year of

	the survey. In year 2, similar visits will take place. In year 3, the Fellow or International PM, and the PIs will travel to [STUDY SETTING] for the second year of the survey.<br><br>Number: [TOTAL NUMBER OF TRIPS]<br><br>Duration: Variable, depending on the needs of the project/person traveling.<br><br>Included: Flight (and internal flight, if applicable), lodging, local transportation and incidentals.<br>Cost: [TOTAL COSTS WITH AVERAGE TRIP COST]
In-person coordinating meetings	Purpose: Overall coordination of the project will be done through teleconferences. However, for project discussions and decisions that are more difficult or impossible to discuss/resolve over the phone, the PIs will meet in [CITIES] [#] a year in order to discuss progress, share information/data, troubleshoot project issues and hold meetings with various members of the other team on-site. These meetings will be especially crucial at the end of year 1 and at the end of year 2.<br><br>Number: Total of [#] trips.<br><br>Duration: Approximately [#] days per trip.<br><br>Included: Flight, lodging, local transportation and incidentals.<br>Cost: [TOTAL COSTS WITH AVERAGE TRIP COST]
Conferences	Purpose: We have budgeted for [#] conferences to present findings and encourage results dissemination. In order to estimate the cost, average cost per conference is imputed from the most recent [CONFERENCE OF INTEREST] in [CONFERENCE YEAR] at [CONFERENCE LOCATION].<br><br>Number: Total of [#] conferences, [#] participants<br><br>Duration: Approximately [#] days per trip.<br><br>Included: Conference registration, flight, lodging, local transportation and incidentals.<br>Cost: [TOTAL COSTS WITH AVERAGE TRIP COST]
C. Direct Consulting	
D. Direct Supplies and Other (<10k)	
Provisions have been made to pay for baseline and endline data-entry, at a total cost of $10,000.	
E. Direct Equipment (>10k)	
During the development of SP scenarios, the team will work with [DATA FIRM] to design the data-entry and data systems that will support the project's analysis and results dissemination goals. An interactive web-based (password protected) query-based system will be designed. This system will allow the research team and stakeholders to access data with specific permissions and as required. The system will also generate a number of reports based on data. The form of the reports will be decided prior to the completion of the system with the relevant stakeholders. The budget for devising this system is $[TOTAL COST].	
F. Sub-Grants and Subcontracts (Sub-Awards)	
[SP FIELD TEAM FIRM NAME]	

Kwan A, *et al. BMJ Global Health* 2019; 4:e001669. doi: 10.1136/bmjgh-2019-001669

The bulk of the project costs are for the implementation of the SP work, which includes SP visits with sampled providers, data entry, and completion of additional surveys. There are a total of [#] SP case scenarios in this project. The subcontracted firm will train [#] SPs for each case scenario, and will keep [#] SPs "in reserve", in case regular SPs should leave, for a total of [#] SPs for the entire study. With an estimated [#] providers in the sample, the total number of interactions will be [#] interactions. SPs will cost around $[MONTHLY COST] per month, including salary and travel per diems. Therefore, the total cost for SPs per year comes to $[YEARLY COST], with a total of $[TOTAL COST] for the duration of the project ([#] years). In addition, $[TOTAL COST] is budgeted for SP training. The firm contracted as the field team will also deploy [#] senior supervisors, whose costs of time and travel will be $[YEARLY COST] per year for a total of $[YEARLY COST] and [#] junior supervisors at $[YEARLY COST] per year for a total of $[YEARLY COST]. In addition, $[YEARLY COST] per year (total of $[TOTAL COST]) has been set aside as contingency funds for unexpected expenses.

G. Other Sources of Support for This Project

[INVESTIGATOR]'s time and effort for this project will not be charged to this grant, and will be provided in-kind. This represents [#]% of time and effort, based on an annual salary of $[SALARY], plus [#]% of benefits, for the duration of the project, i.e. a total of $[TOTAL COST].

H. Other Related Funded Projects

Other funds related to the project include the following.

Grantor/Title of Project Funded:

Amount Funded in USD:

Description of Related Funding:

Kwan A, *et al. BMJ Global Health* 2019; 4:e001669. doi: 10.1136/bmjgh-2019-001669

ANNEX B. DESCRIPTION OF SP METHOD FOR IRB SUBMISSION (SECTION 5.2)

Source: Qutub Project, urban India
PIs: Madhukar Pai, Jishnu Das
Project period: September 2014 – ongoing

To use this annex as a whole, or in part, please cite:

- Kwan A, Bergkvist S, Daniels B, Das J, Das V, Pai M. Using Standardized Patients to Measure Health Care Quality: A Manual and Toolkit for Projects in Low- and Middle-Income Countries. 2019.
- Das J, Kwan A, Daniels B, Satyanarayana S, Subbaraman R, Bergkvist S, Das RK, Das V, Pai M. Use of standardised patients to assess quality of tuberculosis care: a pilot, cross-sectional study. The Lancet Infectious Diseases. 2015 Nov 30;15(11):1305-13.
- Kwan A, Daniels B, Saria V, Satyanarayana S, Subbaraman R, McDowell A, et al. Variations in the quality of tuberculosis care in urban India: A cross-sectional, standardized patient study in two cities. PLOS Medicine. 2018;15(9):e1002653.

Use of Standardized Patients and Other Approaches to Measurement of Quality

A variety of different methods are available to measure quality of care. The table below summarizes different quality measures by assessing (A) the extent to which they measure knowledge versus practice; (B) the extent to which they are able to provide estimates that account for confounders; and (C) the extent to which they are able to provide information on a broad set of illnesses, highlighting the advantages and disadvantages of different measures (Source: MAQARI. Standardized patients and the measurement of healthcare quality. Field guide, manual, and sample instruments).

Measure of Quality	Measures Knowledge	Measures Practice	Accounts for Case-Mix	Accounts for Patient-Mix	Hawthorne Effects (doctors may change their behavior because of the study)	Illnesses Covered
Vignettes	Yes	No	Yes	Yes	Yes	All
Clinical Observation	No	Yes	No	No	Yes	Limited in two ways. First, “serious” illnesses like unstable angina will show up on a sporadic basis. Second, the observer never knows what the patient actually has—and doctors frequently make incorrect diagnoses.
Chart Abstraction	No	Yes	No	No	No	Similar to clinical observation, but providers rarely keep patient charts. Even when they exist, charts tend to be incomplete and don’t accurately reflect

						patient-provider interactions.
Standardized Patients	No	Yes	Yes	Yes	No	Limited to (A) adults only; (B) diseases that don't have any obvious physiological symptoms (which cannot be mimicked) and (C) conditions that don't require invasive exams—particularly in low-income countries.

While the vignettes and provider observation methods generate insight into specific components of the quality of care available, these methods have significant limitations.[1,2] Vignettes provide an accurate picture of provider knowledge for a wide range of illnesses and can control for case and patient-mix, but they do not reflect clinical practice, as a large know-do gap has been documented in a variety of settings.[1,2,3,4]

Direct clinical observation can provide information about clinical practice, but this method is limited in four ways. First, observed differences in quality may be confounded with differences in patients and illnesses presented. Although it is possible to control for case and patient mix using vignettes, observed measures of what happens in practice are subject to the usual confounders of severity and patient characteristics. Second, because the majority of patients on any given day present with self-limiting or "minor" illnesses, it is very difficult to assess process-quality for patients with severe or life-threatening illnesses. It is likely that several weeks of observation would be required before a TB patient is observed in the practice of a regular provider. Third, since it is often not possible – due to ethical reasons – to have medically trained doctors as observers, it is difficult to assess whether the illness that the patient presented with or the course of treatment prescribed or administered by the providers were indeed correct. For instance, if a patient does present with 3 weeks of cough, it will be impossible for the (medically untrained) observer to evaluate whether the patient was genuinely suffering from TB. Finally, the presence of an observer in the clinic may itself change the provider's behavior (i.e. the "Hawthorne effect").[3,4]

While we plan to triage the quality of care using these different methods, the main indicator of the quality of practice will be based on the use of SPs. Formally, a standardized patient is an individual who is extensively coached to portray the historical, physical and emotional features of an actual patient accurately and in a standardized, consistent manner. They come from all walks of life and need to be emotionally mature, affable, and intelligent and have flexible schedules (for assignments are rarely regularly scheduled).

There are two components of the SP: standardization and simulation. The objective of standardization is to present a case in a clinically accurate and consistent manner while the objective of simulation is to imitate the natural environment in which clinical encounters happen in any given social context. The goal is to "pass" as a normal patient without being detected by the medical service provider. All the SPs portraying a particular scenario are meticulously trained and rehearsed to ensure that the clinical presentation as well as the emotional, physical and psychosocial aspects of the patient they represent — speech, body language, dress, reactions to physical examinations — are standardized thus ensuring that each provider, when meeting an SP, will face the same clinical challenge. An ideal SP can also be coached to accurately recall details of his or her encounter with the healthcare providers, thus providing an opportunity to generate data on quality of care (e.g. to what degree a task is done or not done, whether or not a question is asked) and to provide feedback about the process.

The SP methodology thus presents an opportunity to control the case mix and the patient presentation, enabling us to obtain a measure of quality (e.g. case detection rate) that is comparable across all providers. It also provides a measure of clinical quality uncontaminated by Hawthorne effects and recall

bias. Compared to provider observation and vignettes, the use of SPs should give a more "real-world", and presumably more accurate, portrayal of a doctor's effort and expertise. Because vignettes measure the frontier of what the provider can do for a given case, they are relatively good at capturing errors of commission (where the provider does what is clinically inappropriate, possibly due to knowledge-related incompetence) but not as good at capturing errors of omission (where the provider fails to do what is clinically appropriate and essential, although he or she may have the appropriate knowledge). With the appropriate design of clinical cases and carefully trained SPs, it should be possible to detect both errors of omission and commission. For all these reasons, SPs are considered to be the 'gold standard' method of assessing provider communication skills and behavior.

However, SP-based studies also have their limitations. Perhaps the most restricting concern the kinds of cases that can be used in low-income countries. Due to ethical concerns, case presentations by a child are by necessity eliminated, as are those that require invasive examinations. Although invasive examinations do not preclude the use of SPs in medical education in high-income countries, in typical clinics in low-income countries, any kind of invasive examination (including the use of a thermometer) or treatment (e.g. injections) can result in a health-risk to the SP. In addition, SP-based cases are also necessarily limited to those with no clear and highly visible symptoms. However, this does not necessarily limit cases where the symptoms become noticeable only after further testing, as the quality of the provider can be ascertained based on whether the correct tests were prescribed.

Standardized patients have been used extensively in Canada in medical education settings (e.g. clinical skills and licensure exams), and in research studies aimed at improving quality of care. SPs have also been used within McGill-affiliated and MUHC hospitals for this purpose.[5,6]

References

1. Das J, Hammer J. Money for nothing: The dire straits of medical practice in Delhi, India. J Development Economics 2007;83:1-36.
2. Das J, Hammer J, Leonard K. The Quality of Medical Advice in Low-Income Countries. Journal of Economic Perspectives 2008;22:93-114.
3. Leonard K, Masatu MC. Outpatient process quality evaluation and the Hawthorne Effect. Soc Sci Med 2006;63:2330-40.
4. Leonard KL, Masatu MC. The use of direct clinician observation and vignettes for health services quality evaluation in developing countries. Soc Sci Med 2005;61:1944-51.
5. Tamblyn R, Berkson L, Dauphinee WD, et al. Unnecessary prescribing of NSAIDs and the management of NSAID-related gastropathy in medical practice. Ann Intern Med 1997;127:429-38.
6. Tamblyn RM. Use of standardized patients in the assessment of medical practice. CMAJ : Canadian Medical Association journal = journal de l'Association medicale canadienne 1998;158:205-7.

ANNEX C. TEMPLATE FOR PROVIDER CONSENT FORM (SECTION 5.2)

Source: KePSIE project, Kenya
Principal Investigators: Jishnu Das, Guadalupe Bedoya
Project period: 2015 – ongoing

To use this annex as a whole, or in part, please cite:

- Kwan A, Bergkvist S, Daniels B, Das J, Das V, Pai M. Using Standardized Patients to Measure Health Care Quality: A Manual and Toolkit for Projects in Low- and Middle-Income Countries. 2019.
- Daniels B, Dolinger A, Bedoya G, Rogo K, Goicoechea A, Coarasa J, Wafula F, Mwaura N, Kimeu R, Das J. Use of standardised patients to assess quality of healthcare in Nairobi, Kenya: a pilot, cross-sectional study with international comparisons. BMJ global health. 2017 Jun 1;2(2):e000333.

Request For Participation & Informed Consent Form

Title: **[Project name]**

Principal Investigators: **[Names]**
[Institution, Location]
In Collaboration with [Name of collaborators]

Sponsors: **[Sponsor names]**

INTRODUCTION AND PURPOSE OF THE PROJECT

You are being asked to participate in a study designed to understand what goes on when a doctor is treating a patient who may have one of a set of identified common illnesses. Results from this study will be used [*insert study purpose, such as* "to improve our study instruments and methodology before we conduct a big scale version of the study"]. Carefully read the consent form, and do not hesitate to ask questions. If you decide to participate, we will ask you to sign the form, and you will be given a copy. People who take part in such research projects do it voluntarily, and have to give their written consent. Your participation in this study is strictly voluntary. You may refuse to participate or discontinue your participation at any time without explanation, and without any prejudice.

PATIENT VISIT AND FOLLOW-UP

If you agree to participate, the coordinator will assign you a Study ID. In the following 6 months, you will be visited by someone who has been trained by us to act as a patient. These patients are called "standardized patients" and this approach has been used to assess quality of medical care. You will not know exactly when this standardized patient will visit you, but please note the date and time if/when you think you saw this standardized patient. No later than one month after this visit, our research team will contact you to find out if/when you saw our standardized patient.

POTENTIAL BENEFITS, RISKS AND/OR DISCOMFORTS

Kwan A, *et al. BMJ Global Health* 2019; 4:e001669. doi: 10.1136/bmjgh-2019-001669

You will not directly benefit from taking part in this study, and there are no risks to you from this study. The standardized patient who visits you for a consultation will pay your usual consultation fees. So, you will not suffer any economic loss due to participation in this study. While you will not directly benefit from the research, we hope that the information from this study will help us understand how the standardized patient approach can be used to better understand quality of care in [location]. We hope our research will help with this goal.

CONFIDENTIALITY

[*Insert the following if proceeding with audio recording,* "With your permission, we would like to audiotape the standardized patient visit."] All the information collected will be kept strictly confidential by identifying you with a unique code (or study ID) to which only authorized personnel will have access. The results from this study may be published, but your identity will never be revealed. Your name, coordinates, the start and end date of participation in the project, as well as audio recordings, will be stored for five (5) years after the study is over in a separate registry maintained by the investigators. In order to verify the research study data, monitors from the [Name of ethics committee(s) that provided clearance to this project] may review these records.

ETHICAL ASPECTS OF THE STUDY

The ethics committee of [Name of ethics committee(s)] have reviewed and approved this study and ensure the follow-up. They will also approve any changes made to the information/consent form and to the study procedure. In addition, [Name of ethics committee(s)] can make visits to study sites in order to ensure their quality.

QUESTIONS

If you have questions about your rights as a research subject or regarding any damage attributable to the research and wish to discuss this with someone not involved in the study, you may contact:

[Location or Area]: [Contact details]

Declaration of Consent

Title: **[Project name]**

Principal Investigators: **[Names]**
[Institution, Location]
In Collaboration with [Name of collaborators]

Sponsors: **[Sponsor names]**

I have read the content of this consent form, and I agree to participate in this research study. I have had the opportunity to ask questions and all my questions have been answered to my satisfaction. I have been given sufficient time to consider the above information and to seek advice if I choose to do so. I will be given a copy of this signed consent form. By signing the consent form, I have not given up any of my legal rights.

PARTICIPANT

	YES	NO
I consent to take part in this survey	☐	☐

Signature of the participant	Name (in block letters)	Date

PROJECT COORDINATOR
I confirm having met with the participant at the time of enrolment to answer questions about this study.

Signature of the person administering the informed consent	Name (in block letters)	Date

ANNEX D. TEMPLATE LETTER OF FULL DISCLOSURE AT STUDY COMPLETION IN LIEU OF CONSENT (SECTION 5.2)

Source: Qutub project, urban India
Principal Investigators: Madhukar Pai, Jishnu Das
Project period: September 2014 – ongoing

To use this annex as a whole, or in part, please cite:

- Kwan A, Bergkvist S, Daniels B, Das J, Das V, Pai M. Using Standardized Patients to Measure Health Care Quality: A Manual and Toolkit for Projects in Low- and Middle-Income Countries. 2019.
- Das J, Kwan A, Daniels B, Satyanarayana S, Subbaraman R, Bergkvist S, Das RK, Das V, Pai M. Use of standardised patients to assess quality of tuberculosis care: a pilot, cross-sectional study. The Lancet Infectious Diseases. 2015 Nov 30;15(11):1305-13.
- Kwan A, Daniels B, Saria V, Satyanarayana S, Subbaraman R, McDowell A, et al. Variations in the quality of tuberculosis care in urban India: A cross-sectional, standardized patient study in two cities. PLOS Medicine. 2018;15(9):e1002653.

Dear [Provider]:

We are a research team from [institution name (location)].

Over the last [time period], we have been working closely with [relevant organizations and agencies] to understand [research goals]. This is an important issue because [study rationale].

As part of this initiative, we selected providers by [method of sample selection]. These providers were from [study location(s)], and they received standardized patients over [time period of the study].

Standardized patients are regularly used in medical education and are people trained to present symptoms of a disease in a clinical interaction and to answer any questions asked by the provider. The standardized patients we sent to [study sample] presented with certain symptoms to these clinics. With permission from our research institution, all patients were unannounced which allowed us to record the nature of care being provided with validity. The identities of these clinics and providers will not be given to anyone, since our interest is in general patterns across [location] and not in the performance of any individual clinics located in our sample. [This initiative was started after first piloting the approach and checking with a large number of doctors and health care providers that there were no adverse effects on the initiative on their practices.]

We are able to provide general feedback on the results of our study, aggregated at the [level of analysis]. We are eager to hear your opinions about this study and its outcomes. We would also like to be able to discuss with you the relevance of the methods we used and ask your frank opinion about the use of unannounced patients. You are under no compulsion to discuss these findings or issues arising from our study, but if you would like to discuss these issues with us we would be happy to schedule a meeting at your convenience.

If you are interested in hearing more results about this project or would like further information, please contact us through email at [email contact] or through phone at [phone contact] and we will fix a time and place for a member of our team to visit you. Following the discussion with the member of our team, if

you have further concerns, we will put you in contact with the [ethics committee] at [institution name].

Lastly, regardless of whether you wish to contact us for further discussion or not, we want to express our grateful thanks for your contribution to our project and for the work you are doing among the population in [location].

Sincerely,
[Principal Investigators]
[Titles]

Kwan A, *et al. BMJ Global Health* 2019; 4:e001669. doi: 10.1136/bmjgh-2019-001669

ANNEX E. ETHICAL CONSIDERATIONS FOR SP STUDY (SECTION 5.2)

Source: Qutub project, urban India
Principal Investigators: Madhukar Pai, Jishnu Das

To use this annex as a whole, or in part, please cite:

- Kwan A, Bergkvist S, Daniels B, Das J, Das V, Pai M. Using Standardized Patients to Measure Health Care Quality: A Manual and Toolkit for Projects in Low- and Middle-Income Countries. 2019.
- Das J, Kwan A, Daniels B, Satyanarayana S, Subbaraman R, Bergkvist S, Das RK, Das V, Pai M. Use of standardised patients to assess quality of tuberculosis care: a pilot, cross-sectional study. The Lancet Infectious Diseases. 2015 Nov 30;15(11):1305-13.
- Kwan A, Daniels B, Saria V, Satyanarayana S, Subbaraman R, McDowell A, et al. Variations in the quality of tuberculosis care in urban India: A cross-sectional, standardized patient study in two cities. PLOS Medicine. 2018;15(9):e1002653.

ETHICAL CONSIDERATIONS

1.1 Ethics Approvals

This study will be conducted according to ethical principles stated in the Declaration of Helsinki (2008). We will submit our research proposal to the research ethics committee(s) at the McGill University Health Centre (MUHC) to obtain ethics approval prior to initiating the project. Institute of Socio-Economic Research on Development and Democracy (ISERDD), our partner site in India will also seek and obtain the necessary ethics approval.

1.2 Informed Consent – Waiver

With this submission, we are seeking approval for the main project, for which we are requesting a waiver for provider informed consent. On the governmental level, our project is explicitly included in the Memorandum of Understanding (MoU) between the Gates Foundation and the Municipal Corporation of Greater Mumbai (MCGM) [see Additional Documentation], and with a MoU between the Bihar State Government and the Gates Foundation for TB interventions. In addition, we will require the Foundation's support to get explicit approvals from both local governments for sampling providers from the public sector. With this and taking into account previous studies involving the SP method for which informed consent has been waived (described below), we believe that demonstrating a scientifically valid answer to our research questions is not possible unless the requirement of individual provider informed consent is waived.

Previous SP studies conducted by Dr. Das and colleagues from our team have requested and received waivers of informed consent from ethics committees at Harvard University, Duke University, and through the partner institution of the study presented here, ISERDD. We will share the IRB protocols and approvals, if requested, of this precedent. Another SP study conducted at the Universidad Peruana Cayetano Heredia has also received waiver of informed consent.[1]

These waivers have been granted under the provisions for waiver or alteration of the informed consent

A18

requirements under the United States Department of Health and Human Services regulations 45 CFR 46.116(d) (Office for Human Research Protections (OHRP). Accessed at: http://www.hhs.gov/ohrp/policy/consentckls.html).

This research will involve no more than minimal risk to participants. We have documented in our pilot study that our project is minimally intrusive with no risks or harms to the providers participating in the project, and in the next section, we discuss the maintenance of strict confidentiality of our research data involving several mechanisms to protect confidentiality of participating healthcare providers in the study. In our detection survey, we elicited provider opinions on whether participation in the study had adversely affected their practice in any way. The results are stark: of the 98 responding providers, not a single one replied with the affirmative. From the point of view that sending SPs to providers can be harmful, the view of the participants of our pilot unanimously demonstrates that it does not.

The lack of participant consent is unlikely to adversely affect the welfare of the participants. Based on our pilot study, we predict that the lack of the participant's consent is unlikely to adversely affect the welfare of the providers for several reasons. Firstly, no financial losses will be incurred by providers as the SPs, like real patients, will pay them whatever they charge in the clinics. There will be no added inconvenience to other patients as we will train the SPs on how to immediately step aside if there is an emergency that demands the doctor's attention. From our observations, average consultation times are between 3 and 7 minutes, so this will only inconvenience other patients only by that time. None of the identities of the providers or their health facilities will be compromised since we will maintain strict anonymity in the information collected. At no time during or after the project (or in any publications or presentations) will the providers or health facilities be identified.

Additionally, the ethics guidelines on health services audit studies state that SPs should be used in cases where the person being sent the SP is providing a service to other people and where other options have been carefully studied, but cannot answer the research questions required. For our study, we have made the educated decision to select the SP approach after more than a decade of research, during which we were unable to find adequate ways to answer the research questions about quality of health care that can be answered by using SPs. For example, with the direct observation approach, four notable issues arise:

(1) How can the true condition of the client be determined? In this case, research teams are not able to determine the actual percentage of people with three weeks cough asked to do a sputum test when there is no way to determine whether the person really has tuberculosis or not.
(2) A real tuberculosis patient is a rare even and will appear very infrequently in a clinic.
(3) What is a trained team to observe quality of care to do if the observed doctor begins to engage in malpractice?
(4) Direct observation is limited by the Hawthorne effect, which suggests that when observed while doing a job or task, individuals will have the propensity to alter their natural routine.

In short, there is no other way to get at illness-specific metrics of care, or at least none that unequivocally presented any issues. Also, after the pilot in Delhi and based on the pilot's detection results, we do not recommend any changes to the SP practice implemented and believe that the combination of greater spacing of SPs and the waiver of informed consent will bring detection rates to below 1-2 percent. Additionally, our particular study, which will evaluate the quality of care among networked providers who will attend many trainings and workshops together, confronts an added risk of the PPIA networked providers discussing the identities and personal characteristics of the SPs throughout the two-year period of the study during which we will send SPs multiple times as surveillance monitoring for two-month periods for a total of four months in each city of Mumbai and Patna, India. The combination of informed consent and congregation of providers at frequent intervention trainings (at times several are scheduled in one week) threaten the validity of our study as reported responses would not reflect the actual quality of care we are aiming to measure and the risk of SP detection may increase.

We want to emphasize that this research does not involve any therapeutic interventions or other clinical or diagnostic interventions. As part of their training, SPs are fully informed and trained on how to recognize and avoid harmful situations, such as avoiding blood draws and injections. During the pilot in Delhi, we documented 1 adverse event where a standardized patient was injected with a sterile needle. Following the event a full debriefing was conducted, and SPs were led through a refresher on avoiding all invasive examinations. There were no further adverse events noted. The next section discusses this further.

Lastly, we cite a recent study by Rhodes et al. on ethical aspects of simulated patient studies, commissioned by the US Department of Health and Human Services.[2] The review found "several relevant considerations both favor and oppose soliciting consent for simulated patient studies. Making research participation conditional on informed consent protects the autonomy of research subjects and shields them from unreasonable exposure to research risks. However, scientific validity is also an important ethical principle of human subjects research, as the net risks to subjects must be justified by the value to society of the knowledge to be gained. The use of simulated patients to monitor access is a naturalistic and scientifically sound experimental design that can answer important policy-relevant questions, with minimal risks to human subjects. As interaction between researchers and subjects increases, however, so does the need for consent."[2]

The report concluded: "As long as adequate protections of confidentiality of research data are in place, minimally intrusive simulated patient research that gathers policy-relevant data on the health system without the consent of individuals working in that system can be ethically justified when the risks and burdens to research subjects are minimal and the research has the potential to generate socially valuable knowledge." [2]

In order to fully use the potential of this "mystery client" approach and maximize its impact, SPs have to present themselves as regular patients to health providers, who therefore cannot be informed ahead of time that they will be visited by trained SPs posing as patients. We request that the requirement for provider informed consent be waived to ensure that health providers will treat the SPs as they would any regular patient.

At the end of the study, a letter of full disclosure [*See Annex D*] will be sent to debrief any provider who received an SP. The letter will offer providers a chance to further discuss any aspect of the findings or methodology and register any concerns; however, no individual data on any clinic or provider will be disclosed. Any concerns expressed by providers will be promptly communicated to the IRB.

1.3 Risks to Healthcare Providers

As the pilot study confirmed, there are no obvious risks perceived risk by doctors who will be involved in the study. Doctors will receive their usual consultation fees because standardized patients will be instructed to pay the charges, like any other patient in such settings. So, there is no economic loss for the doctors to participate.

Also, we do not anticipate any risks to the real patients of the healthcare providers for two reasons. First, these are clinics that see on average 15-20 patients a day and the providers spend 3-5 minutes per patient (as shown in our previous SP study in urban and rural India[3], and in our pilot project). Therefore, it is not the case that our study is going to add substantially to the waiting time for any of the patients--we estimate the additional waiting time to be at most 5 minutes. In addition, our protocol also dictates that if there is a medical emergency in the clinic, our SP will immediately step aside.

1.4 Risks to standardized patients

In the previous study in rural India[3], detection rates were less than 1% and one risk was uncovered (providers may try to conduct a tuberculin skin test without asking the SP), and the appropriate risk mitigation measure (SPs must keep their hands below any desk) was designed and implemented during SP training.

In our pilot TB study in India, the detection rate was about 5% of all the SP interactions. We documented 1 adverse event where a standardized patient was given an injection with a disposable, sterile needle. The reason for this violation was that the standardized patient thought that the provider was going to check his blood pressure, when the provider injected him instead. Following the event a full debriefing was conducted and standardized patients were led through a refresher on avoiding all invasive examinations. There were no further adverse events noted.

During each month of data collection, the supervisors will hold two meetings with all the SPs to review the dos and don’ts with regard to SP safety and risk mitigation strategies. During these meetings, supervisors will go over instructions for the SPs on how to avoid invasive or potentially unclean examinations (e.g. thermometers) and interventions (e.g. injections), such as avoiding the placement of their arms on the table and always asking the provider what he intends to do if he moves toward the SP for any examination.

Additionally, there will be a review once a week during which SPs will be asked to describe any situation that arose with regard to invasive procedures and what tactics were used to avoid or refuse such events. SPs will be reminded in these weekly meetings that rather than risk invasive procedures, they should reveal their identities and give the supervisor’s phone number to the provider if they feel that the provider is aggressively pursuing an invasive procedure (we note that this situation did not arise during the pilot in Delhi). Any such a case will be recorded as an adverse event with clear documentation of the circumstances that led to the disclosure.

1.5 Potential benefits

There are no direct benefits for the providers/doctors involved in the study. However, this study will serve to assess the usefulness and impact of the standardized patient strategy to evaluate quality of tuberculosis care, which can in turn inform policy and decision makers, and further the goal of TB control in India. Thus, there is an important public health/societal benefit. Our project will be India’s first-ever larger-scale study of quality of TB care using standardized patients, and if our pilot findings hold true on a larger scale, it can offer valuable insights for intervention and policy.

1.6 Confidentiality

We have documented that our project will maintain strict confidentiality of our research data involving several mechanisms to protect the confidentiality of participating healthcare providers in the study. All study data will be kept confidential. The identity of providers who participate in the study will be anonymized through the process described below. This process will be communicated explicitly to those involved.

During training and throughout data collection, all standardized patients participating in the study are debriefed on their critical duty to restrain themselves from discussing SP and fieldwork experiences with individuals outside of the research team (e.g., family members, friends, neighbors). Standardized patients and supervisors conduct the exit questionnaires and debrief sessions in spaces where they are not to be overheard from others and away from the location of the SP-provider interaction.

A21

All exit questionnaires will be completed on paper, and thus will need to be entered by data entry operators. All data entry operators will sign a confidentiality form stating that they will not discuss or expose any information related to the survey to any person outside the research team. To ensure the confidentiality and the safety of the information gathered, all data will be accessed through a secure domain and stored on a Microsoft Windows SQL server 2008 R2. An extensible web server called IIS (Internet Information Services) 7.5 created by Microsoft will be used.

After data from the SP-provider interactions are entered, they will be retrieved through the secure server by the study investigators. Study investigators will then strip all provider identifiers (for this study, the term "provider identifiers" means: provider name, GPS codes, street address, work place and address if applicable, mobile or fixed telephone numbers, other contact information) and assign numerical code IDs to each provider as the first step in receiving data. Each provider in the study will have their own numerical code ID, and the access to the file that matches provider numerical code IDs to provider identifiers will be restricted to the study investigators only. All study documents (e.g., completed exit questionnaires) will be kept in a locked cabinet at a designated office at each study site. The keys to the locked cabinets will be with the project coordinator at each site. The list that associates provider identifiers with code ID will be kept in a password-protected secure server.

Databases will be constructed from these de-identified data and will be used in analysis and generation of the six-month reports for the Private Provider Interface Agencies in Mumbai and Patna. This also pertains to any future use of data generated from this study.

Expert panel members who will participate in treatment coding will at no time receive any data that contain identifiable characteristics for providers, supervisors, or SPs. This will protect participants and maintain their anonymity, in addition to eliminating any coding bias.

Additionally, to minimize the likelihood of identifying providers or their institutions in this study[4], data used by the PIs to generate six-month reports for the PPIA in Mumbai and Patna will be aggregated at the ward level. Participant names and other identifying information will not be used in any reports of the research, and any quote used will be anonymized.

References

1. Planas ME, Garcia P, Bustelo M. Using standardized simulated patients to measure ethnic disparities in family planning services in Peru: Study protocol and pre-trial procedures of a crossover randomized trial. URL: http://publications.iadb.org/bitstream/handle/11319/6387/Using%20standardized%20simulated%20patients%20to%20measure%20ethnic%20disparities%20in%20family%20planning%20services%20in%20Peru.pdf?sequence=4. Inter-American Development Bank Technical Note No IDB-TN-640 2014.
2. Rhodes KV, Miller FG. Simulated patient studies: an ethical analysis. The Milbank quarterly 2012;90:706-24.
3. Das J, Holla A, Das V, Mohanan M, Tabak D, Chan B. In urban and rural India, a standardized patient study showed low levels of provider training and huge quality gaps. Health Aff (Millwood) 2012;31:2774-84.
4. World Health Organization. Ethical issues in patient safety research: Interpreting existing guidance. URL: http://apps.who.int/iris/bitstream/10665/85371/1/9789241505475_eng.pdf. Geneva: WHO; 2013.

ANNEX F. STUDY AUTHORIZATION LETTER TEMPLATE FROM NATIONAL GOVERNMENT (SECTION 5.5)

Source: KePSIE project, Kenya
Principal Investigators: Jishnu Das, Guadalupe Bedoya
Notes: 2-page letter template

To use this annex as a whole, or in part, please cite:

- Kwan A, Bergkvist S, Daniels B, Das J, Das V, Pai M. Using Standardized Patients to Measure Health Care Quality: A Manual and Toolkit for Projects in Low- and Middle-Income Countries. 2019.
- Daniels B, Dolinger A, Bedoya G, Rogo K, Goicoechea A, Coarasa J, Wafula F, Mwaura N, Kimeu R, Das J. Use of standardised patients to assess quality of healthcare in Nairobi, Kenya: a pilot, cross-sectional study with international comparisons. BMJ global health. 2017 Jun 1;2(2):e000333.

Kwan A, *et al. BMJ Global Health* 2019; 4:e001669. doi: 10.1136/bmjgh-2019-001669

[GOVERNMENT INSTITUTION]
OFFICE OF [GOVERNMENT AGENCY/UNIT]

[CONTACT LINE 1]
[CONTACT LINE 2]
[CONTACT LINE 3]

[ADDRESS LINE 1]
[ADDRESS LINE 1]
[ADDRESS LINE 1]

[LETTER REFERENCE NUMBER]

[DATE]

[HEALTHCARE PROVIDER DETAILS LINE 1]
[HEALTHCARE PROVIDER DETAILS LINE 2]
[HEALTHCARE PROVIDER DETAILS LINE 3]

RE: [PROJECT NAME]

Patient safety and quality of care are crucial to the wellbeing of millions of [POPULATION]. The [GOVERNMENT INSTITUTION] is deeply interested in understanding and improving the safety and quality of care that [POPULATION] receive in health facilities, both public and private. As part of our continuing efforts, we have partnered with [ORGANIZATION NAME] to implement the [PROJECT NAME]. Under [PROJECT NAME], [DESCRIPTION OF STUDY] will be evaluated using gold standard evaluation methods between [START YEAR] and [END YEAR].

In order to develop the methodologies and tools necessary to measure patient safety and quality of care, in coordination with the [GOVERNMENT INSTITUTION], the [PROJECT] team will test different instruments to systematically collect information from health facilities between [START DATE] and [END DATE]. The successful completion of these tests will lead to the finalization of important tools to measure patient safety and quality of care in [POPULATION] health facilities. We seek your permission to carry out these important activities in your health facility. You should be aware that you are not required to consent to these activities, and if you choose not to participate, there will be no repercussions on the part of the [GOVERNMENT INSTITUTION]. You should also be aware that any data collected will remain strictly anonymous, and data on any health facility will not be tied to their name or location in a way that allows positive identification to be made by a third party and/or the [GOVERNMENT INSTITUTION].

We hope that you will grant the evaluation team the permission for these activities and look forward to your cooperation. If you have any questions about the impact evaluation or the development of these tools, please feel free to contact [GOVERNMENT CONTACT NAME] at [GOVERNMENT INSTITUTION] at the following email: [EMAIL] and mobile number: [MOBILE NUMBER].

List of activities:

1. **Standardized Patients:** Surveyors drawn from local communities will be extensively trained to present as patients with tracer conditions. Data on adherence to guidelines of care, including adherence to history taking and examination checklists, diagnosis and treatments and patient safety will be will extracted from these interactions.
2. [STUDY ACTIVITY 2]
3. [STUDY ACTIVITY 3]

Kwan A, *et al. BMJ Global Health* 2019; 4:e001669. doi: 10.1136/bmjgh-2019-001669

[AGENCY OFFICIAL'S SIGNATURE]

____

[AGENCY OFFICIAL'S NAME]
[AGENCY OFFICIAL'S TITLE]

ANNEX G. STUDY AUTHORIZATION LETTER TEMPLATE FROM LOCAL GOVERNMENT (SECTION 5.5)

Source: KePSIE project, Kenya
Principal Investigators: Jishnu Das, Guadalupe Bedoya
Notes: 1-page letter template

To use this annex as a whole, or in part, please cite:

- Kwan A, Bergkvist S, Daniels B, Das J, Das V, Pai M. Using Standardized Patients to Measure Health Care Quality: A Manual and Toolkit for Projects in Low- and Middle-Income Countries. 2019.
- Daniels B, Dolinger A, Bedoya G, Rogo K, Goicoechea A, Coarasa J, Wafula F, Mwaura N, Kimeu R, Das J. Use of standardised patients to assess quality of healthcare in Nairobi, Kenya: a pilot, cross-sectional study with international comparisons. BMJ global health. 2017 Jun 1;2(2):e000333.

[LOCAL GOVERNMENT INSTITUTION]
OFFICE OF [LOCAL GOVERNMENT AGENCY/UNIT]

[CONTACT LINE 1]
[CONTACT LINE 2]
[CONTACT LINE 3]

[ADDRESS LINE 1]
[ADDRESS LINE 1]
[ADDRESS LINE 1]

[LETTER REFERENCE NUMBER] **[DATE]**

[RESEARCH CONTACT / NATIONAL GOVERNMENT CONTACT DETAILS LINE 1]
[RESEARCH CONTACT / NATIONAL GOVERNMENT CONTACT DETAILS LINE 2]
[RESEARCH CONTACT / NATIONAL GOVERNMENT CONTACT DETAILS LINE 3]

RE: AUTHORITY TO CARRY OUT [PROJECT NAME]

Thank you for your letter dated [DATE OF NATIONAL GOVERNMENT LETTER].

This is to inform you that the [LOCAL GOVERNMENT INSTITUTION], [LOCAL GOVERNMENT AGENCY/UNIT] has reviewed and approved your above research subject to compliance with the following requirements:

- The team will be expected to adhere to the rules and regulations pertaining to [LOCAL GOVERNMENT INSTITUTION].
- That during their research there will be no cost devolving to the [LOCAL GOVERNMENT INSTITUTION].
- That you undertake to indemnify the [LOCAL GOVERNMENT INSTITUTION] against any claim that may arise from the research.
- A copy of the findings must be submitted to the office of the undersigned.

By copy of this letter the healthcare providers and [LOCAL GOVERNMENT HEALTH UNITS] of [REGION 1], [REGION 2], […] are requested to give you the necessary support.

[AGENCY OFFICIAL’S SIGNATURE]

____

[AGENCY OFFICIAL’S NAME]
[AGENCY OFFICIAL’S TITLE]

C.C. – [LOCAL GOVERNMENT HEALTH UNITS AND HEALTHCARE PROVIDERS IN STATED REGIONS]

ANNEX H. HEALTH SCREENING QUESTIONNAIRE FOR POTENTIAL SPS (SECTION 5.8)

Source: KePSIE project, Kenya
Principal Investigators: Jishnu Das, Guadalupe Bedoya
Notes: 7-page adult health screening questionnaire

To use this annex as a whole, or in part, please cite:

- Kwan A, Bergkvist S, Daniels B, Das J, Das V, Pai M. Using Standardized Patients to Measure Health Care Quality: A Manual and Toolkit for Projects in Low- and Middle-Income Countries. 2019.
- Daniels B, Dolinger A, Bedoya G, Rogo K, Goicoechea A, Coarasa J, Wafula F, Mwaura N, Kimeu R, Das J. Use of standardised patients to assess quality of healthcare in Nairobi, Kenya: a pilot, cross-sectional study with international comparisons. BMJ global health. 2017 Jun 1;2(2):e000333.

Kwan A, *et al. BMJ Global Health* 2019; 4:e001669. doi: 10.1136/bmjgh-2019-001669

Adult Initial Health History

Name ____
First Middle Last

Today's Date______________________Date of Birth ______________

Address____

Telephone Number (home)()________________
(cell) (_______)________________
(work) (_______)________________

GENERAL HEALTH

1. In general, what do you consider to be your **main health problem**(s)? (Check all that apply.)

☐ heart problems ☐ diabetes
☐ stomach problems ☐ depression/emotional problems
☐ ear, nose, or throat problems ☐ joint problems
☐ high blood pressure
☐ Other(s) – please explain________________________________

____

2. How would you **describe your health**?

☐ Excellent ☐ Very Good ☐ Good ☐ Fair ☐ Poor

3. Are you taking any **prescription medicines**?

☐ Yes. Please list your medicines below OR ☐ I brought my pill bottles or a list.
☐ No, I do not take any prescription medicines. (If no, go to question #5.)

**Name of medicine**	**Amount / size of pill**	**How many pills or doses do you take at**
**Example:** Furosemide	20 mg	2 morning 2 noon ___dinner ___bed
		___morning ___noon ___dinner ___bed
		___morning ___noon ___dinner ___bed
		___morning ___noon ___dinner ___bed
		___morning ___noon ___dinner ___bed
		___morning ___noon ___dinner ___bed

Kwan A, *et al. BMJ Global Health* 2019; 4:e001669. doi: 10.1136/bmjgh-2019-001669

		___morning ____noon ___dinner ____bed

(Please use the back of this form if you have more prescription medicines.)

4. What **over-the-counter medicines**, do you take regularly?
- ☐ Pain reliever (for example: Tylenol, Advil, Motrin, Aleve, aspirin)
- ☐ Vitamins
- ☐ Antacid (for example: Tums, Prilosec)
- ☐ Herbal medicine (please list) ____________________
- ☐ Other (please list) ____________________
- ☐ None - I do not take any over-the-counter medicines regularly.

5. Have you ever had any **allergic reaction (bad effects) to a medicine** or a shot?
- ☐ Yes. (Please write the name of the medicine and the effect you had.)
- ☐ No, I am not allergic to any medicines.

**Medicine I am allergic to**	**What happens when I take that medicine**
**Example:** Atenolol	I get a rash

6. Do you get an **allergic reaction (bad effect)** from any of the following? (Check all that apply)
- ☐ Latex (rubber gloves)
- ☐ Grass or pollen
- ☐ Eggs
- ☐ Shellfish
- ☐ Other (please describe) ____________________
- ☐ No - I have no allergies that I know of.

7. Have you ever been a **patient in a hospital** overnight?
- ☐ Yes. (If yes, explain EACH reason and when.)
- ☐ No, I have never been a patient in a hospital. (If no, go to question #9)

**I was in the hospital because:**	**When**
**Example:** Heart Attack	6 years ago

Kwan A, *et al. BMJ Global Health* 2019; 4:e001669. doi: 10.1136/bmjgh-2019-001669

FOR WOMEN ONLY

8. Have you ever been **pregnant**? …………………………………… ☐ Yes ☐No
How many times?__________________
How many children have you given birth to?_________________

9. Have you had a **PAP smear**? ……………………………………… ☐ Yes ☐No
Date of last one ____________________(MM/DD/YY)

10. Have you ever had a **PAP smear that was not normal**? ………… ☐ Yes ☐No

11. Have you had a **mammogram** (breast x-ray)?.................................. ☐ Yes ☐No
Date of last one______________(MM/DD/YY)

SHOTS

12. When was your last **Tetanus shot**?.............Year_____ ☐ Never ☐ Don't know

SOCIAL HISTORY

13. Circle the **highest grade** you finished in school?

Grade School	High School	Vocational School	College
1 2 3 4 5 6 7 8	9 10 11 12 GED	1 2 3	1 2 3 4+

14. What **language** do you prefer to speak? ☐ English ☐Swahili ☐Other ___________

15. How well can you **read**?

☐ Very well ☐ Well ☐ Not well ☐ I can not read

16. Have you **ever smoked cigarettes, cigars, used snuff, or chewed tobacco**?
☐ No (if no, go to question #17.)
☐ Yes
a. When did you start?____
b. How much per week? ____
c. Have you quit?............................☐ No ☐ Yes, when___________________
d. Do you want to quit?...................☐ No ☐Yes ☐ Already Quit

Kwan A, *et al. BMJ Global Health* 2019; 4:e001669. doi: 10.1136/bmjgh-2019-001669

17. Do you drink **alcohol**?
☐ No (if no, go to question #18.)
☐ Yes
a. Have you ever felt you ought to cut down on your drinking? ☐ Yes ☐No
b. Have people ever annoyed you by criticizing your drinking? ☐ Yes ☐No
c. Have you ever felt bad or guilty about your drinking? ……..☐ Yes ☐No
d. Have you ever had a drink first thing in the morning? ……..☐ Yes ☐No

18. Are you ☐ Single ☐ Married ☐ Partnered ☐ Divorced or Separated ☐ Widowed?

19. **EXERCISE**

**Describe what kind of exercise you do. (Check all that apply.)**	**How many days per week do you exercise?**	**For how long do you exercise <u>each day</u>?**
☐ walking<br>☐ biking<br>☐ swimming<br>☐ weight training<br>☐ yoga<br>☐ other<br>☐ I do not exercise	☐ once per week<br>☐ twice per week<br>☐ 3 times a week<br>☐ 4 times a week<br>☐ 5 times a week<br>☐ 6 times a week<br>☐ 7 times a week or more	☐ less than 15 minutes<br>☐ 15-30 minutes<br>☐ 30 – 45 minutes<br>☐ 45 minutes – 1 hour<br>☐ over 1 hour
Comments:		

FAMILY HISTORY

What medical problems do people in your family have?

Family Member	Medical Problems
Mother:	☐ Diabetes (sugar) ☐ High blood pressure ☐ Heart problems<br>☐ Cancer ☐ other: ________________
Father:	☐ Diabetes (sugar) ☐ High blood pressure ☐ Heart problems<br>☐ Cancer ☐ other: ________________
Sisters:	☐ Diabetes (sugar) ☐ High blood pressure ☐ Heart problems<br>☐ Cancer ☐ other: ________________
Brothers:	☐ Diabetes (sugar) ☐ High blood pressure ☐ Heart problems<br>☐ Cancer ☐ other: ________________

Kwan A, *et al. BMJ Global Health* 2019; 4:e001669. doi: 10.1136/bmjgh-2019-001669

HISTORY OF MEDICAL CONDITIONS

Have you **ever** had any of the following conditions? (Check all that apply)

☐ Anemia (low iron blood) ☐ Asthma (wheezing) ☐ Diabetes (sugar)
☐ Heart Trouble ☐ Hemorrhoids (piles) ☐ Cancer
☐ Hepatitis (yellow jaundice) ☐ Tuberculosis (TB) ☐ Liver Trouble
☐ Pneumonia ☐ Rheumatic fever ☐ Ulcers
☐ Stroke ☐ High Blood Pressure
☐ Skin problems ☐ Depression (feeling down or blue)
☐ Epilepsy (fits, seizures) ☐ Anxiety (nerves, panic attacks)
☐ VD, STD (syphilis, gonorrhea, chlamydia, HIV)
☐ Other____

REVIEW OF SYMPTOMS

		YES	NO
**Sleeping**	Do you **feel tired** a lot?	☐ yes	☐ no
	Do you have **trouble falling or staying asleep**?	☐ yes	☐ no
	Do you have **other problems with sleep**?	☐ yes	☐ no
**Eating**	Have you **lost your appetite** recently?	☐ yes	☐ no
	Have you **lost weight** in the last year without trying?	☐ yes	☐ no
	Do you **eat too much** or **have you gained weight** recently?	☐ yes	☐ no
	Do you have **other problems with eating**?	☐ yes	☐ no
**Throat**	Do you have **sore throats** a lot?	☐ yes	☐ no
	Do you have **other problems with your throat**?	☐ yes	☐ no
**Ears**	Do you have **trouble hearing**?	☐ yes	☐ no
	Do you wear a **hearing aid**?	☐ yes	☐ no
	Do you have constant **ringing or noises** in your ears?	☐ yes	☐ no
	Do you have **other problems with your ears?**	☐ yes	☐ no
**Back**	Do you have **back pain**?	☐ yes	☐ no
	Do you have any **other problems with your back**?	☐ yes	☐ no
**Eyes**	Do you have **trouble with your vision** or seeing?	☐ yes	☐ no
	Do you wear **glasses or contacts**?	☐ yes	☐ no

Kwan A, *et al. BMJ Global Health* 2019; 4:e001669. doi: 10.1136/bmjgh-2019-001669

	Do you have **other problems with your eyes**?	☐ yes	☐ no
**Nose and Sinuses**	Do you have a **runny or stopped up nose** a lot?<br><br>Do you have **other problems with your nose or sinuses?**	☐ yes<br><br>☐ yes	☐ no<br><br>☐ no
**Teeth and Mouth**	Do you have **sore or bleeding gums**?<br><br>Do you wear **plates or false teeth**?<br><br>Do you have **other problems with your teeth and mouth?**	☐ yes<br><br>☐ yes<br><br>☐ yes	☐ no<br><br>☐ no<br><br>☐ no
**Heart or Breathing**	Do you ever have **pain/tightness in your chest** when working or exercising?<br><br>Do you **wake up at night with trouble breathing**?<br><br>Do you have a **racing or skipping heartbeat** at times?<br><br>Do you have **other heart or breathing problems**?	☐ yes<br><br>☐ yes<br><br>☐ yes<br><br>☐ yes	☐ no<br><br>☐ no<br><br>☐ no<br><br>☐ no
**Bowel movements**	Do you have **bowel movements (poop) that are black, like tar, or bloody?**<br><br>Do you have **any other problems with your bowel movements (poop)?**	☐ yes<br><br>☐ yes	☐ no<br><br>☐ no
**Peeing and Kidney Stones**	Do you have **trouble passing your urine (peeing)?**<br><br>Does it **burn when you pass urine (pee)?**<br><br>Do you have to **pee more than 2 times a night**?<br><br>Do you **leak urine (pee)?**<br><br>Have you ever passed **kidney stones**?<br><br>Do you have any **other problems with your peeing**?	☐ yes<br><br>☐ yes<br><br>☐ yes<br><br>☐ yes<br><br>☐ yes<br><br>☐ yes	☐ no<br><br>☐ no<br><br>☐ no<br><br>☐ no<br><br>☐ no<br><br>☐ no
**Joints**	Do you have **swollen or painful joints**?<br><br>Do you have any **other problems with your joints**?	☐ yes<br><br>☐ yes	☐ no<br><br>☐ no
**Head, Balance, Fever and Weakness**	Do you have **frequent or severe headaches**?<br><br>Have you ever **fainted (passed out)**?<br><br>Have you **lost your balance and fallen** recently?<br><br>Do you have **weakness** in any part of your body?	☐ yes<br><br>☐ yes<br><br>☐ yes<br><br>☐ yes	☐ no<br><br>☐ no<br><br>☐ no<br><br>☐ no

Kwan A, *et al. BMJ Global Health* 2019; 4:e001669. doi: 10.1136/bmjgh-2019-001669

	Have you had a **fever** within the past month?	☐ yes	☐ no
	Do you have any **other problems with your head or balance?**	☐ yes	☐ no
**Emotional Health**	Do you get **upset easily**?	☐ yes	☐ no
	Do **frightening thoughts** keep coming into your mind?	☐ yes	☐ no
	Have you ever been **hospitalized for nerves, thoughts or moods**?	☐ yes	☐ no
	During the past 2 weeks, have you often been bothered by having **little interest or pleasure in doing things**?	☐ yes	☐ no
	During the past 2 weeks, have you often been bothered by feeling **down, depressed, or hopeless**?	☐ yes	☐ no
	Do you have any **other problems with your emotional health?**	☐ yes	☐ no
		☐ yes	☐ no
**Men Only**	Have you ever had **prostate trouble**?	☐ yes	☐ no
	Do you have any **other male problems**?	☐ yes	☐ no
**Women Only**	Do you have **pain or lumps in your breast**?	☐ yes	☐ no
	Do you have unusual **vaginal discharge or itching**?	☐ yes	☐ no
	Do you or have you taken **hormones (such as birth control pills)**?	☐ yes	☐ no
	Do you have any **other female problems**?	☐ yes	☐ no

ANNEX I. SP CONFIDENTIALITY AGREEMENT TEMPLATE (SECTIONS 5.9, 8.5)

Source: KePSIE project, Kenya
Principal Investigators: Jishnu Das, Guadalupe Bedoya
Project period: 2015 – ongoing

To use this annex as a whole, or in part, please cite:

- Kwan A, Bergkvist S, Daniels B, Das J, Das V, Pai M. Using Standardized Patients to Measure Health Care Quality: A Manual and Toolkit for Projects in Low- and Middle-Income Countries. 2019.
- Daniels B, Dolinger A, Bedoya G, Rogo K, Goicoechea A, Coarasa J, Wafula F, Mwaura N, Kimeu R, Das J. Use of standardised patients to assess quality of healthcare in Nairobi, Kenya: a pilot, cross-sectional study with international comparisons. BMJ global health. 2017 Jun 1;2(2):e000333.

Request for Confidentiality & Disclosure of the Study Form

Title: **[Project title]**

INTRODUCTION AND PURPOSE OF THE PROJECT

You are being asked to participate in a study designed to understand what goes on when a doctor is treating a patient who may have one of a set of identified common illnesses. Results from this study will be used [*insert study purpose, such as* "to improve our study instruments and methodology before we conduct a big scale version of the study"].

[*If the study does not have a waiver for provider informed consent, insert,* "People who will take part in this research project will do it voluntarily, and have to give their written consent.] Your participation in this study is strictly voluntary. You may refuse to continue your participation without explanation, and without any prejudice.

POTENTIAL BENEFITS, RISKS AND/OR DISCOMFORTS

You may only directly benefit from taking part in this study from the allowances and wages that are given based on the level of effort and there are no risks to you from this study.

CONFIDENTIALITY & DISCLOSURE

All the information collected will be kept strictly confidential and only authorized personnel will have access. If you decide to continue with the training and subsequent study, we expect that you will keep the information that you receive during the training and subsequent study strictly confidential and you may not discuss about the facilities and the health workers you interact with your contacts, including family, friends, and relatives during or after the study.

Kwan A, *et al. BMJ Global Health* 2019; 4:e001669. doi: 10.1136/bmjgh-2019-001669

PARTICIPANT

	YES	NO
I agree to keep the information of the survey confidential.	☐	☐

________________________ Signature of the participant

________________________ Name (in block letters)

____________ Date

Kwan A, *et al. BMJ Global Health* 2019; 4:e001669. doi: 10.1136/bmjgh-2019-001669

ANNEX J. SP SCRIPT – SAMPLE FROM QUTUB PROJECT (SECTION 6.2)

Source: Qutub project pilot in Delhi
Note: 6-page script for male and female classic case of suspected tuberculosis (2-3 week cough and fever)

To use this annex as a whole, or in part, please cite:

- Kwan A, Bergkvist S, Daniels B, Das J, Das V, Pai M. Using Standardized Patients to Measure Health Care Quality: A Manual and Toolkit for Projects in Low- and Middle-Income Countries. 2019.
- Das J, Kwan A, Daniels B, Satyanarayana S, Subbaraman R, Bergkvist S, Das RK, Das V, Pai M. Use of standardised patients to assess quality of tuberculosis care: a pilot, cross-sectional study. The Lancet Infectious Diseases. 2015 Nov 30;15(11):1305-13.

ISERDD | SP1-NARRATIVE | QuTub Project

Standardized Case 1: Classic case of suspected TB (with no antibiotics or x-ray)

स्टैन्डराईज्ड केस 1: क्लासिक केस ऑफ सस्पेक्टेड टी बी (बिना एन्टीबायोटिक या एक्स-रे)

Ravi (Male)

Ravi is a 35 year old male who has studied up to 10th standard. He is the owner of a small tea shop. Today, in the morning like any other day, when he leaves for his work, his wife Rekha, handing him his lunch box asks, "why are you not eating your lunch properly - you get most of it uneaten every day"? Ravi replies, "I have cough and seem to have lost my appetite". Ravi's family is small. It consists of his wife and two children, aged six (daughter) and four (son) and they live in a two room house which he owns. His business at the tea stall is doing well as he is able to earn on average rupees 8000 - 10000 per month. Generally Ravi keeps good health. He has not had any major health problems or any chronic Illness. His wife and children too are in good health. But since last 2-3 weeks he is suffering from cough which is more or less present during early morning and night, and it also has expectoration though that does not have any color in it and is clear. He also has low grade mild fever, on and off, which gets worse during the evening time. But since this problem started he feels a bit tired and also has lost some weight, as his clothes have got a bit loose. He does not suffer from any associated chest or body pain. Around 1 week ago, he had visited a local chemist who gave him a cough syrup and some pills for the fever. He smokes 4-5 *beedis* during the day since last 8-10 years and drinks alcohol once or twice in the month. His relationship with his wife is good. He loves her very much. He has a cheerful and an easy going personality but today his face bears a tense look as he is worried about his cough and fever and visits a doctor nearby.

रवि (पुरुष)

रवि एक 35 साल का व्यक्ति है जिसने 10वीं कक्षा तक पढ़ाई की है। रोजगार के लिये वह अपनी चाय की दुकान चलाता है। जिसे वह खोलने के लिये हर रोज की तरह आज भी अपने घर से निकलता है और निकलते समय उसकी पत्नी रेखा खाने का डब्बा देते हुये बोलती है कि "क्या बात है आज-कल आप खाना ठीक से नहीं खा रहे हो"? रवि कहता है "मुझे खाँसी है और भूख भी कम लग रही है"। रवि का छोटा परिवार है, जिसमें उसकी पत्नी और दो बच्चे है, लड़की की उम्र 6 साल और लड़के की 4 साल है। रवि दो कमरे के खुद के मकान में रहता है। रवि की चाय की दुकान ठीक चलती है जिससे वह औसतन 8 से 10 हजार रुपये महीना कमा लेता है।

आमतौर पर रवि का स्वास्थ्य अच्छा रहता है, उसे किसी भी तरह की तकलीफ और कोई लम्बी बिमारी नहीं है। उसकी पत्नी और बच्चों का स्वास्थ्य भी अच्छा है। लेकिन रवि को पिछले 2-3 हफ्तों से खाँसी है, जो सुबह और रात के समय ज्यादा होती है। उसकी खाँसी के साथ बलगम भी आता है जिसका कोई रंग नहीं है, वह साफ है। खाँसी के साथ उसे हल्का बुखार रहता है जो चढ़ता-उतरता है, लेकिन अक्सर शाम के समय ही ज्यादा होता है। जब से उसे यह तकलीफ शुरु हुई है तब से उसे थकावट महसूस हो रही है। उसे लगता है कि उसका कुछ वजन कम हो गया है क्योंकि उसके कपड़े ढीले हो गये है। उसे इस तकलीफ में किसी भी तरह का छाती का दर्द और बदन दर्द नहीं है। एक हफ्ता पहले रवि ने इस तकलीफ के लिये घर के नजदीक के कैमिस्ट से दवा ली थी। कैमिस्ट ने खाँसी का सीरप और बुखार के लिये कुछ गोलियाँ दी थी।

वह दिन में 4 से 5 बीड़ी पीता है और उसकी यह आदत पिछले 8-10 सालों से है। महीने में एक या दो बार शराब का सेवन भी कर लेता है। उसके अपनी पत्नी के साथ अच्छे सम्बन्ध है। वह उसे बहुत प्यार करता है। वह हंसमुख और मिलनसार स्वभाव का व्यक्ति है, लेकिन आज उसके चेहरे पर अपनी खाँसी और बुखार को लेकर थोड़ी परेशानी है जिसको लेकर वह नजदीक के डॉक्टर के पास गया है।

ISERDD **SP1-NARRATIVE** QuTub Project

Rekha (female)

Rekha is a 35 year old female who has studied up to 10th standard. She supplements her family income by stitching clothes at home. She is a little worried today as she has cough and running mild fever and thus does not feel like doing work. Her husband suggests that she should go and see a doctor to day about it. He leaves for work at his tea stall. Rekha's is a small family unit with her husband and two children aged six (daughter) and four (son) and they live in a two room house. She has been stitching clothes since a few years as her husband's work does not generate enough income and with her work she is able to earn an extra 2000 -3000 rupee in a month. Generally Rekha has been in good health and has not had any major health problems or any chronic Illness. Her husband and children too have had good health. But since last 2-3 weeks she has been having cough which is more or less present during early morning and night, and also has expectoration that does not have any color in it and is clear. She is also running low grade mild fever, on and off, which gets worse during the evening time. But since this problem started she feels tired and also has lost some weight, as her clothes have got bit loose. She does not suffer from any associated chest or body pain. Around 1 week ago, she had visited a local chemist who gave her a cough syrup and some pills for the fever. Rekha has a cheerful nature and she abstains from alcohol and smoking. Her relationship with her husband is good and she loves him very much but today she is worried about her cough and fever and visits a doctor nearby

रेखा (महिला)

रेखा पढ़ी–लिखी दसवीं पास 35 साल की महिला है। वह अपने घर के खर्च को पूरा करने के लिये घर में कपड़े सिलाई का काम करती है। आज रेखा का मन काम करने का नहीं कर रहा था क्योंकि आज भी उसे खाँसी, हल्का बुखार और कमजोरी महसूस हो रही थी। यह बात उसने अपने पति को बताई तो उसके पति ने कहा कि तुम आराम करो और आज किसी डॉक्टर को जरुर दिखा लेना। यह कह कर उसका पति अपनी चाय की दुकान पर चला गया।

रेखा के घर में उनके पति और दो बच्चे है। लड़की की उम्र 6 साल और लड़के की 4 साल है। रेखा दो कमरे के खुद के मकान में रहती है। रेखा कुछ सालों से घर पर सिलाई का काम कर रही है ,क्योंकि उसके पति की चाय की दुकान से इतनी कमाई नहीं हो पाती और वह अपने काम से महीने में 2 से 3 हजार रूपये कमा लेती है।

आमतौर पर रेखा का स्वास्थ्य अच्छा रहता है, उसे किसी भी तरह की तकलीफ और कोई लम्बी बिमारी नहीं है। उसके पति और बच्चों का स्वास्थ्य भी अच्छा है। लेकिन रेखा को पिछले 2–3 हफ्तों से खाँसी है, जो सुबह और रात के समय ज्यादा होती है। उसकी खाँसी के साथ बलगम भी आता है जिसका कोई रंग नहीं है, वह साफ है। उसे हल्का बुखार रहता है जो चढ़ता–उतरता है लेकिन अक्सर शाम के समय ही ज्यादा होता है। जब से उसे यह तकलीफ शुरु हुई है तब से उसे थकावट महसूस हो रही है। उसे लगता है कि उसका कुछ वजन कम हो गया है क्योंकि उसके कपड़े ढीले हो गये है। उसे इस तकलीफ में किसी भी तरह का छाती का दर्द और बदन दर्द नहीं है। एक हफ्ता पहले रेखा ने इस तकलीफ के लिये घर के नजदीक के कैमिस्ट से दवा ली थी। कैमिस्ट ने खाँसी का सीरप और बुखार के लिये कुछ गोलियाँ दी थी।

रेखा हंसमुख और मिलनसार स्वभाव की महिला है और वह किसी भी प्रकार का कोई नशा नहीं करती। उसके अपने पति के साथ अच्छे सम्बन्ध है। वह उसे बहुत प्यार करती है। लेकिन आज उसके चेहरे पर अपनी खाँसी और बुखार को लेकर थोड़ी परेशानी है जिसको लेकर वह नजदीक डॉक्टर के पास गयी है।

ISERDD **SP1-NARRATIVE** QuTub Project

Opening statement: **“Doctor, I have cough and fever that is not getting better”**

ओपनिंग स्टेटमेंट : डॉक्टर साहब, “मुझे खाँसी बहुत हो रही है और साथ में बुखार भी है, जो ठीक ही नहीं हो रहा है”

History questions asked by the provider and their answers

प्रोवाइडर द्वारा पूछे गये हिस्ट्री सवाल और उनके जवाब

Q 1: What is the duration of cough?
प्रश्न 1: खाँसी कब से हो रही है?
Ans1 : 2-3 weeks, more during early morning and night
उत्तर 1: 2–3 सप्ताह से, यह सुबह–सुबह और रात को ही ज्यादा होती है।

Q 2: Are you producing sputum (bulgam)?
प्रश्न 2: क्या आपको बलगम बनती है?
Ans 2: Yes
उत्तर 2: हाँ।

Q 3: Does the sputum contain blood?
प्रश्न 3: क्या आपके बलगम में खून आता है?
Ans 3: No
उत्तर 3: नहीं।

Q 4: How long have you had fever?
प्रश्न 4: आपको बुखार कब से है?
Ans 4: Since 2-3 weeks
उत्तर 4: 2–3 हफ्ते हो गये।

Q 5: What type of fever do you have?
प्रश्न 5: बुखार कैसा रहता है?
Ans 5: Low grade (mild), on and off, more during evening times.
उत्तर 5: हल्का बुखार चढ़ता उतरता रहता है, लेकिन ज्यादातर शाम को होता है।

Q 6: Have you taken any medicines for your illness?
प्रश्न 6: क्या आपने इस तकलीफ के लिये कोई दवाई ली है?
Ans 6: Went to a local chemist who gave cough syrup and some pills for fever.
उत्तर 6: नजदीक के कैमिस्ट के पास गया था उसने मुझे खाँसी का सिरप और बुखार के लिये कुछ गोलियाँ दी थी।

Q 7: Do you get any chest pain?
प्रश्न 7: क्या आपकी छाती में दर्द होता है?
Ans 7: No
उत्तर 7: नहीं।

Q 8: Any loss of appetite?

A41

प्रश्न 8: भूख में कोई कमी?
Ans 8: Yes, loss of appetite.
उत्तर 8: हाँ, भूख तो कम लगती है।

Q 9: Have you lost weight?
प्रश्न 9: क्या वजन कम हुआ है?
Ans 9: I think so; my clothes have become a bit loose.
उत्तर 9: हाँ, मुझे लगता है क्योंकि मेरे कपड़े ढीले हो गये हैं।

Q 10: Any wheezing or difficulty in breathing?
प्रश्न 10: साँस लेने में कोई तकलीफ/सीटी जैसी आवाज?
Ans 10: No
उत्तर 10: नहीं।

Q 11: Do you smoke?
प्रश्न 11: क्या आप बीड़ी/सिगरेट पीते हैं?
Ans 11: Yes, I smoke beedis. [in case of male SP]
उत्तर 11: हाँ, मैं बीड़ी पीता हूँ। (मेल SP के लिये)
No [in case of females SP] नहीं (फीमेल SP के लिये)

Q 12: How many beedis in a day?
प्रश्न 12: एक दिन में कितनी पीते हो?
Ans 12: 4-5 beedis, I guess
उत्तर 12: अंदाजन, 4 से 5 बीड़ी।

Q 13: Since when have you been smoking beedis?
प्रश्न 13: कब से बीड़ी पी रहे हो?
Ans 13: Since the last 8 or 10 years
उत्तर 13: पिछले 8 या 10 सालों से।

Q 14: Do you drink alcohol?
प्रश्न 14: क्या आप शराब पीते हैं?
Ans 14: Yes [in case of male] No [in case of female SP]
उत्तर 14: हाँ (मेल SP के लिये), नहीं (फीमेल SP के लिये)

Q 15: How often do you drink?
प्रश्न 15: कितनी बार पी लेते हो?
Ans 15: Once or twice in a month. [in case of male] No [in case of female SP]
उत्तर 15: महीने में एक–दो बार। (मेल SP के लिये), नहीं (फीमेल SP के लिये)

Q 16: Have you been treated for TB in the past?
प्रश्न 16: क्या आपने पहले कभी टी बी का इलाज कराया है?
Ans 16: No
उत्तर 16: नहीं तो।

Q 17: Anyone in your family has TB?
प्रश्न 17: क्या आपके घर में किसी को टीबी है?
Ans 17: No
उत्तर17: नहीं।

Q 18: Do you have diabetes?
प्रश्न 18: क्या आपको शुगर है?
Ans 18: I do not know
उत्तर 18: जी, पता नहीं।

Q 19: Do you have hypertension?
प्रश्न 19: क्या आपको हाईपरटेंशन है?
Ans 19: I do not know
उत्तर 19: जी, पता नहीं।

Q 20: Do you have HIV-AIDS?
प्रश्न 20: क्या आपको HIV-AIDS है?
Ans 20: I do not know
उत्तर 20: जी, पता नहीं।

Q 21: Have you ever been tested for these diseases?
प्रश्न 21: क्या आपने कभी इन बिमारियों कि जाँच या टेस्ट करवाया है?
Ans21: Not been tested
उत्तर 21: कभी टेस्ट नहीं करवाया।

Q 22: Have you ever been tested for these diseases?
प्रश्न 22: क्या आपको किसी दवाई से एलर्जी है?
Ans22: No.
उत्तर 22: नहीं।

ISERDD SP1-NARRATIVE QuTub Project

Important instructions to be remembered by SP

महत्वपूर्ण बातें जो एस पी को याद रखनी है

1.	SP must remember if the provider carried out any of the following examination?<br>SP को याद रखना है कि प्रोवाइडर ने निम्नलिखित में से कोई परीक्षण किये?<br>• Pulse rate नब्ज़ की दर<br>• Respiratory rate साँस की दर<br>• Auscultation of Chest ह्रदय, फेफड़ों की गति को सुनना<br>• Blood Pressure ब्लड प्रेशर<br>• Temperature बुखार मापना – थर्मामीटर<br>• Throat examination गले का परीक्षण<br>• Weight वजन मापना
2.	Did the provider recommend any investigations? क्या प्रोवाइडर ने निम्नलिखित जाँच कराने को कहा?<br>(If yes, SP should ask provider to write the name of the test and the laboratory. And hand over the document to an ISERDD staff.) *Write the specific name of lab given by the provider and if no put (-99).*<br>(यदि हाँ, तो SP को प्रोवाइडर से टेस्ट और लैब का नाम लिखित में लेना है और उस पर्चे को ISERDD स्टॉफ को सौंप देना है) *अगर प्रोवाइडर ने किसी विशेष लैब का नाम दिया है तो वह लिखें, यदि नहीं तो (-99) भरें।*<br>• Chest X-Ray छाती का एक्स-रे<br>• CT Scan सी टी स्केन<br>• Blood- Total count, differential count- ESR रक्त-टोटल काउंट, डिफरेंशियल काउंट-ई एस आर<br>• Blood- HIV test रक्त-एच आईवी टेस्ट<br>• Blood- Diabetes test रक्त-शुगर टेस्ट<br>• Blood- TB Gold रक्त – टी बी गोल्ड<br>• Blood-TB ELISA रक्त – टी बी ऐलाइजा<br>• Sputum smear examination (Sputum AFB) स्पूटम स्मियर एक्जामिनेशन<br>• Sputum GeneXpert test स्पूटम जीनएक्सपर्ट टेस्ट<br>• Sputum culture स्पूटम कल्चर<br>• Mantoux Test मॉनटुक्स टेस्ट<br>• Drug susceptibility test ड्रग स्सेप्टिबिलिटी टेस्ट
3.	ISERDD staff to mark which of the following tests was recommended.<br>जो भी टेस्ट करवाने के लिए बोला गया है उसे ISERDD स्टॉफ को फार्म में मार्क करना है।
4.	SP must collect prescription and/or any medicines given by the provider<br>SP को प्रोवाइडर द्वारा दी गयी दवाई या दवाई का पर्चा अवश्य लेना है।
5.	SP must remember if the provider gave any diagnosis.<br>SP को प्रोवाइडर द्वारा दिये गये डाइग्नोसिस को याद रखना है।
6.	Prescriptions and pills given must be preserved for analysis.<br>SP को प्रोवाइडर द्वारा दिया गया पर्चा और दवाईयाँ एनालिसिस के लिये संभाल कर रखना है।
7.	SP must remember if the provider recorded the information he took from you.<br>SP को यह ध्यान रखना है कि प्रोवाइडर ने आपसे जो जानकारी ली उसको कहीं लिखकर रखा।
8.	SP Should get the prices, brand and generic names of the prescribed medicines from the chemist.<br>SP को प्रोवाइडर द्वारा पर्चे पर लिखी दवाईयों का सही मूल्य, ब्रॉन्ड और जेनेरिक नाम कैमिस्ट से पता करना है
9.	ISERDD staff will identify the provider clinic/chemist for the SP, where the SP will present his case alone.<br>ISERDD स्टाफ फील्ड में SP को प्रोवाइडर का क्लीनिक/कैमिस्ट बतायेगा जहाँ SP को अकेले अपना केस करना है।
10.	SP should refuse any injections/ invasive tests performed by the provider during this encounter but note down details of what was offered/suggested.<br>SP को इस बात चीत के दौरान किसी भी तरह का इन्जेक्शन/इन्वेसिव टेस्ट लेने से इन्कार करना है लेकिन ऐसे किसी भी सुझाव को नोट करके बताना है।

ANNEX K. SP EXIT QUESTIONNAIRE – SAMPLE FROM QUTUB PROJECT (SECTIONS 6.2, 6.5)

Source: Qutub project pilot in Delhi
Notes: 9-page exit interview for male and female classic case of suspected tuberculosis (2-3 week cough and fever)

To use this annex as a whole, or in part, please cite:

- Kwan A, Bergkvist S, Daniels B, Das J, Das V, Pai M. Using Standardized Patients to Measure Health Care Quality: A Manual and Toolkit for Projects in Low- and Middle-Income Countries. 2019.
- Das J, Kwan A, Daniels B, Satyanarayana S, Subbaraman R, Bergkvist S, Das RK, Das V, Pai M. Use of standardised patients to assess quality of tuberculosis care: a pilot, cross-sectional study. The Lancet Infectious Diseases. 2015 Nov 30;15(11):1305-13.

Kwan A, *et al. BMJ Global Health* 2019; 4:e001669. doi: 10.1136/bmjgh-2019-001669

ISERDD ***SP1_ EXIT QUESTIONNAIRE: "Ravi/Rekha"***

Provider ID : **Form No:**

1	City Name शहर का नाम	दिल्ली DELHI	2	City शहर की ID	
3	Clinic Name & Address<br>क्लीनिक का नाम और पता	*English*			
4	Clinic ID<br>क्लीनिक आई डी				
5	Provider Qualification<br>प्रोवाइडर की डिग्री	No Degree=01, RMP=02, BAMS=03, BIMS=04, BUMS =05, BHMS/DHMS=06 BEHMS/BEMS=07, MBBS=08, MBBS+MD=09, Chemist=10 Other (*Specify*) =11			
6	Provider Name<br>प्रोवाइडर का नाम	*English*			
7	Provider ID<br>प्रोवाइडर की ID				

	Visits विज़िटस्	Visit-1 पहला विज़िट		Visit-2 दूसरा विज़िट		Visit-3 तीसरा विज़िट	
8	Date of survey for each visit<br>सर्वे की तारीख हर विजिट के लिये	*DD /MM/YYYY*		*DD/MM/YYYY*		*DD/MM/YYYY*	
9	SP Name SP का नाम	----------------------------------		----------------------------------		----------------------------------	
10	SP ID आई डी						
11	Total time spent at the Provider Clinic<br>In time (Railway time)<br>प्रोवाइडर के क्लीनिक में बिताया गया कुल समय (रेलवे समय)	In time<br>अन्दर जाने का समय<br>Hrs घंटे<br>Min मिनट	Out time<br>बाहर आने का समय<br>Hrs घंटे<br>Min मिनट	In time<br>अन्दर जाने का समय<br>Hrs घंटे<br>Min मिनट	Out time<br>बाहर आने का समय<br>Hrs घंटे<br>Min मिनट	In time<br>अन्दर जाने का समय<br>Hrs घंटे<br>Min मिनट	Out time<br>बाहर आने का समय<br>Hrs घंटे<br>Min मिनट
12	Completion of the case.<br>केस पूरा हुआ *Yes=1, No=2*						
12a	If no, give reason.<br>यदि नहीं, कारण दीजिए।						
	Reason for non- Completion of the case:- यदि केस पूरा नहीं हुआ तो कारण बतायें :-<br>Waited 3 hrs. 3 घंटे इन्तजार किया = 1; Provider in town but not coming to clinic प्रोवाइडर शहर में है लेकिन क्लीनिक में नहीं आया=2 Provider out of town प्रोवाइडर शहर से बाहर है = 3; Clinic locked क्लीनिक बन्द था = 4; Provider no longer practicing प्रोवाइडर ने प्रैक्टिस छोड़ दी =5; Provider migrated प्रोवाइडर ने एरिया छोड़ दिया =6						

13	Do you know if you saw the sampled Provider?<br>क्या आपको पता है कि आपने सेम्पल प्रोवाइडर को ही दिखाया?	*Enter 1=Yes; 2= No; 3=don't know*			
14	Interviewer Name<br>साक्षात्कारकर्ता का नाम		14a	Interviewer ID<br>साक्षात्कारकर्ता की आई डी	
15	जब आप क्लीनिक में पहुँचे तब कितने रोगी इन्तजार कर रहे थे?	How many patients were waiting when you reached the clinic?			
16	जब आप क्लीनिक से बाहर निकले तब कितने रोगी बाकी थे?	How many patients were in the clinic when you left?			

ISERDD ***SP1_ EXIT QUESTIONNAIRE: "Ravi/Rekha"***

Provider ID : ☐☐☐☐☐☐☐ **Form No:** ☐☐☐

NO.	QUESTION (HINDI)	QUESTION (ENGLISH)	*Asked-* YES (1) NO (2)	If not *asked-* given by SP? YES (1) NO (2)
**SECTION 1:**	**ESSENTIAL HISTORY INFORMATION TAKEN BY THE PROVIDER**			
H1	क्या प्रोवाइडर ने खाँसी की अवधि के बारे में पूछा?	Did the provider ask about duration of cough?	☐	☐
H2	क्या प्रोवाइडर ने पूछा कि बलगम बनता है?	Did the provider ask whether sputum is produced?	☐	☐
H3	क्या प्रोवाइडर ने पहले कभी आपको टी बी हुई है इसके बारे में जानकारी ली?	Did the provider ask if you had TB in the past?	☐	☐
H4	क्या प्रोवाइडर ने परिवार में किसी को टी बी है इसके बारे में जानकारी ली?	Did the provider ask about history of TB in the family?	☐	☐
	**RECOMMENDED INFORMATION TAKEN BY THE PROVIDER**			
H5	क्या प्रोवाइडर ने थूक में खून के बारे में पूछा?	Did the provider ask about Blood in the sputum?	☐	☐
H6	क्या प्रोवाइडर ने पूछा कि आपकी खाँसी सारा दिन रहती है?	Did the provider ask that do you have cough throughout the day?	☐	☐
H7	क्या प्रोवाइडर ने बुखार के बारे में पूछा?	Did the provider ask about Fever?	☐	☐
H8	क्या प्रोवाइडर ने बुखार का प्रकार (हल्का या तेज) पूछा?	Did the provider ask about type of fever (low grade vs high grade)	☐	☐
H9	क्या प्रोवाइडर ने परिवार के सदस्यों की जानकारी और परिवार में किसी को इस तरह के लक्षण के बारे में पूछा?	Did the provider ask about family members and similar symptoms in the family	☐	☐
H10	क्या प्रोवाइडर ने छाती में दर्द के बारे में पूछा?	Did the provider ask about chest pain?	☐	☐
H11	क्या प्रोवाइडर ने भूख में कमी के बारे में पूछा?	Did the provider ask about any loss of appetite?	☐	☐
H12	क्या प्रोवाइडर ने वजन के कम होने के बारे में पूछा?	Did the provider asked have you lost weight?	☐	☐
H13	क्या प्रोवाइडर ने साँस लेने् में सीटी जैसी आवाज के बारे में पूछा?	Did the provider ask about any wheezing?	☐	☐
H14	क्या प्रोवाइडर ने साँस लेने् में कोई तकलीफ के बारे में पूछा?	Did the provider ask about any difficulty in breathing?	☐	☐
H15	क्या प्रोवाइडर ने बीड़ी/सिगरेट के बारे में पूछा?	Did the provider ask about anything about smoking?	☐	☐
H16	क्या प्रोवाइडर ने शराब के बारे मे पूछा?	Did the provider ask anything about alcohol history?	☐	☐
H17	क्या आपने इस तकलीफ के लिये कोई दवाई ली है?	Have you taken any medicines for your illness?	☐	☐
H18	क्या प्रोवाइडर ने शुगर के बारे मे पूछा?	Did the provider ask anything about Diabetes?	☐	☐
H19	क्या प्रोवाइडर ने एच आई वी-एड्स के बारे मे पूछा?	Did the provider ask anything about HIV-AIDS?	☐	☐
H20	क्या प्रोवाइडर ने हाई ब्लड प्रेशर या हाईपरटेंशन के बारे में पूछा?	Did the provider ask anything about high blood pressure or hypertension?	☐	☐

ISERDD ***SP1_ EXIT QUESTIONNAIRE: "Ravi/Rekha"***

Provider ID : ☐☐☐☐☐☐☐ **Form No:** ☐☐☐

H21	क्या प्रोवाइडर ने आपकी उम्र पूछी?	Did the provider ask your age?	☐ ☐
H22	प्रोवाइडर ने आपसे जो जानकारी ली उसको कहीं लिखकर रखा। *हाँ=1, नहीं=2*	The provider recorded the information he took from you. *Yes=1, No=2*	☐

**SECTION 2 : CLINICAL EXAMINATION CONDUCTED BY THE PROVIDER**			**Yes (1) No (2)**
E1	नब्ज़ की दर	Pulse rate	☐
E2	ह्रदय, फेफड़ों की गति को सुनना	Auscultation	☐
E3	बुखार मापना – थर्मामीटर	Temperature - thermometer	☐
E4	गले का परीक्षण	Throat examination	☐
E5	ब्लड प्रेशर मापा	Blood Pressure	☐
E6	वजन मापना	Weight	☐
**RECOMMENDED INVESTIGATIONS ORDERED BY THE PROVIDER**			
अगर प्रोवाइडर कोई टेस्ट करवाने के किसी पैथ लैब का नाम बताता है तो उसका नाम लिखें, यदि किसी पैथ लैब का नाम नहीं बताता है तो -99 भरें<br>*Write the name of the path lab from where the provider recommended the test, if no name was given put -99.*			
E7	छाती का एक्स-रे Chest X-Ray		☐
E8	सी टी स्केन CT Scan		☐
E9	स्पूटम स्मियर एक्जामिनेशन<br>Sputum smear examination (Sputum AFB)		☐
E10	स्पूटम जीनएक्सपर्ट टेस्ट<br>Sputum-GeneXpert test		☐
E11	स्पूटम कल्चर और ड्रग स्सेप्टिबिलिटी टेस्ट<br>Sputum culture test and Drug susceptibility test		☐
E12	रक्त – टोटल काउंट, डिफरेंशियल काउंट-ई एस आर<br>Blood -Total Count, Differential Count- ESR		☐
E13	रक्त – एच आईवी टेस्ट Blood- HIV test		☐
E14	रक्त – शुगर टेस्ट Blood- Diabetes test		☐
E15	रक्त – टी बी गोल्ड Blood-TB Gold		☐
E16	रक्त – टी बी ऐलाइजा Blood-TB Elisa		☐
E17	मॉनटूक्स टेस्ट Mantoux test		☐

ISERDD ***SP1_ EXIT QUESTIONNAIRE: "Ravi/Rekha"***

Provider ID : ☐☐☐☐☐☐☐ **Form No:** ☐☐☐

**SECTION 3 : DIAGNOSIS**			**Yes (1)**<br>**No (2)**
**D1**	क्या प्रोवाइडर ने बातचीत में टी बी होने की शंका जताई?	Did the provider mention about suspicion of TB in the whole conversation?	☐
**D2**	क्या प्रोवाइडर ने खुद से कोई पर्चा लिख कर दिया?<br>यदि हाँ, तो D2a & D2b भरें। यदि नहीं, तो D3 पर जायें	Did the provider give a prescription on his/her own? If yes, fill D2a & D2b. If no, go to D3	☐
**D2a**	क्या वह पर्चा दवाईयों के लिये था?	Was the prescription for medicines?	☐
**D2b**	क्या वह पर्चा डायग्नोस्टिक जाँच के लिये था?	Was the prescription for diagnostic test?	☐
**D3**	प्रोवाइडर ने क्या डायग्नोसिस दिया?	Did the provider give a diagnosis?	☐
**D3a**	यदि हाँ, तो डायग्नोसिस क्या था? (यदि, डायग्नोसिस एक या एक से अधिक दिये गये तो सभी को लिखें)<br>If yes, what was the diagnosis? (if, one or more diagnosis given then write all of them)	____	

ISERDD ***SP1_ EXIT QUESTIONNAIRE: "Ravi/Rekha"***

Provider ID : ☐☐☐☐☐☐☐ **Form No:** ☐☐☐

SECTION 4: TREATMENT (In case to be taken SOS code -66 in frequency)

T1	Total time taken by Provider<br>प्रोवाइडर द्वारा लिया गया कुल समय	HH ☐☐ : MM ☐☐ : SS ☐☐
T2	Did the provider dispensed/prescribed any medicine? If yes, fill T3, if no go to T4<br>क्या प्रोवाइडर ने आपको दवाई दी/लिखकर दी? यदि हाँ, तो T3 भरें। यदि नहीं, तो T4 पर जाये	☐

T3	Medicines दवाईयाँ *Dispensed* दवाई दी = *1* *Prescribed* दवाई लिखी =*2*	(a) Name नाम If provider has prescribed/Dispensed less than 6 medicines than write -99 in the medicine name. यदि प्रोवाइडर ने 6 से कम दवाईयाँ दि है तो मेडिसिन नाम में -99 भरें	(b) Types of Medicine दवाईयों का प्रकार Tablets गोली=1, Capsules केप्सूल=2, Syrups सिरप=3, Injectables इंजेक्टेबल=4 Powder चुरण=5	(c) Dose खुराक	(d) Frequency दिन में कितनी बार	(e) Duration कितने दिनों तक	(f) How many days in week हफ्ते में कितनी बार	(g) How many weeks कितने हफ्ते	(h) Drug classification code दवा का कोड refer to annex	(i) Price for full course of prescribed medicine? पर्चे पर लिखी दवाई की कीमत
	1. ☐	ब्रॉण्ड Brand<br>जेनेरिक Generic	☐	☐☐:☐☐	☐☐	☐☐	☐	☐☐	☐☐	?
	2. ☐	ब्रॉण्ड Brand<br>जेनेरिक Generic	☐	☐☐:☐☐	☐☐	☐☐	☐	☐☐	☐☐	?
	3. ☐	ब्रॉण्ड Brand<br>जेनेरिक Generic	☐	☐☐:☐☐	☐☐	☐☐	☐	☐☐	☐☐	?
	4. ☐	ब्रॉण्ड Brand<br>जेनेरिक Generic	☐	☐☐:☐☐	☐☐	☐☐	☐	☐☐	☐☐	?
	5. ☐	ब्रॉण्ड Brand<br>जेनेरिक Generic	☐	☐☐:☐☐	☐☐	☐☐	☐	☐☐	☐☐	?
	6. ☐	ब्रॉण्ड Brand<br>जेनेरिक Generic	☐	☐☐:☐☐	☐☐	☐☐	☐	☐☐	☐☐	?

Annex to T3 (h) Drug classification code :- Unlabelled Tablets/Syrup खुली/बिना नाम की गोलियाँ/सिरप=1; Unlabelled injections खुली या बिना नाम का इंजेक्शन =2; IV bottles/glucose drip आई वी बोतल/ग्लूकोस ड्रिप =3; Ayurvedic medicines आयुर्वेदिक दवाईयाँ =4; Homeopathic medicines होम्योपेथिक दवाईयाँ =5; Antibiotics ऐन्टिबायोटिक दवाईयाँ =6; Analgesics ऐनालजेसिक दवाईयाँ =7; Anti-ulcer medication ऐन्टी-अल्सर दवाईयाँ =8; Steroids (NSAIDS) स्टेरॉयड्स =9; Anti-allergy medicines ऐन्टी-अलर्जिक दवाईयाँ =10; Cardiac medication कारडियक दवाईयाँ =11 ; Psychiatric/ neural medicines साईकाईट्रिक दवाईयाँ =12; Identified as anther type of medication =13; Household remedies घरेलू दवाईयाँ =14; Drugs not classified दवाई जो क्लासिफाइड नहीं है = 50

ISERDD ***SP1_ EXIT QUESTIONNAIRE: “Ravi/Rekha”***

Provider ID : ☐☐☐☐☐☐☐ **Form No:** ☐☐☐

T4	आपने प्रोवाइडर को कुल कितने पैसे दिये?<br>How much money did you give at end of consultation?	Rs. ☐☐☐☐	
T5	क्या प्रोवाइडर ने कोई इंजेक्शन लेने के लिये कहा?	Did the provider offer any Injection?	☐
T6	क्या प्रोवाइडर ने आई वी लेने के लिये कहा?	Did the provider offer an IV?	☐
T7	क्या प्रोवाइडर ने कोई अन्य इनवेसिव जाँच के लिये कहा, जैसे कि ब्लड ग्लूकोज़ टेस्ट? यदि हाँ तो T7a भरें, यदि नहीं, तो T8 पर जायें।	Did the provider offer any other invasive exams, such as blood glucose test? If yes go to T6a. if no, go to T7	☐
T7a	यदि हाँ, तो प्रोवाइडर ने कौन सा इनवेसिव टेस्ट किया/सलाह दी?<br>If yes, what invasive test was offered/suggested?	______________________________	
T8	क्या प्रोवाइडर ने बीमारी के किसी खतरे के निशान के बारे में बताया?	Did the provider inform about any danger signs of the disease?	☐
T9	क्या प्रोवाइडर ने दवाई के साईड इफैक्ट (मिचली, उल्टी, पेशाब का लाल होना) के बारे में बताया?	Did the provider inform about any side effects of drugs? (nausea, vomiting, red discoloration of the urine)	☐
T10	क्या प्रोवाइडर ने खाँसते समय किसी प्रकार की सावधानी बरतने के बारे में बताया?	Did the provider speak about cough hygiene?	☐
T11	क्या प्रोवाइडर ने धूम्रपान बंद करने की सलाह दी?	Did the provider speak about smoking cessation?	☐
T12	क्या प्रोवाइडर ने वापस आने की सलाह दी?<br>यदि हाँ, तो T12a से T12f भरे। यदि नहीं, तो T13 पर जायें।	Did the provider ask the patient to come back?<br>If yes, mark from T12a to T12f and if no, go to T13	☐
T12a	लक्षणों में कोई सुधार नहीं	If the symptoms persist	☐
T12 b	लक्षण और बिगड़ जायें	If the symptoms become worse	☐
T12c	दवाई लेने के लिये	To get medicines	☐
T12d	टेस्ट रिपोर्ट दिखाने के लिए	To show the test results	☐
T12e	अन्य	Other	☐
T12f	विवरण______________________________	Specify______________________________	

T13	**Any other questions asked that were not on the previous list?**<br>ऊपर दिये गये सवालों के अलावा कोई नये सवाल आपसे पूछे गये?<br>☐<br>*1 =Yes* हाँ, *2 =No* ना	1	*हिन्दी*<br>*English*
		2	*हिन्दी*<br>*English*
		3	*हिन्दी*<br>*English*
		4	*हिन्दी*

ISERDD ***SP1_ EXIT QUESTIONNAIRE: "Ravi/Rekha"***

Provider ID : ☐☐☐☐☐☐☐ **Form No:** ☐☐☐

**SECTION 5: REFERRAL**			**Yes (1)**<br>**No (2)**
R	क्या प्रोवाइडर ने रोगी को आगे की देखभाल के लिए कहीं जाने की सलाह दी? यदि हाँ तो R1 से R4a भरें। यदि नहीं, तो सेक्शन 6 पर जायें।	Did the provider ask the patient to go anywhere for further management? If yes fill R1 to R4a, If no, go to section 6.	☐
R1	प्राइवेट प्रोवाइडर/प्राइवेट अस्पताल	Private Provider/ Private Hospital	☐
R2	सरकारी अस्पताल	Government Hospital	☐
R3	डॉट्स सेन्टर	DOTS Centre	☐
R4	अन्य	Other	☐
R4a	विवरण ______________________________	Specify ______________________________	

**SECTION 6: GLOBAL ASSESSMENT SCALE**			
G1	Did you like this doctor?<br>क्या आपको डॉक्टर अच्छा लगा ?	*1 =Yes* हाँ, *2 =No* ना	☐
G2	Would you go to this doctor again?<br>क्या आप इस डॉक्टर के पास दोबारा जाओगे?	*1 =Yes* हाँ, *2 =No* ना	☐
G3	Did the doctor create an environment in which you could convey your symptoms and concerns easily?<br>क्या डॉक्टर ने ऐसा माहौल बनाया कि आप उसे अपनी तकलीफ आसानी से बता सकें?	Definitely निश्चित रुप से =3<br>Somewhat थोड़ा सा =2<br>Not at all बिल्कुल नहीं =1	☐
G4	Did the doctor appear to be knowledgeable about your illness?<br>आपको क्या लगा क्या यह डॉक्टर अच्छे जानकार हैं। क्या आप समझते है कि उन्हें आपकी बीमारी की जानकारी है?	Very knowledgeable अच्छी जानकारी है =3<br>Somewhat knowledgeable सामान्य जानकारी है =2<br>Not at all बिल्कुल नहीं =1	☐
G5	Did the doctor address your worries seriously?<br>क्या आपकी चिन्ता पर डॉक्टर ने पूरा ध्यान दिया?	Very seriously पूरा ध्यान दिया =3<br>Somewhat seriously थोड़ा ध्यान दिया =2<br>Not at all बिल्कुल नहीं =1	☐
G6	Did the doctor explain anything about your illness?<br>क्या डॉक्टर ने आपको बीमारी के बारे में समझाया?	Very well बहुत अच्छी तरह से =3<br>Cursorily थोड़ा सा =2<br>Not at all बिल्कुल नहीं =1	☐
G7	Did the doctor explain your treatment plan?<br>क्या डॉक्टर ने आपको इलाज के बारे में समझाया?	Very well बहुत अच्छी तरह से =3<br>Cursorily थोड़ा सा =2<br>Not at all बिल्कुल नहीं =1	☐
G8	The SP will give a rank to the provider from 1-10, where 10 is the highest and 1 the lowest.<br>SP प्रोवाइडर को 1 से 10 रेंक दे जिसमें 10 सबसे अधिक और 1 सबसे कम है।		☐☐

ISERDD ***SP1_ EXIT QUESTIONNAIRE: "Ravi/Rekha"***

Provider ID : ☐☐☐☐☐☐☐ **Form No:** ☐☐☐

**SECTION 7:** ***Errors and Detection गलतियाँ और पहचाना गया***			
1.	क्या आपको लगता है कि केस प्रेजेंट करने में आपसे कोई गलती हुई? यदि हाँ, तो कौन सी गलती हुई। उनको नीचे लिखें। *हाँ=1, नहीं=2*	Did you think you made any mistakes in the presentation of the case? If yes, what mistakes you made please note them down. *Yes=1, No=2*	☐
2.	क्या आपको प्रोवाइडर ने एक एस पी के रूप में पहचान लिया? यदि हाँ, तो आप कैसे पहचाने गये? *हाँ=1, नहीं=2*	Did the provider detect you as an SP? If yes, how were you detected? *Yes=1, No=2*	☐

Supervision Check सुपरवाइजर चैक			
Supervisor's Name सुपरवाईजर का नाम		Supervisor ID सुपरवाईजर की ID	☐☐
Form checking date फॉर्म चैक करने की तारीख	☐☐/☐☐/☐☐☐☐ *DD/MM/YYYY format*		
Recorder No रिकार्डर नम्बर			☐

Kwan A, *et al. BMJ Global Health* 2019; 4:e001669. doi: 10.1136/bmjgh-2019-001669

ISERDD ***SP1_ EXIT QUESTIONNAIRE: "Ravi/Rekha"***

Provider ID : | | | | | | | | **Form No:** | | | |

Comments/ टिप्पणी :

Kwan A, *et al. BMJ Global Health* 2019; 4:e001669. doi: 10.1136/bmjgh-2019-001669

ANNEX L. FOLLOW-UP DETECTION SURVEY AND VIGNETTE – SAMPLE FROM QUTUB PROJECT (SECTIONS 6.6, 6.7)

Source: Qutub project pilot in Delhi
Notes: 19-page follow-up detection survey and vignette corresponding to case in Annexes F and G (male and female classic case of suspected tuberculosis with 2-3 week cough and fever)

To use this annex as a whole, or in part, please cite:

- Kwan A, Bergkvist S, Daniels B, Das J, Das V, Pai M. Using Standardized Patients to Measure Health Care Quality: A Manual and Toolkit for Projects in Low- and Middle-Income Countries. 2019.
- Das J, Kwan A, Daniels B, Satyanarayana S, Subbaraman R, Bergkvist S, Das RK, Das V, Pai M. Use of standardised patients to assess quality of tuberculosis care: a pilot, cross-sectional study. The Lancet Infectious Diseases. 2015 Nov 30;15(11):1305-13.

QuTub Study–Follow up Detection and Vignettes Module   Provider ID: ☐☐☐☐☐☐☐

Follow up Detection and Vignettes Module

Section : 0					
1	City Name शहर का नाम	दिल्ली DELHI	2	City शहर की ID	☐
3	Clinic Name<br>क्लीनिक का नाम	*English*			
4	Clinic Address<br>क्लीनिक का पता	*English*			
5	Clinic ID<br>क्लीनिक आई डी	☐☐☐☐☐☐☐			
6	Provider Name<br>प्रोवाइडर का नाम	*English*			
7	Provider ID<br>प्रोवाइडर की ID	☐☐☐☐☐☐☐			
8	Completion of the Interview.<br>केस इण्टरव्यू हुआ *Yes हाँ =1, No नहीं =2*	☐			
9	If the Interview was not Completed, give reason.<br>यदि इण्टरव्यू पूरा नहीं हुआ तो उसका कारण दो।<br>Provider could not be located<br>प्रोवाइडर को ढूँढ नहीं पाये =1<br>Provider refused to cooperate प्रोवाइडर ने सहयोग करने से मना कर दिया = 2<br>Provider has left<br>प्रोवाइडर ने छोड़ दिया = 3<br>Other specify अन्य विवरण दें = 4	☐<br>*Other specify अन्य विवरण :* ____________			
10	How many years have you been practicing?<br>आप कितने सालों से प्रैक्टिस कर रहे हैं?	☐☐			
11	How many years have you been practicing in this location?<br>आप इस क्लीनिक में कितने सालों से प्रैक्टिस कर रहे हैं?	☐☐			
12	How many patients do you see on average each day in your practice(s)?<br>आप औसतन एक दिन में कितने पेशन्ट देख लेते हैं?	☐☐			
13	Counting medicines and consulting fees, how much would you say that you charge for an average patient?<br>आप एक पेशन्ट से दवाईयों और कन्सल्टेशन की कितनी फीस लेते हैं?	*(a)Consultation fees with medicine* ☐☐☐<br>*(b)Consultation fees* ☐☐☐			
14	Interviewer Name & ID<br>साक्षात्कारकर्ता का नाम और आई डी	1		a	☐☐
		2		b	☐☐
15	Date of survey<br>सर्वे की तारीख	☐☐ ☐☐ ☐☐☐☐<br>DD MM YYYY			

QuTub Study–Follow up Detection and Vignettes Module Provider ID:

Section 1: Recognition of Standardized Patients स्टैंडर्डाइज़्ड पेशेन्ट की पहचान

Interviewer read aloud: We visited a few months ago to tell you that we might be sending you a standardized patient (someone very carefully trained to portray an actual patient). We would like to follow up with you to see if in fact you did receive any standardized patients since we told you about the study. Do note that you may have been visited by 1, 2 or 3 standardized patients, and in some cases you will *not* have been visited by any standardized patient at all, since there were some doctors who the standardized patients did not visit.

इन्टरव्यूवर पढ़े : हम कुछ महीने पहले आये थे और आपको बताया था कि हम आपके पास एक स्टैंडर्डाइज़्ड पेशेन्ट को भेजेंगे (ऐसा व्यक्ति जिसे एक असली रोगी की तरह व्यवहार करना सिखाया है)। इसी सिलसिले में हम आप से जानना चाहते है कि क्या ऐसा कोई स्टैंडर्डाइज़्ड पेशेन्ट आपके पास आया है जिस अध्ययन के बारे में हमने आपको बताया था। हो सकता है कि आपके पास एक, दो या तीन पेशेन्ट आये हों और यह भी हो सकता है कोई भी ऐसा पेशेन्ट आपके पास ना आया हो क्योंकि कुछ डॉक्टरों के पास ऐसे पेशेन्ट नहीं भेजे गये हैं।

1. Do you think that you received any standardized patients in your practice in the last 5 months?<br>क्या आपको लगता है कि पिछले 5 महीने में आपके पास कोई स्टैंडर्डाइज़्ड पेशेन्ट आया?<br>*1=Yes हाँ,2= No नहीं* (if no, skip to Section 2; यदि नही, सेक्शन 2 पर जायें)	

Please tell us a little about the individual(s) who you believe were standardized patients and that you received in the last 5 months.
कृप्या आप उन व्यक्तियों के बारे में बतायें जो आपको लगा कि वो स्टैंडर्डाइज़्ड पेशेन्ट थे और जिनको आपने पिछले 5 महीनों में देखा।

2	Serial Number क्रम संख्या	1	2	3	4	5
3	Gender of Standardized patient<br>स्टैंडर्डाइज़्ड पेशेन्ट का लिंग<br>1=Male पुरुष: 2=Female स्त्री					
4	Approximate Month of Visit<br>लगभग किस महीने में आया/आई<br>Enter in 3 Letters, for instance JAN, FEB, MAR, APR, MAY ETC.<br>इंगलिश महीने के नाम के केवल पहले 3 लेटर लिखे JAN, FEB, MAR, APR, MAY इत्यादि					
5	Approximate age of patient<br>रोगी की लगभग उम्र<br>1=Child बच्चा 2=Young Adult किशोर व्यस्क<br>3=Middle-aged मध्यम आयु 4=Old बुढ़ा					

A57

QuTub Study–Follow up Detection and Vignettes Module Provider ID:

6	Symptoms presenting with (do not prompt the options) किन लक्षणों के साथ (कृप्या विकल्प ना दें।) 1=Cough/Cold खाँसी/जुखाम 2=Fever बुखार 3=Diarrhea दस्त 4=Vomiting उल्टी 5=Weakness कमजोरी 6=Chest Pain छाती दर्द 7=Breathing problems साँस की परेशानी 8=Pain दर्द 9=Headache सिरदर्द 10=Skin Problem त्वचा की परेशानी 96=Other, specify अन्य विवरण दो______________	a.<br>b.<br>c.<br>____________	a.<br>b.<br>c.<br>____________	a.<br>b.<br>c.<br>____________	a.<br>b.<br>c.<br>____________	a.<br>b.<br>c.<br>____________
7	What was your diagnosis for this patient's condition? इस पेशेन्ट के लिए आपका डायग्नोसिस क्या था ?	__________	__________	__________	__________	__________
8	Did you think this individual was a standardized patient during his/her visit? क्या वह व्यावित आपको बातचीत के दौरान स्टैंडर्डाइज़ड पेशेन्ट लगा? *1=Yes हाँ, 2= No नहीं* (if no, skip to question 10; यदि नही, तो प्रश्न 10 पर जायें)					
9	What were the main signs that made you think that this was a standardized patient? आपको किन संकेतों से लगा कि वह एक स्टैंडर्डाइज़ड पेशेन्ट हैं? *(Do not prompt* ***कृप्या विकल्प ना बतायें)*** 1="Textbook case" किताबी केस 2=Refused to take injection इंजेक्शन लेने से मना करना 3=Refused to put thermometer in mouth to take the temperature तापमान मापने के लिए थर्मामीटर मुँह में रखने से मना करना 4=Did not look like a real patient असली रोगी की तरह नहीं लग रहा था 96=Other अन्य ______________ (For every other, please fill in what the doctor says in 1 line प्रत्येक अन्य के लिए कृप्या डॉक्टर का दिया गया जवाब एक वाक्य में लिखें) (Enter information, then skip to question 12. Do not answer questions 10 & 11 जानकारी भरने के बाद, कृप्या प्रश्न 12 पर जायें और प्रश्न 10 और 11 को छोड़ दें)					

QuTub Study–Follow up Detection and Vignettes Module Provider ID: |_|_|_|_|_|_|_|

10	Did you think this individual was a standardized patient after he left? (not during the visit)<br>क्या उस व्यक्ति के जाने के कुछ समय बाद आपको शक हुआ कि वह एक स्टैंडर्डाइज़्ड पेशेन्ट था? (देखने के वक्त नहीं)<br>*1=Yes हाँ, 2= No नहीं* (if no, skip to question 12 यदि नही, तो प्रश्न 12 पर जायें)	|_|	|_|	|_|	|_|	|_|
11	What were the main signs that made you think that this was a standardized patient?<br>आपको किन संकेतों से लगा कि वह एक स्टैंडर्डाइज़्ड पेशेन्ट हैं?<br>*(Do not prompt कृप्या विकल्प ना बतायें)*<br>1="Textbook case" किताबी केस 2=Refused to take injection इंजेक्शन लेने से मना करना 3=Refused to have temperature taken तापमान मापने से मना करना 4=Did not look like a real patient असली रोगी की तरह नहीं लग रहा था 96=Other अन्य<br>(For every other, please fill in what the doctor says in 1 line प्रत्येक अन्य के लिए कृप्या डॉक्टर का दिया गया जवाब एक वाक्य में लिखें) ____________________	|_|_|	|_|_|	|_|_|	|_|_|	|_|_|
12	For the interviewer: Please note, based on the doctor's presentation did you think that the doctor actually recognized the standardized patient?<br>इन्टरव्यूर के लिए कृप्या ध्यान दें कि डॉक्टर ने जो भी बताया उस आधार पर क्या आपको लगता है कि उसने स्टैंडर्डाइज़्ड पेशेन्ट को पहचान लिया था।<br>1= YES. Doctor recognized DURING VISIT हाँ, डॉक्टर ने बातचीत के दौरान पहचान लिया<br>2= YES, Doctor recognized AFTER VISIT हाँ, डॉक्टर ने पेशेन्ट के जाने के बाद पहचाना<br>3=NO, Doctor did not recognize नहीं, डॉक्टर ने नहीं पहचाना	|_|	|_|	|_|	|_|	|_|

QuTub Study–Follow up Detection and Vignettes Module Provider ID: ☐☐☐☐☐☐☐

Section 2. Common Illnesses सामान्य बीमारियाँ			
1	ऐसी कौन-सी तीन सामान्य बीमारियाँ हैं जो इस क्षेत्र में आप देखते हैं?<br>What are the 3 most common illnesses that you see in this area?		☐☐<br>☐☐<br>☐☐
	खाँसी/जुखाम Cough/Cold	1	
	डायरिया Diarrhea	2	
	पेचिश Dysentery	3	
	बुखार Fever	4	
	ट्युबरक्लोसिस (टी बी) Tuberculosis	5	
	निमोनिया Pneumonia	6	
	टायफाइड Typhoid	7	
	मलेरिया Malaria	8	
	कार्डियोवैसक्यूलर रोग (हार्ट अटैक, स्ट्रोक)<br>Cardiovascular Disease (heart attack, stroke)	9	
	यौन जनित संक्रामक रोग (इसमें एच आई वी/एड्स शामिल हैं)<br>Sexually Transmitted Disease (including HIV/AIDS)	10	
	महिलाओं से संबंधी यौन समस्यायें Gynaecological problems	11	
	अन्य (विवरण)<br>Other (Specify)	12	हिन्दी<br>*English*
		13	हिन्दी<br>*English*
		14	हिन्दी<br>*English*
2	आपके ज्यादातर मरीज़ कहाँ से आते हैं?<br>Where do the majority of your patients come from? (allowed to prompt)		☐
	इस क्षेत्र से This area	1	
	दूसरे क्षेत्र से Another area	2	
	नहीं जानते/कह नहीं सकते Don't know/cant say	-77	
3	आपको क्या लगता है कि आपके मरीज़ कितनी अच्छी तरह से अपनी बीमारी और लक्षणों के बारे में बता पाते हैं? विकल्प पढ़ें।<br>How well do you think that your patients are able to convey their illness and symptoms? READ OPTIONS	☐	☐
	बहुत अच्छी तरह से Very well	1	
	अच्छी तरह से Well	2	
	खराब तरह से Poorly	3	
	बहुत खराब तरह से Very poorly	4	

QuTub Study–Follow up Detection and Vignettes Module Provider ID: ☐☐☐☐☐☐☐

Section 3 : सेवशन

केस Case : I – रवि/रेखा Ravi/Rekha

मान लीजिये एक 35 साल के पुरुष/महिला हैं जो आपके पास आये हैं। यह आपके सारे सवालों का जवाब देगें और सारी दवाईयाँ लेंगे जो आप बतायेंगे। यह आपके सभी निर्देशों का पालन करेंगे और यदि आपको जरुरत हुई तो वापस भी आयेंगे। ऐसे मरीज के सारे शारीरिक परीक्षण और टेस्ट के रिजल्ट हमारे पास पहले से ही मौजूद हैं।

Observer: A 35 year old man/woman comes to you. They will comply with all tests and medications that you recommend and will return to you if you require. We have the results of any physical examination or test you may require.

रोगी : डॉक्टर साहब, "मुझे खाँसी बहुत हो रही है और साथ में बुखार भी है, जो ठीक ही नहीं हो रहा है"

Patient: "Doctor, I have cough and fever that is not getting better"

केस शुरू होने का समय<br>Case start Time:	H H ☐☐ : M M ☐☐

H	इतिहास History		
प्रश्न नम्बर Q. No.	प्रश्न का प्रकार<br>Type of Question	प्रोवाइडर ने Provider पूछा Asked=1 नहीं पूछा Did not ask = 2	रोगी के जवाब<br>Patient Response
H1	आपका नाम क्या है?<br>What is your name?		रवि/रेखा<br>Ravi/Rekha
H2	खाँसी कब से हो रही है?<br>What is the duration of cough?	☐	2-3 सप्ताह से,<br>2-3 weeks,
H3	क्या आपको बलगम बनती है?<br>Are you producing sputum (bulgam)?	☐	हाँ।<br>Yes
H4	क्या आपके बलगम में खून आता है?<br>Is there any blood in the sputum?	☐	नहीं।<br>No
H5	क्या खाँसी सारा दिन रहती है?<br>Do you have cough throughout the day?	☐	यह सुबह-सुबह और रात को ही ज्यादा होती है।<br>More during early morning and night.
H6	आपको बुखार कब से है?<br>How long have you had fever?	☐	2-3 हफ्ते हो गये।<br>Since 2-3 weeks
H7	बुखार कैसा रहता है?<br>What type of fever do you have?	☐	हल्का बुखार चढ़ता उतरता रहता है, लेकिन ज्यादातर शाम को होता है।<br>Low grade (mild), on and off, more during evening times.
H8	क्या रात में पसीना आता है?<br>Are there any night sweats present?		हाँ<br>Yes
H9	क्या छाती में दर्द होता है?<br>Is there any pain in the chest?	☐	नहीं।<br>No
H10	भूख कैसी लगती है?<br>How is your appetite?	☐	भूख तो कम लगती है।<br>loss of appetite.
H11	क्या वजन कम हुआ है?<br>Have you lost weight?	☐	हाँ, मुझे लगता है मेरा वज़न कम हुआ है क्योंकि मेरे कपड़े ढीले हो गये हैं।<br>Yes. I think I have lost weight as my clothes have become a bit loose.
H12	क्या साँस लेने में कोई तकलीफ है?<br>Do you have any difficulty in breathing?	☐	नहीं।<br>No.
H13	क्या साँस लेते समय सीटी जैसी आवाज आती है?<br>Do you have any wheezing?	☐	नहीं।<br>No.
H14	क्या आपने इस तकलीफ के लिये कोई दवाई ली है?<br>Have you taken any medicines for your illness?	☐	नजदीक के कैमिस्ट के पास गया था उसने मुझे खाँसी का सिरप और बुखार के लिये कुछ गोलियाँ दी थी।<br>Went to a local chemist who gave cough syrup and some pills for fever.
H15	क्या आपको पहले कभी टी बी हुई है?<br>Have you had TB in the past?	☐	नहीं।<br>No.

QuTub Study–Follow up Detection and Vignettes Module Provider ID: ☐☐☐☐☐☐☐

H16	क्या आपने पहले कभी टी बी का इलाज कराया है?<br>Have you been treated for TB in the past?		नहीं तो ।<br>No.
H17	क्या आपके परिवार में किसी को कभी टी बी हुई है?<br>Has any one in your family had TB in the past?	☐	नहीं।<br>No.
H18	क्या आप अपने रोजमर्रा के काम कर लेते हैं?<br>Can you perform your normal activities?		हाँ।<br>Yes
H19	क्या आपको कोई बदनदर्द या सरदर्द हैं<br>Is there any bodyache or headache?		नहीं।<br>No.
H20	क्या आपको चक्कर आते हैं?<br>Any dizziness?		नहीं।<br>No.
H21	क्या आपको कंपकंपी लगती हैं?<br>Are there any chills or rigors?		नहीं।<br>No.
H22	क्या आपको शुगर हैं?<br>Are you diabetic?	☐	नहीं।<br>No.
H23	क्या आपको हाई बी पी/हाईपरटेंशन हैं?<br>Do you have High B P/Hypertension?	☐	नहीं।<br>No.
H24	क्या प्रोवाइडर ने एच आई वी–एड्स के बारे मे पूछा?<br>Did the provider ask anything about HIV-AIDS?	☐	नहीं।<br>No.
H25	क्या आपके गले में खराश/दर्द हैं?<br>Any irritation/pain in the throat?		नहीं।<br>No.
H26	क्या आप शराब पीते हैं?<br>Do you drink alcohol?		हाँ।<br>Yes.
H27	कितनी बार पी लेते हो?<br>How often do you drink alcohol?		महीने में एक–दो बार।<br>Once or twice in a month. [in case of male]<br>No [in case of female SP]
H28	क्या आप बीड़ी/सिगरेट पीते हैं?<br>Do you smoke?		हाँ, मैं बीड़ी पीता हूँ। (मेल SP के लिये)<br>नहीं (फीमेल SP के लिये)<br>Yes, I smoke beedis. [in case of male SP]<br>No [in case of females SP]
H29	एक दिन में कितनी पीते हो?<br>How many beedis in a day?		अंदाजन, 4 से 5 बीड़ी।<br>4-5 beedis, I guess
H30	कब से बीड़ी पी रहे हो?<br>Since when have you been smoking beedis?		पिछले 8 या 10 सालों से।<br>Since the last 8 or 10 years
H31	आप आमतौर क्या खाना खाते हो?<br>What is your normal diet?		चावल सब्जी और कभी–कभी माँस/मछली<br>Rice, vegetables and sometimes meat / fish
H32	आपकी लैट्रिंग कैसी है?<br>How is the stool?		सामान्य<br>Normal
H33	आपकी उम्र क्या हैं?<br>What is your age?	☐	35 साल<br>35 years old

QuTub Study–Follow up Detection and Vignettes Module Provider ID: ☐☐☐☐☐☐☐

E	उपयुक्त परीक्षण प्रोवाइडर कर रहा है<br>Relevant Examinations Provider Conducting	1= हाँ Yes<br>2= नहीं No	परिणाम<br>Results
E1	लम्बाई/कद Height	☐	Males : 170 cms , Females : 160 cms
E2	वज़न Weight	☐	Males : 55-60 kgs , Females : 50-55 kgs
E3	नब्ज़ की दर Pulse Rate	☐	80/minutes
E4	ब्लड प्रेशर Blood Pressure	☐	120-80 mmhg
E5	बुखार/तापमान Temperature	☐	99.5 degrees centigrade (low fever)
E6	ह्रदय, फेफड़ों की गति को सुनना Auscultation	☐	No specific sounds/ mild crepitations in the upper part of the chest.
E7	आँखों का परीक्षण (पीलिया के संकेत/खून की कमी के लिये)<br>Check eyes (including for signs of jaundice/anemia)		Mild Paleness
E8	संक्रमण के लिये मुँह का परीक्षण (कैन्डीडियासिस शामिल हैं)<br>Check mouth for Infection (including Candidiasis)		Negative
	उपयुक्त परीक्षण प्रोवाइडर ने करवाने के लिये कहा<br>Relevant Examinations Provider Ordering		
E9	छाती का एक्स-रे<br>Chest X-Ray	☐	छाती के दाईं तरफ धब्बा<br>Opecity in the right apex
E10	मॉनटूक्स ट्युबरक्युलिन स्किन टेस्ट<br>Mantoux Tuberculin Skin Test (TST)	☐	पॉजिटिव Positive (Induration more than 15mm in diameter after 72 hours)
E11	स्पूटम स्मियर एक्जामिनेशन<br>Sputum smear examination (Sputum AFB)	☐	पॉजिटिव<br>Positive
E12	स्पूटम जीनएक्सपर्ट टेस्ट<br>Sputum-GeneXpert test	☐	MTB-Detected organisms are not resistant to rifampicin bacilli load-medium
E13	स्पूटम कल्चर और ड्रग सस्सेप्टिबिलिटी टेस्ट<br>Sputum culture test and Drug susceptibility test	☐	Culture grows MTB and sensitive to INH & Rifampicin
E14	रक्त – हीमोग्लोबिन<br>Blood – Hemoglobin (Hb)		13 g/dl for males and 12 g/dl for females
E15	रक्त – टोटल काउंट, डिफरेंशियल काउंट–ई एस आर<br>Blood -Total Count, Differential Count- ESR	☐	T C - Total Count : 10,000 cells/cu.mm<br>D C - Differential Count<br>Neutrophils 50%<br>Lymphocytes 40%<br>Eosinophils 5%<br>Monocytes 4%<br>Basophils 1%<br>ESR= 30mm/hr
E16	रक्त – एच आईवी टेस्ट<br>Blood- HIV test	☐	Sero-Negative
E17	रक्त – शुगर टेस्ट टेस्ट<br>Blood- Diabetes test	☐	Fasting blood sugar- 80 mg/dl<br>Post prandial blood sugar- 130 mg/dl<br>Random blood sugar levels: 120 mg/dl
E18	रक्त – टी बी गोल्ड टेस्ट<br>Blood-TB Gold test	☐	पॉजिटिव<br>Positive
E19	रक्त – टी बी ऐलाइज़ा<br>Blood-TB Elisa test	☐	पॉजिटिव<br>Positive
E20	रक्त – टायफायड टेस्ट<br>Blood-Typhoid Test		Negative
E21	रक्त – MP (मलेरिया का परजीवी) टेस्ट<br>Blood-MP (Malarial Parasite) Test		Negative- Malarial Parasite
E22	रक्त – विडाल टेस्ट<br>Blood-Widal Test		Negative (titres : Salmonella typhi O and H agglutinin titres < 1:80 and < 1:160 respectively
E23	रक्त –कल्चर रिपोर्ट<br>Blood- culture report		Negative

QuTub Study–Follow up Detection and Vignettes Module Provider ID: ☐☐☐☐☐☐☐

क्या प्रोवाइडर ने कोई अन्य हिस्ट्री और परीक्षण के नये प्रश्न पूछें जो कि पहले नहीं पूछें गये, तो उन्हें नीचे नोट करें।
Did the provider ask any other history and examination questions that were not mentioned above? Please note them down?

प्रश्न का प्रकार : Question Type: इतिहास History = 1 टेस्ट/परीक्षण Test/ Examination = 2	प्रश्न Question	आपका जवाब (जवाब देने के लिए अपने स्वयं के अनुभव का इस्तेमाल करें) Your Response (use your own experience to answer)
☐	*हिन्दी*	*हिन्दी*
	English	*English*
☐	*हिन्दी*	*हिन्दी*
	English	*English*
☐	*हिन्दी*	*हिन्दी*
	English	*English*
☐	*हिन्दी*	*हिन्दी*
	English	*English*
☐	*हिन्दी*	*हिन्दी*
	English	*English*

टिप्पणी : यदि आपने डॉक्टर को हिस्ट्री या परीक्षण संबंधी प्रश्न का भूलवश गलत उत्तर दे दिया है तो कृप्या लिखें।
Comments: (please write if you accidently gave the doctor an incorrect response to one of the history or examination questions)

PT1		बीमारी से ग्रस्त : Suffering From
	डॉक्टर इस क्षेत्र में जिस प्रकार की परिस्थितियाँ हैं और जिस प्रकार के मरीज़ आप आमतौर पर देखते हैं, उसके आधार पर आपकी राय में आप क्या कहेंगे कि मरीज़ को क्या बीमारी है। उसे लिखें और फिर कोड करें। *Doctor, given the conditions in this area and the kinds of patients you normally see, in your view, what would you say the patient is suffering from?* Write down and then code as : पूरी तरह से सही Fully Correct = 1 आंशिक रूप से सही Partially Correct = 2 गलत Incorrect = 3 (सही जवाब है ट्युबरक्लोसिस होने की संभावना/शंका) (Correct response is 'Tuberculosis')	*हिन्दी* *English* ☐ सही Correct:

A64

QuTub Study–Follow up Detection and Vignettes Module Provider ID:

यदि डॉक्टर ने आगे और कोई प्रश्न नहीं पूछा, तो उससे पूछें कि वो किस उपचार की सलाह देंगे

If the doctor has no further questions, ask him what treatments would you recommend?

TREATMENT (In case to be taken SOS code -66 in frequency)

T1	Sr.No.	(a) Medicine Name दवाईयों के नाम If less than 6 medicines were mentioned then fillup the blanks rows with -99 यदि प्रोवाइडर ने 6 से कम दवाईयाँ दी है तो खाली जगह पर -99 भरें	(b) Mg/Ml of Medicines दवाईयाँ मिग्रा/मिली में	(c) Types of Medicine दवाईयों का प्रकार Tablets गोली=1, Capsules कैप्सूल=2, Syrups सिरप=3, Injectables इंजेक्टेबल=4 Powder चूर्ण=5	(d) Dose खुराक	(e) Frequency दिन में कितनी बार	(f) Duration कितने दिनों तक	(g) How many days in week हफ्ते में कितनी बार	(h) How many weeks कितने हफ्ते	(i) Drug classification code दवा का कोड refer to annex
	1.	ब्रैण्ड Brand<br>जेनेरिक Generic								
	2.	ब्रैण्ड Brand<br>जेनेरिक Generic								
	3.	ब्रैण्ड Brand<br>जेनेरिक Generic								
	4.	ब्रैण्ड Brand<br>जेनेरिक Generic								
	5.	ब्रैण्ड Brand<br>जेनेरिक Generic								
	6.	ब्रैण्ड Brand<br>जेनेरिक Generic								

Annex to T1 (i) Drug classification code :- Unlabelled Tablets/Syrup खुली/बिना नाम की गोलियाँ/सिरप=1; Unlabelled injections खुली या बिना नाम का इंजेक्शन =2; IV bottles/glucose drip आई वी बोतल/ग्लूकोस ड्रिप =3; Ayurvedic medicines आयुर्वेदिक दवाईयाँ =4; Homeopathic medicines होम्योपेथिक दवाईयाँ =5; Antibiotics ऐन्टिबायोटिक दवाईयाँ =6; Analgesics ऐनालजेसिक दवाईयाँ =7; Anti-ulcer medication ऐन्टी-अल्सर दवाईयाँ =8; Steroids (NSAIDS) स्टेरॉयड्स =9; Anti-allergy medicines ऐन्टी-अलर्जिक दवाईयाँ =10; Cardiac medication कारडियक दवाईयाँ =11 ; Psychiatric/ neural medicines साईकाईट्रिक दवाईयाँ =12; Identified as anther type of medication =13; Household remedies घरेलू दवाईयाँ =14; Drugs not classified दवाई जो क्लासिफाइड नहीं है = 50

Kwan A, *et al. BMJ Global Health* 2019; 4:e001669. doi: 10.1136/bmjgh-2019-001669

QuTub Study–Follow up Detection and Vignettes Module Provider ID:

कृप्या पूछें ना, सिर्फ नोट करें<br>DO NOT ASK, NOTE OBSERVATION			1= हाँ Yes<br>2= नहीं No
T2	बीमारी के किसी खतरे के निशान के बारे में सलाह दी?	Any recommendations on of the danger signs of the disease?	
T3	दवाई के साईड इफैक्ट (मिचली, उल्टी, पेशाब का लाल होना) के बारे में बताया?	Any recommendations on side effects of drugs? (nausea, vomiting, red discoloration of the urine)	
T4	नियंत्रण के संबंध में किसी प्रकार की सलाह (बीमारी को फैलने से रोकने के लिए दूसरों से ना मिलना)	Any recommendations on containment (not interacting with others in order to stop the spread of disease)	
T5	धूम्रपान बंद करने की सलाह दी?	Any recommendations on smoking cessation?	
T6	वापस आने की सलाह दी?<br>यदि हाँ, तो T6a से T6e भरे। यदि नहीं, तो T7 पर जायें।	Any recommendations to come back?<br>If yes, mark from T6a to T6e and if no, go to T7	
T6a	लक्षणों में कोई सुधार नहीं	If the symptoms persist	
T6 b	लक्षण और बिगड़ जायें	If the symptoms become worse	
T6c	दवाई लेने के लिये	To get medicines	
T6d	अन्य	Other	
T6e	विवरण___________________________	Specify___________________________	
T7	क्या प्रोवाइडर ने रोगी को आगे की देखभाल के लिए रेफर की सलाह दी? यदि हाँ तो T7a से T7d भरें। यदि नहीं, तो PT2 पर जायें।	Did the provider refer the patient to go anywhere for further management? If yes fill T7a to T7d, If no, go to PT2.	
T7a	प्राइवेट प्रोवाइडर/प्राइवेट अस्पताल	Private Provider/ Private Hospital	
T7b	सरकारी अस्पताल	Government Hospital	
T7c	डॉट्स सेन्टर	DOTS Centre	
T7d	अन्य विवरण___________________________	Other Specify ___________________________	

PT2 & PT3 (तब ही पूछें यदि प्रावाइडर ने ट्युबरक्लोसिस डायग्नोस किया हो और उसका इलाज़ करता हो।)<br>PT2 & PT3 (ASK ONLY IF DIAGNOSIS WAS TUBERCULOSIS AND HE IS GIVING THE TREATMENT)			
PT2	आप इसके लिए कितने समय तक इलाज़ करेंगे?<br>(यदि जरूरी हो तो रेंज लिखें।)	*How long will you give the treatment for?*<br>(Record range if necessary)	Days -- to days
PT3	केवल तब ही पूछें जब थूक/बलगम के परीक्षण और एक्स-रे के लिए सलाह दी हो। क्या आप थूक/बलगम का परीक्षण और एक्स-रे दोबारा करवायेंगे।	ASK ONLY IF SPUTUM TEST AND CHEST X-RAY WERE RECOMMENDED<br>*Will you have the sputum test and Chest X-ray repeated?*	

A66

QuTub Study–Follow up Detection and Vignettes Module Provider ID: ☐☐☐☐☐☐☐

PT4	*तब ही पूछें यदि प्रोवाइडर ने ट्युबरक्लोसिस डायग्नोस नहीं किया।* हमें यह बताया गया है कि इस तरह के लक्षण ट्युबरक्लोसिस के भी होते हैं, यदि इस मरीज़ को ट्युबरक्लोसिस होता तो आप क्या इलाज़ देते।	**ASK ONLY IF DIAGNOSIS WAS NOT TUBERCULOSIS** *We have been told that sometimes these cases could also be tuberculosis. If this patient had tuberculosis, what treatment would you give?*

TREATMENT (In case to be taken SOS code -66 in frequency)

Sr.No.	(a) Medicine Name दवाईयों के नाम If less than 6 medicines were mentioned then fillup the blanks rows with -99 यदि प्रोवाइडर ने 6 से कम दवाईयाँ दी है तो खाली जगह पर -99 भरें	(b) Mg/Ml of Medicines दवाईयाँ मिग्रा/मिली में	(c) Types of Medicine दवाईयों का प्रकार Tablets गोली=1, Capsules कैप्सूल=2, Syrups सिरप=3, Injectables इंजेक्शन=4 Powder चूरन=5	(d) Dose खुराक	(e) Frequency दिन में कितनी बार	(f) Duration कितने दिनों तक	(g) How many days in week हफ्ते में कितनी बार	(h) How many weeks कितने हफ्ते	(i) Drug classification code दवा का कोड refer to annex
1	ब्रांड Brand		☐	☐☐:☐☐	☐☐	☐☐	☐	☐☐	☐☐
	जेनेरिक Generic								
2	ब्रांड Brand		☐	☐☐:☐☐	☐☐	☐☐	☐	☐☐	☐☐
	जेनेरिक Generic								
3	ब्रांड Brand		☐	☐☐:☐☐	☐☐	☐☐	☐	☐☐	☐☐
	जेनेरिक Generic								
4	ब्रांड Brand		☐	☐☐:☐☐	☐☐	☐☐	☐	☐☐	☐☐
	जेनेरिक Generic								
5	ब्रांड Brand		☐	☐☐:☐☐	☐☐	☐☐	☐	☐☐	☐☐
	जेनेरिक Generic								
6	ब्रांड Brand		☐	☐☐:☐☐	☐☐	☐☐	☐	☐☐	☐☐
	जेनेरिक Generic								

QuTub Study–Follow up Detection and Vignettes Module Provider ID:

PT5	क्या दवाई के साईड इफैक्ट (मिचली, उल्टी, पेशाब का लाल होना) के बारे में रोगी को बतायेंगे?	Would you provide any recommendations on side effects of drugs? (nausea, vomiting, red discoloration of the urine)	☐
PT6	क्या रोग नियंत्रण के संबंध में किसी प्रकार की सलाह देंगे? (बीमारी को फैलने से रोकने के लिए दूसरों से ना मिलना)	Would you provide any recommendations on containment (not interacting with others in order to stop the spread of disease)	☐
PT7	क्या धूम्रपान बंद करने की सलाह देंगे?	Would you provide any recommendations on smoking cessation?	☐
PT8	क्या आप रोगी को आगे की देखभाल के लिए रेफर करेंगे? यदि हाँ तो PT8a से PT8d भरें।	Would you refer the patient to go anywhere for further management? If yes fill PT8a to PT8d.	
PT8a	प्राइवेट प्रोवाइडर/प्राइवेट अस्पताल	Private Provider/ Private Hospital	☐
PT8b	सरकारी अस्पताल	Government Hospital	☐
PT8c	डॉट्स सेन्टर	DOTS Centre	☐
PT8d	अन्य विवरण______________________	Other Specify ____________________	
समाप्ति का समयः End Time:	H H ☐☐ : M M ☐☐		

QuTub Study–Follow up Detection and Vignettes Module Provider ID:

Section 4: Characteristics of facility. क्लिनिक में मौजूद सुविधाएँ

Interviewer read aloud: Now I will ask some questions about the characteristics of your facility.
इन्टरव्यूवर पढ़े अब मैं आपसे कुछ सवाल क्लिनिक में मौजूद सुविधाओं के बारे में पूछूँगा

1	Is this facility able to do chest X-rays?<br>क्या आपके क्लिनिक में एक्स-रे करने की सुविधा है?	1=Yes हाँ<br>2=No नहीं	
2	Does this facility have a lab?<br>क्या आपके क्लिनिक में लैब है?	1=Yes हाँ<br>2=No नहीं (if no,skip to question 4; यदि नही, तो प्रश्न 4 पर जायें)	
3	Can you run the following tests at this facility's lab?<br>क्या आपके क्लिनिक में नीचे लिखे टेस्ट करने की सुविधा है?<br><br>*(Answer each of the following with–**प्रत्येक टेस्ट के आगे 1=Yes हाँ, 2=No नहीं लिखें**)*	A. Blood tests (ESR, TLC/DLC, Blood Smear) रक्त – ई एस आर, टोटल काउंट, डिफरेंशियल काउंट रक्त सीमियर	
		B. Sputum tests स्पूटम जीनएक्सपर्ट टेस्ट	
		C. Urinalysis पेशाब की जाँच	
		D. Stool analysis लेट्रिंन की जाँच	
		E. Other, specify अन्य कोई की जाँच______________	
4	Does this facility collect samples from patients and sends them to another location for processing?<br>क्या आप अपने क्लिनिक में रोगी का सेम्पल लेकर आगे किसी दूसरे जगह (लैब) में जाँच के लिए भेजते हैं	1=Yes हाँ<br>2=No नहीं (if no, skip to question 5; यदि नही, तो प्रश्न 5 पर जायें)	
5	What samples do you collect at this facility and send to another location? आप इस क्लिनिक में किस तरह के सेम्पल लेकर आगे किसी दूसरी जगह (लैब) में जाँच के लिए भेजते हैं<br><br>*(Answer each of the following with–**प्रत्येक टेस्ट के आगे 1=Yes हाँ, 2=No नहीं लिखें**)*	A. Blood tests (ESR, TLC/DLC, Blood Smear) रक्त – ई एस आर, टोटल काउंट, डिफरेंशियल काउंट रक्त सीमियर	
		B. Sputum tests स्पूटम जीनएक्सपर्ट टेस्ट	
		C. Urinalysis पेशाब की जाँच	
		D. Stool analysis लेट्रिंन की जाँच	
		E. Other, specify अन्य कोई की जाँच____________	

Kwan A, *et al. BMJ Global Health* 2019; 4:e001669. doi: 10.1136/bmjgh-2019-001669

QuTub Study–Follow up Detection and Vignettes Module Provider ID: |__|__|__|__|__|__|__|

Section 5: Knowledge of Tuberculosis टी बी के बारे में जानकारी

Interviewer read aloud: Doctor, as you know, these days there is a lot of concern about TB. We are asking doctors about how they diagnose and treat TB, since we understand that depending on the patient different tests may be better or worse and different medicines may work better or worse. At this stage, we are only trying to understand what are the ways in which TB patients may be diagnosed, and what tests and medicines work well for you.

इन्टरव्यूवर पढ़े : डॉक्टर जैसे की आप जानते है कि आजकल टी बी की बीमारी को लेकर काफी चिन्ता है। हम डॉक्टरो से पूछ रहे है कि वो टी बी को कैसे डायग्नोज़ करते है और फिर उसका क्या इलाज करते है, क्योकि हमारी समझ से हर रोगी को अलग–अलग तरह के जॉंचो की जरूरत होती है जो उसके लिये अच्छी और बुरी हो सकती है। उसी तरह से दवाईयों का असर भी अच्छा और बुरा हो सकता है। फिलहाल, हम यह समझने की कोशिश कर रहे है कि किन तरीको से टी बी के मरीजों का डायग्नोज़ किया जाता है और आपकी समझ से कौन सी जॉंच और दवाईयॉं उनके लिये ठीक है।

SUSPICION OF TB टी बी की शंका

1. When do you suspect pulmonary (lung) TB in a patient?
   आपको कब शंका होती है कि रोगी को पल्मनरी (फेफड़ो की) टी बी है?
   ____
   ____
   ____

2. In the last 1 month, how many patients has this entire clinic suspected for TB?
   पिछले 1 महीने में इस क्लीनिक मे कितने मरीज आये जिनको टी बी होने की आशंका थी?
   |_____||_____||_____| -77 Don't Know/can't say -77 मालूम नहीं /नही बता सकते

3. In the last 1 month, how many patients have you yourself suspected for TB?
   पिछले 1 महीने में आपने कितने मरीज देखे जिनको टी बी होने की आशंका थी?
   |_____||_____||_____| -77 Don't Know/can't say -77 मालूम नहीं /नही बता सकते

DIAGNOSTIC PRACTICES डायग्नोस्टिक प्रैक्टिसिस

4. What diagnostic tests/investigations do you order for persons suspected of pulmonary TB that work well in your opinion? —जिन रोगियो में आपको पल्मनरी टी बी होने की शंका होती है उनको आप कौन से डायग्नोस्टिक टेस्ट/जॉंच कराने की सलाह देते है जो आपकी नजर में उस मरीज़ के लिये सही है। -77 Don't Know/can't say -77 मालूम नहीं/नही बता सकते
   [1]____
   [2]____
   [3]____
   [4]____
   [5]____

A70

QuTub Study–Follow up Detection and Vignettes Module Provider ID:

5. Based on your own experience, which test/ investigation for TB has been most accurate for your patients of the ones you mentioned? –आपके अनुभव के आधार पर टी बी की जाँच के लिये कौन सा टेस्ट/जाँच आपके मरीजों के लिये सबसे सही साबित हुआ है? -77 Don't Know/can't say -77 मालूम नहीं/नही बता सकते?

   ____

6. We understand that in some cases, if patients do not take their medicines regularly, their TB can become more complicated and some of the usual medicines may not work. Among your patients, do you suggest any tests for drug resistant TB?—इसी तरह के कुछ केसो में हमने जाना की यदि मरीज अपनी रेगुलर दवाईयाँ नही लेता तो टी बी की बिमारी और बिगड जाती है और जिसमे टी बी की दवाईयाँ काम नही करती। क्या आप आपने ऐसे मरीजो को ड्रग्स रजिस्टेन्ट टी बी टेस्ट करवाने की सलाह देते है।

   1=Yes हाँ, 2=No नहीं (If no, Skip to 8 यदि नही, तो प्रश्न 8 पर जायें)

7. When would you test for drug resistance?—आप ड्रग्स रजिस्टेन्ट का टेस्ट कब कराते है।

   ____

8. In the last 1 month, how many TB patients have this entire clinic diagnosed with TB?
   पिछले 1 महीने में आपके पूरे क्लीनिक में कितने मरीजो को टी बी डायग्नोज़ किया गया।

   |_____||_____||_____| -77 Don't Know/can't say -77 मालूम नहीं/नही बता सकते

9. In the last 1 month, how many TB patients have you yourself diagnosed?—
   पिछले 1 महीने में आपने अपने क्लीनिक में कितने मरीजो को टी बी डायग्नोज़ किया?

   |_____||_____||_____| -77 Don't Know/can't say -77 मालूम नहीं/नही बता सकते

10. If you diagnose a TB patient, do you notify the patient to public health authorities?—
    अगर आप किसी मरीज को टी बी डायग्नोज़ करते है तो क्या आप उसकी जानकारी सरकारी संस्था को भी देते है?

    1=Yes हाँ, 2=No नहीं

TREATMENT PRACTICES

11. Do you treat TB patients yourself?
    क्या आप टी बी के रोगी का इलाज़ करते हैं ?

    1=Yes हाँ, 2=No नहीं 2 (if no, skip to 14, यदि नही, तो प्रश्न 14 पर जायें)

12. For how long do you treat a patient for TB?
    टी बी के रोगी का आप कितने समय तक इलाज़ करते हैं?

    |_____||_____||_____| toसे |_____||_____||_____| Months or Days महीने और दिनों में

13. For how many TB patients have you initiated treatment in the last one month? (if -77 Don't Know/can't say , then probe further for 6 months to 1year)–पिछले 1 महीने में आपने कितने टी बी के मरीजों का इलाज शुरु किया है? (यदि -77 मालूम नहीं/नही बता सकते, तो पिछले 6 महीने से 1 साल के बारे में पूछें)
    a.|_____||_____||_____|पिछले 1 महीने में last 1 month
    b.|_____||_____||_____|पिछले 6 महीने में last 6 months
    c.|_____||_____||_____|पिछले 1 साल में last 1year
    -77 Don't Know/can't say, skip to question 15 -77 मालूम नहीं/नही बता सकते, तो प्रश्न 15 पर जायें)

A71

QuTub Study–Follow up Detection and Vignettes Module Provider ID: [ ][ ][ ][ ][ ][ ][ ]

14. Where do you send / refer patients for treatment?
आप रोगी को इलाज़ के लिये कहाँ रेफर करते हैं?
____

15. Do you treat patients with drug resistant TB?
आप ड्रग्स रजिस्टेन्ट टी बी वाले रोगियों का इलाज़ करते हैं?
[ ] 1=Yes हाँ, 2=No नहीं 2 (if no, skip to 19, यदि नही, तो प्रश्न 19 पर जायें)

16. What drugs would you prescribe for a patient with drug resistant TB that work well in your patients?
आप ड्रग्स रजिस्टेन्ट टी बी वाले रोगी को क्या दवाई लिखकर देंगे जो उसके लिए अच्छी साबित हो?
[1]____
[2]____
[3]____
[4]____
[5]____

17. For how long do you treat a patient with drug resistant TB?
आप ड्रग्स रजिस्टेन्ट टी बी वाले रोगी का कितने समय तक इलाज़ करेंगे?
|_____||_____||_____| toसे |_____||_____||_____|Months or Days महीने और दिनों में

18. How many TB patients have you initiated on treatment in the last 1 year?
आपने पिछले एक साल में कितने ड्रग्स रजिस्टेन्ट टी बी वाले रोगियों का इलाज़ शुरू किया है?
|_____||_____||_____| (enter response and then skip to question 6, जवाब लिखने के बाद ही सेक्शन 6 पर जायें)

19. Where do you send/refer patients for treatment?
आप रोगी को इलाज़ के लिये कहाँ रेफर करते हैं?
____

20. Have you ever participated in trainings/meetings/ CME on TB diagnosis and treatment?
क्या आपने टी बी की बीमारी और उसके इलाज़ के संबंध में किसी भी ट्रेंनिग/मिटिंग/सीएमई में भाग लिया हैं
[ ] 1=Yes हाँ, 2=No नहीं 2 (if no, skip to Section 6; यदि नही ,तो सेक्शन 6 पर जायें)

21. If yes, then who organized them? यदि हाँ, तो वो किसके द्वारा आयोजित किया गया था?
1= Government सरकार, 2= NGOs गैर सरकारी,
3= Indian Medical Association इंडियन मेडिकल एसोसिएशन, 4= other specify --

22. When was it organized? ये कब आयोजित हुआ था?
[ ][ ]/[ ][ ][ ][ ]
M M Y Y Y Y

Kwan A, *et al. BMJ Global Health* 2019; 4:e001669. doi: 10.1136/bmjgh-2019-001669

QuTub Study–Follow up Detection and Vignettes Module Provider ID: ☐☐☐☐☐☐☐

Section 6: Final section. फाइनल सेक्शन

Interviewer read outloud: Doctor, thank you for the time you spent today answering my questions.

इन्टरव्यूवर पढ़े : डॉक्टर, आज आपने अपना कीमती समय निकालकर हमारे प्रश्नों का जवाब दिया उसके लिए धन्यवाद।

1. Your participation in this project has been very important for us.Could you tell us briefly if there is any problem that you have faced due to your participation? 1=Yes हाँ, 2=No नहीं ☐

If yes, what was the problem? (Interviewer please record comment below)

इस प्रोजेक्ट में आपका सहयोग बहुत महत्वपूर्ण रहा है। क्या आपको इस प्रोजेक्ट में भाग लेने से किसी मुश्किल का सामना करना पडा? (यदि हाँ तो क्या मुश्किलें आयीं? कृप्या संक्षेप मे नींचे लिखें)

____

____

____

2.Do you have any suggestion for improving the project?

क्या आपके इस संबंध में कोई सुझाव हैं, जिससे इस प्रोजेक्ट में और सुधार लाया जा सके?

____

____

____

Kwan A, *et al. BMJ Global Health* 2019; 4:e001669. doi: 10.1136/bmjgh-2019-001669

QuTub Study–Follow up Detection and Vignettes Module Provider ID: ☐☐☐☐☐☐☐

Section 7: Interviewer section. इन्टरव्यूवर सेक्शन

Interviewer instructions: You should complete this section after leaving the clinic.
इन्टरव्यूवर : के लिए निर्देश यह सेक्शन क्लिनिक से बाहर आने के बाद भरें

1. When you entered the clinic, were there people apart from the patient sitting with the provider?
   जब आप क्लिनिक के अन्दर गये, तो क्या वहाँ पर रोगियों के अलावा कोई अन्य व्यक्ति प्रोवाइडर के साथ बैठे थे?
   ☐ 1=Yes हाँ, 2=No नहीं
2. Is there a TV or radio at the clinic? क्या क्लिनिक में रेडियो या टी वी था?
   ☐ 1=Yes हाँ, 2=No नहीं If no, skip to 4, यदि नही, तो प्रश्न 4 पर जायें)
3. Could you here the TV or Radio at any time when you were with the provider? क्या आपने किसी भी समय रेडियो या टीवी की आवाज सुनी जब आप प्रोवाइडर के साथ थे?
   ☐ 1=Yes हाँ, 2=No नहीं
4. Did the doctor answer his/her mobile phone or send text messages while seeing a patient?
   रोगी देखे समय क्या डॉक्टर ने अपने मोबाइल फोन पर बात की/मेसिज़ लिखा?
   ☐ 1=Yes हाँ, 2=No नहीं
5. Was the doctor seeing patients in a private room that was closed from the street or from other patients?
   क्या डॉक्टर रोगी को एक अलग बन्द कमरे में देख रहा था जिसको बाहर से नहीं देख सकते?
   ☐ 1=Yes हाँ, 2=No नहीं
6. List the SPs who visited this provider and also completed the exit interview.
   उन एस पी का नाम लिखें जिसने इस डॉक्टर के साथ अपना केस पूरा किया था?
   Provide the SP IDs. Leave blank if no SP visited.
   एस पी की आई डी लिखें, यदि कोई एस पी नहीं गया है तो उस स्थान को खाली छोड़ दें।
   A. SP1 |____|____|
   B. SP2 |____|____|
   C. SP3 |____|____|
   D. SP4 |____|____|
7. List the SPs who visited this provider and did NOT completed the exit interview.
   उन एस पी का नाम लिखें जिसने इस डॉक्टर के साथ अपना केस पूरा नहीं हो पाया?
   Provide the SP ID's. Leave blank if not applicable.
   एस पी की आई डी लिखें, यदि लागू नहीं होता तो उस स्थान को खाली छोड़ दें।
   A. SP1 |____|____|
   B. SP2 |____|____|
   C. SP3 |____|____|
   D. SP4 |____|____|

Supervision Check सुपरवाइजर चैक			
Checked By<br>किसके द्वारा जाँचा गया		ID आई डी	☐☐
Form checking date<br>फॉर्म चैक करने की तारीख	☐☐/☐☐/☐☐☐☐<br>*DD/MM/YYYY format*		

A74

ANNEX M. 3-WEEK SP TRAINING SCHEDULE

To use this annex as a whole, or in part, please cite:

- Kwan A, Bergkvist S, Daniels B, Das J, Das V, Pai M. Using Standardized Patients to Measure Health Care Quality: A Manual and Toolkit for Projects in Low- and Middle-Income Countries. 2019.
- Daniels B, Dolinger A, Bedoya G, Rogo K, Goicoechea A, Coarasa J, Wafula F, Mwaura N, Kimeu R, Das J. Use of standardised patients to assess quality of healthcare in Nairobi, Kenya: a pilot, cross-sectional study with international comparisons. BMJ global health. 2017 Jun 1;2(2):e000333.

	Week 1						Week 2						Week 3				
	MONDAY	TUESDAY	WEDNESDAY	THURSDAY	FRIDAY	SATURDAY	MONDAY	TUESDAY	WEDNESDAY	THURSDAY	FRIDAY	SATURDAY	MONDAY	TUESDAY	WEDNESDAY	THURSDAY	FRIDAY
9.00 - 9.30	SP Introduction	Introduction to Group Work	Group work: Script and Narrative Development	Group reenactment of scripts and SP cases	Introduction to Exit Questionnaire	Exit Questionnaire	Introduction to Mock Interviews	Mock interviews	Mock interviews for SP cases with practice recall questions	Mock interviews	Quality of care introduction	Mock interviews	Mock interviews to practice recall questions	Dry runs for the team	Dry runs for the team	Dry runs for the team	Final debriefing of the team
10.00 - 10.30	Review of SP cases	Review of SP cases in groups			Exit Questionnaire		Mock interviews				Mock interviews						
10.30 - 11.00																	
11.00 - 11.30																	
11:30 - 11:44	TEA/ COFFEE BREAK	TEA/ COFFEE BREAK	TEA/ COFFEE BREAK	TEA/ COFFEE BREAK	TEA/ COFFEE BREAK	TEA/ COFFEE BREAK	TEA/ COFFEE BREAK	TEA/ COFFEE BREAK	TEA/ COFFEE BREAK	TEA/ COFFEE BREAK	TEA/ COFFEE BREAK	TEA/ COFFEE BREAK	TEA/ COFFEE BREAK	TEA/ COFFEE BREAK	TEA/ COFFEE BREAK	TEA/ COFFEE BREAK	TEA/ COFFEE BREAK
11:44 - 12:00	Review of SP cases	Review of SP cases in groups	Group work: Script and Narrative Development	Risk Mitigation Strategies (Thermometers, invasive exams, lab tests, treatments given, avoiding detection)	Exit Questionnaire	Exit Questionnaire	Mock interviews	Mock interviews	Mock interviews for SP cases with practice recall questions	Mock interviews for SP cases	Mock interviews	Mock interviews	Mock interviews to practice recall questions	Dry runs for the team	Dry runs for the team	Dry runs for the team	Final debriefing of the team
12.00 - 12.30																	
12.30 - 13.00																	
13.00 - 13.30	LUNCH	LUNCH	LUNCH	LUNCH	LUNCH	LUNCH	LUNCH	LUNCH	LUNCH	LUNCH	LUNCH	LUNCH	LUNCH	LUNCH	LUNCH	LUNCH	LUNCH
13.30 - 13.44																	
13:44 - 14:00	Review of SP cases	Group work: Script and Narrative Development	Group work: Script and Narrative Development	Risk Mitigation Strategies	Exit Questionnaire	Exit Questionnaire	Mock interviews	Mock interviews	Mock interviews for SP cases with improvisation questions practice	Mock interviews for SP cases	Training on audio recorders	Mock interviews to practice recall questions	Mock interviews to practice recall and improvisation questions	Debriefing of the teams after dry runs	Debriefing of the teams after dry runs	Debriefing of the teams after dry runs	Last round of mock interviews practice recall and improvisation questions
14.00 - 14.30																	
14.30 - 14.00											Mock interviews to practice recall questions						
14.00 - 14.30																	
14:30 - 14:44	TEA/ COFFEE BREAK	TEA/ COFFEE BREAK	TEA/ COFFEE BREAK	TEA/ COFFEE BREAK	TEA/ COFFEE BREAK	TEA/ COFFEE BREAK	TEA/ COFFEE BREAK	TEA/ COFFEE BREAK	TEA/ COFFEE BREAK	TEA/ COFFEE BREAK	TEA/ COFFEE BREAK	TEA/ COFFEE BREAK	TEA/ COFFEE BREAK	TEA/ COFFEE BREAK	TEA/ COFFEE BREAK	TEA/ COFFEE BREAK	TEA/ COFFEE BREAK
14:44 - 16:00	Review of SP cases	Group work: Script and Narrative Development	Group work: Script and Narrative Development	Risk Mitigation Strategies	Exit Questionnaire	Exit Questionnaire	Mock interviews	Mock interviews	Mock interviews for SP cases with improvisation questions practice	Mock interviews for SP cases	Mock interviews to practice recall questions	Mock interviews to practice recall questions	Mock interviews to practice recall and improvisation questions	Debriefing of the teams after dry runs	Debriefing of the teams after dry runs	Debriefing of the teams after dry runs	Last round of mock interviews practice recall and improvisation questions
16.00 - 16.30																	
16.30 - 17.00																	

ANNEX N. SUPERVISOR FIELDWORK SCHEDULE – EXAMPLE (SECTION 9.2)

To use this annex as a whole, or in part, please cite:

- Kwan A, Bergkvist S, Daniels B, Das J, Das V, Pai M. Using Standardized Patients to Measure Health Care Quality: A Manual and Toolkit for Projects in Low- and Middle-Income Countries. 2019.
- Daniels B, Dolinger A, Bedoya G, Rogo K, Goicoechea A, Coarasa J, Wafula F, Mwaura N, Kimeu R, Das J. Use of standardised patients to assess quality of healthcare in Nairobi, Kenya: a pilot, cross-sectional study with international comparisons. BMJ global health. 2017 Jun 1;2(2):e000333.

SP FIELDWORK SCHEDULE

SPID: |____|____|

SP Name: ________________________________

	SUNDAY	*MONDAY*	*TUESDAY*	*WEDNESDAY*	*THURSDAY*	*FRIDAY*	*SATURDAY*
MORNING	1	2	3<br>Arrival time:<br>Wait time:<br>Facility name:<br>Facility location:<br>Supervisor and Mobile:	(DAY 1)<br>4<br>Arrival time:<br>Wait time:<br>Facility name:<br>Facility location:<br>Supervisor and Mobile:	(DAY 2)<br>5<br>Arrival time:<br>Wait time:<br>Facility name:<br>Facility location:<br>Supervisor and Mobile:	(DAY 3)<br>6<br>Arrival time:<br>Wait time:<br>Facility name:<br>Facility location:<br>Supervisor and Mobile:	(DAY 4)<br>7<br>Arrival time:<br>Wait time:<br>Facility name:<br>Facility location:<br>Supervisor and Mobile:
AFTERNOON/EVENING			Arrival time:<br>Wait time:<br>Facility name:<br>Facility location:<br>Supervisor and Mobile:	Arrival time:<br>Wait time:<br>Facility name:<br>Facility location:<br>Supervisor and Mobile:	Arrival time:<br>Wait time:<br>Facility name:<br>Facility location:<br>Supervisor and Mobile:	Arrival time:<br>Wait time:<br>Facility name:<br>Facility location:<br>Supervisor and Mobile:	Arrival time:<br>Wait time:<br>Facility name:<br>Facility location:<br>Supervisor and Mobile:
MORNIN G	8<br>Arrival time:	(DAY 5)<br>9<br>Arrival time:	(DAY 6)<br>10<br>Arrival time:	(DAY 7)<br>11<br>Arrival time:	(DAY 8)<br>12<br>Arrival time:	(DAY 9)<br>13<br>Arrival time:	(DAY 10)<br>14<br>Arrival time:

Kwan A, *et al. BMJ Global Health* 2019; 4:e001669. doi: 10.1136/bmjgh-2019-001669

	Wait time:<br>Facility name:<br>Facility location:<br>Supervisor and Mobile:	Wait time:<br>Facility name:<br>Facility location:<br>Supervisor and Mobile:	Wait time:<br>Facility name:<br>Facility location:<br>Supervisor and Mobile:	Wait time:<br>Facility name:<br>Facility location:<br>Supervisor and Mobile:	Wait time:<br>Facility name:<br>Facility location:<br>Supervisor and Mobile:	Wait time:<br>Facility name:<br>Facility location:<br>Supervisor and Mobile:	Wait time:<br>Facility name:<br>Facility location:<br>Supervisor and Mobile:
AFTERNOON/EVENING	Arrival time:<br>Wait time:<br>Facility name:<br>Facility location:<br>Supervisor and Mobile:	Arrival time:<br>Wait time:<br>Facility name:<br>Facility location:<br>Supervisor and Mobile:	Arrival time:<br>Wait time:<br>Facility name:<br>Facility location:<br>Supervisor and Mobile:	Arrival time:<br>Wait time:<br>Facility name:<br>Facility location:<br>Supervisor and Mobile:	Arrival time:<br>Wait time:<br>Facility name:<br>Facility location:<br>Supervisor and Mobile:	Arrival time:<br>Wait time:<br>Facility name:<br>Facility location:<br>Supervisor and Mobile:	Arrival time:<br>Wait time:<br>Facility name:<br>Facility location:<br>Supervisor and Mobile:

ANNEX O. SP FIELDWORK SCHEDULE – EXAMPLE (SECTION 9.2)

To use this annex as a whole, or in part, please cite:

- Kwan A, Bergkvist S, Daniels B, Das J, Das V, Pai M. Using Standardized Patients to Measure Health Care Quality: A Manual and Toolkit for Projects in Low- and Middle-Income Countries. 2019.
- Daniels B, Dolinger A, Bedoya G, Rogo K, Goicoechea A, Coarasa J, Wafula F, Mwaura N, Kimeu R, Das J. Use of standardised patients to assess quality of healthcare in Nairobi, Kenya: a pilot, cross-sectional study with international comparisons. BMJ global health. 2017 Jun 1;2(2):e000333.

Kwan A, *et al. BMJ Global Health* 2019; 4:e001669. doi: 10.1136/bmjgh-2019-001669

SPID: 11
SP NAME: ______________________________

FACILITY ID	CASE	SPID	ASSIGNED VISIT 1 DATE DD/MM/YYYY	DAY	SUPERVISOR ID	SUPERVISOR NAME	NAME OF FACILITY	ZONE	FACILITY LOCATION	AVG # OF PATIENTS PER DAY	BUSIEST DAY OF THE WEEK
900001	SP1	11	4/9/20XX	1	S3	[NAME S3]	CLINIC 1	ZONE 1	LOCATION 1	300	MONDAY
900002	SP1	11	4/9/20XX	1	S5	[NAME S5]	CLINIC 2	ZONE 2	LOCATION 2	3	UNPREDICTABLE
900003	SP1	11	5/9/20XX	2	S1	[NAME S1]	CLINIC 3	ZONE 3	LOCATION 3	10	SUNDAY
900004	SP1	11	5/9/20XX	2	S3	[NAME S3]	CLINIC 4	ZONE 4	LOCATION 4	80	MONDAY
900005	SP1	11	6/9/20XX	3	S2	[NAME S2]	CLINIC 5	ZONE 5	LOCATION 5	120	MON,WED,FRI
900006	SP1	11	6/9/20XX	3	S4	[NAME S4]	CLINIC 6	ZONE 6	LOCATION 6	16	MON, TUES
900007	SP1	11	7/9/20XX	4	S4	[NAME S4]	CLINIC 7	ZONE 7	LOCATION 7	22	MON, SUN
900008	SP1	11	7/9/20XX	4	S5	[NAME S5]	CLINIC 8	ZONE 8	LOCATION 8	10	MONDAY
900009	SP1	11	9/9/20XX	5	S4	[NAME S4]	CLINIC 9	ZONE 9	LOCATION 9	6	SATURDAY
900010	SP1	11	9/9/20XX	5	S5	[NAME S5]	CLINIC 10	ZONE 10	LOCATION 10	70	MONDAY
900011	SP1	11	10/9/20XX	6	S1	[NAME S1]	CLINIC 11	ZONE 11	LOCATION 11	5	SATURDAY
900012	SP1	11	10/9/20XX	6	S5	[NAME S5]	CLINIC 12	ZONE 12	LOCATION 12	70	MONDAY
900013	SP1	11	11/9/20XX	7	S1	[NAME S1]	CLINIC 13	ZONE 13	LOCATION 13	100	MONDAY
900014	SP1	11	11/9/20XX	7	S5	[NAME S5]	CLINIC 14	ZONE 14	LOCATION 14		SUNDAY
900015	SP1	11	12/9/20XX	8	S1	[NAME S1]	CLINIC 15	ZONE 15	LOCATION 15	20	NOT SPECIFIC
900016	SP1	11	12/9/20XX	8	S3	[NAME S3]	CLINIC 16	ZONE 16	LOCATION 16	100	MONDAY
900017	SP1	11	13/9/20XX	9	S3	[NAME S3]	CLINIC 17	ZONE 17	LOCATION 17	10	MONDAY
900018	SP1	11	13/9/20XX	9	S5	[NAME S5]	CLINIC 18	ZONE 18	LOCATION 18	130	MONDAY
900019	SP1	11	14/9/20XX	10	S4	[NAME S4]	CLINIC 19	ZONE 19	LOCATION 19	25	WEDNESDAY
900020	SP1	11	14/9/20XX	10	S5	[NAME S5]	CLINIC 20	ZONE 20	LOCATION 20	20	WEEKENDS(SATURDAY)

Kwan A, *et al. BMJ Global Health* 2019; 4:e001669. doi: 10.1136/bmjgh-2019-001669

ANNEX P. SP COMMENTS – EDITED (SECTION 10.4)

Source: KePSIE project, Kenya
Principal Investigators: Jishnu Das, Guadalupe Bedoya
Project period: 2015 – ongoing

To use this annex as a whole, or in part, please cite:

- Kwan A, Bergkvist S, Daniels B, Das J, Das V, Pai M. Using Standardized Patients to Measure Health Care Quality: A Manual and Toolkit for Projects in Low- and Middle-Income Countries. 2019.
- Daniels B, Dolinger A, Bedoya G, Rogo K, Goicoechea A, Coarasa J, Wafula F, Mwaura N, Kimeu R, Das J. Use of standardised patients to assess quality of healthcare in Nairobi, Kenya: a pilot, cross-sectional study with international comparisons. BMJ global health. 2017 Jun 1;2(2):e000333.

Health Facility ID	SP ID	SP Case Type	Comments
9041302	43	Angina	Today I visited a public health facility. I arrived at the facility at 2:23pm and there were 9 patients before me. At the registration, I was not charged anything, and they only took my name, age and place of residence. I was attended to by the provider after waiting for 9 minutes. My consultation lasted 2 minutes and 47 seconds. The provider just asked one question and the entry time. He was just writing and never looked at me or even examined me. The facility is well structured but understaffed. The signage is equally poor and finding the various points is difficult. My provider was also busy on phone and he looked like he was in a hurry. There were few clients, mostly expectant and breastfeeding mothers.
9062304	43	Angina	Today I visited a health facility in [LOCATION]. The facility was a private one. When I arrived, there was only one patient being attended to. I arrived at the facility around 10:35am and was immediately attended to by the receptionist who took my name, age, and place of residence. I was ushered into the doctor's room at 10:45am and my consultation lasted 7 minutes. The provider was not in a hurry since there were very few patients. He left me in the consultation room at some point and returned after a minute. He did front auscultation only and asked me if I was coughing the time I usually wake up, if I smoke and the nature of the pain. He did a diagnosis and told me that I had pneumonia and he wanted to inject me there and then, then I would come back for two others. I declined to be injected by telling him that I do my own business and I was alone at my stall, so I needed to close it up. He had stated to me that I could pay by installments so the issue of not having money did not apply.

9041303	31	Asthma	The provider told me to avoid allergens cats, dogs, dust, carpets. The provider was very concerned and even wanted to do the test even if I had no money. The provider did not want to give a diagnosis until he is done with the test. The provider explained to me what happens to the airways when one is exposed to the allergens. He explained very well what a full hemogram is. He wanted to do the test there and then, but I said that I had no money but will come back in the afternoon. Said he will give me all antibiotics after the test.
9031203	13	Diarrhea	I entered the facility at 12:30pm and left at 12:36pm. There were no other patients in the facility. The provider was very understanding and showed that she was really worried about the baby. She asked me the age, duration, frequency, fever and vomiting. She then said that even though she was really sorry about my baby she couldn't give me any medicine because the baby has to be examined first before being given any medicine. She tried to explain to me more about diarrhea and said that if a child has diarrhea, it should be taken very seriously because she loses a lot of water and it is very risky. She also said that the child should be given a lot of fluids and ORS. If I am able to, I should bring her back to that facility but if I can't manage to bring her back to her, I should take her to the nearest clinic. Generally, I liked her. She didn't even ask for any money from me. To me that means she's not after money.
9062306	13	Diarrhea	I entered the facility at 14:20 and left at 15:25. I spent 5 minutes with the doctor. He tried to explain to me that for a child to have diarrhea 6-7 times a night, the child needs to be seen by a doctor. He also said that he cannot give me any medicine because he doesn't know how the baby is right now. That he must see the child first to know what to do. He also said that there might be need for admission of the baby so he has to see the baby first before he can start any treatment. He advised me to take ORS and go and give it to the baby first when I am still preparing to take her to the hospital. He said that being that it's late I should just take her to the nearest health center when I return home. Generally, he seemed knowledgeable and tried to explain everything well. I liked him as a provider and I would go back to that health center if at all I am sick or if my baby is sick
9012205	11	Diarrhea	The lady nurse was very interested in knowing more about the child and she did ask me to come with the child for her to run more tests as she had a lab. She was the only person at the health facility operating as the receptionist, consultant as well as the pharmacist. The environment at the clinic was very clean and it was not that busy. She was able to explain to me more about the child's health and she was much more concerned about my worries and advised me to bring the child for a check-up. She told me to give the child a lot of boiled water. She asked me to give the child ORS mixed with boiled water (500ml).
9012207	11	Diarrhea	The health provider gave me all the time and explained to me how dangerous it is for a child to have watery diarrhea. He was also able to educate me on how to prepare the ORS and how to give to the child through the day and night. He also told me about how to mix the zinc, advantages of the zinc and how it helps the child. I was given three options: (i) Mix with little breast milk. (ii) Mix with little ORS. (iii) Mix with little boiled water. He also told me to make sure that everything that I use to store water is clean and the water must be boiled. The facility is small, and they have many patients to be seen. He also told me to go home and take care of my child.
9061301	12	Diarrhea	I went to the facility at 9:40am. Waited for 20 minutes. After registering, I went to the triage and insisted that I wanted to see the provider. They allowed me. I went to give my complaint to the provider. She asked me the age, frequency, and duration. She asked me where I live. I told her [LOCATION OF HOME]. She advised me to go back home, come with the child because it was good the child to be checked for temperature and weight. The stool should be investigated to find the cause of diarrhea. She told me to leave the facility immediately and bring the child. I left the facility at 10:09 am.

Kwan A, *et al. BMJ Global Health* 2019; 4:e001669. doi: 10.1136/bmjgh-2019-001669

9062201	24	TB	The facility is a private one and consists of three rooms: waiting bay, pharmacy and consultation room, which also doubles as a triage. There were no patients in the waiting bay when I arrived, and the health service provider ushered me to the consultation room where he went directly into asking me what was wrong with me. No demographics was taken at this stage. After questioning me for a few minutes and carrying out auscultation, he went on to explain that I could be suffering from TB, but he could not rule out brucellosis. He then proceeded to explain me in detail the clinical manifestations of both diseases and their treatment courses. Finally, he dispensed some drugs, which he said would probably cure the condition and asked me to go back in the evening to receive the first of my three injections. In the event that this treatment course failed, he was to follow it up with the sputum test and chest X-ray to ascertain whether I could be suffering from TB. He also asked me whether I was married and what was my occupation. Additionally, he carried out a clinical exam that I have never heard of which involved placing the index finger between two of my ribs and tapping the finger all this while listening for any unusual sounds. The last thing he asked me was my name from then on. He switched on to local [DIALECT] as he put the dispensed drugs in a black paper bag.
9062204	24	TB	The facility is a private one and appears to be managed by only one health service provider. It is partitioned into three rooms - waiting bay (looked more of a corridor), consultation room and a store. The consultation room also served as the pharmacy. The health service provider asked me to take the medication until all is used up except in the case of syrup, which I was supposed to stop taking once I stop coughing. The health service provider asked whether he had seen me before (i.e., in his facility) and when I replied that he hadn't he proceeded to record my name in his register. He didn't ask me how old I was although he remarked that he had never seen me in that area before. Upon noticing that my name was [FROM AN ETHNIC GROUP], he carried on the rest of the consultation in [DIALECT]. In the waiting bay were 9 posters on the wall but only one had a health-related message. The rest were "decorations". The syrup dispensed was poured from a jerry can into a bottle he had just washed in the wash-hand basin.
9062302	24	TB	The facility is a private one and has three separate rooms - the consultation room (which is equipped with an ultra sound machine), waiting bay and the pharmacy. In spite of undertaking general consultations the facility has a [FRANCHISE NAME] clinic, which offers MCH services. In fact, out of all the patients I saw in the facility I was the only man and most of the rest had small babies with them. Although the facility was not "roomy" enough the health service provider interacted with went off her way to ensure that she left nothing to chance by referring me to a nearby facility for TB screening. She did not charge me any consultation fees in spite of my asking her what the charges would be. No clinical exams were carried out nor were any drugs prescribed by the health service provider. She said she would only do so after she had ruled out a TB infection after the screening.
9062304	22	TB	I walked into the facility and there was no one waiting. So, after registration I walked directly to the consultation room. The provider who was a male and did not have a white coat, just casually dressed, asked the questions in regard to my illness then came in a lab technician who the provider consulted about the symptoms of my illness and that I was required to do a sputum test, which they said that it will be done 3 times and that I should bring the first sample tomorrow morning. Then the lab technician left then the provider recommended that I should be given some antibiotic injections and medicines, then again, he recommended for typhoid and another test, which is indicated on the lab request form to be done there and then. I was given two tins labeled 1 and 2. Tin 1 I was to put the sputum any time from now till before bedtime, and tin 2 I was to put the sputum in the morning before doing anything.

9062303	21	TB	When I arrived, I found the receptionist watching news on TV. He directed me to a room, which I was to meet the doctor but to my surprise he came to attend to me. There was no registration done to me but asked my problem. He then asked me the duration of the cough and medication I had used previously. Then he did auscultation and told me that I should get three injections one day each. The cost of medication was 1500. I tried to ask for another option like medicines, but he told me since I had used medicine previously and it did not work there was no need. He insisted on injection and demanded that I should first pay whatever I have and come with the rest. I told him that I live in the next plot and I only wanted to know my problem and can start medication in the afternoon. He dismissed me on grounds that I should come back in the afternoon with some money. To me this provider did not appear to be a doctor but was just a conman that was interested in my money.
9061301	21	TB	The provider asked me only two questions which is cough duration and if I had been treated. He was not interested to know my condition that had brought me to him but decided to send me to the lab for a sputum test. The lab tech was interested a bit because she asked me about people I live with and talked about cough hygiene, which I should do to avoid infecting family members with TB. She gave me 2 test tubes to go and collect sputum and then bring back the next day. I did not like the kind of service offered by the provider and I can't go back to him for treatment. I was able to know the provider's name and qualification from the lab form that was given.

ANNEX Q. SP DATA FILES (SECTION 11)

To use this annex (Q1-Q6) as a whole, or in part, please cite:

- Kwan A, Bergkvist S, Daniels B, Das J, Das V, Pai M. Using Standardized Patients to Measure Health Care Quality: A Manual and Toolkit for Projects in Low- and Middle-Income Countries. 2019.
- Das J, Kwan A, Daniels B, Satyanarayana S, Subbaraman R, Bergkvist S, Das RK, Das V, Pai M. Use of standardised patients to assess quality of tuberculosis care: a pilot, cross-sectional study. The Lancet Infectious Diseases. 2015 Nov 30;15(11):1305-13.
- Kwan A, Daniels B, Saria V, Satyanarayana S, Subbaraman R, McDowell A, et al. Variations in the quality of tuberculosis care in urban India: A cross-sectional, standardized patient study in two cities. PLOS Medicine. 2018;15(9):e1002653.

Q1. Provider universe master code file
Q2. Sample master code file
Q3. Schedule and tracking master code file
Q4. SP staff master code file
Q5. Medicines master code file
Q6. Exit questionnaire master data dictionary file

Kwan A, *et al. BMJ Global Health* 2019; 4:e001669. doi: 10.1136/bmjgh-2019-001669

ANNEX Q1. SP DATA FILES – PROVIDER UNIVERSE MASTER CODE EXAMPLE (SECTION 11.1)

This example file can be accessed at:
https://github.com/qutubproject/using-standardized-patients

ANNEX Q2. SP DATA FILES – SAMPLE MASTER CODE FILE EXAMPLE (SECTION 11.1)

This example file can be accessed at:
https://github.com/qutubproject/using-standardized-patients

ANNEX Q3. SP DATA FILES – SCHEDULE AND TRACKING MASTER CODE FILE EXAMPLE (SECTION 11.1)

This example file can be accessed at:
https://github.com/qutubproject/using-standardized-patients

ANNEX Q4. SP DATA FILES – SP STAFF MASTER CODE FILE EXAMPLE (SECTION 11.1)

This example file can be accessed at:
https://github.com/qutubproject/using-standardized-patients

ANNEX Q5. SP DATA FILES – MEDICINES MASTER CODE FILE EXAMPLE (SECTION 11.1)

This example file can be accessed at:
https://github.com/qutubproject/using-standardized-patients

ANNEX Q6. SP DATA FILES – EXIT QUESTIONNAIRE MASTER DATA DICTIONARY FILE EXAMPLE (SECTION 11.3)

This example file can be accessed at:
https://github.com/qutubproject/using-standardized-patients

Kwan A, *et al. BMJ Global Health* 2019; 4:e001669. doi: 10.1136/bmjgh-2019-001669
